# Targeting the AcrAB-TolC efflux pump by small molecules selectively addressing the outer membrane factor TolC

**DOI:** 10.3389/fphar.2026.1895729

**Published:** 2026-09-24

**Authors:** Janis Veliks, Cristina D. Cruz, Tania Szal, Marina Madre, Irena Leite, Laura Paunina, Philipp Lewe, Maren Hinz, Susanne Witt, Päivi Tammela, Aigars Jirgensons, Björn Windshügel

**Affiliations:** 1 Latvian Institute of Organic Synthesis, Riga, Latvia; 2 Drug Research Program, Division of Pharmaceutical Biosciences, Faculty of Pharmacy, University of Helsinki, Helsinki, Finland; 3 Fraunhofer Institute for Translational Medicine and Pharmacology ITMP, Discovery Research ScreeningPort, Hamburg, Germany; 4 Constructor University, School of Science, Bremen, Germany; 5 Centre for Structural Systems Biology (CSSB), University Medical Center Hamburg-Eppendorf, Hamburg, Germany

**Keywords:** AcrAB-TolC, antimicrobial resistance, *E. coli*, efflux pumps, outer membrane factor, TolC

## Abstract

**Background:**

Gram-negative bacteria express several efflux pump complexes extruding a broad spectrum of antibiotics. The major efflux pump in *E. coli* is the AcrAB-TolC complex that expels a broad spectrum of antibiotics from the bacterium, resulting in impaired efficacy of these drugs. Therefore, the inhibition of efflux pump function is an attractive approach to restore the susceptibility of Gram-negative bacteria for antibiotics.

**Methods:**

A virtual screening campaign was utilized to identify potential TolC binders. Virtual hits were investigated using a functional efflux assay, followed by synergy tests with piperacillin and determination of the intrinsic antibacterial potential. Bindings studies with individual efflux pump proteins were carried out. Synthetic chemistry was applied to improve the efficacy of the compound. Microbiological and biophysical tests were used to characterize the compounds with respect to synergistic activity with antibiotics and efflux pump binding.

**Results:**

We identified a small molecule interfering with efflux pump function, showing synergistic activity with piperacillin and binding to TolC which was further optimized at various positions. The most promising compounds revealed TolC-selective binding and shifted the minimum inhibitory concentration of piperacillin and levofloxacin at low micromolar concentrations.

**Conclusion:**

Our study provides evidence that TolC may represent a valid target for the development of an antibiotic adjuvant.

## 1 Introduction

Antimicrobial resistance (AMR) is a global threat with tremendous implications for human and animal health (Dadgostar, 2019). Infections with drug-resistant bacteria are estimated to contribute to nearly five million deaths every year (Antimicrobial Resistance Collaborators, 2022) and are predicted to be responsible for 39.1 million deaths between 2025 and 2050, an immense number that could be decreased by approximately one-third if a new Gram-negative targeting antibiotic is developed (Naghavi et al., 2024). Therefore, there is an urgent need to develop new therapeutic approaches to treat infections caused by multidrug-resistant (MDR) Gram-negative bacteria (Theuretzbacher and Piddock, 2019). The increasing prevalence of AMR has stimulated the research on adjuvants that directly address bacterial resistance mechanisms. Such drugs could revive the efficacy of already available antibiotics, for example, membrane permeabilizers, efflux inhibitors, or β-lactamase inhibitors (Wright, 2016; Annunziato, 2019; Laws et al., 2019).

Efflux systems play a major role in AMR of Gram-negative bacteria, several of which are listed on the World Health Organization priority pathogens list for R&D of new antibiotics (Santajit and Indrawattana, 2016; Tacconelli et al., 2018; Sati et al., 2025). In particular, the overexpression of efflux pumps belonging to the resistance-nodulation-cell division (RND) superfamily is a major factor of AMR (Yasufuku et al., 2011), causing sub-inhibitory antibiotic levels, thereby impairing therapeutic efficacy. In addition, this may promote the development of target-based resistance (Cernicchi et al., 2021). Indeed, efflux pump overexpression correlates with the development of other resistance mechanisms to fluoroquinolones due to the coupled lower expression of the DNA mismatch repair gene *mutS* (El Meouche and Dunlop, 2018). Therefore, the development of efflux pump inhibitors (EPIs) is a promising strategy to overcome AMR and to restore the susceptibility of MDR bacteria to existing and future antibiotics by preventing their extrusion (Bolla et al., 2011). Due to the reported roles of efflux systems in biofilm formation and virulence, EPIs could also be employed as antibiofilm and antivirulence agents (Alav et al., 2018; Reza et al., 2019; Piddock, 2006).

The main efflux system in *E. coli* is the tripartite complex AcrAB-TolC, which actively transports antibiotics from the bacterium into the external medium (Li et al., 2015). The complex is composed of the RND-type multidrug efflux pump AcrB, the periplasmic adapter protein AcrA, and the outer membrane (OM) factor TolC (Du et al., 2014). TolC is a homotrimer embedded in the OM with a 12-stranded β-barrel and a periplasmic domain composed of 12 α-helices forming an α-barrel that extends approximately 100 Å into the periplasmic space (Koronakis et al., 2000). In the absence of other efflux system components, TolC remains closed, whereas after formation of the tripartite complex TolC opens by an iris-like movement of the helices at the periplasmic tip (Andersen et al., 2002a; Sjuts et al., 2016). In addition to AcrA and AcrB, TolC can be associated with several RND- and non-RND efflux systems (e.g., MdtEF, MacAB, EmrAB) and its deletion has been shown to result in increased susceptibilities of *E. coli* for several antibiotics (Alav et al., 2021; Sulavik et al., 2001).

Various classes of antimicrobials have been identified as AcrAB-TolC substrates, including chloramphenicol as well as different macrolide and β-lactam antibiotics (Bolla et al., 2011). Therefore, targeting the AcrAB-TolC efflux system represents an attractive strategy for pharmaceutical intervention to reconstitute the effect of antibiotics (Annunziato, 2019). Pyranopyridine derivatives and pyridylpiperazine-based compounds have been reported as AcrB inhibitors, thereby decreasing the minimum inhibitory concentration (MIC) of the antibiotics levofloxacin and piperacillin (Sjuts et al., 2016; Plé et al., 2022). Similarly, several EPIs targeting AcrA have been identified that shift the MIC of different antibiotics in *E. coli* (Haynes et al., 2017; Abdali et al., 2017). The colicin E1-TR fragment has been demonstrated to plug the TolC channel and to shift the MIC of erythromycin, ciprofloxacin, and benzalkonium chloride (Budiardjo et al., 2022). Inorganic cations such as hexaamminecobalt have been shown to block TolC by interacting with conserved aspartates (Asp371, Asp374) within the TolC lumen close to the periplasmic tip (Andersen et al., 2002b; Higgins et al., 2004), although lacking synergistic activity with antibiotics (Andersen et al., 2002a; Gilardi et al., 2017). Recently, we reported the first small molecule (LP-115) targeting the AcrA-TolC interface resulting in the disassembly of the AcrAB-TolC complex *in vitro* (Szal et al., 2025). Small organic molecules interfering with TolC function or its homologs in other Gram-negative bacteria might be further developed into adjuvants that restore the sensitivity of bacteria for antibiotics (Phan et al., 2015).

In this study we report the discovery and optimization of compounds that interfere with antimicrobial efflux. Based on a virtual screen against *E. coli* TolC, several compounds were identified, one of which shifted the MIC of piperacillin and levofloxacin. Using comprehensive medicinal chemistry efforts, microbiological assays, and binding studies, the initial hit was further optimized, resulting in two compounds selectively binding to TolC.

## 2 Materials and methods

### 2.1 Molecular modeling

A subset of approximately 5.9 million compounds was downloaded from the ZINC database (Irwin et al., 2020) based on the following selection criteria: (i) molecular weight >375 Da (based on internal data of TolC-binding compounds), (ii) logP −1 to 5 (−1 lowest possible logP in ZINC tranche browser, five based on Lipinski et al., 2001), (iii) reactivity: standard, (iv) purchasability: agent, (v) pH: reference and mid, (vi) formal charge 0 to +2 (negative charge associated with poor membrane permeability, positive charge limited to +2 to limit membrane binding). Using the molecular modeling suite Molecular Operating Environment (MOE) version 2019.01.02 (Chemical Computing Group Inc., Montreal, Canada), the set was further filtered (logS > -4.5, modified from Jorgensen and Duffy, 2002; compounds containing a positively charged nitrogen atom), resulting in a final library of 653,617 compounds.

*E. coli* TolC X-ray crystal structures (PDB IDs 1TQQ and 1EK9) were downloaded from the Protein Data Bank (Koronakis et al., 2000; Higgins et al., 2004; wwPDB consortium, 2019). All non-protein atoms were removed. Both TolC structures were superposed (Cα atoms) and hydrogen atoms were added using MOE, followed by energy minimization (Amber14:EHT force field with R-Field implicit solvation model) using a three-step procedure (Cα atoms, backbone, all atoms), each step including a tether of 0.5 and an RMS gradient of 5. Compounds were docked into the periplasmic tip area of both structures using GOLD version 1.8 (Cambridge Crystallographic Data Centre, Cambridge, UK). The search space was defined by a sphere of 15 Å radius centered within the small TolC opening at the periplasmic tip. For each compound 10 docking runs were conducted. The early termination option was switched off.

### 2.2 Bacterial strains and reagents

Clinical control strain *E. coli* BW25113 (parental strain) and the single-gene knock-out mutant strains *E. coli* JW5503 (Δ*tolC*) and *E. coli* JD17464 (Δ*lpxC*) were obtained from the *E. coli* collection of the National BioResource Project at the National Institute of Genetics (Japan) (Baba et al., 2006). Uropathogenic *E. coli* UMN026, selected as model for a multi-resistant and clinically relevant strain (Manges et al., 2001). was obtained from the Leibniz Institute DSMZ German Collection of Microorganisms and Cell Culture. Piperacillin sodium salt, phenylalanine-arginine β-naphthylamide dihydrochloride (PAβN), bis-benzimide Hoechst 33,342 trihydrochloride (H33342), levofloxacin and mefloquine hydrochloride were purchased from Sigma.

### 2.3 Hoechst 33342 accumulation assay

The assay was conducted as previously described (Coldham et al., 2010). Compounds were added to the bacterial suspension to achieve a final concentration of 100 µM. PAβN was used as positive control (25 μg/mL). *E. coli* JW5503 cells can naturally and rapidly accumulate H33342 intracellularly, thus showing a maximum fluorescence. The accumulation obtained in *E. coli* BW25113 treated with compounds and PAβN was compared to the accumulation observed in JW5503 and calculated as percentage values (*E. coli* JW5503 was assumed as 100%). A compound was considered a contributor to the increased intracellular H33342 concentration if it reached an equivalent of 80% of that observed in *E. coli* JW5503. H33342 efflux was monitored using a fluorescence microplate reader (VarioskanLUX, Thermo Fisher Scientific) to measure the fluorescence (excitation 355 nm, emission 460 nm) at 37 °C every 5 min for 30 min. The experiment was carried out once with two technical replicates for each compound. Further details are available in the Supplementary Material.

### 2.4 Antibiotic potentiation assay and antimicrobial activity tests

The assays were carried out by the broth microdilution method in 96-well plate format according to the Clinical and Laboratory Standards Institute (CLSI) guidelines (CLSI, 2015). MIC values were determined according to CLSI guidelines and the definition used in our assay corresponds to ≥90% growth inhibition as determined spectrophotometrically. For the potentiation activity, an equal volume of bacterial suspension and compound solution, diluted into Cation-Adjusted Mueller Hinton Broth (CAMHB) containing piperacillin (final concentrations of 1 μg/mL) was used. The intrinsic MIC of each compound against *E. coli* BW25113 was determined using a similar assay, with the exception that the serial dilutions of compounds did not contain antibiotics. PAβN at 50 μg/mL was used as positive control in potentiation assays, while piperacillin alone (4 μg/mL) for antibacterial activity of compounds. Compounds were tested at concentrations ranging from 125 to 0.39 µM. Two independent experiments with three technical replicates each were carried out. MIC determinations for PAβN were also carried out to ascertain that the concentration used was not inhibitory in itself (Supplementary Table S3). Further details are available in the Supplementary Material.

For the most promising compounds (MPC_4_ ≤6.25 μM, and TolC-binding) similar assays as described above, were conducted to test the ability to potentiate the activity of levofloxacin. Similarly, compounds were assessed in clinical multi-resistant uropathogenic *E. coli* UMN026 strain for the potentiation of piperacillin. Mefloquine hydrochloride at 3.2 μg/mL combined with 128 μg/mL of piperacillin and piperacillin alone at 512 μg/mL were used as a positive control on every assay plate for MPC_4_ and MIC determinations. Two independent experiments with three technical replicates each were carried out.

### 2.5 Protein expression and purification

TolC, AcrA, and AcrB expression and purification were conducted according to a previously reported protocol (Szal et al., 2025; Szal et al., 2023). Tobacco, etch virus protease (TEVp) was kindly provided by the Protein Production Core Facility at CSSB. The pET24a based plasmid for the expression of a C-terminally His-tagged full-length TolC was kindly provided by Mathias Winterhalter, Constructor University, Bremen, Germany, and *E. coli* C43 (DE3) Δ*acrAB* was kindly provided by Martin Pos, Goethe University, Frankfurt, Germany.

### 2.6 Protein-ligand binding studies

Microscale thermophoresis (MST) studies with AcrA, AcrB, or TolC and test compounds were carried out in quintuplicates on a Monolith NT.115 LabelFree device (NanoTemper Technologies) at the Sample Preparation and Characterization (SPC) facility at EMBL Hamburg, as previously described (Szal et al., 2023; Szal et al., 2025). The compounds were titrated in a two-fold (**1a**) or three-fold (**2c**, **2f**, **4b**, **4c**, **4d**, **4e**) dilution series in the respective buffer, and the final DMSO concentration was 4% (**2c**, **2f**, **4b**, **4c**, **4d**, **4e**) or 0% (**1a**) throughout all samples of the dilution series. The highest compound concentrations in the assay were limited by solubility constraints or unspecific quenching of the initial fluorescence (for MST data analysis). Compound binding to TEVp was assessed in quintuplicates for compounds that did not bind TEVp (**1a**, **4b**, **4c**, **4d**, **4e**), and in duplicate (**4f**) or once (**4c**) for TEVp-binding compounds.

MST data points that showed irregular capillary shape or differed by more than 20% in the initial fluorescence were excluded (López-Méndez et al., 2021). Initial fluorescence (IF) data points with irregular capillary shape or MST curves were removed. IF values or MST signals at a certain time point (specified at the tables or graphs) were fitted with the ThermoAffinity tool (https://spc.embl-hamburg.de/) (Burastero et al., 2021) using a one-site fitting equation, and plotted against the concentrations of the compound using GraphPad Prism 10.0.2 (GraphPad Software, Inc.). Compounds with curves that could not yield a 95% confidence interval (Paketurytė et al., 2021) for the K_D_ estimation or for which fitting parameters could be estimated were interpreted as not binding.

Samples for nano differential scanning fluorimetry (nDSF) using a Prometheus (NanoTemper Technologies) were prepared in a similar manner to that for the MST studies (identical protein and compound concentration, dilution series and the buffer composition). After incubation, the samples (triplicates) were loaded into Prometheus High Sensitivity Capillaries (NanoTemper Technologies). The samples were heated 1 °C per minute in a temperature range adapted to the melting temperature of TolC (T_M_ = 75 °C–79 °C). For K_D_ determination, the melting curves were analyzed with the FoldAffinity tool (Niebling et al., 2021) using the “Local” equation for parameters (i.e., T_M_ estimation, unfolded fraction). The T_M_ shift was fitted using the One-site model. Isothermal fitting was conducted close to the T_M_ of TolC without compound (78 °C) using the one-site model. Data points that exhibited irregular melting curves were excluded. The data points and curves were plotted against the concentrations of the compound using GraphPad Prism 10.0.2 (GraphPad Software, Inc.).

### 2.7 Chemistry

Reagents and starting materials were obtained from commercial sources and used as received. Starting materials and dry solvents (1,4-dioxane, MeCN, DMF, DMSO) were purchased from Fluorochem, SigmaAldrich, Acros, or Alfa Aeser and used as received. Dry THF, Et_2_O and DCM were obtained from dry solvent still. Flash column chromatography was carried out using silica gel (35–70 µm). Thin layer chromatography (TLC) was performed on silica gel using Merck TLC Silica gel 60 F254 Aluminum sheets and was visualized by UV lamp. NMR spectra were recorded on 300 or 400 MHz Bruker spectrometers with chemical shift values (δ) in parts per million using the residual solvent signal as an internal standard. HRMS analyses were performed on a hybrid quadrupole time -of -flight mass spectrometer equipped with an electrospray ion source. The purity of all the tested compounds was estimated by HPLC (Empower three or SHIMADZU LabSolutions, UV detection at λ = 210 nm) analysis on an Adamas C18 column size: 4.6 × 150 mm, mobile phase: MeCN - 0.1% H_3_PO_4_, flow rate: 1.0 mL/min, detector: UV 210 nm and UV 254 nm column temperature: 40 °C, sample concentration: 0.5 mg/mL. All compounds are >95% pure by HPLC analysis unless otherwise stated. Further information on the synthesis of the compounds is included in the Supplementary Material.

## 3 Results

### 3.1 Virtual screening

Recently, the periplasmic tip of TolC has been shown to be a potential target for small molecules (Szal et al., 2025). To identify additional compounds addressing TolC, a structure-based virtual screening campaign was conducted. For this purpose, a screening library was designed, containing commercially available small organic compounds with a molecular weight above 375 Da and possessing a positively charged nitrogen atom, which is predicted to facilitate uptake into Gram-negative bacteria (Richter et al., 2017) (Figure 1). Subsequently, the library containing 654,000 compounds was docked into the propeller-shaped cavity at the periplasmic tip of TolC. Based on the docking scores and the normalized docking scores (score divided by the number of heavy atoms), the binding modes of the 250 top-ranked compounds were visually inspected. In the analysis, special attention was paid to the geometric fitting of the docking pose into the putative binding pocket and the presence of polar interactions with TolC, in particular with the conserved aspartates Asp371 and Asp374. Finally, eleven compounds with promising binding modes were identified as potential TolC binders (Supplementary Table S1).

**FIGURE 1:**
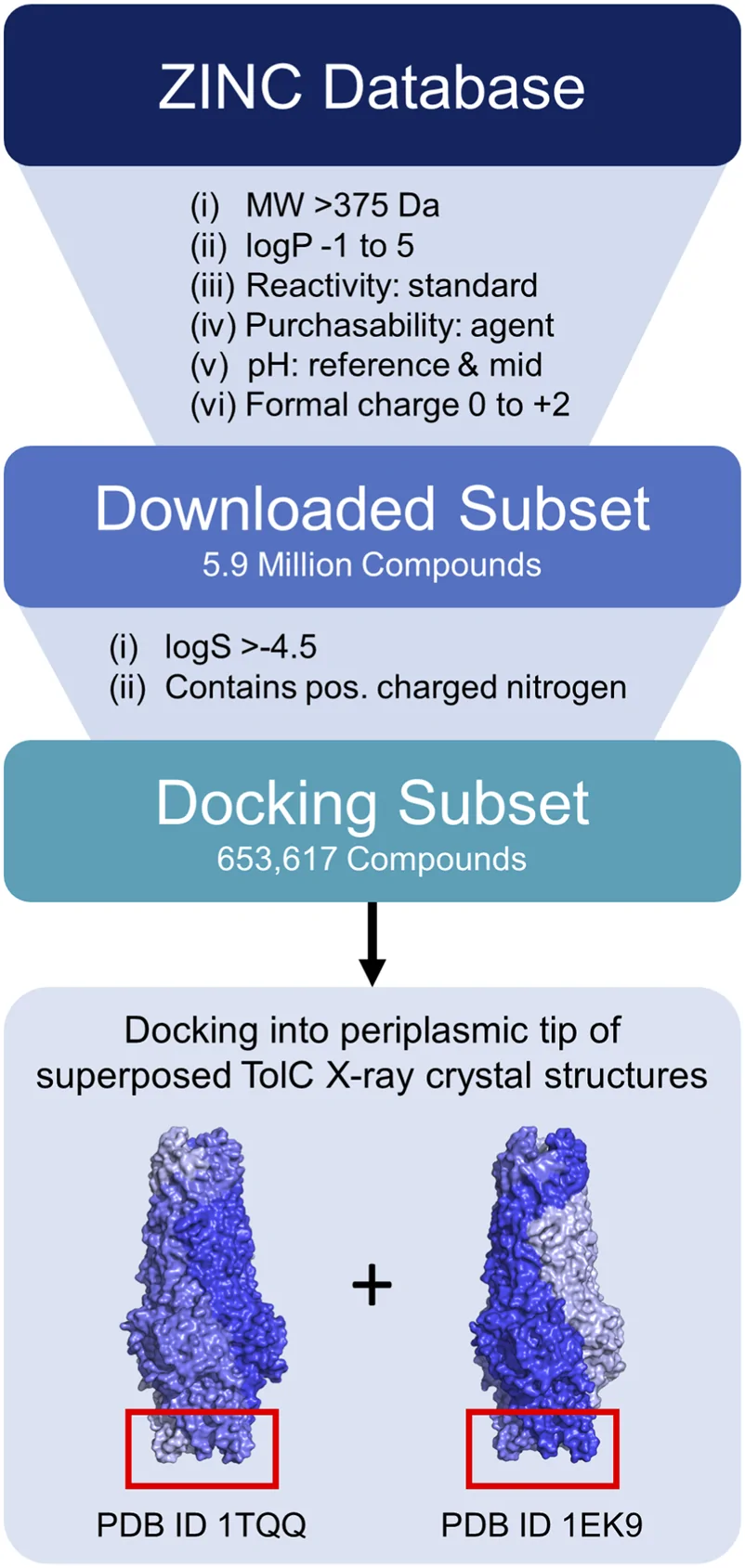
Computational workflow illustrating the selection criteria for ZINC database subset download, additional descriptors calculated within MOE to focus on compounds with better solubility and likelihood of bacterial accumulation, and docking of the resulting collection into the periplasmic tip of two TolC X-ray crystal structures.

### 3.2 Hit validation and characterization

All virtual hits were validated experimentally using a two-step procedure. Initially, we assessed each compound for interference with efflux pump function by quantifying the accumulation of the fluorescent DNA dye H33342, a known AcrAB-TolC substrate. Subsequently, efflux-inhibiting compounds were tested for synergistic activity in combination with the antibiotic piperacillin.

Only one compound (**1a**) resulted in an intracellular accumulation of H33342 in *E. coli* BW25113 ≥ 80% relative to the *tolC*-deficient *E. coli* strain JW5503 at 100 μM (Supplementary Table S1). All other compounds showed an accumulation ranging between 14.5% and 78.3%. Similarly, **1a** inhibited bacterial growth in combination with sub-inhibitory concentration of piperacillin (1 μg/mL, 0.25 MIC). Subsequently, we determined the minimum potentiating concentration (MPC_4_) that resulted in a 4-fold improvement in the MIC of piperacillin (4 μg/mL), which was 75 µM. To exclude any interference due to an intrinsic antimicrobial activity of **1a**, the assay was carried out in the absence of piperacillin. The MIC for **1a** was not reached at the highest tested concentration (125 µM) (Table 1; Supplementary Table S5), indicating synergism between **1a** at 75 µM and piperacillin.

**TABLE 1:** Modification of substructure B at the middle benzene ring.

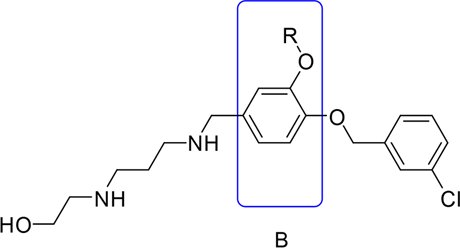					
Compound	Structure, R	MPC_4_ ^a^ PIP (µM)	MIC^b^ (µM)	MIC/MPC_4_	H33342 accumulation (%, average ± SD)^c^
**1a**	Me-	75	>125	>1.67	142.1 ± 1.4
**2a**	Et-	50	>125	>2.5	113.4 ± 0.3
**2b**	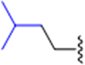	12.5	25	2	137.4 ± 1.3
**2c**	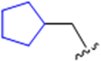	6.25	12.5	2	146.1 ± 4.4
**2f**	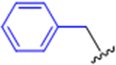	6.25	25	4	152.0 ± 0.9
PAβN (25 μg/mL)	​	na	na	na	113.9 ± 1.6

^a^ MPC_4_ PIP: Minimum Potentiating Concentration was defined as the concentration of compound that decreased MIC, of piperacillin by 4-fold (i.e., from 4 μg/mL to 1 μg/mL) in *Escherichia coli* BW25113. If MPC_4_ was not achieved, the value is represented by the highest concentration of compound tested.

^b^ MIC: Minimum Inhibitory Concentration was defined as the concentration of compound that inhibited *Escherichia coli* BW25113 growth by ≥ 90%. If MIC, was not achieved, the value is represented by the highest concentration of compound tested.

^c^ Compounds were tested at 100 µM. The accumulation of H33342 is expressed as a percentage and standard deviation in relation to *tolC*-deficient strain *Escherichia coli* JW5503. Values obtained in relative fluorescence units were converted into percentage and values for *Escherichia coli* JW5503 were attributed as 100%. The fluorescence intensity was recorded at excitation and emission wavelengths of 355 and 460 nm, respectively, after 30 min incubation period at 37 °C. PAβN (25 μg/mL) was used as a positive control (Lomovskaya et al., 2001). Experiments were performed once in duplicate.na: not applicable.

Next, we assessed **1a** binding to individual efflux pump subunits using microscale thermophoresis and fluorescence quenching. MST signals obtained for TolC and AcrA were used for analysis, since no quenching of the fluorescence occurred. We observed quenching for AcrB in the concentration range used for the binding studies. By analyzing the quenching of the initial fluorescence for AcrB, it was ensured that the same compound concentrations were analyzed. Compound **1a** demonstrated weak affinity for TolC with a K_D_ of 347 µM (95% confidence interval [127; 1200 µM]) and AcrB (K_D_ = 182 µM [49.5; 690 µM]) (Figures 2a,b). In contrast, the compound did not bind to AcrA (Figure 2c). To further rule out any non-specific protein binding or measurement artefacts, such as an inner filter effect, binding of **1a** to the structurally unrelated TEVp was examined. **1a** did not interact with TEVp (Figure 2c).

**FIGURE 2:**
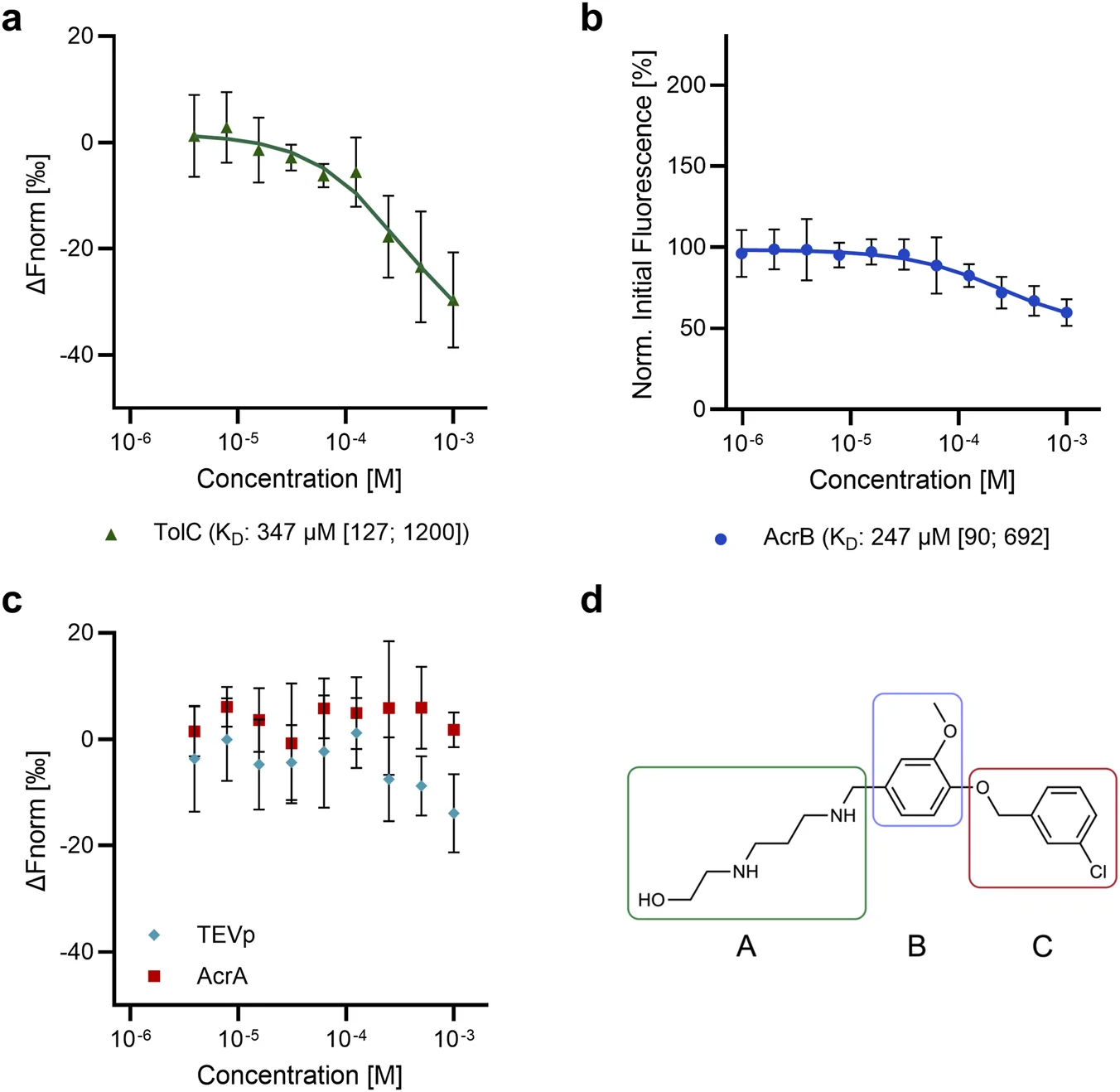
Dose-response curves for **1a** binding to efflux pump proteins TolC **(a)**, AcrB **(b)** and AcrA **(c)**, as well as tobacco, etch virus protease (TEVp, **(c)**, obtained by analyzing MST signals (TolC, AcrA) or initial fluorescence values (AcrB). Signals recorded at 9–10 s were used for data analysis of TolC, AcrA and TEVp. For AcrB fitting, the signal for each concentration is the mean of the initial fluorescence intensity at 1 to 0 s before heating by laser normalized on the signal of AcrB with DMSO alone. **a**, **b**, **c** Each data point is presented as mean ± SD (n = 5). K_D_ values are presented together with their respective 95% confidence interval (Paketurytė et al., 2021). Data points were derived from Supplementary Figures S1–S4. **(d)** Chemical structure of the initial hit **1a** and the investigated substructures A, B, and C.

### 3.3 Structure-activity relationship (SAR) studies

To identify the essential parts of **1a** for synergistic activity and to improve efficacy and selectivity, an optimization campaign was initiated, which comprised simple modifications through synthesizing analogues with subtle changes to the flexible side chain (A), the middle core (B), and the benzylic substituent (C) (Figure 2d). All synthesized analogues were investigated experimentally. Compounds displaying a MPC_4_ of ≤125 μM in combination with piperacillin were subjected to MIC determination. All compounds demonstrated H33342 accumulation in *E. coli* BW25113 of at least 80% relative to the *tolC*-deficient *E. coli* strain JW5503 at a concentration of 100 μM, irrespective of the piperacillin-potentiating activity (Table 1). Data for compounds without improved synergistic activity compared to **1a** are presented in Supplementary Tables S2-4.

In a first step, the flexible side chain consisting of a 2-({3-[(2-hydroxyethyl)amino]propyl}amino)methyl substituent (substructure A) was investigated with respect to its relevance for the synergistic effect of **1a** with piperacillin. Two analogues containing a shortened side chain were synthesized. This substructure was found to be critical for the biological activity, as a reduction in the length by a methyl (**1b**) or aminoethyl group (**1c**) abolished the synergistic effect with piperacillin (Supplementary Table S2).

Next, we focused on altering the substituents at the middle benzene ring (substructure B). Replacing the methyl group of the methoxy substituent with larger nonpolar aliphatic groups (**2a**–**2e,** Table 1; Supplementary Table S3) substantially decreased the MPC_4_, thereby improving the synergy with piperacillin. However, these compounds also demonstrated low MICs (6.25–25 μM, Table 1; Supplementary Table S3), resulting in a poor ratio between intrinsic antibacterial activity and synergy for compounds **2d** and **2e**. Larger aliphatic substituents at substructure B such as tert-butyl (**2d**) or cyclohexane (**2e**) led to a loss of synergistic activity. However, the benzylic substituent (**2f**) demonstrated a low MPC_4_ (6.25 µM) and a 4-fold MIC/MPC_4_ ratio.

Subsequently, we investigated the SAR of substituents at the aromatic ring in substructure C. Removal of chlorine (**3a**) resulted in the loss of synergistic properties (Supplementary Table S4). Changing the chlorine substituent of **1a** to ortho (**3b**) or para position (**3c**) at the benzene ring slightly impaired the synergistic activity. Likewise, the exchange of chlorine for fluorine (**3d**) or bromine (**3f**) in the meta-position resulted in loss or no change in synergism with piperacillin (Supplementary Table S3). However, increasing the size of the halogen in para position was beneficial for the synergistic effect, as compounds containing a bromine (**3e**) or iodine substituent (**3g**) were slightly more potent than **1a** (Table 2). None of the two monohalobenzyl analogues exhibited an intrinsic antibacterial activity (MIC >125 µM), resulting in a >1.67 to >2.5-fold ratio between MPC_4_ and MIC.

**TABLE 2:** Substructure C modifications connected via an oxymethylene group to substructure B.

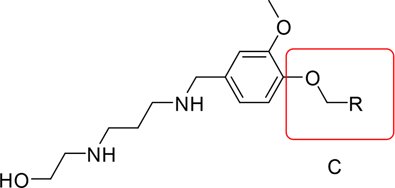					
Compound	Structure, R	MPC_4_ ^a^ PIP (µM)	MIC^b^ (µM)	MIC/MPC_4_	H33342 accumulation (%, average ± SD)^c^
**1a**	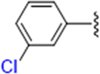	75	>125	>1.67	142.1 ± 1.4
**3e**	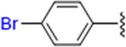	50	>125	>2.5	99.9 ± 2.6
**3g**	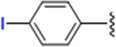	50	>125	>2.5	103.8 ± 3.0
**3k**	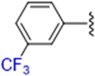	50	>125	>2.5	92.8 ± 1.4
**3L**	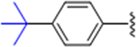	25	125	5	121.4 ± 0.1
**3m**	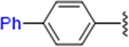	25	75	3	148.1 ± 1.5
**3o**	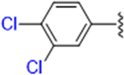	25	>125	>5	113.2 ± 1.0
**3p**	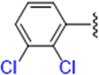	25	>125	>5	113.0 ± 1.6
**3q**	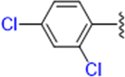	25	>125	>5	118.3 ± 0.6
**3r**	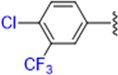	25	100	4	127.2 ± 1.7
**3s**	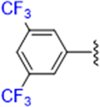	12.5	75	6	127.3 ± 0.6
**3u**	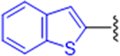	50	>125	>2.5	107.5 ± 1.7
PAβN (25 μg/mL)	​	na	na	na	117.5 ± 0.5

^a^ MPC_4_ PIP: Minimum Potentiating Concentration was defined as the concentration of compound that decreased MIC, of piperacillin by 4-fold (i.e., from 4 μg/mL to 1 μg/mL) in *Escherichia coli* BW25113. If MPC_4_ was not achieved, the value is represented by the highest concentration of compound tested.

^b^ MIC: Minimum Inhibitory Concentration was defined as the concentration of compound that inhibited *Escherichia coli* BW25113 growth by ≥ 90%. If MIC, was not achieved, the value is represented by the highest concentration of compound 5tested.

^c^ Compounds were tested at 100 µM. The accumulation of H33342 is expressed as a percentage and standard deviation in relation to *tolC*-deficient strain *Escherichia coli* JW5503. Values obtained in relative fluorescence units were converted into percentage and values for *Escherichia coli* JW5503 were attributed as 100%. The fluorescence intensity was recorded at excitation and emission wavelengths of 355 and 460 nm, respectively, after 30 min incubation period at 37 °C. PAβN (25 μg/mL) was used as a positive control (Lomovskaya et al., 2001). Experiments were performed once in duplicate. na: not applicable.

Next, we investigated the impact of hydrophobic substituents at the benzylic group (substructure C). Replacement of the nonpolar chlorine (**1a**, lipophilicity contribution ΔlogD_radius=0_: 0.6) with polar, hydrophilic groups (**3h**: cyano, ΔlogD_radius=0_: −0.28; **3i**: MeO ΔlogD_radius=0_: −0.05; Supplementary Table S4) resulted in loss of activity. A methyl substituent in the same position as the chlorine (**3j**, ΔlogD_radius=0_: 0.3; Supplementary Table S4) impaired the MPC_4_. Conversely, lipophilic substituents improved the MPC_4_. The trifluoromethyl group (**3k**, ΔlogD_radius=0_: 0.9) increased the ratio between MPC_4_ and MIC by > 2.5-fold, and the tert-butyl-containing analogue **3L** (ΔlogD_radius=0_: 1.3) resulted in a 5-fold ratio (Table 2). However, we observed an intrinsic antibacterial activity for a sterically demanding and strongly lipophilic biphenyl analogue **3m** (ΔlogD_radius=0_: 1.4).

Furthermore, a series of Cl- and CF_3_- substituted analogues **3n**–**3s** were synthesized to systematically probe different double substitutions at the benzene ring of substructure C. The 2,6-substitution with chlorine (**3n**, Supplementary Table S3) resulted in a slightly higher synergistic effect than the mono-chlorine substitution in ortho position (**3b**, Supplementary Table S3) but showed no improvement compared to **1a**. The 3,4-, 2,3-, and 2,4-substitution patterns of chlorine (**3o**, **3p**, and **3q**; Table 2) improved the synergistic activity by a factor of three compared to **1a**, without increasing the intrinsic antibacterial activity. The exchange of chlorine in the two-position in **3o** with a trifluoromethyl-substituent (**3r**, Table 2) resulted in a higher intrinsic antibacterial activity. The 3,5-substitution with trifluoromethyl was most beneficial for synergism with piperacillin in *E. coli* BW25113 (**3s**) with a comparably low MPC_4_ (12.5 µM, Table 2) and an MIC/MPC_4_ ratio of 6.

Most of the heterocyclic analogues **3t**–**3w** did not show an improved MPC_4_ compared to compound **1a** (Table 2; Supplementary Table S4). The bioisosteric replacement of the aryl group with a thiophene ring in **3t** resulted in an activity comparable to that of **1a** (Supplementary Table S4), while the benzothiophene-containing compound was slightly more potent (**3u**, Table 2). However, the inclusion of an isoquinoline (**3v**) or 2-methyl pyridine (**3w**) abolished any synergistic activity.

In a final step, we combined a side chain length as present in **1a** at substructure A, a 3,5-trifluoromethylsubstituted benzylic ring as in **3s** at substructure C, and a benzyl ring at substructure B, containing additional ring substitutions. To this end, several 3,5-disubstituted benzyl analogues **4b**–**4e** were synthesized (Table 3). A compound missing the benzylic substituent and ether (**4a**, Table 3) was synthesized for comparison with the other analogues. Indeed, all compounds exhibited a synergistic effect with piperacillin at lower concentrations than **1a** with varying MICs. Compound **4a** demonstrated the highest MPC_4_ (12.5 µM, Table 3) of this series. The addition of a large steric substituent in para-position slightly improved the MPC_4_ (6.25 µM) and showed antibacterial activity at 12.5 µM (**4b**). The inclusion of a trifluoro-benzylic ring in compound **4c**, revealed the same synergistic activity with piperacillin (MPC_4_ = 6.25 µM) but a reduced intrinsic antibacterial effect, resulting in an 8-fold MIC/MPC_4_ ratio. Substitution with a dichloro-benzylic ring in **4d** and **4e** resulted in the lowest MPC_4_ values of all tested compounds in this study (3.13 µM, Table 3) but also showed a strong intrinsic antibacterial activity.

**TABLE 3:** Modification of substructure B with **3s**-pattern at substructure C.

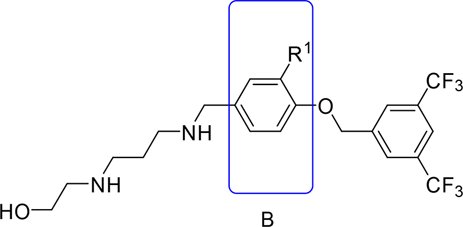					
Compound	Structure, R	MPC_4_ ^a^ PIP (µM)	MIC^b^ (µM)	MIC/MPC_4_	H33342 accumulation (%, average ± SD)^c^
**4a**	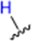	12.5	50	4	117.6 ± 1.3
**4b**	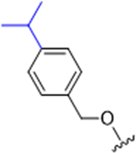	6.25	12.5	2	211.7 ± 8.6
**4c**	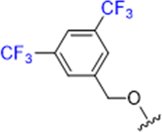	6.25	50	8	223.4 ± 11.0
**4d**	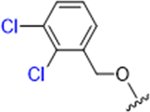	3.13	6.25	2	142.8 ± 7.2
**4e**	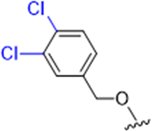	3.13	6.25	2	207.4 ± 16.4

^a^ MPC_4_ PIP: Minimum Potentiating Concentration was defined as the concentration of compound that decreased MIC, of piperacillin by 4-fold (i.e., from 4 μg/mL to 1 μg/mL) in *Escherichia coli* BW25113.

^b^ MIC: Minimum Inhibitory Concentration was defined as the concentration of compound that inhibited *Escherichia coli* BW25113 growth by ≥ 90%.

^c^ Compounds were tested at 100 µM. The accumulation of H33342 is expressed as a percentage and standard deviation in relation to *tolC*-deficient strain *Escherichia coli* JW5503. Values obtained in relative fluorescence units were converted into percentage and values for *Escherichia coli* JW5503 were attributed as 100%. The fluorescence intensity was recorded at excitation and emission wavelengths of 355 and 460 nm, respectively, after 30 min incubation period at 37 °C. PAβN (25 μg/mL) was used as a positive control (Lomovskaya et al., 2001). Experiments were performed once in duplicate.

### 3.4 Prioritization of analogues

Altogether six compounds demonstrated a MPC_4_ ≤6.25 μM (**2c**, **2f**, **4b**, **4c**, **4d**, and **4e**, Tables 1, 3). To prioritize compounds for further studies, the interaction of each compound with TEVp was examined. Only **2c** (K_D_ = 298 µM CI [36.6, 1910]) and **2f** (2494 µM CI [690, 7060]) showed weak TEVp binding and were therefore excluded from further investigations (Supplementary Figure S6).

To ensure that the observed potentiation of piperacillin in *E. coli* BW25113 was specific to AcrAB-TolC inhibition, compounds with off-target activity were excluded by conducting antibiotic potentiation assays with efflux-deficient *E. coli* JW5503 (Δ*tolC*) in presence of piperacillin or levofloxacin (Table 4). The MIC/MPC_4_ ratio of EPIs in the mutant strain is expected to be lower than in wildtype bacteria, ideally approaching 1. Except for **4b**, all selected compounds showed a lower MIC/MPC_4_ ratio in the mutant strain. **4d** and **4e** exhibited an MIC/MPC_4_ ratio of one together with piperacillin and levofloxacin and **4c** displayed an MIC/MPC_4_ ratio of one in combination with levofloxacin. Interestingly, compound **4c** demonstrated increased antimicrobial activity in efflux-deficient *E. coli*, indicating that it is a substrate of the AcrAB-TolC efflux pump complex. Furthermore, the activity of levofloxacin was not potentiated by PA*β*N, suggesting that it is not affected by the membrane-permeabilizing off-target effect.

**TABLE 4:** MPC_4_ and MIC of compounds tested in combination with piperacillin or levofloxacin in *Escherichia coli* JW5503 (Δ*tolC*).

Compound	MPC_4_ PIP^a^ (µM)	MIC^b^ (µM)	MIC/MPC_4_ PIP	MPC_4_ LEV^c^ (µM)	MIC/MPC_4_ LEV
**4b**	6.25	12.5	2	6.25	2
**4c**	6.25	12.5	2	12.5	1
**4d**	6.25	6.25	1	6.25	1
**4e**	6.25	6.25	1	6.25	1
**PA*β*N**	12.5 μg/mL	50 μg/mL	4	50 μg/mL	1

^a^ MPC_4_ PIP: Minimum Potentiating Concentration was defined as the concentration of compound that decreased MIC, of piperacillin by 4-fold (i.e., from 0.25 μg/mL to 0.0625 μg/mL) in *Escherichia coli* JW5503.

^b^ MIC: Minimum Inhibitory Concentration was defined as the concentration of compound that inhibited JW5503 growth by ≥ 90%.

^c^ MPC_4_ LEV: Minimum Potentiating Concentration was defined as the concentration of compound that decreased MIC, of levofloxacin by 4-fold (i.e., from 0.01 μg/mL to 0.0025 μg/mL) in *Escherichia coli* JW5503.

Compounds **4c**, **4d**, and **4e** were assessed for potentiation of levofloxacin in wildtype *E. coli* and *E. coli* JD17464 (Δ*lpxC*) possessing an impaired OM (Table 5) to investigate whether the synergistic effect of the compounds is due to permeabilizing the OM which may lead to increased antibiotic permeation into the cells (Mood et al., 2021). EPIs are expected to retain their activity in OM-compromised bacteria. We observed no change in MPC_4_ for **4c** and **4e** with levofloxacin in BW25113 and JD17464. Only **4d** lost its potentiating activity in *E. coli* JD17464, attributing its potentiating effect with antibiotics to off-target mechanisms, and therefore was excluded from further investigations.

**TABLE 5:** MPC_4_ and MIC of compounds **4c** to **4e** in combination with piperacillin or levofloxacin in *Escherichia coli* BW25113 (wildtype) and JD17464 (Δ*lpxC*).

​	*E. coli* BW25113			*E. coli* JD17464 (lpxC)		
Compound	MPC_4_ LEV^a^ (µM)	MIC^b^ (µM)	MIC/MPC_4_ LEV	MPC_4_ LEV^c^ (µM)	MIC (µM)	MIC/MPC_4_ LEV
**4c**	6.25	50	8	6.25	50	8
**4d**	3.13	6.25	2	6.25	6.25	1
**4e**	3.13	6.25	2	3.13	6.25	2

^a^ MPC_4_ LEV: Minimum Potentiating Concentration was defined as the concentration of compound that decreased MIC, of piperacillin by 4-fold (i.e., from 4 μg/mL to 1 μg/mL) in *Escherichia coli* BW25113.

^b^ MIC: Minimum Inhibitory Concentration was defined as the concentration of compound that inhibited *Escherichia coli* BW25113 or JD17464 growth by ≥ 90%.

^c^ MPC_4_ LEV: Minimum Potentiating Concentration was defined as the concentration of compound that decreased MIC, of levofloxacin by 4-fold (i.e., from 0.0625 μg/mL to 0.0156 μg/mL) in *Escherichia coli* JD17464.

### 3.5 Characterization of selected analogues

Uropathogenic *E. coli* is a leading cause of urinary tract infections and frequently exhibits elevated expression of the AcrAB-TolC efflux system, a major determinant of intrinsic and acquired MDR (Sulavik et al., 2001). In this context, the UPEC reference strain UMN026 was selected in this study as a clinically relevant MDR isolate model (Lescat et al., 2009) To assess the antimicrobial potentiating activity of **4c** and **4e** in clinically relevant settings, the compounds were tested in combination with piperacillin against *E. coli* UMN026. **4e** retained its MIC/MPC_4_ ratio, whereas this ratio was slightly lower for **4c** compared to BW25113 (Table 6). Notably, the intrinsic antibacterial activity of **4c** and **4e** increased against the MDR strain compared to *E. coli* BW25113.

**TABLE 6:** MPC_4_ and MIC of **4c** and **4e** in combination with piperacillin in uropathogenic *Escherichia coli* UMN026.

Compound	MPC_4_ ^a^ PIP (µM)	MIC^b^ (µM)	MIC/MPC_4_
**4c**	1.56	6.25	4
**4e**	1.56	3.13	2

^a^ MPC_4_: Minimum Potentiating Concentration was defined as the concentration of compound that decreased MIC, of piperacillin by 4-fold (i.e., from 512 μg/mL to 128 μg/mL) in uropathogenic *Escherichia coli* UMN026.

^b^ MIC: Minimum Inhibitory Concentration was defined as the concentration of compound that inhibited *Escherichia coli* UMN026 growth by ≥ 90%.

Finally, binding studies with individual efflux pump subunits were conducted to identify the bacterial target protein of compounds **4c** and **4e**. Both compounds showed binding to TolC in a similar concentration range to **1a**, as determined by fluorescence quenching, (Figure 3; Table 7). Since all proteins’ initial fluorescence was quenched by the compounds, the quenching was used for analysis, so higher concentrations could be analyzed. In contrast to **1a**, both compounds demonstrated a very weak affinity for AcrA. Notably, the binding affinity of **4c** and **4e** for TolC was 20-fold higher than that for AcrB, whereas **1a** exhibited similar binding affinities for TolC and AcrB.

**FIGURE 3:**
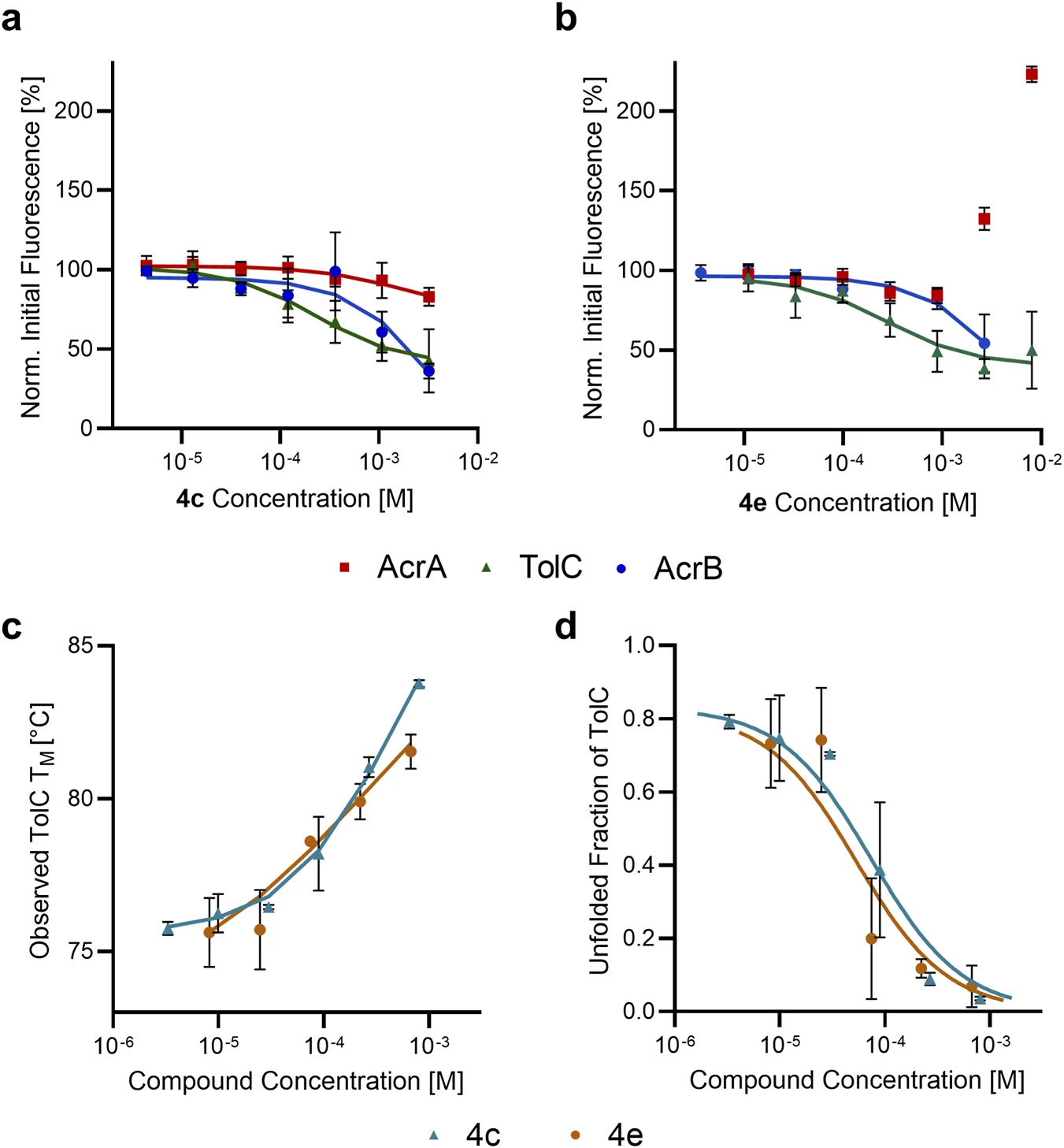
Binding curves obtained by label-free MST **(a,b)** and nano differential scanning fluorimetry **(c,d)**. Interaction of 4c **(a)** and 4e **(b)** with AcrA (red squares), TolC (green triangles), and AcrB (blue dots) based on fluorescence quenching signals, normalized to the fluorescence intensity of the proteins with DMSO only. **(c)** Dose-response curves for **4c** (turquoise triangles) and **4e** (orange dots) with TolC using nDSF and analyzed by fitting the observed melting temperatures of TolC with and without compound treatment at different concentrations. **(d)** Dose-response curves for **4c** and **4e** analyzed by fitting the unfolded fractions of TolC at 78 °C (close to the non-treated melting temperature) with and without compound treatment at different concentrations. Data points were derived from Supplementary Figures S12–S19. Each data point is presented as mean ± SD (n = 5 for MST, n = 3 for nDSF). The corresponding K_D_ values are shown in Table 7.

**TABLE 7:** Summary of binding affinities obtained by MST (quenching and MST signals at 14–15 s) and nano differential scanning fluorimetry (T_M_ shift and unfolded fractions at 78 °C).

Compound^a^	TolC (quenching)		TolC (T_M_ shift)		TolC (unfolded fractions at 78 °C)		AcrA (quenching)		AcrB (quenching)	
	K_D_ [µM]	95% CI	K_D_ [µM]	95% CI	K_D_ [µM]	95% CI	K_D_ [µM]	95% CI	K_D_ [µM]	95% CI
**1a**	347^b^	127, 1200	no shift		no shift		n.b		247	90, 692
**4c**	224	90.4, 563	69	39, 120	12	4.2, 27	1587	201, 6580	4263	1060, 7360
**4e**	274	99.9, 699	12	2.7, 43	9.6	2.1, 27	>8000	​	6526	967, 9930

^a^ Compounds were used as diacetate salts.

^b^ MST, signals at 9–10 s were used for analysis of the **1a**-TolC interaction.

n.b.: not binding; K_D_: equilibrium dissociation constant; CI: confidence interval.

As an orthogonal assay nano differential scanning fluorimetry was used for TolC and analyzed either by fitting the T_M_ shift (Schellman, 1975) or the unfolded fractions close to the melting temperature of TolC (Niebling et al., 2021). The observed T_M_ shift of TolC and in the unfolded fractions upon compound treatment confirmed binding of **4c** and **4e** to TolC in the micromolar range (Figures 3C,D; Table 7), whereas no shift was observed for TolC in presence of **1a** (Table 7; Supplementary Figure S5).

## 4 Discussion

The ever-increasing demand for novel antibiotics requires novel approaches in drug discovery (Miethke et al., 2021). EPIs are considered as a solution to tackle the AMR burden as they may restore the efficacy of antibiotics. However, to date, no such compound has reached clinical trials. Most EPI drug discovery programmes have focused on the efflux pump components AcrA and AcrB as primary targets and have predominantly been conducted using *E. coli* K12-derived laboratory strains or engineered efflux backgrounds (Lomovskaya et al., 2001; Bohnert et al., 2005; Opperman et al., 2014). While a limited number of studies have evaluated EPIs in MDR clinically relevant isolates, including Enterobacteriaceae, *Acinetobacter baumannii*, and *Klebsiella pneumoniae*, these investigations remain relatively scarce (Schumacher et al., 2006; Coban et al., 2011; Vera-Leiva et al., 2018). The fact that TolC deletion increases the susceptibility of *E. coli* to several antibiotics and a recent study reporting the discovery of peptides sterically blocking TolC and shifting the MIC of various antibiotics suggests that TolC is indeed a potential drug target (Sulavik et al., 2001; Budiardjo et al., 2022). Recently, we described the first small molecule (LP-115) interfering with efflux pump assembly by binding to the AcrA-TolC interface (Szal et al., 2025). The identification of LP-115 reassured TolC as a potential target for developing EPIs. TolC offers a significant advantage over AcrA and AcrB, as compounds targeting TolC do not necessarily need to cross the OM, depending on the binding site. The OM largely restricts permeation of large and hydrophobic compounds; therefore, the chemical space of drugs targeting bacterial proteins is limited and has led to the development of rules to prioritize compounds likely to penetrate the OM (Richter et al., 2017; Acosta-Gutiérrez et al., 2018). Targeting TolC might have the advantage of inhibiting not only a crucial protein for efflux, but also a factor for other physiological functions. TolC is associated with OM homeostasis (Zhu et al., 2024), virulence factor transport (Zgurskaya et al., 2011), and iron metabolism (Alav et al., 2021).

The pyranopyridine inhibitor MBX2319 is a potent EPI in laboratory *E. coli* strains, where it shifted the MIC of fluoroquinolones and β-lactams between 4- and 8-fold, with no activity in Δ*acrB* or Δ*tolC* mutants (Opperman et al., 2014). Complementary computational and functional analyses showed that MBX2319 binds within the distal binding pocket of AcrB (Vargiu et al., 2014). Related AcrB-directed inhibitors, including D13-9001 (Nakashima et al., 2013), WK2 (Wang et al., 2021), and BDM88855 (Plé et al., 2022) displayed pronounced antibiotic-dependent activity profiles. WK2, evaluated in *E. coli* K12 strains, potentiated chloramphenicol, erythromycin, and levofloxacin with ∼2–4-fold MIC reductions, but showed minimal activity against rifampicin. In contrast, BDM88855, studied in K12-derived systems, resulted in ∼4–8-fold MIC reductions for chloramphenicol, novobiocin, fusidic acid, and linezolid, while exhibiting little or no effect in combination with aminoglycosides and several β-lactams such as ceftazidime. Collectively, these inhibitors act by stabilizing non-productive AcrB conformational states or blocking substrate access within the distal pocket, but their efficacy remains strongly dependent on the antibiotic class and is largely derived from laboratory K12-based efflux models.

In this study, we report the identification of a compound (**1a**) that interferes with TolC and AcrB and potentiates the activity of piperacillin in *E. coli*. A comprehensive SAR analysis of **1a** resulted in analogues with higher potentiating activity. The subsequent exclusion of non-specific compounds identified analogues **4c** and **4e** with an additional aromatic ring at substructure B as promising candidates for further development. Both compounds feature a lower MPC_4_ and a slightly higher binding affinity to TolC compared to **1a**, but show a much lower binding affinity to AcrB (factor of 20), indicating increased selectivity for TolC. Binding to TolC was confirmed to be in the micromolar range, whereas the affinity to AcrB is in the millimolar range. Compared to previously identified EPIs, the determined binding affinities of **4c** and **4e** for TolC are about 2-fold weaker compared to the AcrA-binding NSC60339 and about one order of magnitude weaker than AcrB-targeting inhibitors (Haynes et al., 2017; Grimsey et al., 2020). Compared with the recently reported LP-115, these compounds showed higher binding affinity and selectivity for TolC and displayed an antimicrobial-potentiating effect in wild-type *E. coli* that is reduced or absent in the *tolC*-deficient strain. LP-115 is mechanistically intriguing because it was shown to act through a novel mode of action, namely, disruption of the AcrA-TolC interface. In contrast, the mechanism of action of compounds **4c** and **4e** has not yet been identified. Nevertheless, **4c** and **4e** appear to be more promising starting points for hit optimization, owing to their antimicrobial-potentiating effect and TolC selectivity, both of which are lacking for LP-115.

The observed differences in TolC binding affinity for compounds **4c** and **4e**, obtained by MST and nDSF, can be explained by the underlying measurement principles. MST data are acquired at room temperature, whereas nDSF data are obtained in the range of the T_M_, which is about 77 °C for TolC. The K_D_ is influenced by several factors, for example, solvent viscosity and temperature (Niebling et al., 2021; Papaneophytou et al., 2014). While moderate temperature changes have only a marginal effect on the K_D_, larger temperature differences are expected to yield more divergent results. Considering this, the absence of a T_M_-shift of compound **1a** does not necessarily mean that binding does not occur, but that the K_D_ may be shifted to another concentration range.

A direct comparison of biophysical (K_D_) and microbiological data (MPC_4_) is challenging as both types of results can be influenced by many factors (Holdgate, et al., 2019). In particular, the environment of the target protein differs substantially between assays, especially when studying an isolated membrane protein like TolC. The observed increased cellular potency may be influenced by off-target effects, the mechanism of action, or compound partitioning (Schwaid and Cornella-Taracido, 2018; Mateus, et al., 2017). The possibility of off-target effects was carefully addressed and several common non-specific effects were investigated. Importantly, compounds **4c** and **4e** showed reduced or no activity in a *tolC*-knockout strain, which strongly indicates that the mechanism of action is at least partially TolC-dependent. Testing the compounds in membrane-compromised *E. coli* JD17464 (Δ*lpxC*) further excluded the compounds to act through a common off-target effect. Furthermore, the compounds inhibited efflux and/or potentiated three AcrAB-TolC substrates in two different assays. Together, these experiments support the conclusion that **4c** and **4e** mainly act through a TolC-dependent mechanism. Nevertheless, additional off-target effects may contribute in part, particularly for **4c**, since the erythromycin-potentiating activity is not completely lost in the *tolC*-knockout strain. Another reason for the increased intracellular potency compared to the biophysical affinity may be partitioning of the compounds into lipophilic regions. In cellular assays, partitioning into the lipid-bilayer may potentiate activity through a non-specific (Rusinova et al., 2021) or specific effect (Aguayo-Ortiz et al., 2021), depending on the mechanism of action and whether the binding site is situated at a transmembrane region or at another membrane-distant site of the protein. Given the number of factors influencing the intracellular potency and the substantial differences in target protein environments between the assays, the affinity data mainly act as confirmation of TolC interaction and selectivity (Holdgate et al., 2019) and as a useful indicator for future compound optimization, but these values should not be directly equated with cellular potency.

Compound **4c** demonstrated a MIC/MPC_4_ ratio of eight for both piperacillin and levofloxacin in *E. coli* BW25113, the highest ratio in this set of compounds. The compound continued to potentiate the activity of piperacillin in the *tolC*-deficient bacteria to a minor extent, which could be caused by off-target effects, something to consider in further optimization. However, the activity of levofloxacin was not potentiated by **4c**, a behavior similar to EPI PA*β*N, which is known to have a membrane-permeabilizing activity as off-target effect (Lamers et al., 2013). In uropathogenic *E. coli* UMN026, the compound was even more potent with an MPC_4_ of 1.56 µM, translating laboratory conditions to clinically relevant ones. However, the intrinsic antimicrobial effect was substantially lower for both compounds. Similarly, in BW25113, the MICs of **4c** and **4e** were low compared to other EPIs, for which no antimicrobial effect at concentrations of more than 500 µM have been reported (Plé et al., 2022). The increased antimicrobial activity for the TolC-selective compounds may indicate that inhibition of TolC increases the antimicrobial activity, presumably caused by interference with crucial physiological processes. EPIs and antibiotic adjuvants in general are expected to lack any intrinsic antibacterial properties (Wright, 2016), leading to the question of whether TolC-targeting compounds could be categorized as antibiotic adjuvants. To answer this question, more research concerning TolC inhibitors is needed.

The compounds in the present study provide insights into the chemical structure of compounds capable of binding to TolC and may guide the future search for compounds with higher affinity and better potentiation of antibiotics. This study further emphasizes that TolC represents a potential target for antibiotic adjuvants.

## Data Availability

The raw data supporting the conclusions of this article will be made available by the authors, without undue reservation.
