# Supplementary material for: Targeting the AcrAB-TolC efflux pump by small molecules selectively addressing the outer membrane factor TolC
Source: Front Pharmacol. 2026 Sep 24;17:1895729. doi: 10.3389/fphar.2026.1895729 (PMC13645612; doi:10.3389/fphar.2026.1895729)
Supplement: Supplementary file 1 [file DataSheet1.pdf]

Supplementary Material

1 Supplementary Data

Biological Assays

H33342-based accumulation assay

Bacteria were grown in Luria Broth (LB, HispanLab) for *E. coli* BW25113 and LB supplemented with 25 µg/mL of kanamycin (Sigma) for *E. coli* JW5503, at 37°C for 24 h under 200 rpm agitation. On the following day, overnight culture was inoculated into 5 mL of fresh LB and tubes incubated at 37° C at 200 rpm for about 2 h until the logarithmic phase was achieved (i.e. McFarland scale of 2.5 - 3.0). Cells were harvested by centrifugation (4,000 x g, 5 min) and resuspended in 1 × phosphate buffer saline [137 mM NaCl (Riedel), 2.7 mM KCl (Riedel), 10 mM Na_2HPO_4 (J.T.Baker); and 2 mM KH_2PO_4 (Merck); pH 7.4, PBS]. Resuspended cells were then diluted (1:4) in PBS. Compounds were added to the bacterial suspension in order to achieve a final concentration of 100 µM. As positive control PAβN was used at 25 µg/mL. Negative control constituted of PBS. Then, 180 µL of this mixture was added into 96-well black, clear bottom, microtiter plates (ViewPlate, Revvity). Plates were incubated at 37 °C for 15 min, and then H33342 was added to each well (2.5 mM final concentration). Plates were then covered in foil and mixed for 5 min at 500 rpm. H33342 efflux was monitored using a fluorescence microplate reader (VarioskanLUX, Thermo Fisher Scientific) to measure the fluorescence of the wells at 37°C every 5 min for 30 min. The excitation and emission wavelengths were set at 355nm and 460nm. Experiment was performed once with two technical replicates for each compound.

MIC-based assay for potentiation of antibiotics and antibacterial activity of compounds

Bacterial colonies were taken from the Mueller Hinton agar (MHA, Neogen) overnight culture, inoculated into 0.9% saline solution and vortexed to ensure that the bacterial suspension was homogeneous. Bacterial suspensions were analysed using a densitometer (DEN-1, BioSan, Warren MI, USA) and adjusted to 1 × 10^6 colony forming units (CFU/mL) by diluting with cation adjusted Mueller Hinton broth (CAMHB, BD). An equal volume of bacterial suspension and test compound solution, diluted into CAMHB containing piperacillin or levofloxacin (final concentrations of 1 µg/mL and 0.01 µg/mL, respectively), were mixed together into plate wells (Thermo Fisher Scientific) and incubated for 24 h at 37°C. Absorbance values measured at 600 nm using MultiskanGO plate reader (Thermo Fisher Scientific) were used for evaluating the antibacterial effects, by comparing to untreated controls, and expressed as percentage of growth inhibition. The MIC values were defined as the lowest compound's concentration at which bacterial growth was 90% inhibited compared to antibiotic- and compound-free control.

Synthesis of Compound S1

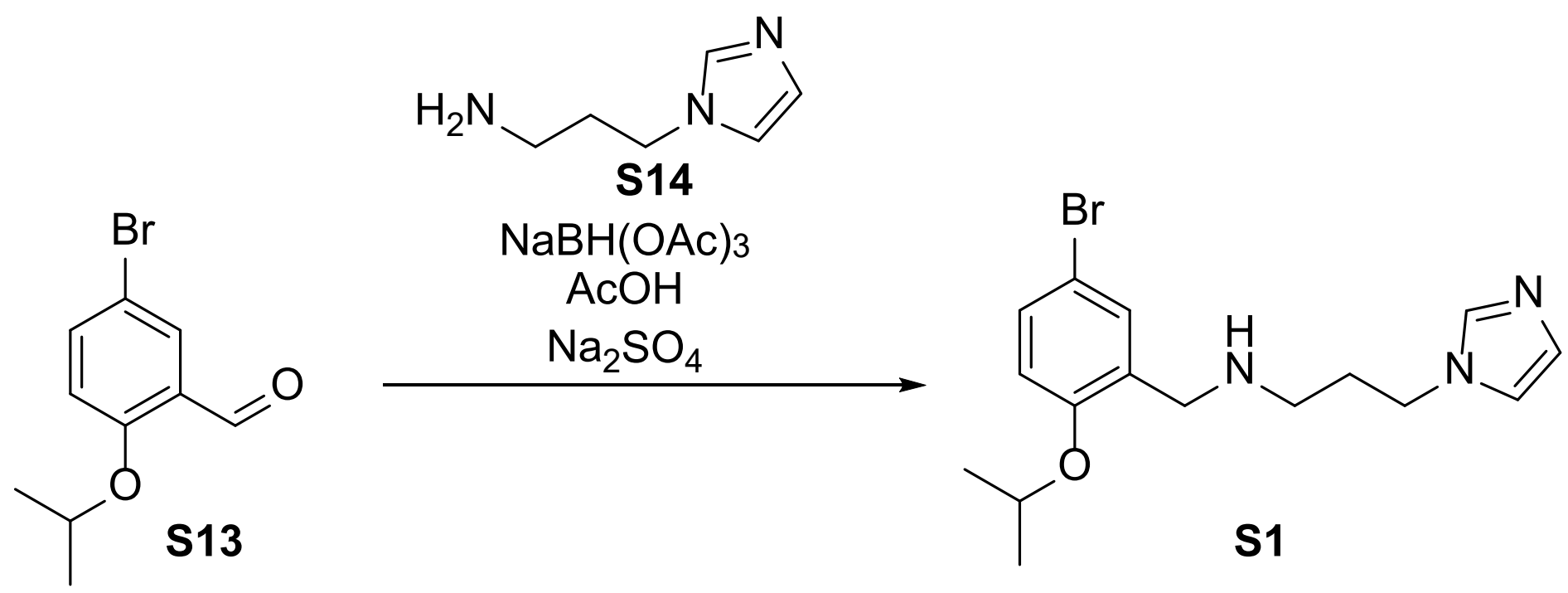

N-(5-bromo-2-isopropoxybenzyl)-3-(1H-imidazol-1-yl)propan-1-amine (***S1*****).**

To a solution of 5-bromo-2-isopropoxybenzaldehyde (**S13**) (0.408 g, 1.68 mmol) in dry methanol (6 ml), 3-(1H-imidazol-1-yl)propan-1-amine (**S14**) (0.210 g, 1.68 mmol) and anhydrous Na_2SO_4 (0.477 g, 3.36 mmol) were added. After stirring at room temperature for 30 min, $NaBH(OAc)_3$ (0.533 g, 2.52 mmol) was added in several portions followed by glacial acetic acid (0.096 ml, 1.68 mmol). The reaction mixture was stirred at room temperature for 24 h and evaporated to dryness.

The crude product was purified by silica gel column chromatography (CH_2Cl_2/MeOH 400:20_400:30_100:35 v/v) to obtain compound **S1** as a colorless oil (0.6*AcOH adduct, 0.319 g, 54 %).

Purity (HPLC): 98.1%

^{1}H NMR (DMSO-d_6, 400 MHz): δ (ppm) δ 7.59 (s, 1H), 7.48 (d, *J*=2.6 Hz, 1H), 7.32 (dd, *J*=8.7; 2.6 Hz, 1H), 7.13 (s, 1H), 6.93 (d, *J*=8.7 Hz, 1H), 6.87 (s, 1H), 4.57 (hept, *J*=6.0 Hz, 1H), 4.02 (t, *J*=7.0 Hz, 2H), 3.62 (s, 2H), 2.43 (t, *J*=6.7 Hz, 2H), 1.89 (s, 2H), 1.88-1.81 (m, 2H) 1.24 (d, *J*=6.0 Hz, 6H).

^{13}C NMR (DMSO-d_6, 100 MHz): δ (ppm) 172.3, 154.5, 137.2, 132.1, 131.3, 130.1, 128.2, 119.3, 115.1, 111.7, 70.0, 46.7, 45.3, 44.0, 30.8, 21.8, 21.3

ESI-MS (m/z): 352.2 $[M+H]^+$

HR-MS (ESI) (m/z): Calculated for $C_{16}H_{23}N_3OBr$ $[M+H]^+$ 352.1030, found 352.1038

N-(5-bromo-2-isopropoxybenzyl)-3-(1H-imidazol-1-yl)propan-1-amine (***S1***)

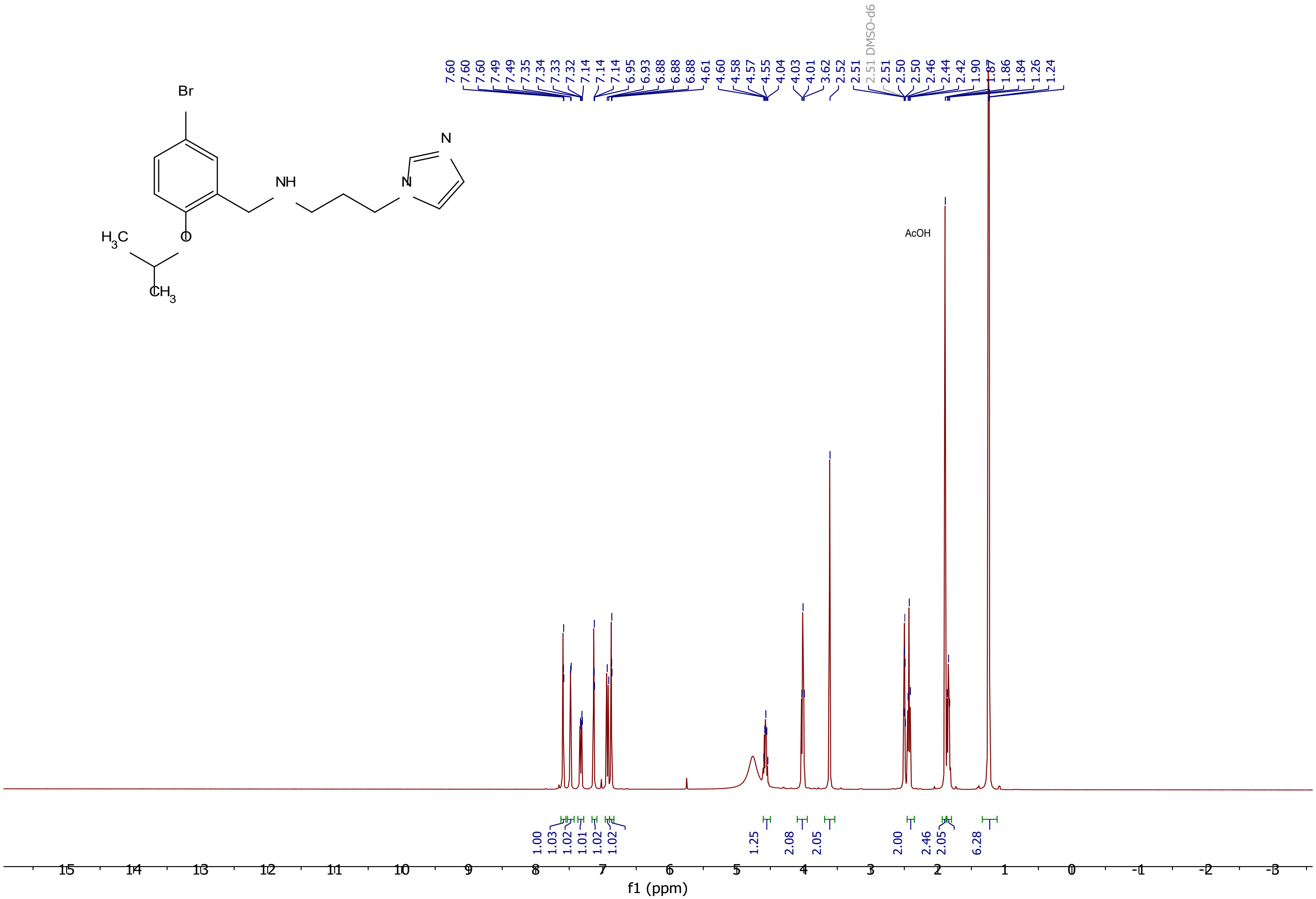

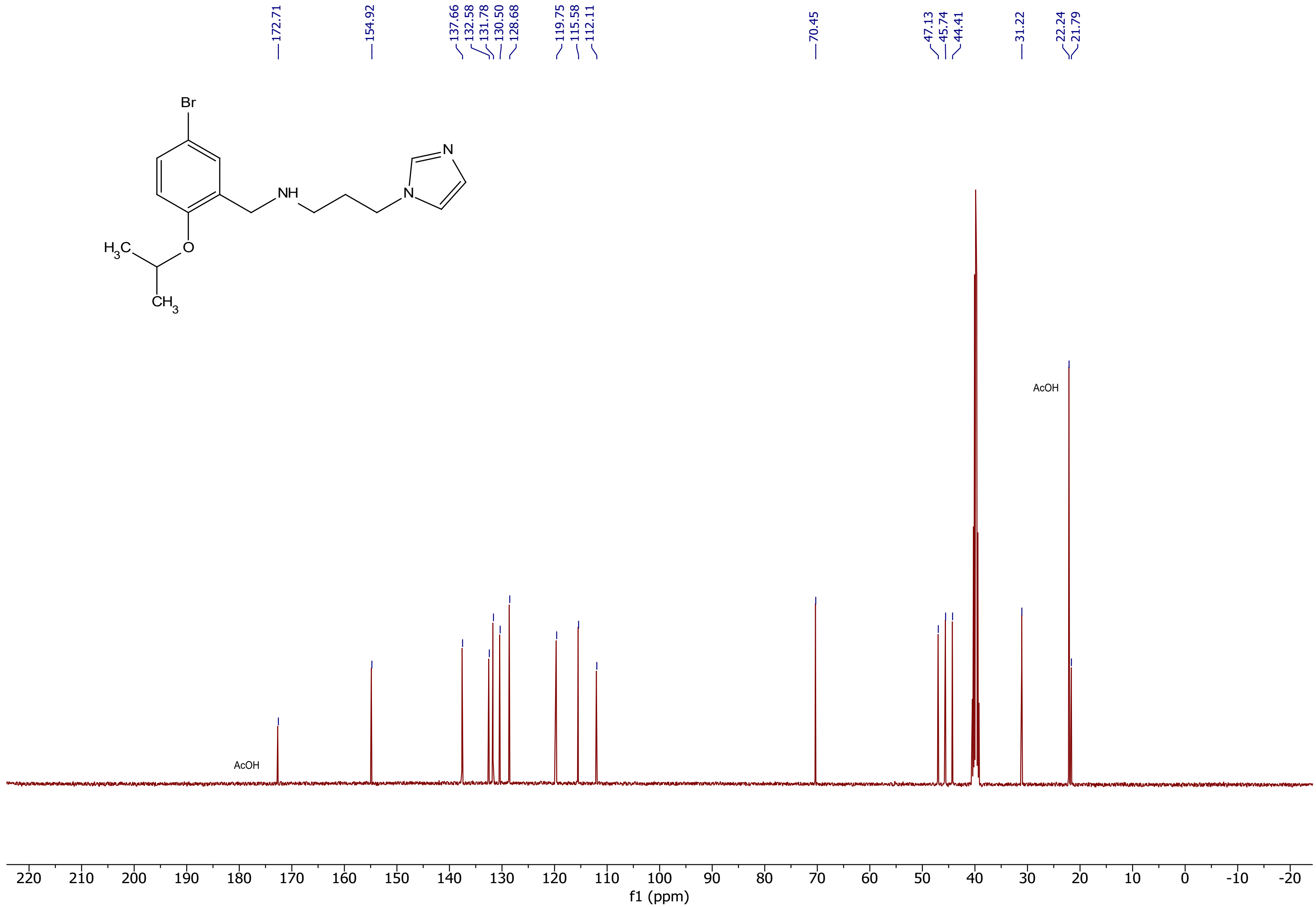
172.71
154.92
137.66
132.58
131.78
130.50
128.68
119.75
115.58
112.11
70.45
47.13
45.74
44.41
31.22
22.24
21.79
Br
NH
N
N
H_3C
O
CH_3
AcOH
AcOH
220 210 200 190 180 170 160 150 140 130 120 110 100 90 80 70 60 50 40 30 20 10 0 -10 -20
f1 (ppm)

Synthesis of Compounds 1a to 4e.

Scheme 1. Synthesis of compounds 1a-1c

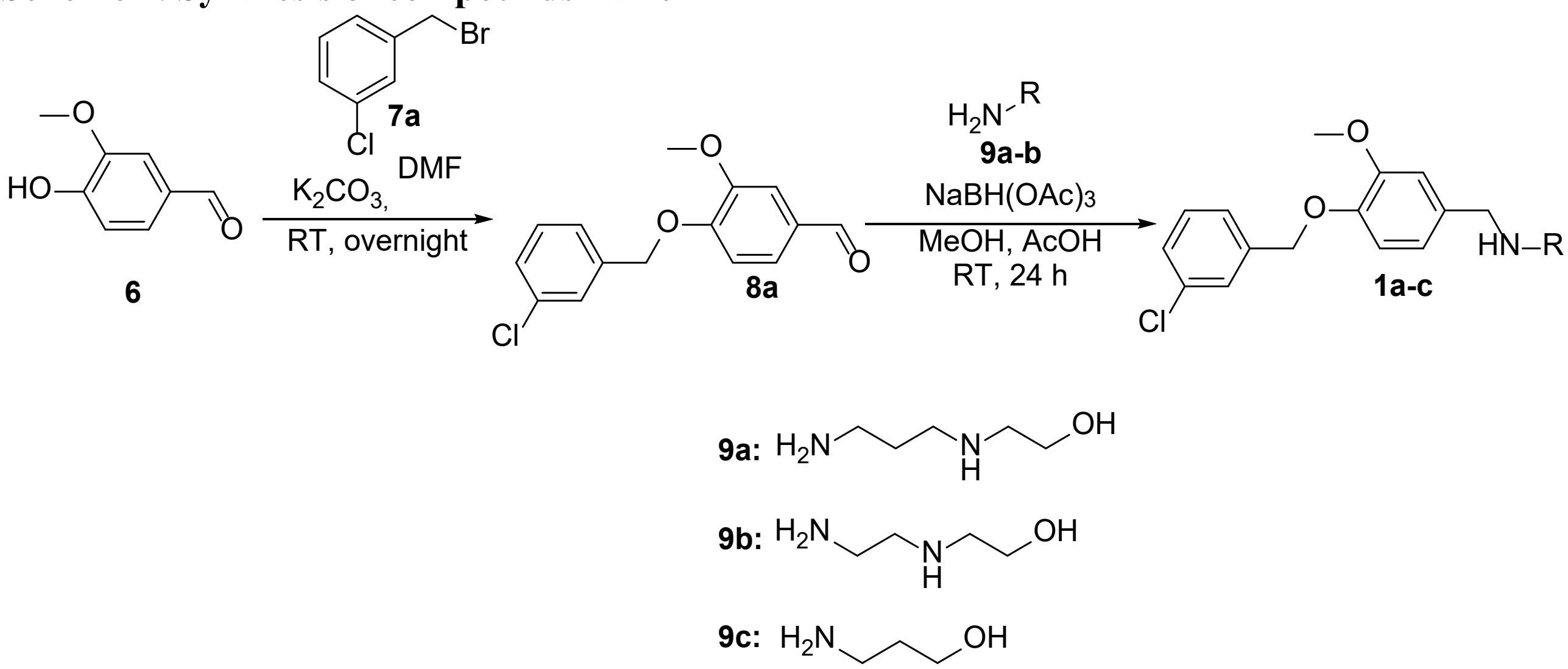

The initial hit compound **1a** and its related structural analogues **1b-1c** with altered polar side-chain (fragment C modifications) were synthesized according to Scheme 1. Benzylation of 4-hydroxy-3-methoxybenzaldehyde (**6**) with the 1-(bromomethyl)-3-chlorobenzene (**7a**) using potassium carbonate as a base in DMF afforded aldehyde **8a**. The reductive amination of intermediate **8a** with amines **9a-9b** using NaBH(OAc)3 as reducing agent resulted in the desired products **1a-1c**. Modification of the fragment A of parent structure **1a** was achieved according to Scheme 2. Benzylation of 4-hydroxy-3-methoxybenzaldehyde (**6**) with a range of different benzyl halides **7b-7z** and following reductive amination provided analogues **2b-2z**.

Analogues **2a-2f** with modified fragment B were synthesized according to Scheme 2, where catechol aldehyde **10** was subjected to the benzylation, alkylation and reductive amination sequence.

Scheme 2. Synthesis of compounds 2a-2f

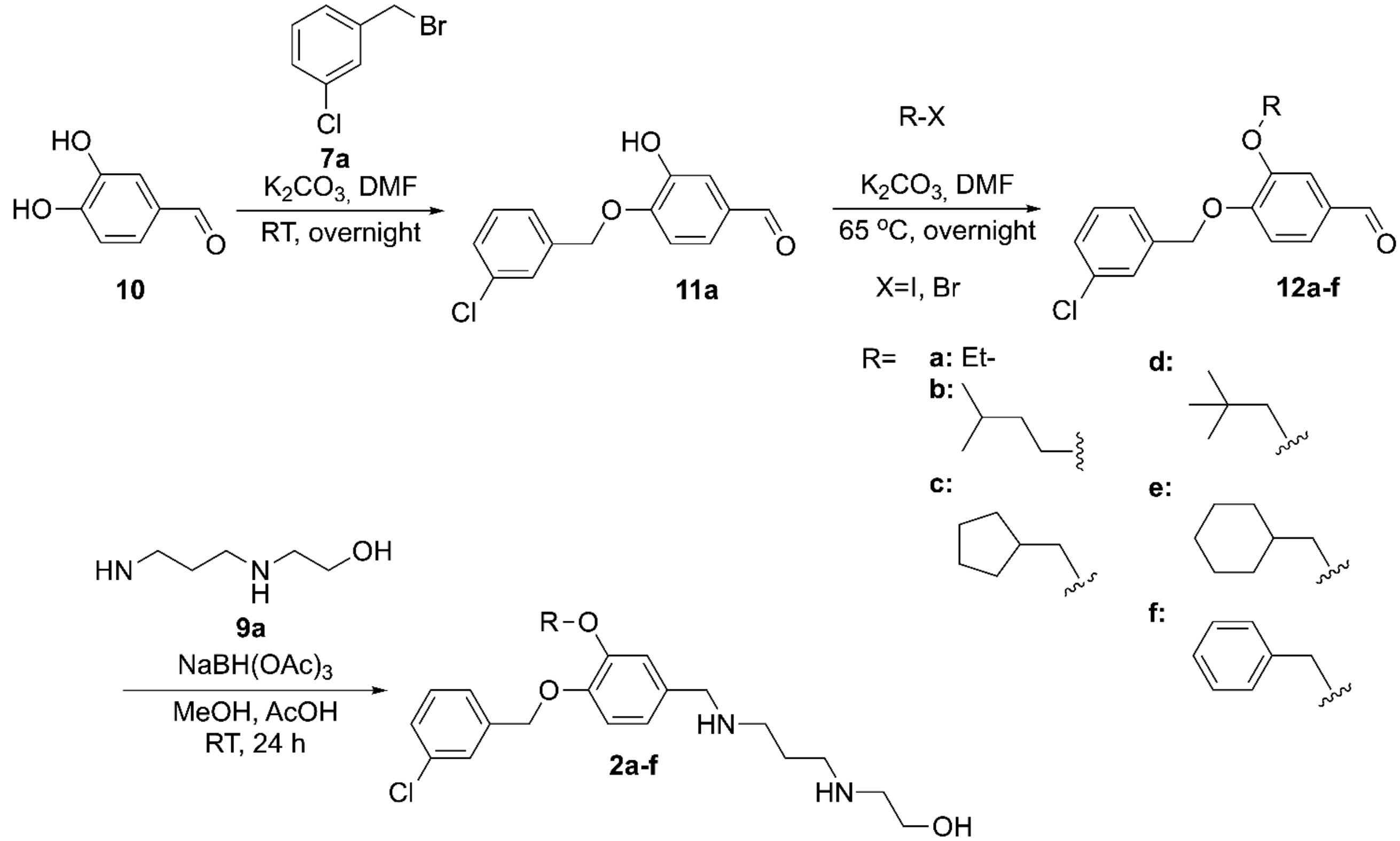

Scheme 3. Synthesis of compounds 3a-3w

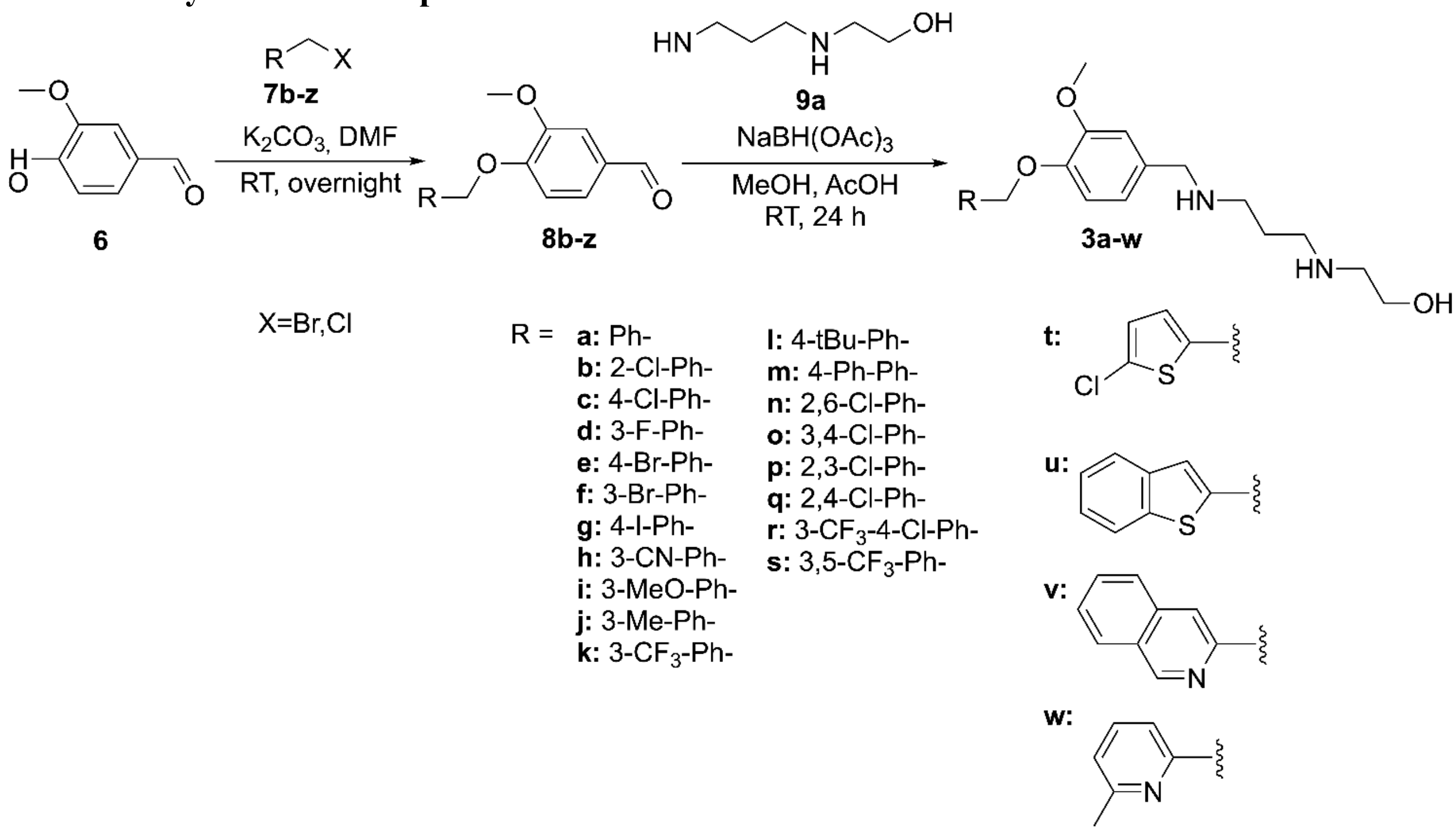

Compound **4a** was prepared in a similar sequence using 4-hydroxybenzaldehyde (**13**) as a starting material as outlined in Scheme 4.

Scheme 4. Synthesis of compound 4a

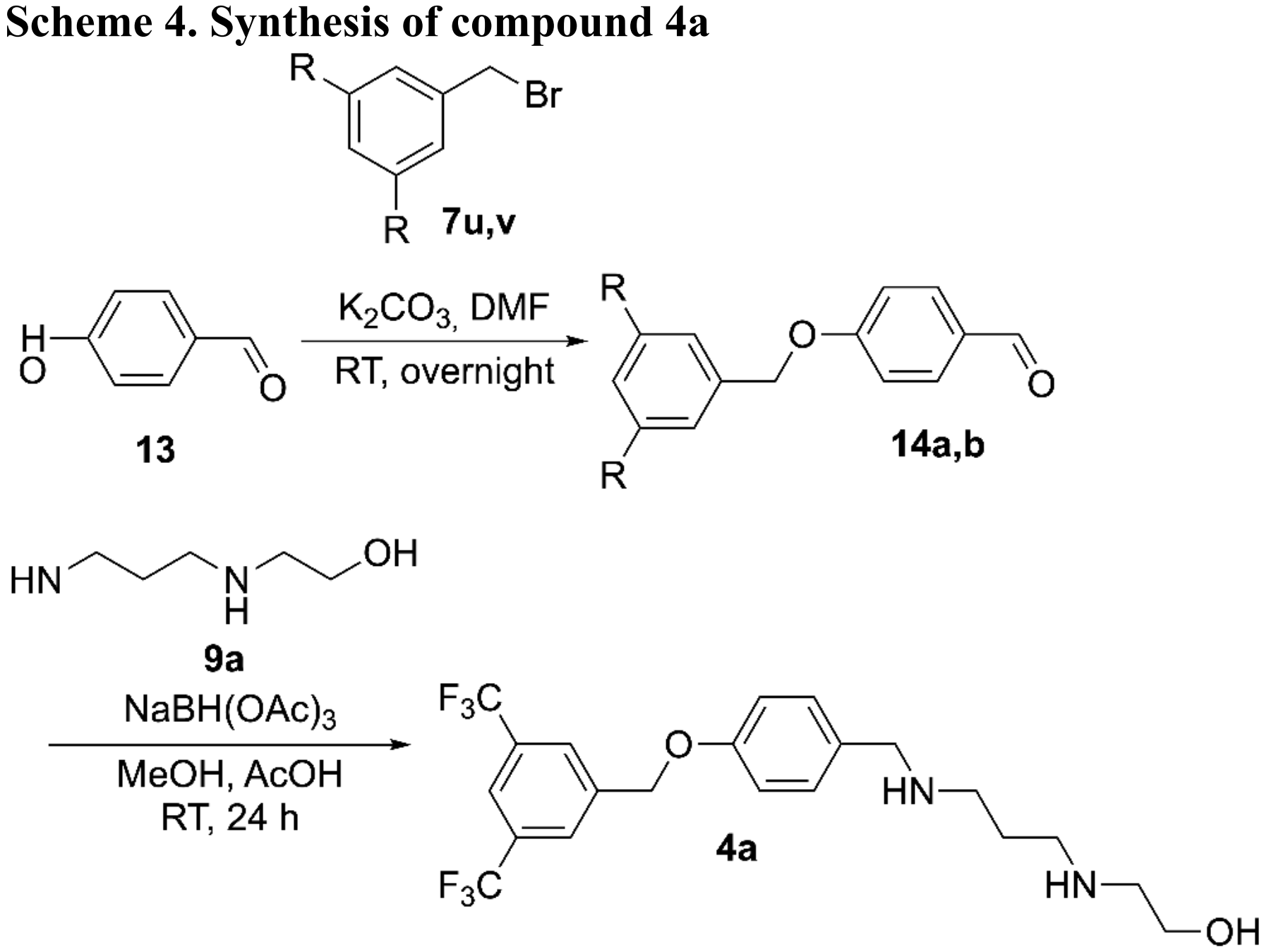

Dibenzylated analogues **4b-4e** were synthesized following the Scheme 5, where bis-trifluoromethylated benzylbromide (**7v**) was used to obtain a mixture of mono- and bis- benzylated aldehydes **15** and **16**, correspondingly. Bis-bezylated intermediate **16** was directly subjected to reductive amination with **9a** affording the desired product **4c**. However, the mono-substituted aldehyde **15** was repeatedly benzylated with **7q**, **7s** or **7za**, followed by the usual amination step with **9a** to afford the desired unsymmetrically substituted analogues **4.**

Scheme 5. Synthesis of compounds 4b-4e

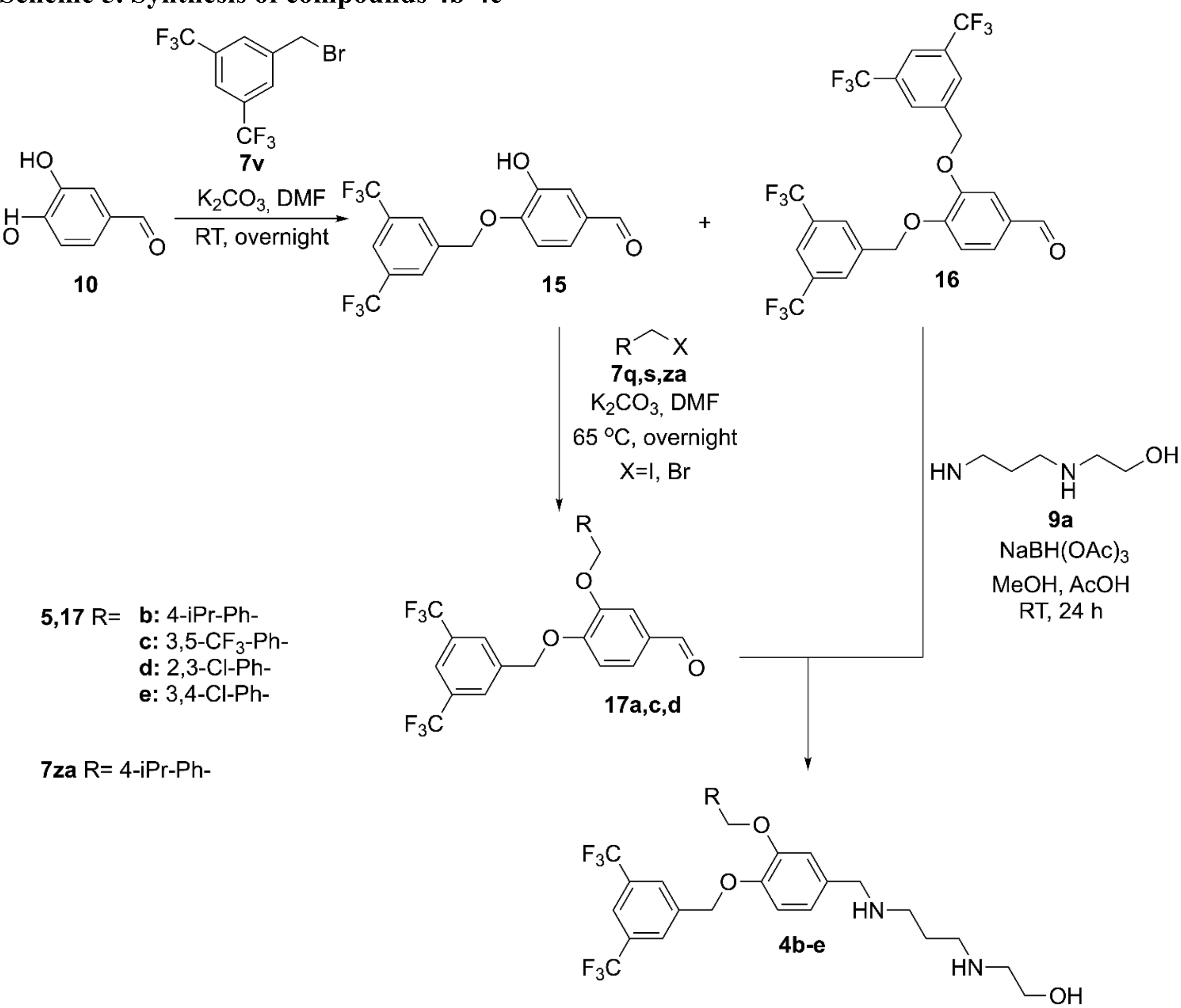

General procedure for reductive amination for the synthesis of compounds 1a to 1b and 3a to 3w
2-((3-((4-((3-Chlorobenzyl)oxy)-3-methoxybenzyl)amino)propyl)amino)ethan-1-ol (***1a*****).**
To a solution of compound **8a** (0.281 g, 1.02 mmol) in dry methanol (5 mL), N-(2-hydroxyethyl)-1,3-propanediamine (**9a**) (0.139 g, 0.14 mL, 1.17 mmmol) and anhydrous Na_2SO_4 (0.289 g, 2.03 mmol) were added. After stirring at room temperature for 30 min, $NaBH(OAc)_3$ (0.323 g, 1.52 mmol) was added in several portions followed by glacial acetic acid (0.139 g, 0.14 mL, 2.04 mmol). The reaction mixture was stirred at room temperature for 24 h and evaporated to dryness.
The crude product was purified by silica gel column chromatography (CH_2Cl_2/MeOH/25%NH_4OH 100:10:0_150:20:10_100:20:10 v/v) to obtain compound **1a** as a foam (0.221 g, 57.4%).
Purity (HPLC): 99.2%
^{1}H NMR ($CDCl_3$, 400 MHz): δ (ppm) δ 7.37 (dt, *J* = 2.1, 1.1 Hz, 1H), 7.27 – 7.17 (m, 3H), 6.84 (d, *J* = 1.8 Hz, 1H), 6.75 – 6.67 (m, 2H), 5.02 (s, 2H), 3.83 (s, 3H), 3.64 (s, 2H), 3.59 – 3.49 (m, 2H), 2.71 – 2.66 (m, 2H), 2.63 (td, *J* = 6.8, 1.2 Hz, 4H), 1.63 (p, *J* = 6.8 Hz, 2H)
^{13}C NMR (DMSO-d_6, 100 MHz): δ (ppm) 173.3, 149.1, 146.8, 139.9, 133.1, 130.7, 130.4, 127.8, 127.3, 126.2, 120.9, 113.6, 112.9, 69.1, 57.4, 55.6, 51.4, 49.6, 46.0, 45.6, 24.9, 22.3
ESI-MS (m/z): 379.3 $[M+H]^+$
HR-MS (ESI) (m/z): Calculated for $C_{20}H_{28}N_2O_3Cl[M+H]^+$ 379.1788, found 379.1798

2-((2-((4-((3-Chlorobenzyl)oxy)-3-methoxybenzyl)amino)ethyl)amino)ethan-1-ol (***1b*****).**
Compound **1b** was prepared in a similar manner as described for compound **1a** from aldehyde **8a** (0.304 g**,** 1.10 mmol) and 2-((2-aminoethyl)amino)ethan-ol (**9b**) (0.115 g, 1.10 mmol). Yield: 0.291 g (72.6%).
Viscous liquid
Purity (HPLC): 98.2%
^{1}H NMR (DMSO-d_6, 400 MHz): δ (ppm) 7.51-7.48 (m, 1H), 7.44-7.36 (m, 3H), 6.99 (d, *J*=2.0 Hz, 1H), 6.93 (d, *J*=8.2 Hz, 1H), 6.79 (dd, *J*=8.2, 2.0 Hz, 1H), 5.06 (s, 2H), 3.77 (s, 3H), 3.62 (s, 2H), 3.45 (t, *J*=5.7 Hz, 2H), 2.68-2.53 (m, 6H)
^{13}C NMR (DMSO-d_6, 100 MHz): δ (ppm) 149.1, 146.2, 140.0, 134.2, 133.1, 130.3, 127.7, 127.3, 126.1, 119.8, 113.7, 112.1, 69.2, 60.2, 55.5, 52.6, 51.5, 48.7, 48.1
ESI-MS (m/z): 365.2 $[M+H]^+$
HR-MS (ESI) (m/z): Calculated for $C_{19}H_{26}N_2O_3Cl$ $[M+H]^+$ 365.1632, found 365.1644

2-((4-((3-Chlorobenzyl)oxy)-3-methoxybenzyl)amino)ethan-1-ol (***1c***).
Compound **1c** was prepared in a similar manner as described for compound **1a** from aldehyde **8a** (0.313 g**,** 1.13 mmol) and 2-aminoethan-1-ol (**8c**) (0.085 g, 0.09 mL, 1.13 mmol). Yield: 0.274 g (64.2%).
Foam
Purity (HPLC): 97.8%
^{1}H NMR (DMSO-d_6, 400 MHz): δ (ppm) 7.51-7.48 (m, 1H), 7.44-7.35 (m, 3H), 6.99 (d, J=1.9 Hz, 1H), 6.93 (d, J=8.1 Hz, 1H), 6.80 (dd, J=8.1, 1.9 Hz, 1H), 5.06 (s, 2H), 3.77 (s, 3H), 3.61 (s, 2H), 3.47 (t, J=6.3 Hz, 2H), 2.55 (t, J=6.9 Hz, 2H), 1.58 (p, J=6.6 Hz, 2H)
^{13}C NMR (DMSO-d_6, 100 MHz): δ (ppm) 149.1, 146.2, 140.0, 134.1, 133.1, 130.3, 127.7, 127.3, 126.1, 119.8, 113.6, 112.1, 69.2, 59.7, 55.5, 52.8, 46.1, 32.5
ESI-MS (m/z): 336.2 $[M+H]^+$
HR-MS (ESI) (m/z): Calculated for $C_{18}H_{23}NO_3Cl$ $[M+H]^+$ 336.1366, found 336.1370

2-((3-((4-(Benzyloxy)-3-methoxybenzyl)amino)propyl)amino)ethan-1-ol (***3a*****).**
Compound **3a** was prepared in a similar manner as described for compound **1a** from 0.300 g (1.24 mmol) of intermediate **8b** and 0.146 g (0.15 mL, 1.24 mmol) of amine **9a**. Yield: 0.327 g (76.0%).

Viscous liquid
Purity (HPLC): 98.9%
^{1}H NMR (DMSO-d_6, 400 MHz): δ (ppm) 7.46-7.29 (m, 5H), 6.96 (d, *J*=1.9 Hz, 1H), 6.93 (d, *J*=8.1 Hz, 1H), 6.78 (dd, *J*=8.1, 1.9 Hz, 1H), 5.04 (s, 2H), 3.76 (s, 3H), 3.60 (s, 2H), 3.44 (t, *J*=5.8 Hz, 2H), 2.62-2.49 (m, 6H), 1.56 (p, *J*=6.9 Hz, 2H)
^{13}C NMR (DMSO-d_6, 100 MHz): δ (ppm) 149.0, 146.4, 137.4, 134.1, 128.4, 127.8, 127.7, 119.8, 113.5, 112.0, 70.0, 60.3, 55.5, 52.8, 51.8, 47.7, 47.0, 29.8
ESI-MS (m/z): 345.3 $[M+H]^+$
HR-MS (ESI) (m/z): Calculated for $C_{20}H_{29}N_2O_3$ $[M+H]^+$ 345.2178, found 345.2184

2-((3-((4-((2-Chlorobenzyl)oxy)-3-methoxybenzyl)amino)propyl)amino)ethan-1-ol (***3b***).
Compound **3b** was prepared in a similar manner as described for compound **1a** from 0.332 g (1.20 mmol) of intermediate **8c** and 0.142 g (0.14 mL, 1.20 mmol) of amine **9a**. Yield: 0.212 g (56.5%).
Viscous liquid
Purity (HPLC): 99.5%
^{1}H NMR (DMSO-d_6, 400 MHz): δ (ppm) 7.61-7.55 (m, 1H), 7.52-7.46 (m, 1H), 7.41-7.35 (m, 2H), 6.99 (d, *J*=1.9 Hz, 1H), 6.94 (d, *J*=8.2 Hz, 1H), 6.80 (dd, *J*=8.2, 1.9 Hz, 1H), 5.10 (s, 2H), 3.76 (s, 3H), 3.61 (s, 2H), 3.45 (t, *J*=5.7 Hz, 2H), 2.70-2.42 (m, 6H), 1.57 (p, *J*=6.9 Hz, 2H)
^{13}C NMR (DMSO-d_6, 100 MHz): δ (ppm) 149.1, 146.3, 134.7, 134.4, 132.5, 130.0, 129.7, 129.3, 127.4, 119.9, 113.6, 112.1, 67.6, 60.2, 55.5, 52.8, 51.7, 47.7, 47.0, 29.5
ESI-MS (m/z): 379.3 $[M+H]^+$
HR-MS (ESI) (m/z): Calculated for $C_{20}H_{28}N_2O_3Cl$ $[M+H]^+$ 379.1788, found 379.1786

2-((3-((4-((4-Chlorobenzyl)oxy)-3-methoxybenzyl)amino)propyl)amino)ethan-1-ol (***3c***).
Compound **3c** was prepared in a similar manner as described for compound **1a** from 0.322 g (1.2 mmol) of intermediate **8a** and 0.142 g (0.14 mL, 1.2 mmol) of amine **9a**. Yield: 0.307 g (67.5%).
Tan solid
Purity (HPLC): 99.6%
^{1}H NMR (DMSO-d_6, 400 MHz): δ (ppm) 7.52-7.40 (m, 4H), 6.97 (d, *J*=1.9 Hz, 1H), 6.91 (d, *J*=8.2 Hz, 1H), 6.78 (dd, *J*=8.2, 1.9 Hz, 1H), 5.04 (s, 2H), 3.76 (s, 3H), 3.59 (s, 2H), 3.44 (t, *J*=5.7 Hz, 2H), 2.70-2.39 (m, 6H), 1.55 (p, *J*=6.9 Hz, 2H)
^{13}C NMR (DMSO-d_6, 100 MHz): δ (ppm) 149.1, 146.2, 136.5, 134.3, 132.3, 129.5, 128.4, 119.8, 113.6, 112.0, 69.2, 60.3, 55.5, 52.8, 51.7, 47.7, 47.0, 29.7
ESI-MS (m/z): 379.3 $[M+H]^+$
HR-MS (ESI) (m/z): Calculated for $C_{20}H_{28}N_2O_3Cl$ $[M+H]^+$ 379.1785, found 379.1801

2-((3-((4-((3-Flurobenzyl)oxy)-3-methoxybenzyl)amino)propyl)amino)ethan-1-ol (***3d***).
Compound **3d** was prepared in a similar manner as described for compound **1a** from 0.260 g (1.0 mmol) of intermediate **8e** and 0.118 g (0.12 mL, 1.0 mmol) of amine **9a**. Yield: 0.247 g (68.2%).
Viscous liquid
Purity (HPLC): 99.2%
^{1}H NMR (DMSO-d_6, 400 MHz): δ (ppm) 7.81 (s, 1H), 7.76-7.72 (m, 1H), 7.70-7.66 (m, 1H), 7.65—7.60 (m, 1H), 6.98 (d, *J*=1.9 Hz, 1H), 6.95 (d, *J*=8.2 Hz, 1H), 6.80 (dd, *J*=8.2, 1.9 Hz, 1H), 5.15 (s, 2H), 3.77 (s, 3H), 3.60 (s, 2H), 3.45 (t, *J*=5.7 Hz, 2H), 2.65-2.46 (m, 6H), 1.56 (p, *J*=6.9 Hz, 2H)
^{13}C NMR (DMSO-d_6, 100 MHz): δ (ppm) 162.2 (d, J_{CF}=243.7 Hz), 149.1, 146.2, 140.4, 134.3, 130.5 (d, J_{CF}=8.3 Hz), 123.5 (d, J_{CF}=2.6 Hz), 119.8, 114.5 (d, J_{CF}=20.8 Hz), 114.2 (d, J_{CF}=21.9 Hz), 113.7, 112.1, 69.2, 60.2, 55.5, 52.8, 51.7, 47.68, 47.00, 30.0

^{19}F NMR (DMSO-d$_6$, 376 MHz): δ (ppm) -113.00
ESI-MS (m/z): 363.3 [M+H]$^+$
HR-MS (ESI) (m/z): Calculated for $C_{20}H_{28}N_2O_3F$ [M+H]$^+$ 363.2084, found 363.2089

2-((3-((4-((4-Bromobenzyl)oxy)-3-methoxybenzyl)amino)propyl)amino)ethan-1-ol (***3e***)**.**
Compound **3e** was prepared in a similar manner as described for compound **1a** from 0.321 g (1.0 mmol) of intermediate **8f** and 0.118 g (0.12 mL, 1.0 mmol) of amine **9a**. Yield: 0.246 g (58.6%).
Foam
Purity (HPLC): 99.5%
^{1}H NMR (DMSO-d$_6$, 400 MHz): δ (ppm) 7.61-7.55 (m, 2H), 7.42-7.36 (m, 2H), 6.97 (d, *J*=1.9 Hz, 1H), 6.91 (d, *J*=8.1 Hz, 1H), 6.78 (dd, *J*=8.1, 1.9 Hz, 1H), 5.02 (s, 2H), 3.76 (s, 3H), 3.59 (s, 2H), 3.44 (t, *J*=5.7 Hz, 2H), 2.62-2.48 (m, 6H), 1.55 (p, *J*=6.9 Hz, 2H)
^{13}C NMR (DMSO-d$_6$, 100 MHz): δ (ppm) 149.1, 146.2, 136.9, 134.3, 131.3, 129.8, 120.8, 119.8, 113.6, 112.0, 69.2, 60.2, 55.5, 52.8, 51.7, 47.7, 47.0, 29.7
ESI-MS (m/z): 425.2 [M+H]$^+$
HR-MS (ESI) (m/z): Calculated for $C_{20}H_{28}N_2O_3Br$ [M+H]$^+$ 423.1283, found 423.1287
*2-((3-((4-((3-Bromobenzyl)oxy)-3-methoxybenzyl)amino)propyl)amino)ethan-1-ol (**3f**).*
Compound **3f** was prepared in a similar manner as described for compound **1a** from 0.321 g (1.0 mmol) of intermediate **8g** and 0.118 g (0.12 mL, 1.0 mmol) of amine **9a**. Yield: 0.260 g (61.9%).
Viscous liquid
Purity (HPLC): 98.6%
^{1}H NMR (DMSO-d$_6$, 400 MHz): δ (ppm) 7.66-7.62 (m, 1H), 7.54-7.49 (m, 1H), 7.46-7.41 (m, 1H), 7.35 (t, *J*=7.8 Hz, 1H), 7.01-6.96 (m, 1H), 6.92 (d, J=8.1 Hz, 1H), 6.79 (dd, J=8.1, 1.9 Hz, 1H), 5.05 (s, 2H), 3.76 (s, 3H), 3.60 (s, 2H), 3.45 (t, J=5.7 Hz, 2H), 2.68-2.47 (m, 6H), 1.57 (p, J=6.8 Hz, 2H)
^{13}C NMR (DMSO-d$_6$): δ (ppm) 149.1, 146.2, 140.3, 134.2, 130.6, 130.5, 130.2, 126.5, 121.6, 119.8, 113.7, 112.0, 69.1, 60.1, 55.5, 52.7, 51.5, 47.6, 46.9, 29.4
ESI-MS (m/z): 425.2 [M+H]$^+$
HR-MS (ESI) (m/z): Calculated for $C_{20}H_{28}N_2O_3Br$ [M+H]$^+$ 423.1283, found 423.1288

2-((3-((4-((4-Iodobenzyl)oxy)-3-methoxybenzyl)amino)propyl)amino)ethan-1-ol (***3g***).
Compound **3g** was prepared in a similar manner as described for compound **1a** from 0.376 g (1.02 mmol) of intermediate **8h** and 0.121 g (0.12 mL, 1.02 mmol) of amine **9a**. Yield: 0.346 g (72.1%).
White solid
Purity (HPLC): 99.3%
^{1}H NMR (DMSO-d$_6$, 400 MHz): δ (ppm) 7.77-7.71 (m, 2H), 7.27-7.20 (m, 2H), 6.96 (d, *J*=1.9 Hz, 1H), 6.90 (d, *J*=8.1 Hz, 1H), 6.77 (dd, *J*=8.1, 1.9 Hz, 1H), 5.00 (s, 2H), 3.75 (s, 3H), 3.59 (s, 2H), 3.44 (t, *J*=5.7 Hz, 2H), 2.68-2.49 (m, 6H), 1.55 (p, *J*=6.9 Hz, 2H)
^{13}C NMR (DMSO-d$_6$, 100 MHz): δ (ppm) 149.0, 146.2, 137.2, 137.1, 134.2, 129.8, 119.8, 113.6, 112.0, 93.8, 69.4, 60.2, 55.5, 52.8, 51.7, 47.7, 47.0, 29.6
ESI-MS (m/z): 471.2 [M+H]$^+$
HR-MS (ESI) (m/z): Calculated for $C_{20}H_{28}N_2O_3I$ [M+H]$^+$ 471.1145, found 471.1155

3-((4-(((3-((2-Hydroxyethyl)amino)propyl)amino)methyl)-2-methoxyphenoxy)- benzonitrile (***3h***).
Compound **3h** was prepared in a similar manner as described for compound **1a** from 0.267 g (1.0 mmol) of intermediate **8i** and 0.118 g (0.12 mL, 1.0 mmol) of amine **9a**. Yield: 0.182 g (49.3%).
Viscous liquid
Purity (HPLC): 98.3%

^{1}H NMR (DMSO-d_6, 400 MHz): δ (ppm) 7.89-7.85 (m, 1H), 7.82-7.75 (m, 2H), 7.60 (t, *J*=7.7 Hz, 1H), 6.98 (d, *J*=1.9 Hz, 1H), 6.94 (d, *J*=8.1 Hz, 1H), 6.79 (dd, *J*=8.1, 1.9 Hz, 1H), 5.11 (s, 2H), 3.77 (s, 3H), 3.60 (s, 2H), 3.44 (t, *J*=5.7 Hz, 2H), 2.66-2.40 (m, 6H), 1.56 (p, *J*=6.9 Hz, 2H)
^{13}C NMR (DMSO-d_6, 100 MHz): δ (ppm) 149.1, 146.0, 139.2, 134.5, 132.4, 131.6, 131.0, 129.7, 119.8, 118.7, 113.8, 112.1, 111.4, 69.0, 60.2, 55.5, 52.8, 51.7, 47.7, 47.0, 29.6
ESI-MS (m/z): 370.3 $[M+H]^+$
HR-MS (ESI) (m/z): Calculated for $C_{21}H_{28}N_3O_3$ $[M+H]^+$ 370.2131, found 370.2134
2-((3-((3-Methoxy-4-((3-methoxybenzyl)oxy)benzyl)amino)propyl)amino)ethan-1-ol (***3i***).
Compound **3i** was prepared in a similar manner as described for compound **1a** from 0.272 g (1.0 mmol) of intermediate **8j** and 0.118 g (0.12 mL, 1.0 mmol) of amine **9a**. Yield: 0.220 g (58.8%).
Viscous liquid
Purity (HPLC): 98.6%
^{1}H NMR (DMSO-d_6, 400 MHz): δ (ppm) 7.33-7.27 (m, 1H), 7.04-6.96 (m, 3H), 6.93 (d, *J*=8.2 Hz, 1H), 6.91-6.86 (m, 1H), 6.79 (dd, *J*=8.2, 1.9 Hz, 1H), 5.03 (s, 2H), 3.77 (s, 3H), 3.76 (s, 3H), 3.61 (s, 2H), 3.45 (t, *J*=5.7 Hz, 2H), 2.67-2.48 (m, 6H), 1.57 (p, *J*=6.9 Hz, 2H)
^{13}C NMR (DMSO-d_6, 100 MHz): δ (ppm) 159.3, 149.1, 146.4, 139.0, 133.9, 129.5, 119.8, 119.7, 113.5, 113.2, 113.1, 112.1, 69.9, 55.5, 55.0, 54.9, 47.7, 47.0, 29.5
ESI-MS (m/z): 375.3 $[M+H]^+$
HR-MS (ESI) (m/z): Calculated for $C_{21}H_{31}N_2O_4$ $[M+H]^+$ 375.2284, found 375.2279

2-((3-((3-Methoxy-4-((3-methybenzyl)oxy)benzyl)amino)propyl)amino)ethan-1-ol (***3j***).
Compound **3j** was prepared in a similar manner as described for compound **1a** from 0.333 g (1.30 mmol) of intermediate **8k** and 0.169 g (0.17 mL, 1.43 mmol) of amine **9a**. Yield: 0.371 g (79.6%).
Viscous liquid
Purity (HPLC): 99.6%
^{1}H NMR (DMSO-d_6, 400 MHz): δ (ppm) 7.30-7.19 (m, 3H), 7.16-7.10 (m, 1H), 6.97 (d, *J*=1.9 Hz, 1H), 6.92 (d, *J*=8.1 Hz, 1H), 6.79 (dd, *J*=8.1, 1.9 Hz, 1H), 4.99 (s, 2H), 3.76 (s, 3H), 3.60 (s, 2H), 3.45 (t, *J*=5.7 Hz, 2H), 2.68-2.45 (m, 6H), 2.31 (s, 3H), 1.56 (p, *J*=6.9 Hz, 2H)
^{13}C NMR (DMSO-d_6, 100 MHz): δ (ppm) 149.0, 146.5, 137.5, 137.3, 134.0, 128.4, 128.3, 128.3, 124.8, 119.8, 113.4, 112.0, 70.1, 60.2, 55.5, 52.8, 51.7, 47.7, 47.0, 29.6, 21.0
ESI-MS (m/z): 359.3 $[M+H]^+$
HR-MS (ESI) (m/z): Calculated for $C_{21}H_{31}N_2O_3$ $[M+H]^+$ 359.2335, found 359.2334

2-((3-((3-Methoxy-4-((3-trifluoromethylbenzyl)oxy)benzyl)amino)propyl)amino)- ethan-1-ol (***3k***).
Compound **3k** was prepared in a similar manner as described for compound **1a** from 0.341 g (1.1 mmol) of intermediate **8l** and 0.129 g (0.13 mL, 1.1 mmol) of amine **9a**. Yield: 0.223 g (49.1%).
Yellowish foam
Purity (HPLC): 99.5%
^{1}H NMR (DMSO-d_6, 400 MHz): δ (ppm) 7.81 (s, 1H), 7.76-7.72 (m, 1H), 7.70-7.66 (m, 1H), 7.65—7.60 (m, 1H), 6.98 (d, *J*=1.9 Hz, 1H), 6.95 (d, *J*=8.2 Hz, 1H), 6.80 (dd, *J*=8.2, 1.9 Hz, 1H), 5.15 (s, 2H), 3.77 (s, 3H), 3.60 (s, 2H), 3.45 (t, *J*=5.7 Hz, 2H), 2.65-2.46 (m, 6H), 1.56 (p, *J*=6.9 Hz, 2H)
^{13}C NMR (DMSO-d_6, 100 MHz): δ (ppm) 149.2, 146.1, 139.0, 134.5, 131.6, 129.5, 129.1 (q, J_{CF}=31.3 Hz), 124.5 (q, J_{CF}=3.8 Hz), 124.2 (q, J_{CF}=272.4 Hz), 124.0 (q, J_{CF}=3.8 Hz), 119.8, 113.9, 112.1, 69.4, 60.2, 55.5, 52.8, 51.8, 47.7, 47.1, 29.7
^{19}F NMR (DMSO-d_6, 376 MHz): δ (ppm) -61.01
ESI-MS (m/z): 413.3 $[M+H]^+$
HR-MS (ESI) (m/z): Calculated for $C_{21}H_{28}N_2O_3F_3$ $[M+H]^+$ 413.2052, found 413.2045

*2-((3-((4-((4-(tert-Butyl)benzyl)oxy)-3-methoxybenzyl)amino)propyl)amino)eth-an-1-ol (**3l**).*
Compound **3l** was prepared in a similar manner as described for compound **1a** from 0.343 g (1.15 mmol) of intermediate **8n** and 0.136g (0.14 mL, 1.15 mmol) of amine **9a**. Yield: 0.311 g (66.6%).
Foam
Purity (HPLC): 96.9%
^{1}H NMR (DMSO-d$_6$, 400 MHz): δ (ppm) 7.43-7.32 (m, 4H), 6.96 (d, *J*=1.9 Hz, 1H), 6.93 (d, *J*=8.2 Hz, 1H), 6.79 (dd, *J*=8.2, 1.9 Hz, 1H), 4.99 (s,2H), 3.75 (s, 3H), 3.60 (s, 2H), 3.45 (t, *J*=5.7 H, 2H), 2.64-2.47 (m, 6H), 1.56 (p, *J*=6.9 Hz, 2H), 1.28 (s, 9H)
^{13}C NMR (DMSO-d$_6$, 100 MHz): δ (ppm) 150.2, 149.0, 146.6, 134.3, 133.7, 127.7, 125.1, 119.8, 113.2, 112.0, 69.8, 60.1, 55.4, 52.8, 51.7, 47.7, 47.0, 34.3, 31.1, 29.5
ESI-MS (m/z): 401.3 $[M+H]^+$
HR-MS (ESI) (m/z): Calculated for $C_{24}H_{37}N_2O_3$ $[M+H]^+$ 401.2804, found 401.2805

*2-((3-((4-([1,1'-Biphenyl]-4-ylmethoxy)-3-methoxybenzyl)amino)propyl)amino)ethan-1-ol (**3m**).*
Compound **3m** was prepared in a similar manner as described for compound **1a** from 0.343 g (1.15 mmol) of intermediate **8o** and 0.340 g (1.07 mmol) of amine **9a**. Yield: 0.311 g (66.6 %).
Solid (mp = 77-78°C).
Purity (HPLC): 98.4%
^{1}H NMR (400 MHz, CDCl$_3$): δ (ppm): 7.63 – 7.55 (m, 4H), 7.53 – 7.48 (m, 2H), 7.47 – 7.39 (m, 2H), 7.38 – 7.31 (m, 1H), 6.92 (d, *J* = 1.9 Hz, 1H), 6.86 (d, *J* = 8.2 Hz, 1H), 6.78 (dd, *J* = 8.2, 1.9 Hz, 1H), 5.18 (s, 2H), 3.91 (s, 3H), 3.72 (s, 2H), 3.68 – 3.59 (m, 2H), 2.77 (t, *J* = 5.2 Hz, 2H), 2.72 (td, *J* = 6.8, 2.2 Hz, 4H), 1.71 (p, *J* = 6.8 Hz, 2H).
^{13}C NMR (100 MHz, CDCl$_3$): δ (ppm) 149.8, 147.3, 140.8, 140.8, 136.3, 133.2, 128.8, 127.8, 127.3, 127.3, 127.1, 120.4, 114.0, 112.1, 70.9, 60.5, 56.1, 53.7, 51.0, 47.9, 47.6, 29.4.
ESI-MS (m/z):421.4 $[M+H]^+$
HRMS (ESI) (m/z): Calculated for $C_{26}H_{33}N_2O_3$ $[M+H]^+$ 421.2502, found 421.2491

2-((3-((4-((2,6-Dichlorobenzyl)oxy)-3-methoxybenzyl)amino)propyl)amino)ethan-1-ol (***3n***).
Compound **3n** was prepared in a similar manner as described for compound **1a** from 0.311 g (1.0 mmol) of intermediate **8p** and 0.118 g (0.12 mL, 1.0 mmol) of amine **9a**. Yield: 0.218 g (52.8%).
Foam
Purity (HPLC): 99.4%
^{1}H NMR (DMSO-d$_6$, 400 MHz): δ (ppm) 7.58-7.52 (m, 2H), 7.45 (dd, *J*=8.9, 7.1 Hz, 1H), 7.02 (d, *J*=8.1 Hz, 1H), 6.97 (d, *J*=1.9 Hz, 1H), 6.83 (dd, *J*=8.1, 1.9 Hz, 1H), 5.17 (s, 2H), 3.72 (s, 3H), 3,61 (s, 2H), 3.44 (t, *J*=5.7 Hz, 2H), 2.62-2.48 (m, 6H), 1.56 (p, *J*=6.9 Hz, 2H)
^{13}C NMR (DMSO-d$_6$, 100 MHz): δ (ppm) 149.2, 146.4, 136.2, 134.8, 132.0, 131.5, 128.7, 119.8, 113.8, 112.1, 65.7, 60.3, 55.4, 52.8, 51.8, 47.8, 47.1, 29.9
ESI-MS (m/z): 413.2 $[M+H]^+$
HR-MS (ESI) (m/z): Calculated for $C_{20}H_{27}N_2O_3Cl_2$ $[M+H]^+$ 413.1399, found 413.1405.

2-((3-((4-((3,4-Dichlorobenzyl)oxy)-3-methoxybenzyl)amino)propyl)amino)ethan-1-ol (***3o***)**.**
Compound **3o** was prepared from 0.350 g (1.12 mmol) of intermediate **8q** and 0.133 g (0.132 mL, 1.12 mmol) amine **9a**. Yield 0.335 g (72.0%).
Colourless oil.
Purity (HPLC): 97.9%

^{1}H NMR (400 MHz, $CDCl_3$): δ (ppm) 7.54 (d, J = 2.0 Hz, 1H), 7.42 (d, J = 8.2 Hz, 1H), 7.29 – 7.22 (m, 1H), 6.91 (br, s, 1H), 6.80 – 6.74 (m, 2H), 5.06 (s, 2H) 3.89 (s, 3H), 3.71 (s, 2H), 3.65 – 3.60 (m, 2H), 2.79 – 2.73 (m, 2H), 2.70 (td, J = 6.8, 2.6 Hz, 4H), 2.37 (br, s, 3H), 1.70 (p, J = 6.8 Hz, 2H).
^{13}C NMR (100 MHz, $CDCl_3$): δ (ppm) 149.8, 146.6, 137.6, 134.3, 132.7, 131.8, 130.5, 129.2, 126.5, 120.2, 114.3, 112.0, 69.9, 60.8, 56.0, 53.8, 51.0, 47.8, 47.8, 30.1.
ESI-MS (m/z): 414.5 $[M+H]^+$
HRMS (ESI) (m/z): Calculated for $C_{20}H_{27}Cl_2N_2O_3$ $[M+H]^+$ 413.1410, found 413.1399.

2-((3-((4-((4-Chloro-3-(trifluoromethyl)benzyl)oxy)-3-methoxybenzyl)amino)propyl)amino)ethan-1-ol (***3r***).
Compound **3r** was prepared from 0.386 g (1.12 mmol) of intermediate **8r** and 0.133 g (0.132 mL, 1.12 mmol) amine **9a**. Yield 0.334 g (67.3%).
Off-white solid.
Purity (HPLC): 99.2%
^{1}H NMR (400 MHz, $CDCl_3$): δ (ppm) 7.78 (d, J = 2.1 Hz, 1H), 7.55 (dd, J = 8.2, 2.1 Hz, 1H), 7.49 (d, J = 8.3 Hz, 1H), 6.93 (s, 1H), 6.82-6.77 (m, 2H), 5.12 (s, 2H), 3.90 (s, 3H), 3.73 (s, 2H), 3.63 (t, J = 5.2 Hz, 2H), 2.77 (t, J = 5.2 Hz, 2H), 2.72 (td, J = 6.8, 2.1 Hz, 4H), 2.02 (br s, 3H), 1.71 (p, J = 6.8 Hz, 2H).
^{13}C NMR (100 MHz, $CDCl_3$): δ (ppm) 150.0, 146.7, 136.7, 134.5, 131.73, 131.68, 131.68-131.65 (m), 128.6 (q, J_{CF} = 31.3 Hz), 126.5 (q, J_{CF} = 5.4 Hz), 122.8 (q, J_{CF} = 274 Hz), 120.3, 114.7, 112.2, 70.3, 60.8, 56.0, 53.9, 51.2, 48.0, 47.8, 30.0.
^{19}F NMR (376 MHz, $CDCl_3$): δ (ppm) -62.62.
ESI-MS (m/z): 447.2 $[M+H]^+$
HRMS (ESI) (m/z): Calculated for $C_{21}H_{27}ClF_3N_2O_3$ $[M+H]^+$ 447.1662, found 447.1675.

2-((3-((4-((2,3-Dichlorobenzyl)oxy)-3-methoxybenzyl)amino)propyl)amino)ethan-1-ol (***3p*).**
Compound **3p** was prepared in a similar manner as described for compound **1a** from 0.311 g (1.0 mmol) of intermediate **2s** and 0.130 g (0.13 mL, 1.1 mmol) of amine **9a**. Yield: 0.279 g (67.6%).
Foam
Purity (HPLC): 98.5%
^{1}H NMR (DMSO-d$_6$, 400 MHz): δ (ppm) 7.62 (dd, J=8.0, 1.6 Hz, 1H), 7.56 (dd, J=7.9, 1.6 Hz, 1H), 7.40 (t, J=7.9 Hz, 1H), 6.99 (d, J=1.9 Hz, 1H), 6.93 (d, J=8.2 Hz, 1H), 6.80 (dd, J=8.2, 1.9 Hz, 1H), 5.13 (s, 2H), 3.77 (s, 3H), 3.60 (s, 2H), 3.44 (t, J=5.7 Hz, 2H), 2.68-2.47 (m, 6H), 1.56 (p, J=6.9 Hz, 2H)
^{13}C NMR (DMSO-d$_6$, 100 MHz): δ (ppm) 149.1, 146.0, 137.4, 134.7, 131.9, 130.3, 130.0, 128.3, 128.2, 120.0, 113.7, 112.1, 68.0, 60.2, 55.5, 52.8, 51.8, 47.8, 47.1, 29.7
ESI-MS (m/z): 413.2 $[M+H]^+$
HR-MS (ESI) (m/z): Calculated for $C_{20}H_{27}N_2O_3Cl_2$ $[M+H]^+$ 413.1399, found 413.1409

2-((3-((4-((2,4-Dichlorobenzyl)oxy)-3-methoxybenzyl)amino)propyl)amino)ethan-1-ol (***3q***).
Compound 3q was prepared from 0.270 g (0.87 mmol) of intermediate **8t** and 0.103 g (0.10 mL, 0.87 mmol) amine **9a**. Yield 0.207 g (58.2%).
White solid (mp = 102-103°C).
Purity (HPLC): 99.4%
^{1}H NMR (400 MHz, $CDCl_3$): δ (ppm) 7.55 (d, J = 8.2 Hz, 1H), 7.47 – 7.38 (m, 1H), 7.29 – 7.24 (m, 1H), 6.99 – 6.90 (m, 1H), 6.85 – 6.76 (m, 2H), 5.20 (s, 2H), 3.93 (s, 3H), 3.74 (s, 2H), 3.70 – 3.60 (m, 2H), 2.83 – 2.77 (m, 2H), 2.73 (t, J = 6.8 Hz, 4H), 1.88 (br s, 3H), 1.72 (p, J = 6.8 Hz, 2H).

^{13}C NMR (100 MHz, $CDCl_3$)): δ (ppm) 149.7, 146.6, 134.2, 133.9, 133.7, 132.8, 129.5, 129.1, 127.3, 120.3, 114.1, 112.1, 67.7, 60.8, 56.0, 53.8, 51.0, 47.8, 47.7, 30.0.
ESI-MS (m/z):413.2 $[M+H]^+$
HRMS (ESI) (m/z): Calculated for $C_{20}H_{27}Cl_2N_2O_3$ $[M+H]^+$ 413.1399, found 413.1413.

2-((3-((4-((3,5-Trifluoromethylbenzyl)oxy)-3-methoxybenzyl)amino)propyl)- amino)ethan-1-ol (***3s***).
Compound **3s** was prepared in a similar manner as described for compound **1a** from 0.363 g (0.96 mmol) of intermediate **8v** and 0.113 g (0.13 mL, 0.96 mmol) of amine **9a**. Yield: 0.334 g (72.5%).
Foam
Purity (HPLC): 98.9%
^1H NMR (DMSO-d_6, 400 MHz): δ (ppm) 8.14 (s, 2H), 8.05 (s, 1H), 7.00 (d, J=1.9 Hz, 1H), 6.98 (d, J=8.2 Hz, 1H), 6.81 (dd, J=8.2, 1.9 Hz, 1H), 5.26 (s, 2H), 3.77 (s, 3H), 3.60 (s, 2H), 3.43 (t, J=5.7 Hz, 2H), 2.66-2.44 (m, 6H), 1.55 (p, J=6.9 Hz, 2H)
^{13}C NMR (DMSO-d_6, 100 MHz): δ (ppm) 149.3, 145.8, 141.2, 136.1, 130.3 (q, J_{CF}=33.0 Hz), 128.1 (q, J_{CF}=3.1 Hz), 123.3 (q, J_{CF}= 272.6 Hz), 121.4 (m), 119.8, 114.4, 112.1, 69.0, 60.3, 55.5, 52.8, 51.8, 47.8, 47.1, 29.8
^{19}F NMR (DMSO-d_6, 376 MHz): δ (ppm) -61.43
ESI-MS (m/z): 481.3 $[M+H]^+$
HR-MS (ESI) (m/z): Calculated for $C_{22}H_{27}N_2O_3F_6$ $[M+H]^+$ 481.1926, found 481.1931

2-((3-((4-((5-chlorothiophen-2-yl)methoxy)-3-methoxybenzyl)amino)propyl)amino)ethan-1-ol (***3t***).
Compound **3t** was prepared in a similar manner as described for compound **1a** from 0.490 g (1.73 mmol) of intermediate **8w** and 0.205 g (0.20 mL, 1.73 mmol) amine **9a**. Yield 0.420 g (62.9 %).
Light brown oil.
Purity (HPLC): 96.1%
^1H NMR (400 MHz, $CDCl_3$): δ (ppm) 6.90 (d, J = 2.0 Hz, 1H), 6.86 (d, J = 8.1 Hz, 1H), 6.84 – 6.81 (m, 1H), 6.81 – 6.74 (m, 2H), 5.14 – 5.12 (m, 2H), 3.87 (s, 3H), 3.71 (s, 2H), 3.65 – 3.57 (m, 2H), 2.78 – 2.73 (m, 2H), 2.70 (td, J = 6.8, 3.0 Hz, 4H), 2.38 (br s, 3H), 1.70 (p, J = 6.8 Hz, 2H).
^{13}C NMR (100 MHz, $CDCl_3$)): δ (ppm) 150.2, 146.3, 138.6, 134.6, 130.6, 126.2, 125.7, 120.2, 115.4, 112.2, 66.7, 60.8, 55.9, 53.8, 51.1, 47.8, 47.8, 30.0.
ESI-MS (m/z): 385.2 $[M+H]^+$
HRMS (ESI) (m/z): Calculated for $C_{18}H_{26}ClN_2O_3S$ $[M+H]^+$ 385.1351, found 385.1353.

2-((3-((4-(Benzo[b]thiophen-2-ylmethoxy)-3-methoxybenzyl)amino)propyl)amino)ethan-1-ol (***3u***).
Compound **3u** was prepared in a similar manner as described for compound **1a** from 0.160 g (0.54 mmol) of intermediate **8x** and 0.063 g (0.06 mL, 0.54 mmol) amine **9a**. Yield 0.120 g (56.0%).
Solid (mp = 93-94°C).
Purity (HPLC): 94.2%
^1H NMR (400 MHz, $CDCl_3$): δ (ppm) 7.82 – 7.76 (m, 1H), 7.75 – 7.66 (m, 1H), 7.36 – 7.27 (m, 3H), 6.95 – 6.89 (m, 2H), 6.77 (dd, J = 8.2, 2.0 Hz, 1H), 5.36 (s, 2H), 3.90 (s, 3H), 3.70 (s, 2H), 3.65 – 3.58 (m, 2H), 2.77 – 2.72 (m, 2H), 2.69 (td, J = 6.8, 2.5 Hz, 4H), 2.41 (br s, 3H), 1.69 (p, J = 6.8 Hz, 2H).
^{13}C NMR (100 MHz, $CDCl_3$): δ (ppm) 150.0, 146.6, 140.8, 140.2, 139.4, 134.3, 124.4, 124.3, 123.6, 122.8, 122.4, 120.2, 114.9, 112.2, 67.2, 60.7, 56.1, 53.8, 51.1, 47.8, 47.7, 29.9.
ESI-MS (m/z):401.3 $[M+H]^+$
HRMS (ESI) (m/z): Calculated for $C_{22}H_{29}N_2O_3S$ $[M+H]^+$ 401.1905, found 401.1899.

2-((3-((4-(Isoquinolin-3-ylmethoxy)-3-methoxybenzyl)amino)propyl)amino)-ethan-1-ol (***3v***).
Compound **3v** was prepared in a similar manner as described for compound **1a** from 0.352 g (1.2 mmol) of intermediate **8y** and 0.154 g (0.16 mL, 1.3 mmol) of amine **9a**. Yield: 0.417 g (86.3%).
Viscous liquid
Purity (HPLC): 98.8%
^{1}H NMR (DMSO-d$_6$, 400 MHz): δ (ppm) 8.43-8.37 (m, 1H), 8.04-7.95 (m, 2H), 7.59-7.53 (m, 1H), 7.44-7.32 (m, 3H), 6.97 (d, *J*=1.9 Hz, 1H), 6.94 (d, *J*=8.1 Hz, 1H), 6.79 (dd, *J*=8.1, 1.9 Hz, 1H), 5.09 (s, 2H), 3.63 (s, 2H), 3.59 (s, 2H), 3.45 (t, *J*=5.7 Hz, 2H), 2.69-2.45 (m, 6H), 1.56 (p, *J*=6.9 Hz, 2H), 1.02 (s, 9H)
^{13}C NMR (DMSO-d$_6$, 100 MHz): δ (ppm) 158.0, 149.1, 147.0, 146.3, 137.0, 134.3, 129.9, 128.6, 128.0, 127.2, 126.6, 119.9, 119.6, 113.7, 112.2, 71.7, 60.0, 55.6, 52.8, 51.6, 47.6, 47.0, 29.3
ESI-MS (m/z): 396.3 $[M+H]^+$
HR-MS (ESI) (m/z): Calculated for $C_{23}H_{30}N_3O_3$ $[M+H]^+$ 396.2287, found 396.2298

2-((3-((3-Methoxy-4-((6-methylpyridin-2-yl)methoxy)benzyl)amino)propyl)- amino)ethan-1-ol (***3w***).
Compound **3w** was prepared in a similar manner as described for compound **1a** from 0.235 g (0.9 mmol) of intermediate **8z** and 0.118 g (0.12 mL, 1.0 mmol) of amine **9a**. The crude product was purified by silica gel column chromatography (CH_2Cl_2/MeOH/25%NH_4OH 100:10:0_150:20:10_100:20:10 v/v) followed by reverse phase flash column chromatography (BIOTAGE (R) SNAP Cartrige KP-C18-HS, linear gradient elution from 0% to 40% CH_3CN in 0.2% acetic acid in water) to obtain product **2z** as diacetate**.** Yield: 0.285 g (66.0%).
Viscous liquid
Purity (HPLC): 98.0%
^{1}H NMR (DMSO-d$_6$, 400 MHz): δ (ppm) 8.48 (d, *J*=2.2 Hz, 1H), 7.71 (dd, *J*=7.9, 2.4 Hz, 1H), 7.26 (d, *J*=7.9 Hz, 1H), 7.11-7.07 (m, 1H), 6.97 (d, *J*=8.2 Hz, 1H), 6.79 (dd, *J*=8.2, 1.9 Hz, 1H), 5.04 (s, 2H), 3.75 (s, 3H), 3.73 (s, 2H), 3.58 (t, *J*=5.2 Hz, 2H), 2.88-2.80 (m, 4H), 2.68-2.65 (m, 2H), 2.46 (s, 3H), 1.86-1.77 (m, 2H), 1.82 (s, 5H, CH_3COOH)
^{13}C NMR (DMSO-d$_6$, 100 MHz): δ (ppm) 173.6, 157.6, 149.2, 148.6, 146.8, 136.3, 130.9, 129.8, 123.0, 120.9, 113.8, 112.9, 67.8, 57.6, 55.6, 51.5, 49.9, 46.0, 45.5, 25.1, 23.8, 22.6
ESI-MS (m/z): 360.3 $[M+H]^+$
HR-MS (ESI) (m/z): Calculated for $C_{20}H_{30}N_3O_3$ $[M+H]^+$ 360.2287, found 360.2288

2-((3-((4-((3,5-Dichlorobenzyl)oxy)benzyl)amino)propyl)amino)ethan-1-ol (***4a***)**.**
Compound **4a** was prepared in a similar manner as described for compound **1a** from 0.400 g (1.42 mmol) of intermediate **14a** and 0.186 g (0.18 mL, 1.57 mmol) of amine **9a**. Yield: 0.250 g (46.0%).
Viscous liquid
Purity (HPLC): 98.2%
^{1}H NMR (DMSO-d$_6$, 400 MHz): δ (ppm) 7.53 (t, *J*=2.0 Hz, 1H), 7.48 (d, *J*=2.0 Hz, 2H), 7.29-7.18 (m, 2H), 6.99-6.88 (m, 2H), 5.10 (s, 2H), 3.59 (s, 2H), 3.44 (t, *J*=5.7 Hz, 2H), 2.65-2.42 (m, 6H), 1.55 (p, *J*=6.9 Hz, 2H)
^{13}C NMR (DMSO-d$_6$, 100 MHz): δ (ppm) 156.6, 141.7, 134.1, 133.5, 129.1, 127.3, 126.0, 114.4, 67.5, 60.1, 52.4, 51.6, 47.6, 47.0, 29.5
ESI-MS (m/z): 383.2 $[M+H]^+$
HR-MS (ESI) (m/z): Calculated for $C_{19}H_{25}N_2O_2Cl_2$ $[M+H]^+$ 383.1293, found 383.1299

Synthesis of compounds 4b to 4e

Aldehyde **17** (1.0 mmol) was dissolved in 5 mL of MeOH and N-(2-hydroxyethyl)-1,3-propanediamine (**9a**) (1.0 mmol), Na_2SO_4 (2.0 mmol) and glacial acetic acid (2.0 mmol). After stirring for 5 minutes at room temperature, $NaBH(OAc)_3$ (1.5 mmol) was added to the reaction mixture in portions over 5 minutes. The reaction mixture was stirred at room temp for 18h, then 4 N NaOH was added (pH 14 by pH paper) and the mixture was extracted with EtOAc (3 x 25 mL). The organic layers were combined and washed with brine, dried over Na_2SO_4. The filtrate was concentrated under reduced pressure and the residue was purified by column chromatography on silica gel (eluent MeOH/NH_4OH (15:1) to (10:1)) to give desired products **5**.
The free bases **5** if needed can be converted to water soluble acetate salts by reverse phase flash column chromatography (BIOTAGE (R) SNAP Cartrige KP-C18-HS, linear gradient elution from 0% to 40% CH_3CN in 0.2% acetic acid in water) to obtain products **5** as diacetate salts**.**

2-((3-((4-((3,5-Bis(trifluoromethyl)benzyl)oxy)-3-((4-isopropylbenzyl)oxy)benzyl)amino)propyl)amino)ethan-1-ol (***4b***).
Compound 4b was prepared from 0.210 g (0.42 mmol) of intermediate **17a** and 0.050 g (0.05 mL, 0.42 mmol) amine **9a**. Yield 0.165 g (65.0%).
Solid (mp = 62-64°C).
Purity (HPLC): 99.6%
^{1}H NMR (400 MHz, $CDCl_3$): δ (ppm) 7.92 (s, 2H), 7.81 (s, 1H), 7.40 – 7.34 (m, 2H), 7.25 – 7.20 (m, 2H), 7.03 (d, *J* = 1.9 Hz, 1H), 6.91 (d, *J* = 8.1 Hz, 1H), 6.85 (dd, *J* = 8.1, 1.9 Hz, 1H), 5.18 (s, 2H), 5.10 (s, 2H), 3.73 (s, 2H), 3.68 – 3.59 (m, 2H), 2.91 (hept, *J* = 6.9 Hz, 1H), 2.81 – 2.74 (m, 2H), 2.74 – 2.66 (m, 4H), 2.00 (br s, 3H), 1.70 (p, *J* = 6.8 Hz, 2H), 1.26 (s, 3H), 1.24 (s, 3H).
^{13}C NMR (100 MHz, $CDCl_3$): δ (ppm) 149.5, 148.8, 146.8, 140.3, 135.1, 134.2, 131.7 (q, J_{CF} = 33.3 Hz), 127.5, 127.2 (m), 126.6, 123.3 (q, J_{CF} = 272.8 Hz), 121.6 (p, J_{CF} = 3.8 Hz), 120.8, 115.7, 114.3, 70.9, 70.3, 60.9, 53.8, 51.0, 47.8, 47.7, 33.9, 30.2, 24.0.
^{19}F NMR (376 MHz, $CDCl_3$): δ (ppm) -62.83.
ESI-MS (m/z): 599.4 $[M+H]^+$
HRMS (ESI) (m/z): Calculated for $C_{31}H_{37}F_6N_2O_3$ $[M+H]^+$ 599.2708, found 599.2703.

2-((3-((3,4-bis((3,5-Bis(trifluoromethyl)benzyl)oxy)benzyl)amino)propyl)amino)ethan-1-ol (***4c***).
Compound **4c** was prepared from 0.280 g (0.47 mmol) of intermediate **16** and 0.056 g (0.06 mL, 0.47 mmol) amine **9a**. Yield 0.220 g (67.1%).
Solid (mp = 105-107°C).
Purity (HPLC): 99.5%
^{1}H NMR (400 MHz, $CDCl_3$): δ (ppm) 7.90 (s, 2H), 7.88 (s, 2H), 7.82 (s, 2H), 7.05 (s, 1H), 6.93 (s, 2H), 5.23 (s, 2H), 5.20 (s, 2H), 3.73 (s, 2H), 3.66 – 3.61 (m, 2H), 2.80 – 2.74 (m, 2H), 2.71 (td, *J* = 6.8, 5.3 Hz, 4H), 2.04 (br s, 3H), 1.70 (p, *J* = 6.8 Hz, 2H).
^{13}C NMR (100 MHz, $CDCl_3$): δ (ppm) 148.5, 137.3, 139.7, 139.6, 135.1, 131.9 (q, J_{CF} = 33.5 Hz), 131.8 (q, J_{CF} = 33.5 Hz), 127.2 (m), 124.5, 121.9 (m), 119.1, 115.2, 115.1, 70.2, 70.0, 60.9, 53.6, 47.8, 47.8, 30.1.
^{19}F NMR (376 MHz, $CDCl_3$): δ (ppm) -63.05.
ESI-MS (m/z): 693.2 $[M+H]^+$
HRMS (ESI) (m/z): Calculated for $C_{30}H_{29}F_{12}N_2O_3$ $[M+H]^+$ 693.1987, found 693.1980.

2-((3-((4-((3,5-Bis(trifluoromethyl)benzyl)oxy)-3-((2,3-dichlorobenzyl)oxy)benzyl)amino)propyl)amino)ethan-1-ol (***4d***).
Compound **4d** was prepared from 0.210 g (0.40 mmol) of intermediate **17c** and 0.047 g (0.05 mL, 0.40 mmol) amine **9a**. Yield 0.152 g (61.0%).
White solid (mp = 115-117°C).
Purity (HPLC): 97.3%
^{1}H NMR (400 MHz, $CDCl_3$): δ (ppm) 7.94 (s, 2H), 7.83 (s, 1H), 7.59-7.53 (m, 1H), 7.43 (dd, J = 8.1, 1.6 Hz, 1H), 7.22 (t, J = 7.9 Hz, 1H), 7.02 (d, J = 1.9 Hz, 1H), 6.93 (d, J = 8.1 Hz, 1H), 6.90 (dd, J = 8.1, 1.9 Hz, 1H), 5.22 (s, 2H), 5.21 (s, 2H), 3.74 (s, 2H), 3.67 – 3.59 (m, 2H), 2.80 – 2.74 (m, 2H), 2.70 (td, J = 6.8, 5.3 Hz, 4H), 2.30 (br s, 3H), 1.70 (p, J = 6.8 Hz, 2H).
^{13}C NMR (100 MHz, $CDCl_3$): δ (ppm) 148.6, 146.9, 140.1, 137.1, 135.0, 133.0, 131.8 (q, J_{CF} = 33.3 Hz), 130.3, 129.6, 127.5, 127.1 (m), 126.4, 123.3 (q, J_{CF} = 272.6 Hz), 121.7 (p, J_{CF} = 3.8 Hz), 121.3, 115.0, 114.2, 70.0, 68.2, 60.8, 53.7, 51.0, 47.8, 47.7, 30.1.
^{19}F NMR (376 MHz, $CDCl_3$): δ (ppm) -62.84.
ESI-MS (m/z): 625.2 $[M+H]^+$
HRMS (ESI) (m/z): Calculated for $C_{28}H_{29}Cl_2F_6N_2O_3$ $[M+H]^+$ 625.1459, found 625.1497.

2-((3-((4-((3,5-Bis(trifluoromethyl)benzyl)oxy)-3-((3,4-dichlorobenzyl)oxy)benzyl)amino)propyl)amino)ethan-1-ol (***4e***).
Compound **4e** was prepared from 0.300 g (0.57 mmol) of intermediate **17d** and 0.068 g (0.07 mL, 0.57 mmol) amine **9a**. Yield 0.215 g (60.1%).
Solid (mp = 111-113°C).
Purity (HPLC): 98.9%
^{1}H NMR (400 MHz, $CDCl_3$): δ (ppm) 7.91 (s, 2H), 7.83 (s, 1H), 7.51 (d, J = 2.0 Hz, 1H), 7.42 (d, J = 8.2 Hz, 1H), 7.29 (dd, J = 8.2, 2.0 Hz, 1H), 6.98 (d, J = 1.9 Hz, 1H), 6.91 (d, J = 8.2 Hz, 1H), 6.87 (dd, J = 8.2, 1.9 Hz, 1H), 5.19 (s, 2H), 5.09 (s, 2H), 3.71 (s, 2H), 3.68 – 3.59 (m, 2H), 2.80 – 2.73 (m, 2H), 2.71 (t, J = 6.8 Hz, 2H), 2.67 (t, J = 6.8 Hz, 2H), 2.29 (br s, 3H), 1.69 (p, J = 6.8 Hz, 2H).
^{13}C NMR (100 MHz, $CDCl_3$): δ (ppm) 148.7, 147.1, 140.0, 137.2, 135.0, 132.7, 131.9, 131.8 (q, J_{CF} = 33.4 Hz), 130.6, 129.1, 127.1 (m), 126.4, 123.3 (q, J_{CF} = 272.6 Hz), 121.8 (p, J_{CF} = 3.8 Hz), 121.5, 115.3, 114.6, 70.1, 69.7, 60.8, 53.6, 51.0, 47.8, 47.7, 30.1.
^{19}F NMR (376 MHz, $CDCl_3$): δ (ppm) -62.86.
ESI-MS (m/z): 625.2 $[M+H]^+$
HRMS (ESI) (m/z): Calculated for $C_{28}H_{29}Cl_2F_6N_2O_3$ $[M+H]^+$ 625.1459, found 625.1469.

General Synthetic Procedure for Compounds 8a-8z
4-((3-Chlorobenzyl)oxy)-3-methoxybenzaldehyde (***8a***).
To a suspension of vanilin (**6**) (1.25 g, 8.22 mmol) and K_2CO_3 (2.27 g, 16.4 mmol) in dry DMF (10 mL), 1-(bromomethyl)-3-chlorobenzene (**7a**) (1.77 g, 1.14 mL, 8.63 mmol) was added dropwise. The resulting mixture was stirred at room temperature overnight and poured into a solution of EtOAc/H_2O (50 mL/50 mL). The organic layer was separated and the aqueous layer was extracted with an additional portion of EtOAc (50 mL). The combined organic extracts were washed with water (5 x 20 mL), brine (25 mL), dried (Na_2SO_4), filtrated and concentrated in vacuum. The oily residue was treated with hexane (50 mL). The resulted preciptate was collected by filtration and dried to obtain compound **8a** as a white solid (1.88 g, 82.8%).
^{1}H NMR (DMSO-d_6, 400 MHz): δ (ppm) 9.85 (s, 1H), 7.62-7.50 (m, 2H), 7.45-7.41 (m, 4H), 7.26 (d, J=8.2 Hz, 1H), 5.24 (s, 2H), 3.85 (s, 3H)

4-(Benzyl)oxy)-3-methoxybenzaldehyde (***8b***).
Compound **8b** was prepared from vanilin (**6**) and bromide **7b** in a similar manner as described for compound **8a**.
Spectral data of intermediate **3** were identical to those previously reported (Zhang et al., 2016).
4-((2-Chlorobenzyl)oxy)-3-methoxybenzaldehyde (***8c***).
Compound **8c** was prepared from vanilin (**6**) and bromide **7c** in a similar manner as described for compound **8a**. Yield: 80.9%
^{1}H NMR (DMSO-d_6, 400 MHz): δ (ppm) 9.86 (s, 1H), 7.63-7.51 (m, 3H), 7.46-7.39 (m, 3H), 7.30 (d, *J*=8.3 Hz, 1H), 5.26 (s, 2H), 3.84 (s, 3H)
4-((4-Chlorobenzyl)oxy)-3-methoxybenzaldehyde (***8d***).
Compound **8d** was prepared from vanilin (**6**) and bromide **7d** in a similar manner as described for compound **8a**. Yield: 94.7%
Spectral data of intermediate **8d** were identical to those previously reported (Carrasco-Gomez et al., 2014).
4-((3-Fluorobenzyl)oxy)-3-methoxybenzaldehyde (***8e***).
Compound **8e** was prepared from vanilin (**6**) and bromide **7e** in a similar manner as described for compound **8a**. Yield: quantitative
^{1}H-NMR (400 MHz, DMSO-d$_6$): δ (ppm) 9.84 (s, 1H), 7.56 – 7.52 (m, 1H), 7.49 – 7.42 (m, 2H), 7.32 – 7.23 (m, 3H), 7.21 – 7.15 (m, 1H), 5.24 (s, 2H), 3.85 (s, 3H).
4-((4-Bromobenzyl)oxy)-3-methoxybenzaldehyde (***8f***).
Compound **8f** was prepared from vanilin (**6**) and bromide **7f** in a similar manner as described for compound **8a.** Yield: quantitative
^{1}H NMR (DMSO-d_6, 400 MHz): δ (ppm) 9.84 (s, 1H), 7.65-7.57 (m, 2H), 7.54 (dd, *J*=8.2, 1.9 Hz, 1H), 7.45-7.40 (m, 3H), 7.25 (d, *J*=8.2 Hz, 1H), 5.20 (s, 2H), 3.84 (s, 3H)
4-((3-Bromobenzyl)oxy)-3-methoxybenzaldehyde (***8g***).
Compound **8g** was prepared from vanilin (**6**) and bromide **7g** in a similar manner as described for compound **8a.** Yield: 81.2%
^{1}H-NMR (400 MHz, DMSO-d$_6$): δ (ppm) 9.84 (s, 1H), 7.70 – 7.65 (m, 1H), 7.58 – 7.52 (m, 2H), 7.49 – 7.41 (m, 2H), 7.41 – 7.34 (m, 1H), 7.28 – 7.22 (m, 1H), 5.22 (s, 2H), 3.85 (s, 3H)
4-((4-Iodobenzyl)oxy)-3-methoxybenzaldehyde (***8h***).
Compound **8h** was prepared from vanilin (**6**) and bromide **7h** in a similar manner as described for compound **8a.**Yield: quantitative
^{1}H NMR (DMSO-d_6, 400 MHz): δ (ppm) 9.84 (s, 1H), 7.79-7.74 (m, 2H), 7.56-7.50 (m, 1H), 7.43-7.40 (m, 1H), 7.32-7.18 (m, 3H), 5.18 (s, 2H), 3.84 (s, 3H)
3-((4-Formyl-2-methoxyphenoxy)methyl)benzonitrile (***8i***).
Compound **8i** was prepared from vanilin (**6**) and bromide **7i** in a similar manner as described for compound **8a.** Yield: 98.9%
^{1}H-NMR (400 MHz, DMSO-d$_6$): δ (ppm) 9.85 (s, 1H), 7.94 – 7.92 (m, 1H), 7.86 – 7.79 (m, 2H), 7.64 (t, *J* = 7.6 Hz, 1H), 7.55 (dd, *J* = 8.2, 1.9 Hz, 1H), 7.44 (d, *J* = 1.9 Hz, 1H), 7.27 (d, *J* = 8.2 Hz, 1H), 5.28 (s, 2H), 3.85 (s, 3H).
3-Methoxy-4-((3-methoxylbenzyl)oxy)benzaldehyde (***8j***).
Compound **8j** was prepared from vanilin (**6**) and bromide **7j** in a similar manner as described for compound **8a.** Yield: 92.1%
^{1}H-PMR (400 MHz, DMSO-d$_6$): δ (ppm) 9.84 (s, 1H), 7.53 (dd, *J*=8.3, 1.9 Hz, 1H), 7.42 (d, *J*=1.9 Hz, 1H), 7.34-7.29 (m, 1H), 7.25 (d, *J*=8.3 Hz, 1H), 7.06-7.00 (m, 2H), 6.94-6.89 (m, 1H), 5.19 (s, 2H), 3.84 (s, 3H), 3.76 (s, 3H)
3-Methoxy-4-((3-methylbenzyl)oxy)benzaldehyde (***8k***).

Compound **8k** was prepared from vanilin (**6**) and bromide **7k** in a similar manner as described for compound **8a.** Yield: quantitative

Spectral data of intermediate **8k** were identical to those previously reported (Zhang et al., 2017).

3-Methoxy-4-((3-(trifluoromethyl)benzyl)oxy)benzaldehyde (***8l***).

Compound **8l** was prepared from vanilin (**6**) and bromide **7l** in a similar manner as described for compound **8a.** Yield**:**
95.8%

^{1}H NMR (DMSO-d_6, 400 MHz): δ (ppm) 9.88 (s, 1H), 7.63-7.57 (m, 3H), 7.53-7.46 (m, 1H), 7.45-7.41 (m, 2H), 5.33 (s, 2H), 3.80 (s, 3H)

4-((4-(tert-Butyl)benzyl)oxy)-3-methoxybenzaldehyde (***8n***).

Compound **8n** was prepared from vanilin (**6**) and bromide **7n** in a similar manner as described for compound **8a.** Yield: quantitative

^{1}H NMR ($CDCl_3$, 400 MHz): δ (ppm) 9.84 (s, 1H), 7.48-7.34 (m, 6H), 7.01 (d, *J*=8.2 Hz, 1H), 5.21 (s, 2H), 3.94 (s, 3H), 1.32 (s, 9H)

Procedure for benzylation using biphenylmethanol (7o)

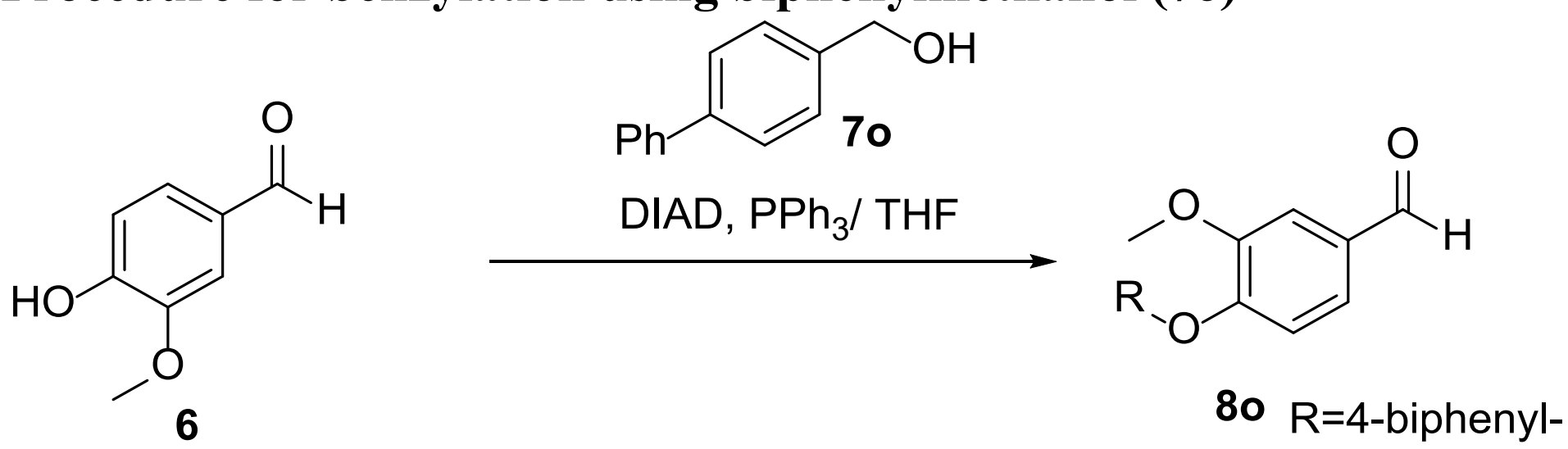

4-([1,1'-Biphenyl]-4-ylmethoxy)-3-methoxybenzaldehyde (***8o***).

A solution of vanillin (**6**) (0.330 g, 2.17 mmol), biphenylmethanol (**7o**) (0.400 g, 2.17 mmol)and PPh_3 (1.5 mmol) was stirred in dry THF (13 mL) at 0 °C under a nitrogen atmosphere. To this mixture was added dropwise DIAD (1.5 mmol) over a period of 5 min. After stirred for 30 min, the reaction mixture was warmed to room temperature and stirred overnight. The mixture was evaporated under reduced pessure and the residue was purified by column chromatography on silica gel eluting with a mixture of petroleum ether/EtOAc to afford the desired product **8o**. Yield 0.230 g (33%), white solid. ^{1}H NMR (400 MHz, $CDCl_3$): δ (ppm) 9.86 (s, 1H), 7.72 – 7.30 (m, 11H), 7.03 (d, *J* = 8.1 Hz, 1H), 5.29 (s, 2H), 3.97 (s, 3H); ESI-MS (m/z): 319.2 $[M+H]^+$.

4-((2,6-Dichlorobenzyl)oxy)-3-methoxybenzaldehyde (***8p***).

Compound **8p** was prepared from vanilin (**6**) and bromide **7p** in a similar manner as described for compound **8a.** Yield: 95.5%

^{1}H NMR (DMSO-d_6, 400 MHz): δ (ppm) 9.88 (s, 1H), 7.63-7.57 (m, 3H), 7.53-7.46 (m, 1H), 7.45-7.41 (m, 2H), 5.33 (s, 2H), 3.80 (s, 3H)

4-((3,4-dichlorobenzyl)oxy)-3-methoxybenzaldehyde (***8q***).

Compound **8q** was prepared from vanilin (**6**) and bromide **7q** in a similar manner as described for compound **8a.** Yield: 86%

^{1}H NMR (400 MHz, $CDCl_3$): δ (ppm) 9.86 (s, 1H), 7.57 – 7.53 (m, 1H), 7.49 – 7.43 (m, 2H), 7.41 (dd, *J* = 8.2, 2.0 Hz, 1H), 7.28 (ddt, *J* = 8.2, 2.0, 0.7 Hz, 1H), 6.95 (d, *J* = 8.2 Hz, 1H), 5.17 (s, 2H), 3.96 (s, 3H); ESI-MS (m/z): 311.0 $[M+H]^+$.

4-((4-Chloro-3-(trifluoromethyl)benzyl)oxy)-3-methoxybenzaldehyde (***8r***).

Compound **8r** was prepared from vanilin (**6**) and bromide **7r** in a similar manner as described for compound **8a.** Yield: quantitative.

^{1}H NMR ($CDCl_3$, 400 MHz): δ 9.87 (s, 1H), 7.79 (dt, *J* = 2.0, 0.7 Hz, 1H), 7.60 – 7.56 (m, 1H), 7.53 (d, *J* = 8.3 Hz, 1H), 7.45 (d, *J* = 1.9 Hz, 1H), 7.42 (dd, *J* = 8.1, 1.9 Hz, 1H), 6.98 (d, *J* = 8.1 Hz, 1H), 5.22 (s, 2H), 3.96 (s, 3H).

4-((2,3-Dichlorobenzyl)oxy)-3-methoxybenzaldehyde (***8s***).

Compound **8s** was prepared from vanilin (**6**) and bromide **7s** in a similar manner as described for compound **8a.** Yield: quantitative

^{1}H NMR ($CDCl_3$, 400 MHz): δ (ppm) 9.86 (s, 1H), 7.51-7.44 (m, 3H), 7.42 (dd, *J*=8.2, 1.9 Hz, 1H), 7.26-7.21 (m, 1H), 6.96 (d, *J*=8.2 Hz, 1H), 5.33 (s, 2H), 3.98 (s, 3H)

4-((2,4-Dichlorobenzyl)oxy)-3-methoxybenzaldehyde (***8t***).

Compound **8t** was prepared from vanilin (**6**) and bromide **7t** in a similar manner as described for compound **8a.** Yield: 96 %

^{1}H NMR (400 MHz, $CDCl_3$): δ (ppm) 9.86 (s, 1H), 7.50 (d, *J* = 8.3 Hz, 1H), 7.47 – 7.39 (m, 3H), 7.28 (dd, *J* = 8.3, 2.1 Hz, 1H), 6.96 (d, *J* = 8.2 Hz, 1H), 5.28 (s, 2H), 3.97 (s, 3H); ESI-MS (m/z): 311.4 $[M+H]^+$.

4-((3,5-Dichlorobenzyl)oxy)-3-methoxybenzaldehyde (***8u***).

Compound **8u** was prepared from vanilin (**6**) and bromide **7u** in a similar manner as described for compound **8a.** Yield: 94.4%

^{1}H NMR ($CDCl_3$, 400 MHz): δ (ppm) 9.86 (s, 1H), 7.45 (d, *J*=1.9 Hz, 1H), 7.42 (dd, *J*=8.2, 1.9 Hz, 1H), 7.35-7.32 (m, 3H), 6.96 (d, *J*=8.2 Hz, 1H), 5.16 (s, 2H), 3.97 (s, 3H)

4-((3,5-Trifluoromethylbenzyl)oxy)-3-methoxybenzaldehyde (***8v***).

Compound **8v** was prepared from vanilin (**6**) and bromide **7v** in a similar manner as described for compound **8a.** Yield: quantitative

^{1}H NMR ($CDCl_3$, 400 MHz): δ (ppm) 9.88 (s, 1H), 7.97-7.92 (m, 2H), 7.89-7.85 (m, 1H), 7.47 (d, *J*=1.9 Hz, 1H), 7.45 (dd, *J*=8.1, 1.9 Hz, 1H), 7.01 (d, *J*=8.1 Hz, 1H), 5.31 (s, 2H), 3.97 (s, 3H)

4-((5-Chlorothiophen-2-yl)methoxy)-3-methoxybenzaldehyde (***8w***).

Compound **8w** was prepared from (0.300 g, 1.97 mmol) of vanillin **6** and 0.329 g (1.97 mmol) 2-chloro-5-(chloromethyl)thiophene (**7w**). Yield 0.493 g (88%), beige solid. ^{1}H NMR (400 MHz, $CDCl_3$): δ (ppm) 9.86 (s, 1H), 7.45 – 7.39 (m, 2H), 7.07 – 7.01 (m, 1H), 6.91 (dt, *J* = 3.8, 0.9 Hz, 1H), 6.81 (d, *J* = 3.8 Hz, 1H), 5.27 (s, 2H), 3.94 (s, 3H); ESI-MS (m/z): 283.1 $[M+H]^+$.

4-(Benzo[b]thiophen-2-ylmethoxy)-3-methoxybenzaldehyde (***8x***).

Compound **8x** was prepared in a similar manner as described for compound **8o** using vanillin **6** (0.139 g, 0.91 mmol) and benzothiophene-2-methanol **7x** (0.150 g, 0.91 mmol). Yield 0.100 g (37%), white solid. ^{1}H NMR (400 MHz, $CDCl_3$): δ (ppm) 9.85 (s, 1H), 7.84 – 7.71 (m, 2H), 7.45 (d, *J* = 1.9 Hz, 1H), 7.41 (dd, *J* = 8.2, 1.9 Hz, 1H), 7.39 – 7.30 (m, 3H), 7.10 (d, *J* = 8.2 Hz, 1H), 5.50 (d, *J* = 1.1 Hz, 2H), 3.96 (s, 3H); ESI-MS (m/z): 299.1 $[M+H]^+$.

4-*((Isoquinolin-3-yl)methoxy)-3-methoxybenzaldehyde* (***8y***).

Compound **8y** was prepared from vanilin (**6**) and bromide **7y** in a similar manner as described for compound **8a.**

The crude product was purified by silica gel column chromatography (PET/EtOAc 10:1_5:1_3:1 v/v).Yield: 81.1%

^{1}H NMR ($CDCl_3$, 400 MHz): δ (ppm) 9.83 (s, 1H), 8.20 (d, *J*=8.5 Hz, 1H), 8.09 (d, *J*=8.5 Hz, 1H), 7.85-7.81 (m, 1H), 7.76 (ddd, *J*=8.2, 6.9, 1.2 Hz, 1H), 7.67 (d, *J*=8.5 Hz, 1H), 7.57 (ddd, *J*=8.2, 6.9, 1.2 Hz, 1H), 7.46 (d, *J*=1.9 Hz, 1H), 7.36 (dd, *J*=8.2, 1.9 Hz, 1H), 7.06 (d, *J*=8.2 Hz, 1H), 5.55 (s, 2H0, 4.00 (s, 3H)

3-Methoxy-4-((6-methylpyridin-2-yl)methoxy)benzaldehyde (***8z***).

Compound **8z** was prepared from vanilin (**6**) and bromide **7z** in a similar manner as described for compound **8a.** Yield: 76.9%

^{1}H NMR ($CDCl_3$, 400 MHz): δ (ppm) 9.85 (s, 1H), 8.60-8.55 (m, 1H), 7.69 (dd, *J*=8.0, 2.4 Hz, 1H), 7.45-7.39 (m, 2H), 7.19 (d, *J*=8.0 Hz, 1H), 7.01 (d, *J*=8.0 Hz, 1H), 5.20 (s, 2H), 3.93 (s, 3H), 2.57 (s, 3H)

Synthesis of compounds 11a, 12a to 12f.

4-((3-Chlorobenzyl)oxy)-3-hydroxybenzaldehyde (***11a***).

To a suspension of 3,4-dihydroxy benzaldehyde (**10**) (1.15 g, 8.33 mmol), K_2CO_3 (1.15 g, 8.33 mmol) and KI (0.05g, 0.3 mmol) in dry DMF (10 mL), 1-(bromomethyl)-3-chlorobenzene (**7a**) (1.88 g, 1.2 mL, 9.15 mmol) was added. The reaction mixture was heated at 60 °C for 16 h, cooled to room temperature and diluted with water (50 mL). The aqueous solution was extrated with EtOAc.(3 x 50 mL). The combined organic extract was washed with H_2O (5 x 15 mL) and brine (20 mL), dried (Na_2SO_4), filtered and concentrated in vacuum.

The crude product was purified by silica gel column chromatography (CH_2Cl_2) to obtain compound **23a** as a tan solid (1.130 g, 53.3%).

Spectral data of intermediate **11a** were identical to those previously reported (Plourde and Spaetzel, 2002).

4-((3-Chlorobenzyl)oxy)-3-ethoxybenzaldehyde (***12a***).

To a suspension of intermediate **11a** (0.39g, 1.48 mmol) and K_2CO_3 (0.409 g, 2.96 mmol) in dry DMF (5 mL), ethyl iodide (0.463 g, 0.24 mL, 2.97 mmol) was added. The reaction mixture was heated in a closed vial at 65 °C for 24 h, cooled to room temperature and diluted with water (30 mL). The aqueous solution was extrated with EtOAc.(3 x 25 mL). The combined organic extract was washed with H_2O (5 x 10 mL) and brine (20 mL), dried (Na_2SO_4), filtered and concentrated in vacuum.

The crude product was purified by silica gel column chromatography (PET/EtOAc 10:1_10:2 v/v) to obtain compound **12a** (0.363 g, 84.4%).

^{1}H NMR ($CDCl_3$, 400 MHz): δ (ppm) 9.84 (s, 1H), 7.47-7.45 (m, 1H), 7.43 (d, *J*=1.9 Hz, 1H), 7.44 (dd, *J*=8.1, 1.9 Hz, 1H), 7.33-7.29 (m, 3H), 6.97 (d, *J*=8.1 Hz, 1H), 5.21 (s, 2H), 4.19 (q, *J*=7.0 Hz, 2H), 1.50 (t, *J*=7.0 Hz, 3H)

4-((3-Chlorobenzyl)oxy)-3-(isopenthyloxy)benzaldehyde (***12b***).

Compound **12b** was prepared in a similar manner as described for compound **12a.** Yield: 71.2%

^{1}H NMR ($CDCl_3$, 400 MHz): δ (ppm) 9.80 (s, 1H), 7.44-7.42 (m, 1H), 7.40 (d, *J*=1.9 Hz, 1H), 7.35 (dd, *J*=8.2, 1.9 Hz, 1H), 7.28-7.24 (m, 3H), 6.93 (d, *J*=8.2 Hz, 1H), 5.14 (s, 2H), 4.08 (t, *J*=6.7 Hz, 2H), 1.85 (hept, *J*=6.6 Hz, 1H), 1.73 (q, *J*=6.6 Hz, 2H), 0,95 (d, *J*=6.6 Hz, 6H)

4-((3-Chlorobenzyl)oxy)-3-(cyclopentyl)methoxy)benzaldehyde (***12c***).

Compound **12c** was prepared in a similar manner as described for compound **12a** using (iodomethyl)cyclopentane as an alkylating agent. Yield: 72.3%.

^{1}H NMR ($CDCl_3$, 400 MHz): δ (ppm) 9.84 (s, 1H), 7.50-7.48 (m, 1H), 7.43 (d, *J*=1.9 Hz, 1H), 7.40 (dd, *J*=8.1, 1.9 Hz, 1H), 7.34-7.28 (m, 3H), 6.98 (d, *J*=8.1 Hz, 1H), 5.19 (s, 2H), 3.97 (d, J=6.9 Hz, 1H), 2.46 (hept, *J*=7.4 Hz, 1H), 1.94-1.82 (m, 2H), 1.73-1.57 (m, 4H), 1.48-1.37 (m, 2H)

4-((3-Chlorobenzyl)oxy)-3-(neopenthyloxy)benzaldehyde (***12d***).

Compound **12d** was prepared in a similar manner as described for compound **12a**. Yield: 75.0%

^{1}H NMR ($CDCl_3$, 400 MHz): δ (ppm) 9.85 (s, 1H), 7.54-7.51 (m, 1H), 7.43-7.39 (m, 2H), 7.35-7.28 (m, 3H), 7.00 (d, *J*=8.1 Hz, 1H), 5.17 (s, 2H), 3.75 (s, 2H), 1.09 (s, 9H)

4-((3-Chlorobenzyl)oxy)-3-(cyclohexyl)methoxy)benzaldehyde (***12e***).

Compound **12e** was prepared in a similar manner as described for compound **12a**. Yield: 77.4%

^{1}H NMR ($CDCl_3$, 400 MHz): δ (ppm) 9.84 (s, 1H), 7.52-7.48 (m, 1H), 7.42 (d, *J*=1.9 Hz, 1H), 7.39 (dd, *J*=8.1, 1.9 Hz, 1H), 7.32-7.28 (m, 3H), 6.98 (d, *J*=8.1 Hz, 1H), 5.18 (s, 2H), 3.89 (d, J=6.0 Hz, 1H), 1.97-1.66 (m, 6H), 1.41-1.04 (m, 5H)

3-(Benzyloxy)-4-((3-chlorobenzyl)oxy)benzaldehyde (***12f***).
Compound **12f** was prepared in a similar manner as described for compound **12a**. The crude product was purified by silica gel column chromatography (CH_2Cl_2). Yield: 87.6%
^{1}H-NMR ($CDCl_3$,400 MHz): δ (ppm) 9.83 (s, 1H), 7.53 – 7.47 (m, 4H), 7.45 – 7.38 (m, 3H), 7.35 – 7.30 (m, 4H), 7.00 (d, *J* = 8.2 Hz, 1H), 5.22 (s, 2H), 5.21 (s, 2H)

Synthesis of compounds 14a and 14b
4-*((3,5-Dichlorobenzyl)oxy)benzaldehyde* (***14a***).
Compound **14a** was prepared in a similar manner as described for compound **8a.** Yield: 91.7%
^{1}H NMR ($CDCl_3$, 400 MHz): δ (ppm) 9.91 (s, 1H), 7.90-7.81 (m, 2H), 7.37-7.30 (m, 3H), 7.11-7.01 (m, 2H), 5.10 (s, 2H)

4-((3,5-Bis(trifluoromethyl)benzyl)oxy)benzaldehyde (***14b***).
Compound **14b** was prepared in a similar manner as described for compound **8a.** Yield: 94.8%.
^{1}H NMR ($CDCl_3$, 400 MHz): δ (ppm) 9.92 (s, 1H), 7.90-7.85 (m, 5H), 7.17-7.05 (m, 2H), 5.25 (s, 2H)

Synthesis of compounds 15 and 16
4-((3,5-Bis(trifluoromethyl)benzyl)oxy)-3-hydroxybenzaldehyde (***15***) *and 3,4-bis((3,5-bis(trifluoromethyl)benzyl)oxy)benzaldehyde* (***16***).
The 3,4-dihydroxybenzaldehyde (400 mg, 2.90 mmol) and bromide (**7v**) (890 mg, 2.90 mmol) was dissolved in 6 mL of DMF and K_2CO_3 (800 mg, 5.80 mmol) was added to the mixture. The mixture was stirred at 65°C for 18 hours. The solution was cooled to room temperature, diluted with water and extracted with EtOAc (3x). The organic layers were combinied and washed with brine and dried over Na_2SO_4. The solvent was evaporated under reduced pressure and the residue was purified by silica gel column chromatography, eluting with PE/EA (5:1), to afford bis substituted product **16** (285 mg, yield 16%) as a beige solid ^{1}H NMR (400 MHz, $CDCl_3$): δ (ppm) 9.90 (s, 1H), 7.92 – 7.89 (m, 4H), 7.86 (d, *J* = 4.6 Hz, 2H), 7.57 – 7.53 (m, 2H), 7.12 – 7.07 (m, 1H), 5.31 (s, 2H), 5.28 (s, 2H); ESI-MS (m/z): 591.2 $[M+H]^+$ and mono substituted product **15** (480 mg, yield 45%) as a beige solid ^{1}H NMR (400 MHz, $CDCl_3$): δ (ppm) 9.87 (s, 1H), 7.95 – 7.88 (m, 3H), 7.51 (d, *J* = 2.0 Hz, 1H), 7.45 (dd, *J* = 8.3, 2.0 Hz, 1H), 7.02 (d, *J* = 8.3 Hz, 1H), 5.66 (s, 1H), 5.31 (s, 2H); ESI-MS (m/z): 365.2 $[M+H]^+$.

General procedure for synthesis of compounds 17. The 3-hydroxybenzaldehyde **15** (1 mmol) and corresponding bromide **7** (1 mmol) was dissolved in 4 mL of DMF, then K_2CO_3 (2 mmol) and KI (0.3 mmol) were added to the mixture. The mixture was stirred at 80 °C for 20 hours. The solution was cooled to room temperature, diluted with water and extracted with EtOAc (2x). The organic layers were combinied and washed with brine and dried over Na_2SO_4. The solvent was evaporated under reduced pressure and the residue was purified by column chromatography on silica gel, eluting with mixture of petroleum ether and ethyl acetate to afford products **17** in 67-100% yield.
4-((3,5-Bis(trifluoromethyl)benzyl)oxy)-3-((4-isopropylbenzyl)oxy)benzaldehyde (***17a***)**.**
Compound **17a** was prepared from 0.225 g (0.62 mmol) of intermediate **15** and 0.132 g (0.62 mmol) of 1-(bromomethyl)-4-isopropylbenzene (**7za**). Yield 0.205 g (67%), white solid; ^{1}H NMR (400 MHz, $CDCl_3$): δ (ppm) 9.88 (s, 1H), 7.95 (s, 2H), 7.85 (s, 1H), 7.56 (d, *J* = 1.9 Hz, 1H), 7.48 (dd, *J* = 8.2, 1.9 Hz, 1H), 7.42 – 7.37 (m, 2H), 7.28 – 7.23 (m, 2H), 7.05 (d, *J* = 8.2 Hz, 1H), 5.30 (s, 2H), 5.17 (s, 2H), 2.93 (p, *J* = 7.0 Hz, 1H), 1.27 (s, 3H), 1.25 (s, 3H); ESI-MS (m/z): 497.4 $[M+H]^+$.
4-((3,5-Bis(trifluoromethyl)benzyl)oxy)-3-((2,3-dichlorobenzyl)oxy)benzaldehyde (***17c***)**.**
Compound **17c** was prepared from 0.155 g (0.43 mmol) of intermediate **15** and 0.102 g (0.43 mmol) of 1-(bromomethyl)-2,3-dichlorobenzene (**7s**). Yield 0.213 g (96%), white solid; ^{1}H NMR (400 MHz, $CDCl_3$): δ (ppm) δ 9.90 (s, 1H), 7.99 – 7.96 (m, 2H), 7.89 – 7.86 (m, 1H), 7.61 – 7.56 (m, 2H), 7.54

(dd, *J* = 8.2, 1.9 Hz, 1H), 7.46 (dd, *J* = 8.1, 1.6 Hz, 1H), 7.28 – 7.22 (overlapped, 1H), 7.10 (d, *J* = 8.2 Hz, 1H), 5.32 (s, 2H), 5.28 (s, 2H); ESI-MS (m/z): 524.0 $[M+H]^+$.

4-((3,5-Bis(trifluoromethyl)benzyl)oxy)-3-((3,4-dichlorobenzyl)oxy)benzaldehyde (***17d***).

Compound **17d** was prepared from 0.250 g (0.69 mmol) of intermediate **15** and 0.165 g (0.69 mmol) of 4-(bromomethyl)-1,2-dichlorobenzene (**7q**). Yield 0.300 g (83%), white solid. ^{1}H NMR (400 MHz, $CDCl_3$): δ (ppm) 9.88 (s, 1H), 7.96 – 7.92 (m, 2H), 7.89 – 7.85 (m, 1H), 7.55 – 7.49 (m, 3H), 7.46 (d, *J* = 8.2 Hz, 1H), 7.32 (dd, *J* = 8.2, 2.0 Hz, 1H), 7.07 (d, *J* = 8.6 Hz, 1H), 5.31 (s, 2H), 5.14 (s, 2H); ESI-MS (m/z): 524.2 $[M+H]^+$.

Zhang, H. Q., Gong, F. H., Li, C. G., Zhang, C., Wang, Y. J., Xu, Y. G., et al. (2016) Design and discovery of 4-anilinoquinazoline-acylamino derivatives as EGFR and VEGFR-2 dual TK inhibitors. *Eur. J. Med. Chem.* 109, 371−379. doi: 10.1016/j.ejmech.2015.12.032

Carrasco-Gomez, R., Keppner-Witter, S., Hieke, M., Lange, L., Schneider, G., Schubert-Zsilavecz, M., et al. (2014). Vanillin-derived antiproliferative compounds influence Plk1 activity. *Bioorg. Med. Chem. Lett.* 24 (21), 5063−5069. doi: 10.1016/j.bmcl.2014.09.015

Zhang, J., Zhao, L., Zhu, C., Wu, Z., Zhang, G., Gan, X., et al. (2017) Facile Synthesis of Novel Vanillin Derivatives Incorporating a Bis(2-hydroxyethyl)dithhioacetal Moiety as Antiviral Agents. *J. Agric. Food Chem.* 65 (23), 4582−4588. doi: 10.1021/acs.jafc.7b01035

Plourde, G. L.; Spaetzel, R. R. (2002) Regioselective protection of the 4-Hydroxyl of 3,4-dihydroxy-benzaldehyde. *Molecules* 7 (9), 697−705. doi: 10.3390/70900697

Representative HPLC traces

Empower2 software

Apollo C18_Gr 5%_95%

SAMPLE INFORMATION

Sample Name:	1075-382-MZ-1054-A		
Sample Type:	Unknown	Sample Set Name:	16122020_Serviss_1
Vial:	17	Acq. Method Set:	Serviss Gr5%_iz_UV
Injection #:	1	Processing Method:	Gr5%
Injection Volume:	10.00 ul	Channel Name:	2998 Ch2 210nm@1.2nm
Run Time:	25.0 Minutes	Proc. Chnl. Descr.:	2998 Ch2 210nm@1.2nm
Date Acquired:	12/16/2020 12:39:36 PM EET	Acquired By:	System
Date Processed:	12/16/2020 1:20:51 PM EET		

Apollo C18-9 (4.6x150 mm)

15min Gr. 5%-95%ACN+0.1%H3PO4; 5min Iz. 95%ACN; 3min Gr. 95-5%ACN, 2min Iz. 5%ACN; F=1mL/min; 40oC

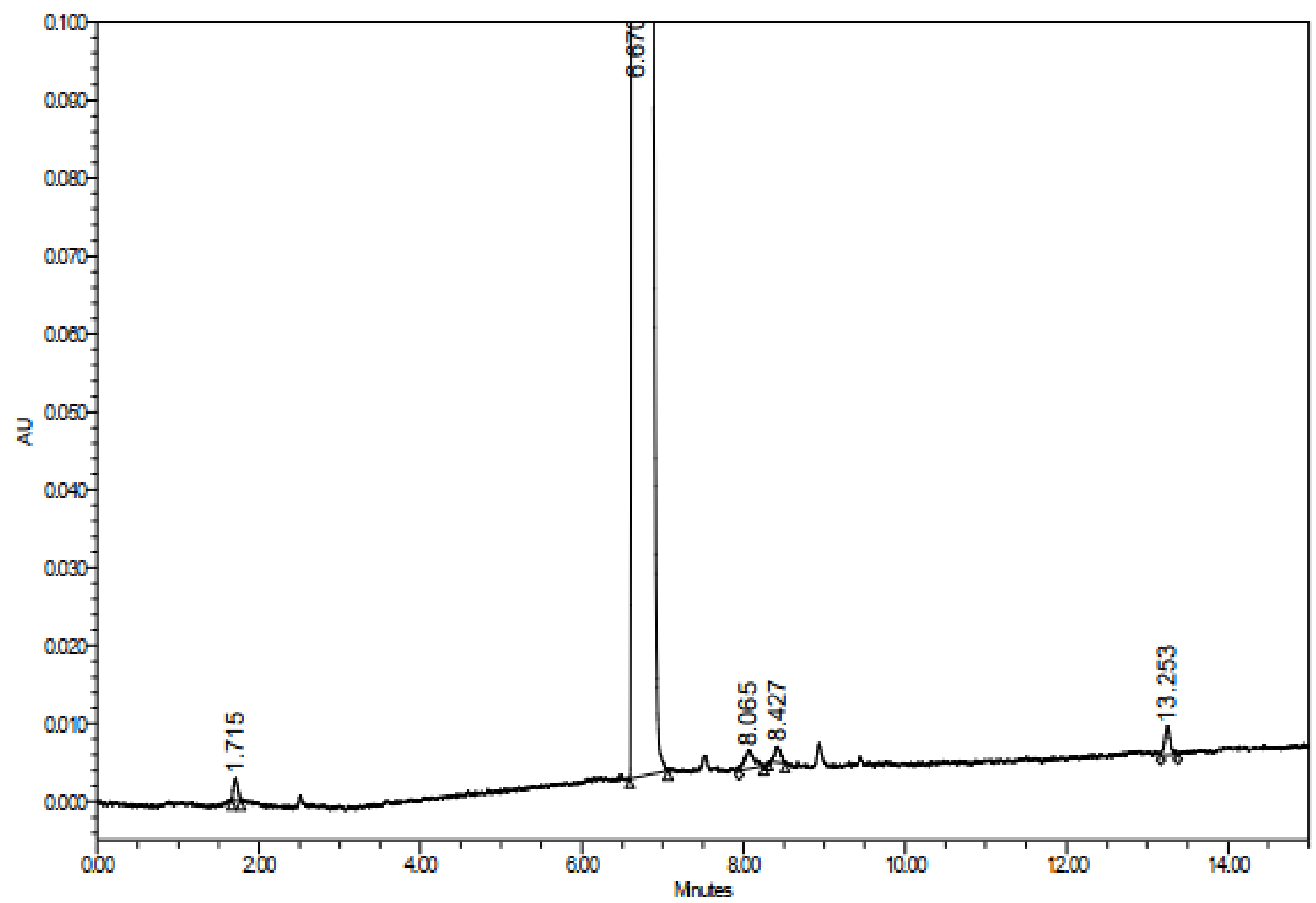

C=0.5 mg/ml (5%ACN0.1%PA)

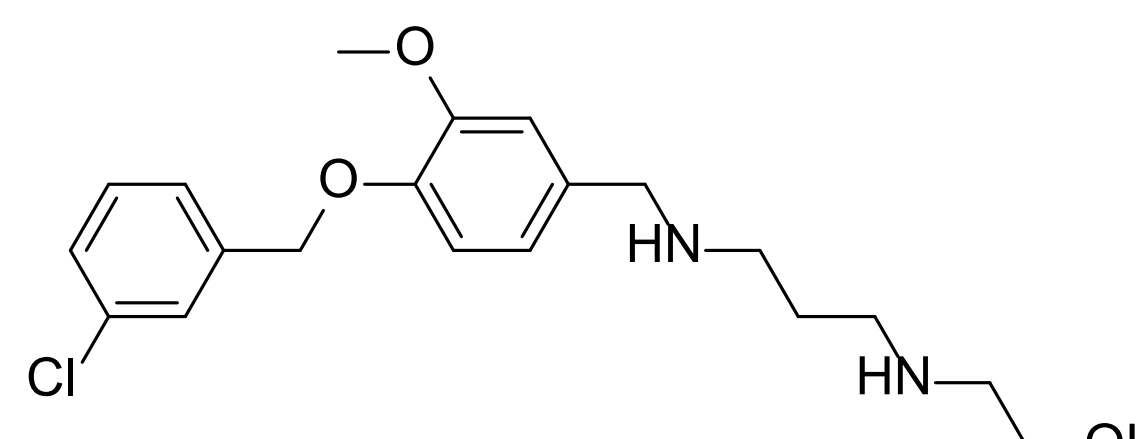

1a

	RT	Area	% Area	Height	EP Plate Count	Width @50%	Resolution	Selectivity
1	1.715	11211	0.05	2881	3849	0.07		
2	6.670	21403208	99.73	2163698	8772	0.17	25.13	24.06
3	8.065	19603	0.09	2425	26536	0.12	5.79	1.27
4	8.427	10196	0.05	1984	72048	0.07	2.24	1.06
5	13.253	16696	0.08	3701	215038	0.07	40.34	1.70
Sum		21460913.5						

Reported by User: System
Report Method: Apollo C18_Gr 5%_95%
Report Method ID: 258 25885
Page: 1 of 2

Project Name: Alliance-1_2020 PDA
Date Printed:
12/16/2020
1:21:22 PM Europe/Riga

Empower2 software

Adamas C18_Gr 5%_95%AU

SAMPLE INFORMATION

Sample Name:	817-419-IL-573		
Sample Type:	Unknown	Sample Set Name:	20_10_2021_serviss
Vial:	14	Acq. Method Set:	Serviss Gr5%_iz_UV
Injection #:	1	Processing Method:	Gr_5%
Injection Volume:	10.00 ul	Channel Name:	2998 Ch2 210nm@1.2nm
Run Time:	25.0 Minutes	Proc. Chnl. Descr.:	2998 Ch2 210nm@1.2nm
Date Acquired:	10/20/2021 10:49:48 AM EET	Acquired By:	System
Date Processed:	10/20/2021 12:05:38 PM EET		

Adamas C18-2 (4,6x150 mm)
15min Gr 5%-95%ACN+0.1%H3PO4; 5 min IZ_95%ACN 3min Gr 95-5%ACN, 2min IZ 5%ACN; 1mL/min; 40oC

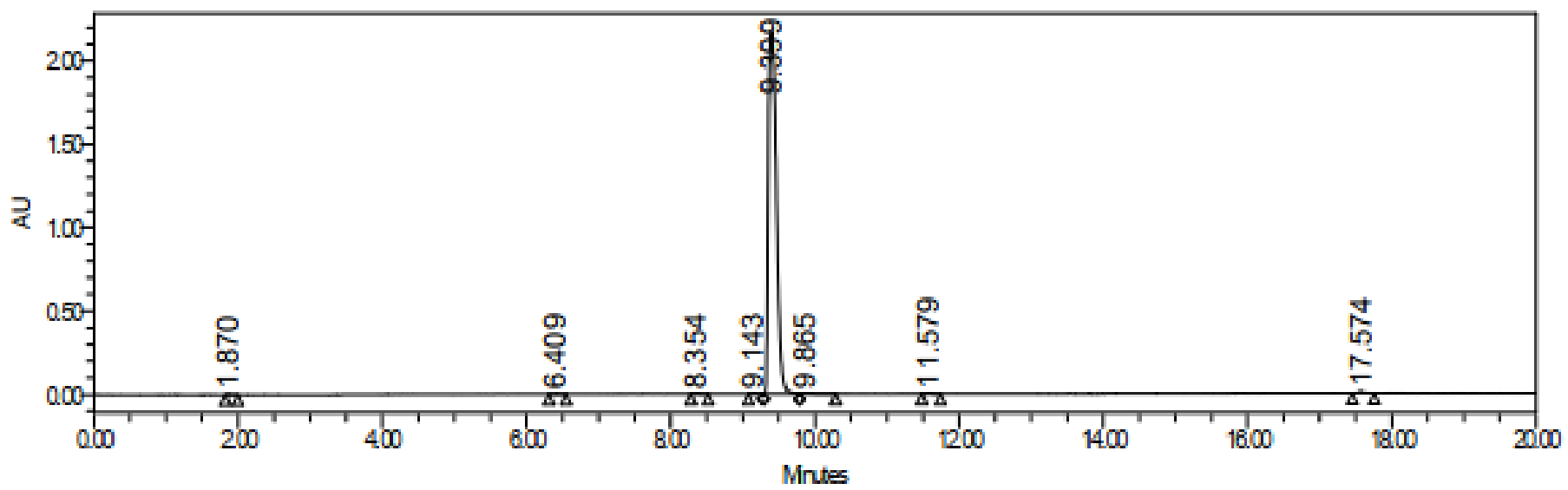

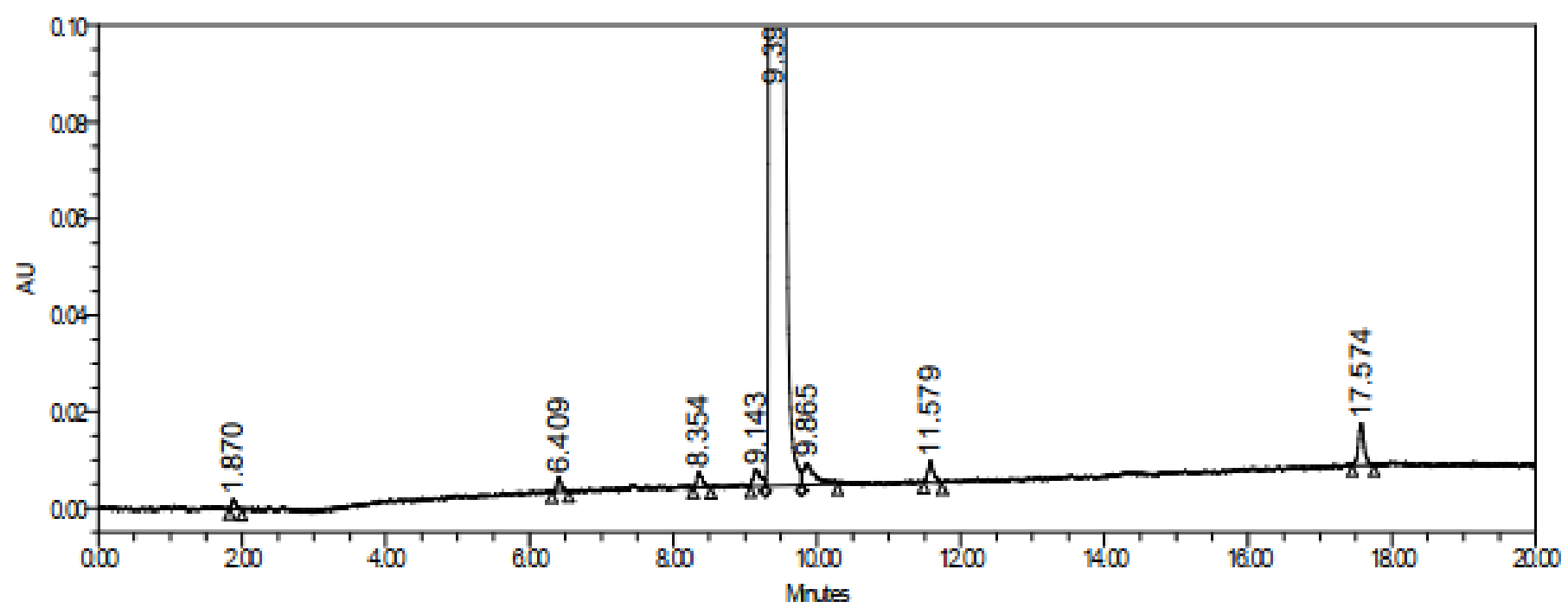

C=0.5mg/ml (5%ACN+0.1%PA)

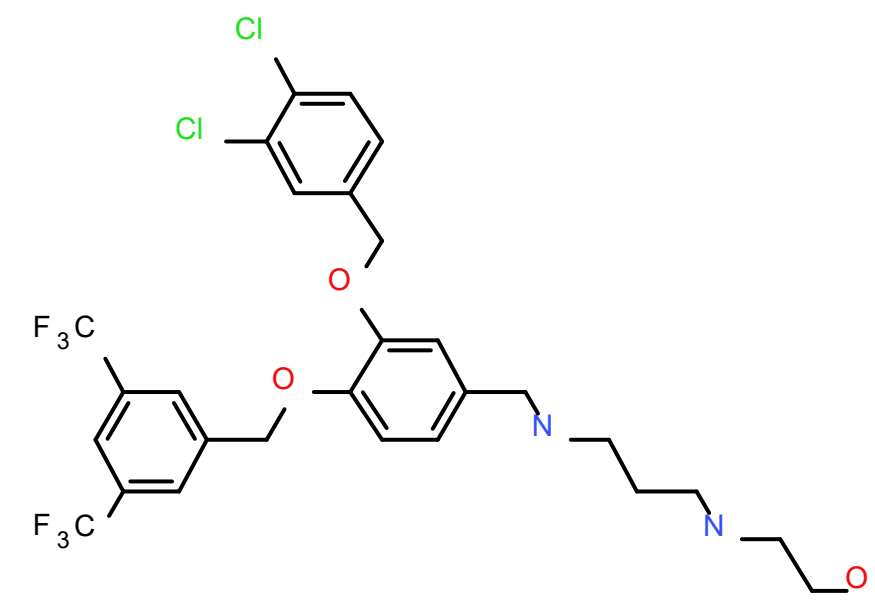

4e

	RT	Area	%Area	Height	EP Plate Count	Width @50%	Resolution	Selectivity
1	1.870	8375	0.05	1981	3879	0.0707		
2	6.409	13721	0.08	3051	54818	0.0644	39.639	13.259
3	8.354	17963	0.10	3124	58001	0.0816	15.715	1.396
4	9.143	25359	0.14	3482	39410	0.1084	4.898	1.115
5	9.399	17399215	98.93	2166647	29441	0.1289	1.273	1.034
6	9.865	49725	0.28	4496				1.059
7	11.579	27063	0.15	4439	89395	0.0911		1.205
8	17.574	45164	0.26	8686	321119	0.0730	43.102	1.595
Sum		17586585.0						

	R
1	10.
2	10.
3	12.
4	14.

Reported by User: System
Report Method: Adamas C18_Gr 5%_95%AU
Report Method ID: 2105 21050
Page: 1 of 2

Project Name: Alliance-1_2021 PDA
Date Printed:
10/20/2021
12:05:53 PM Europe/Riga

NMR Spectra of Synthesised Compounds

2-((3-((4-((3-Chlorobenzyl)oxy)-3-methoxybenzyl)amino)propyl)amino)ethan-1-ol (***1a***).

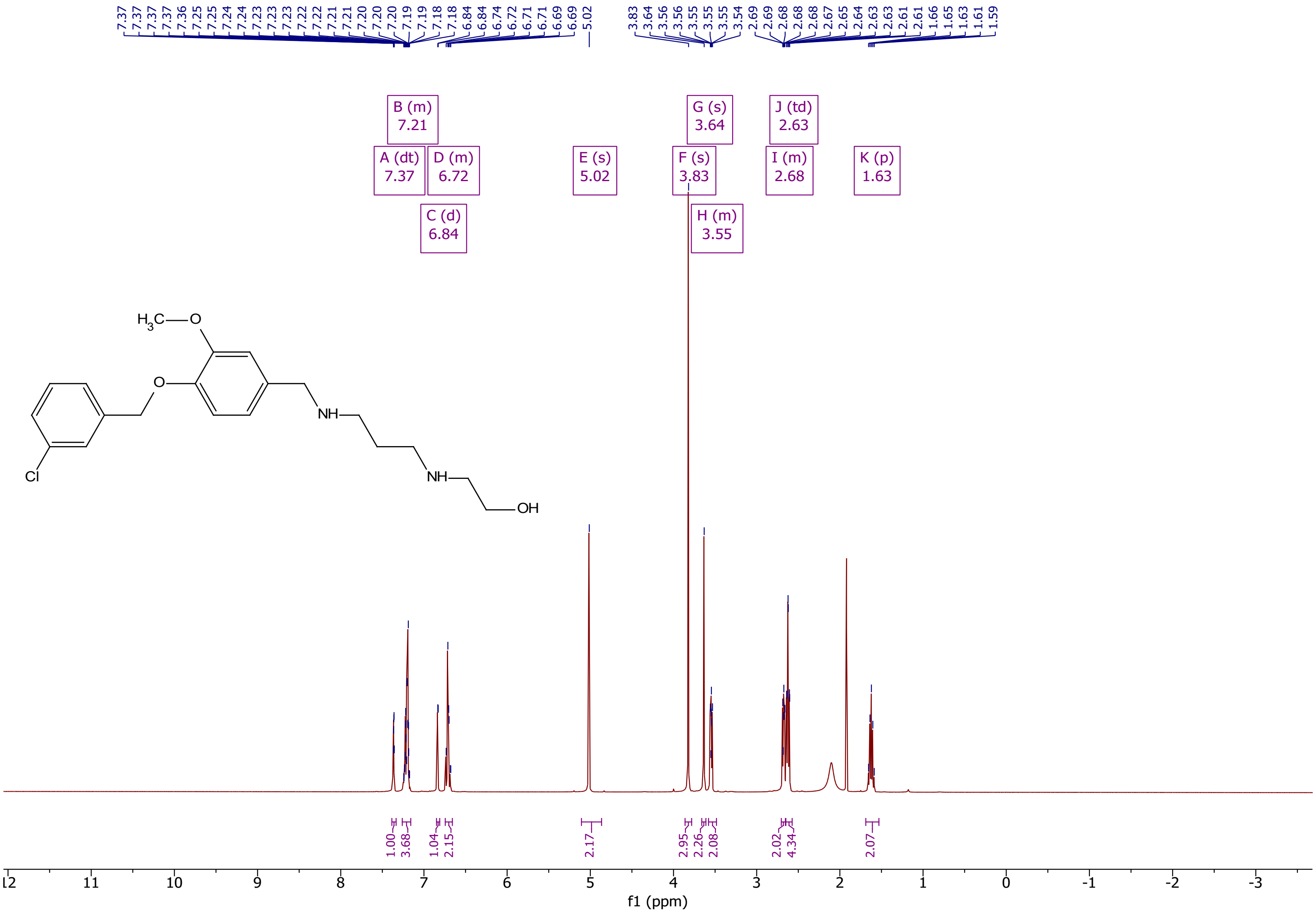

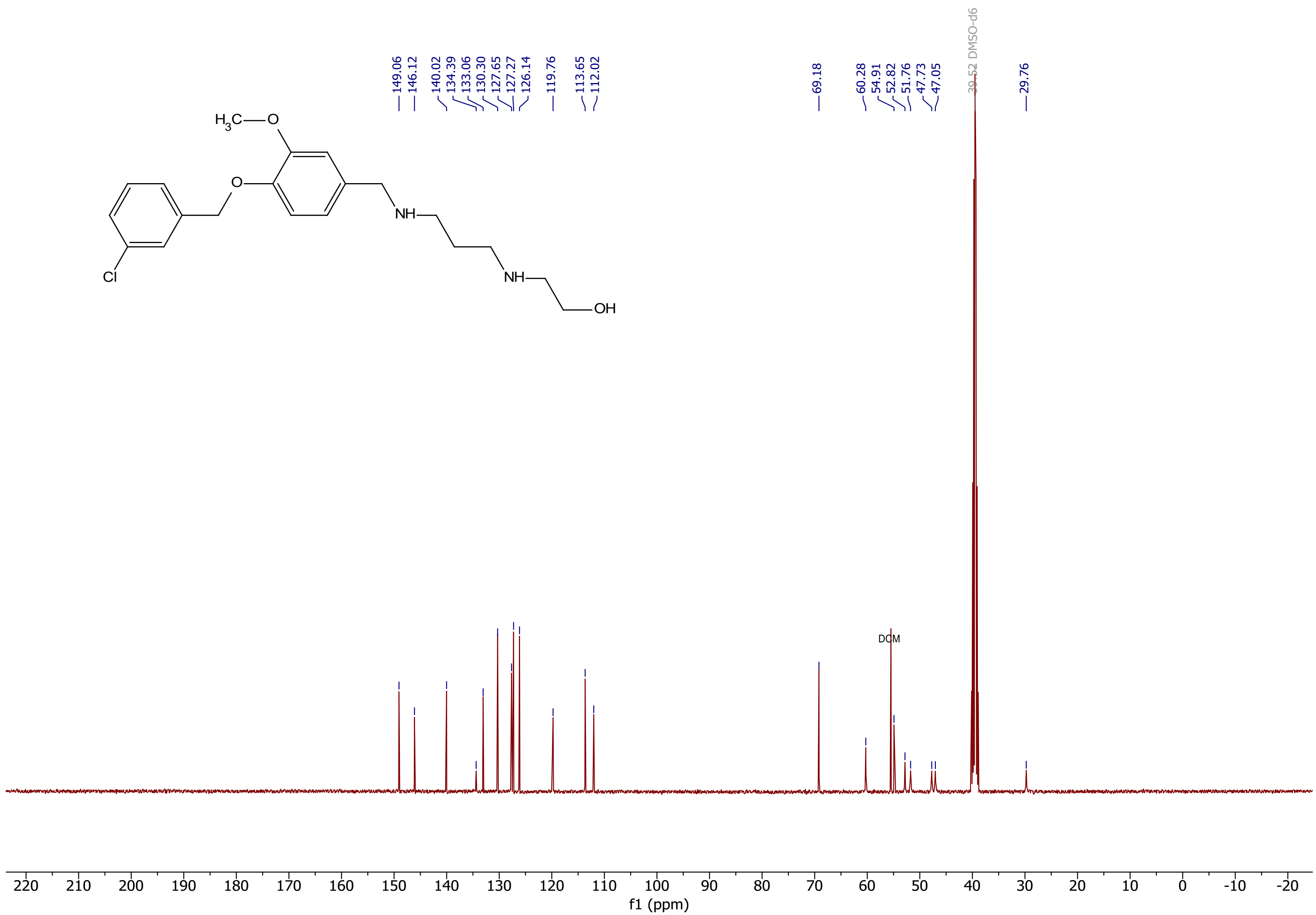

149.06
146.12
140.02
134.39
133.06
130.30
127.65
127.27
126.14
119.76
113.65
112.02
69.18
60.28
54.91
52.82
51.76
47.73
47.05
39.52 DMSO-d6
29.76
DCM
H3C—O
O
NH
NH
OH
Cl
220 210 200 190 180 170 160 150 140 130 120 110 100 90 80 70 60 50 40 30 20 10 0 -10 -20
f1 (ppm)

2-((2-((4-((3-Chlorobenzyl)oxy)-3-methoxybenzyl)amino)ethyl)amino)ethan-1-ol (***1b***).

OSM3-MZ-1141.10.1.1r

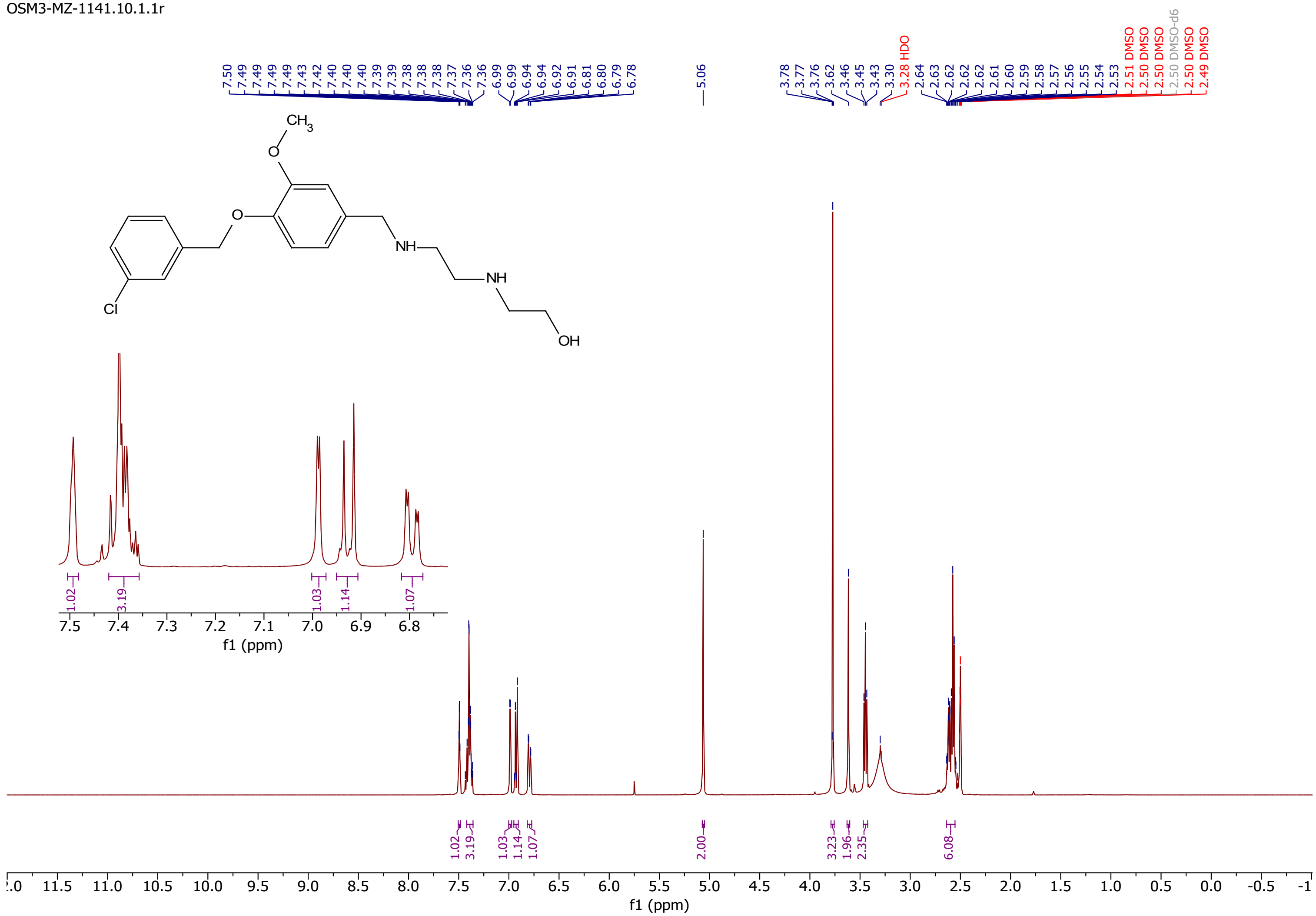

OSM3-MZ-1141.11.1.1r

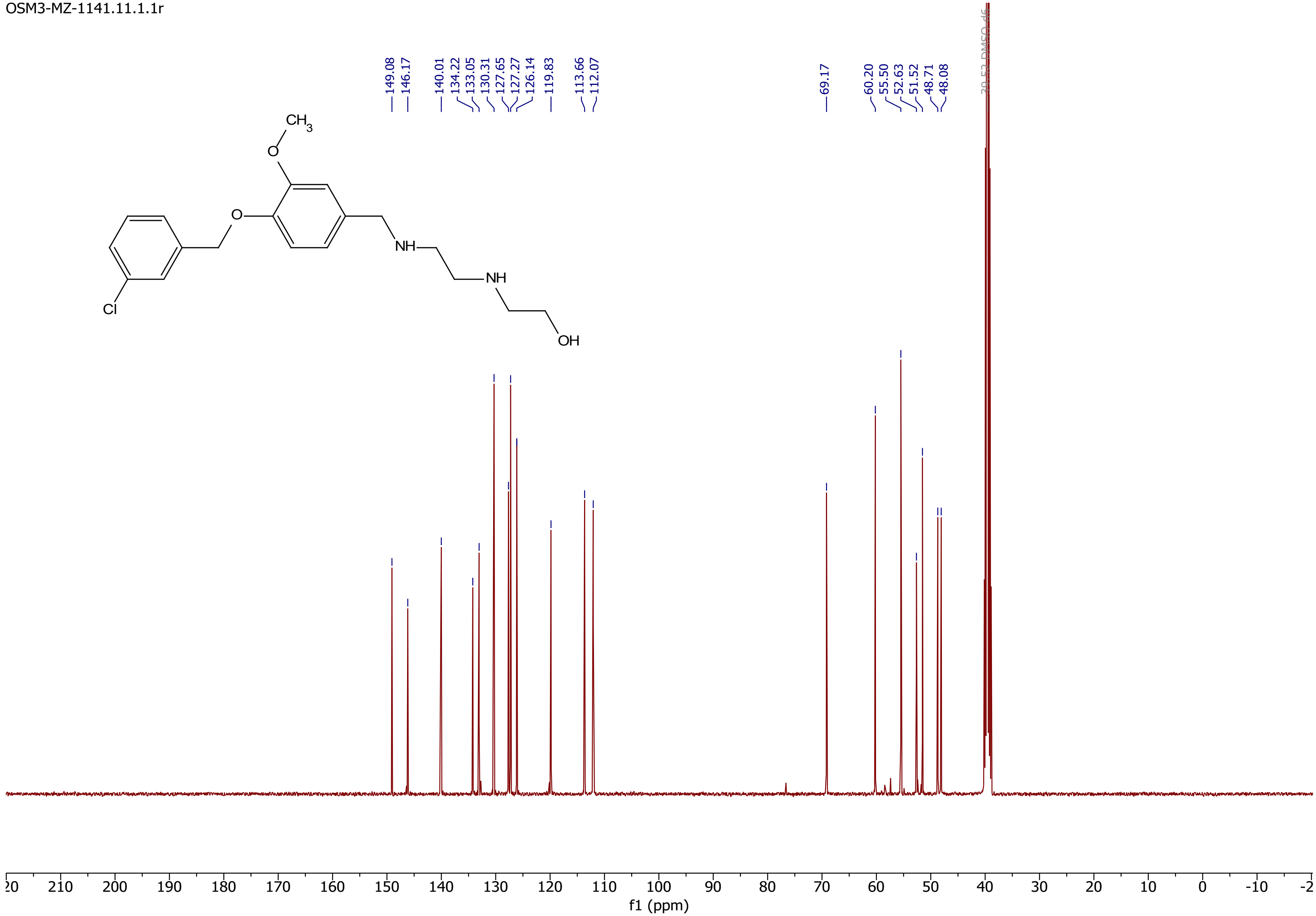

*2-((4-((3-Chlorobenzyl)oxy)-3-methoxybenzyl)amino)ethan-1-ol (**1c**).*

OSM3-MZ-1142.10.1.1r

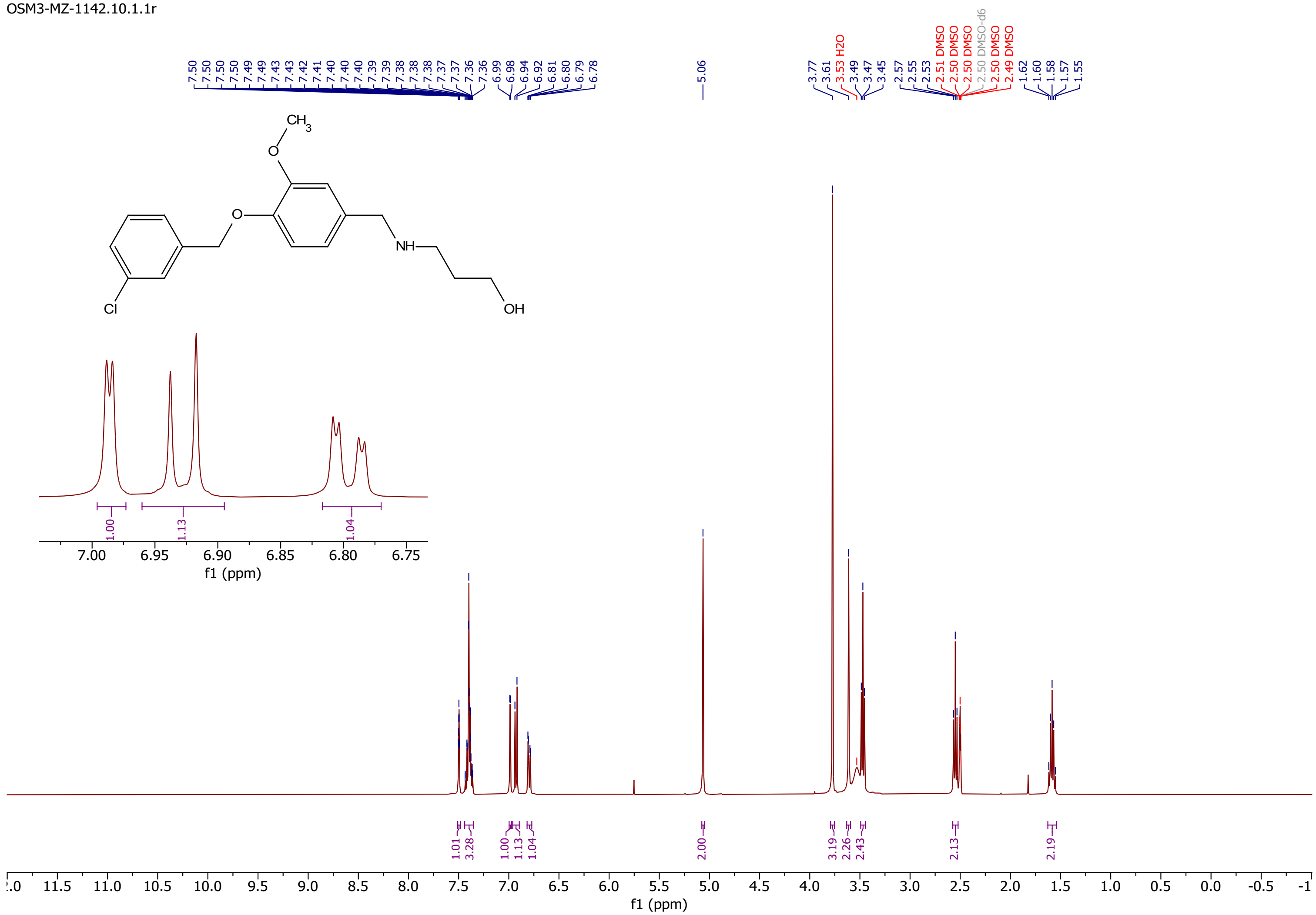

OSM3-MZ-1142.11.1.1r

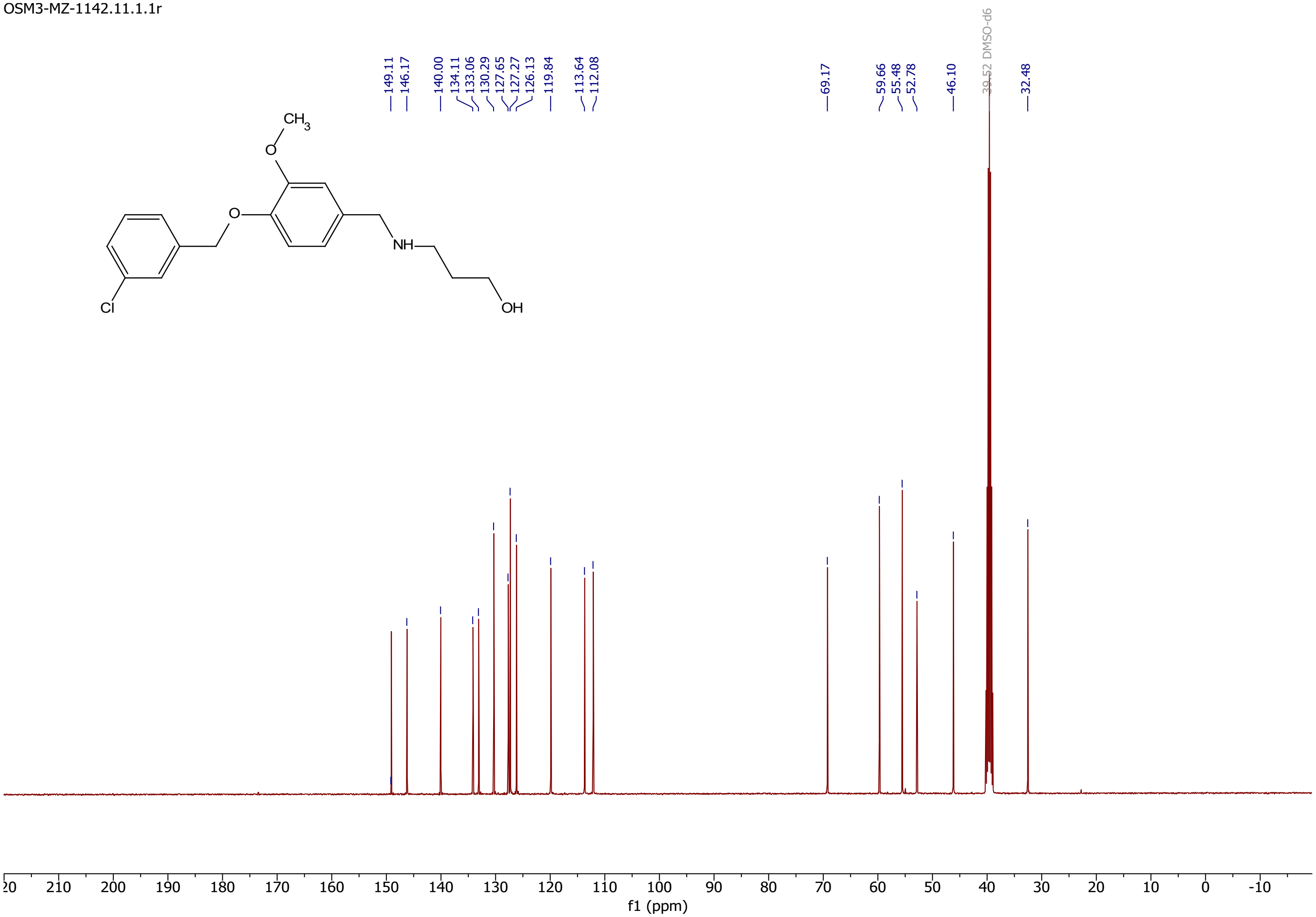

2-((3-((4-(Benzyloxy)-3-methoxybenzyl)amino)propyl)amino)ethan-1-ol (***2b***).

OSM3-MZ-1128.10.1.1r

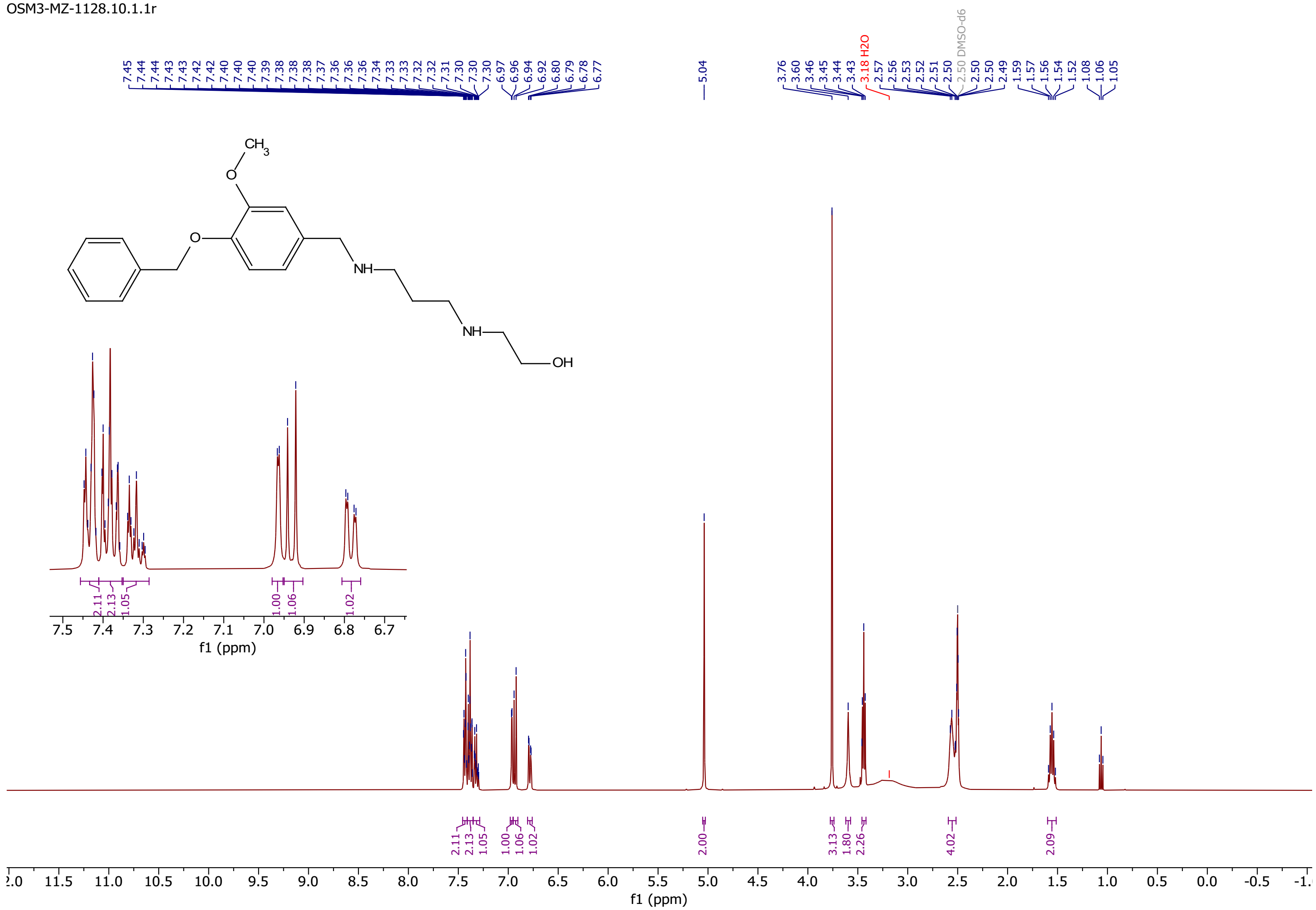

2-((3-((4-((2-Chlorobenzyl)oxy)-3-methoxybenzyl)amino)propyl)amino)ethan-1-ol (***2c***).

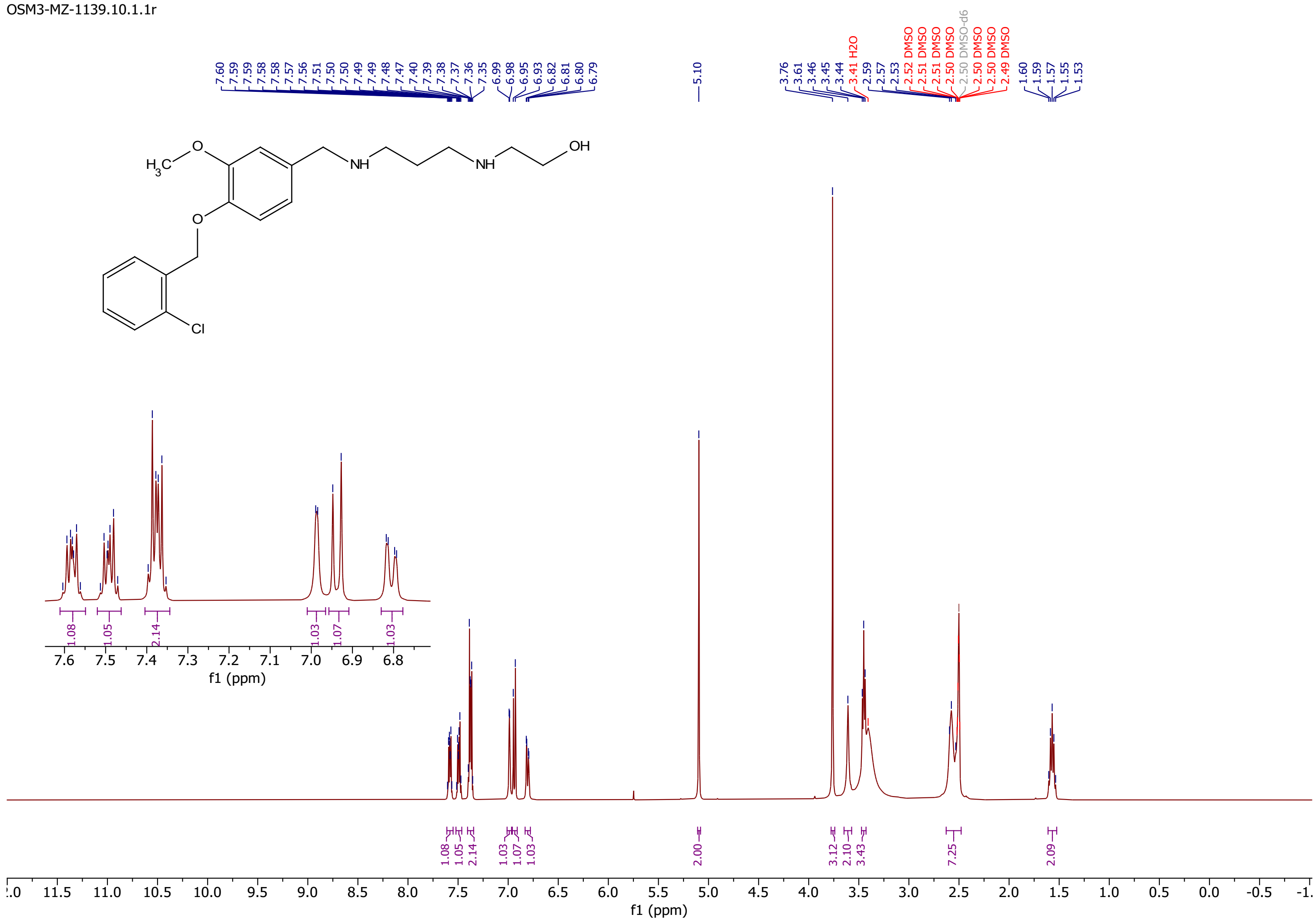

OSM3-MZ-1139.11.1.1r

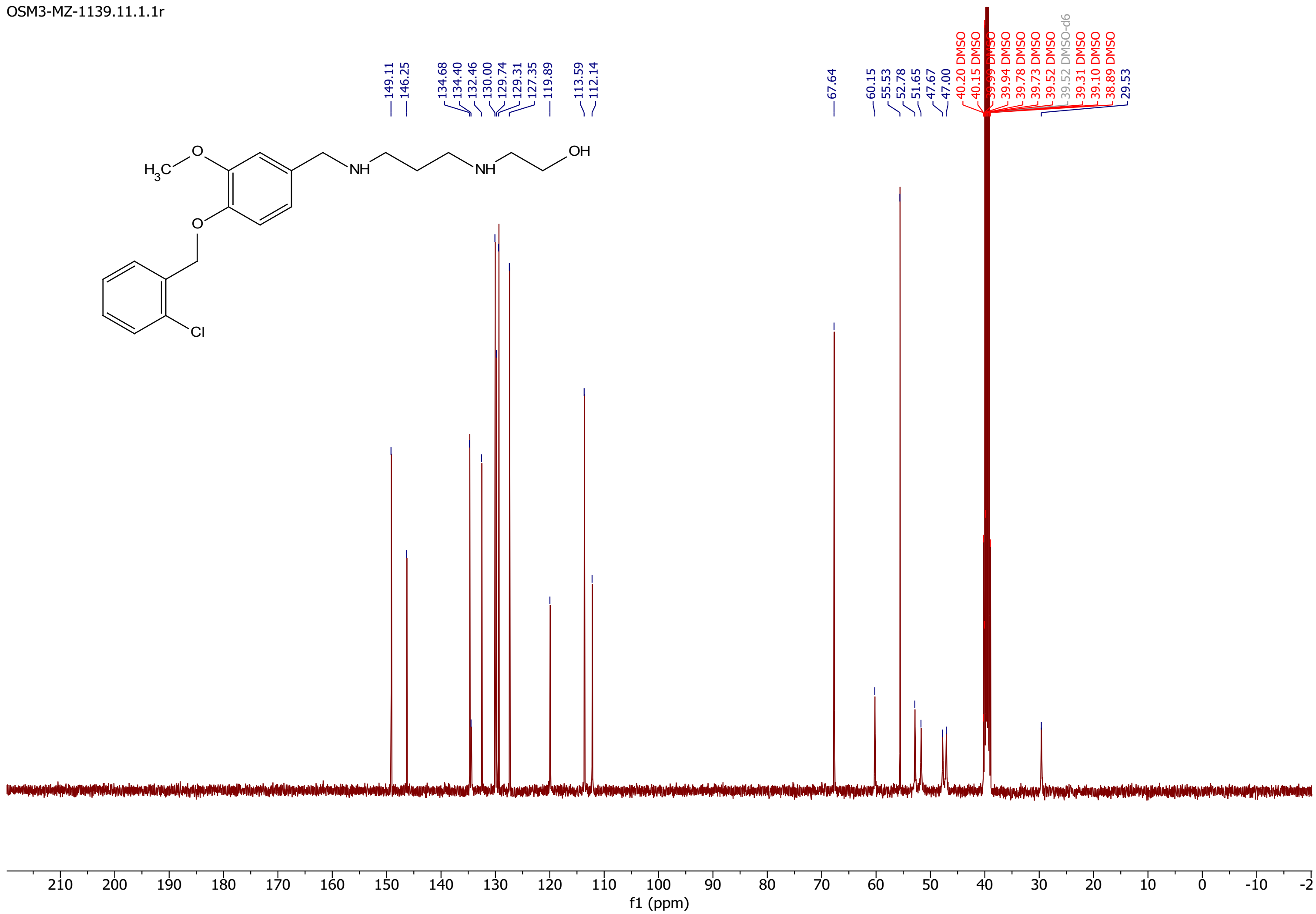

2-((3-((4-((4-Chlorobenzyl)oxy)-3-methoxybenzyl)amino)propyl)amino)ethan-1-ol (***2d***).

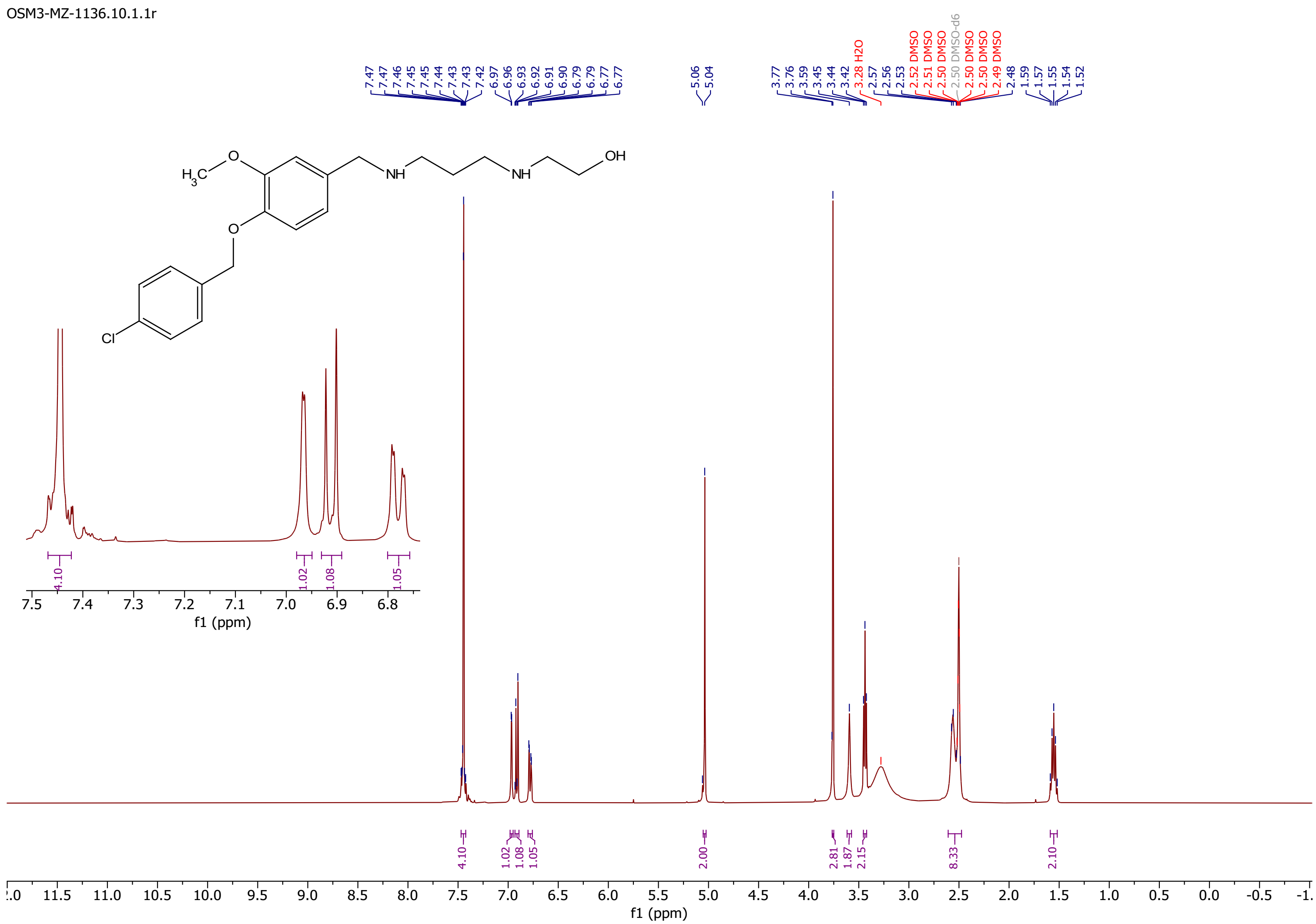

OSM3-MZ-1136.11.1.1r

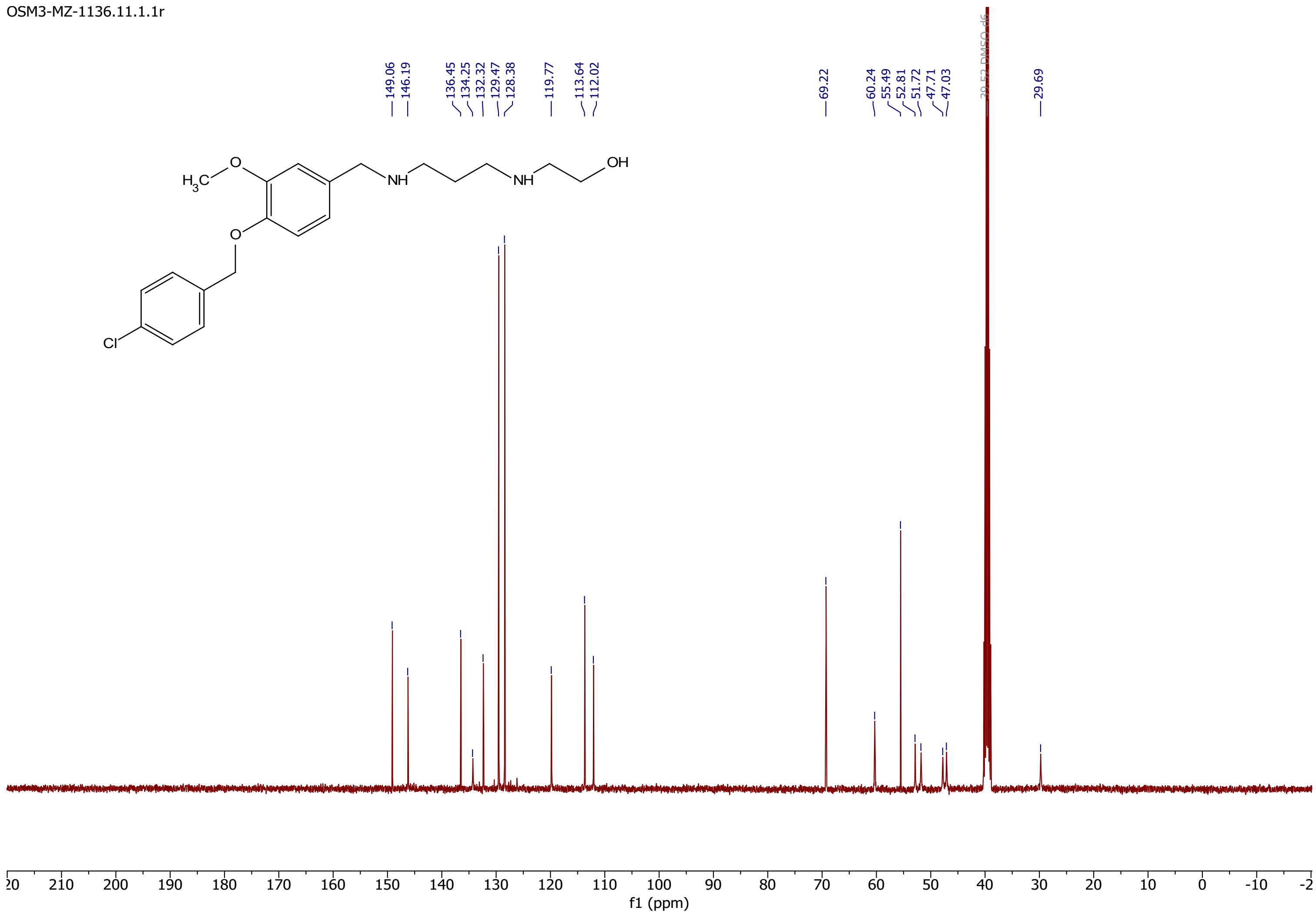

2-((3-((4-((3-Flurobenzyl)oxy)-3-methoxybenzyl)amino)propyl)amino)ethan-1-ol (***2e***).

OSM3-MZ-1148.10.1.1r

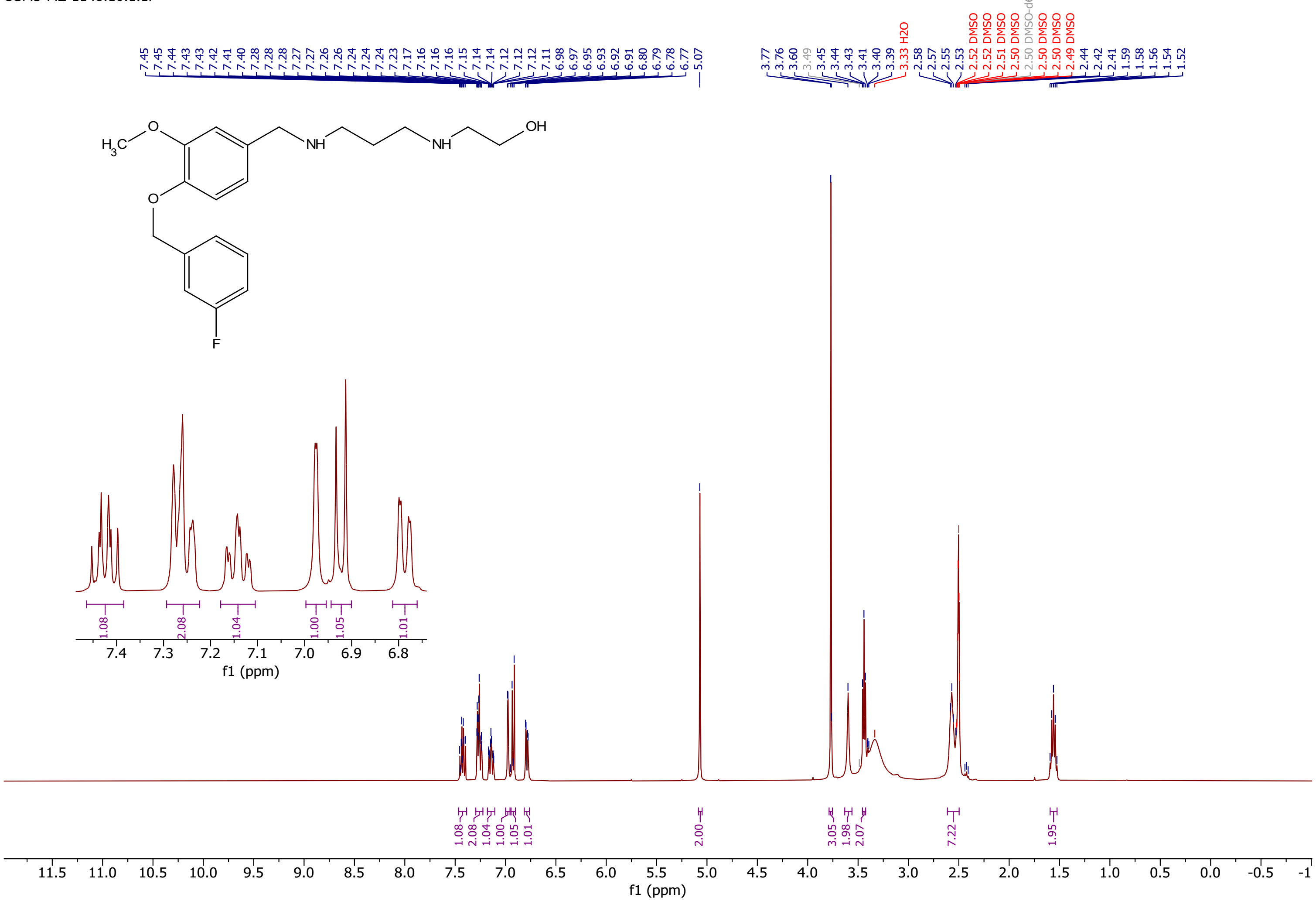

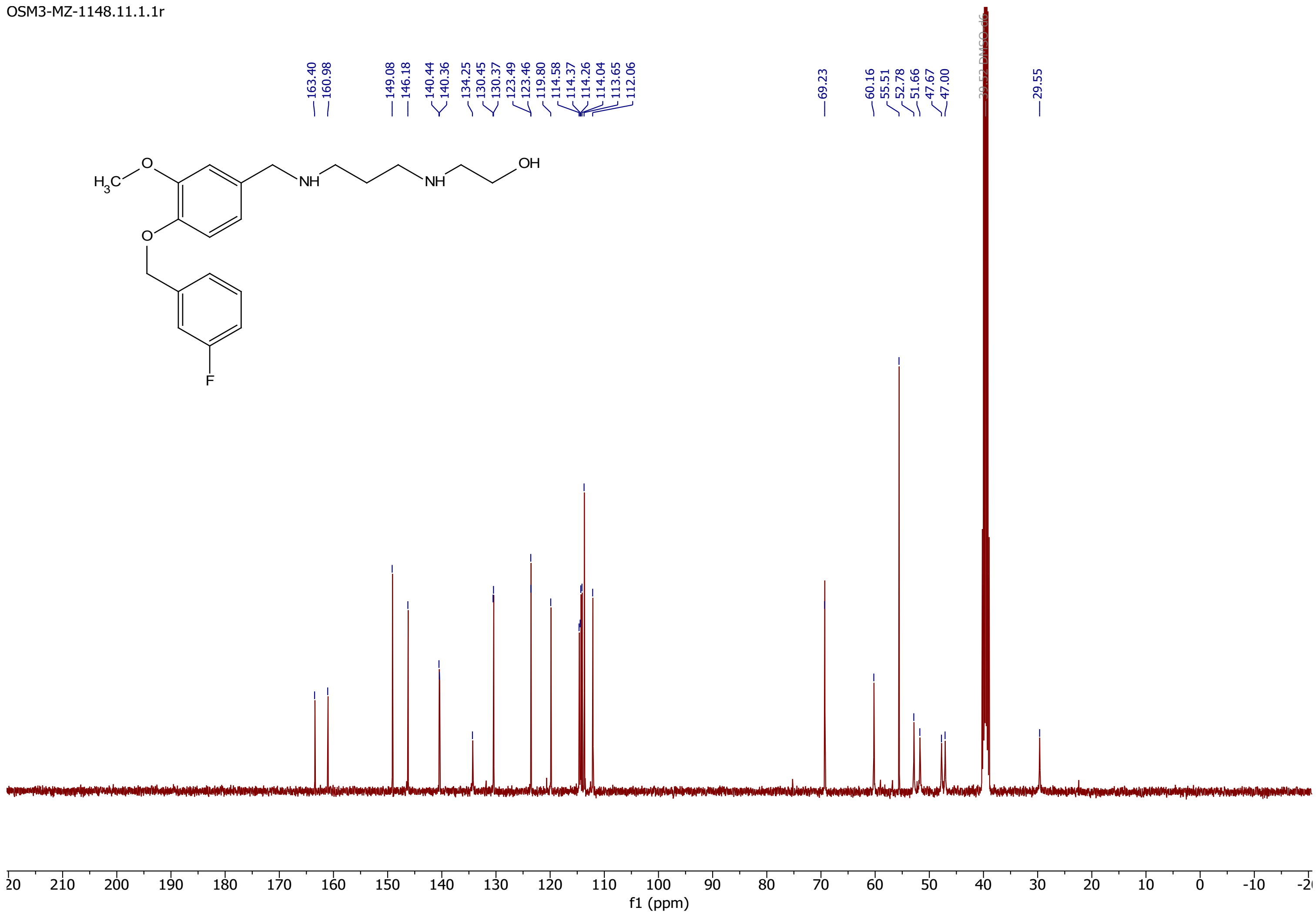
OSM3-MZ-1148.11.1.1r
163.40
160.98
149.08
146.18
140.44
140.36
134.25
130.45
130.37
123.49
123.46
119.80
114.58
114.37
114.26
114.04
113.65
112.06
69.23
60.16
55.51
52.78
51.66
47.67
47.00
39.52 DMSO-d6
29.55
H3C
O
O
NH
NH
OH
F
f1 (ppm)

2-((3-((4-((4-Bromobenzyl)oxy)-3-methoxybenzyl)amino)propyl)amino)ethan-1-ol (***2f***)**.**

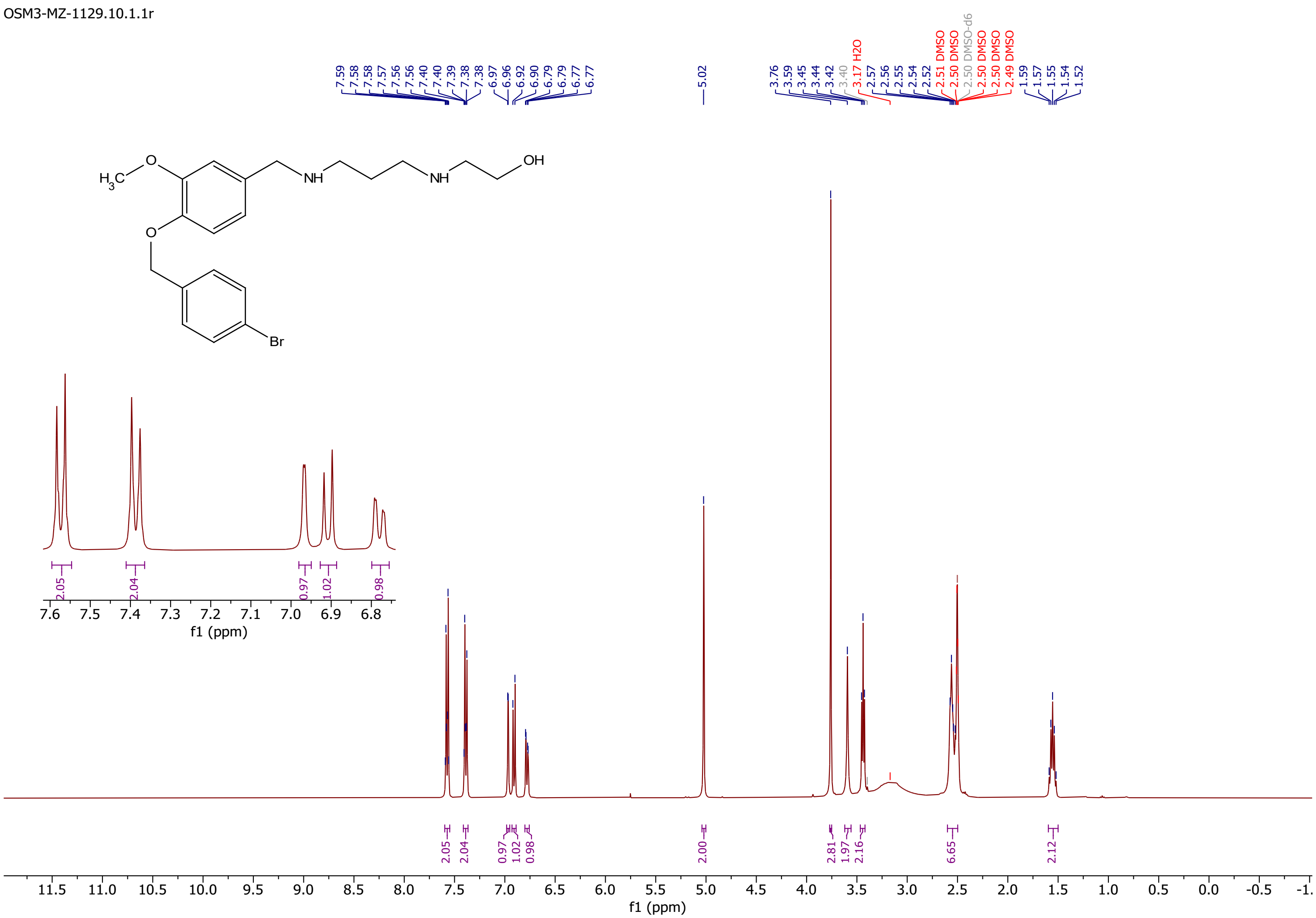

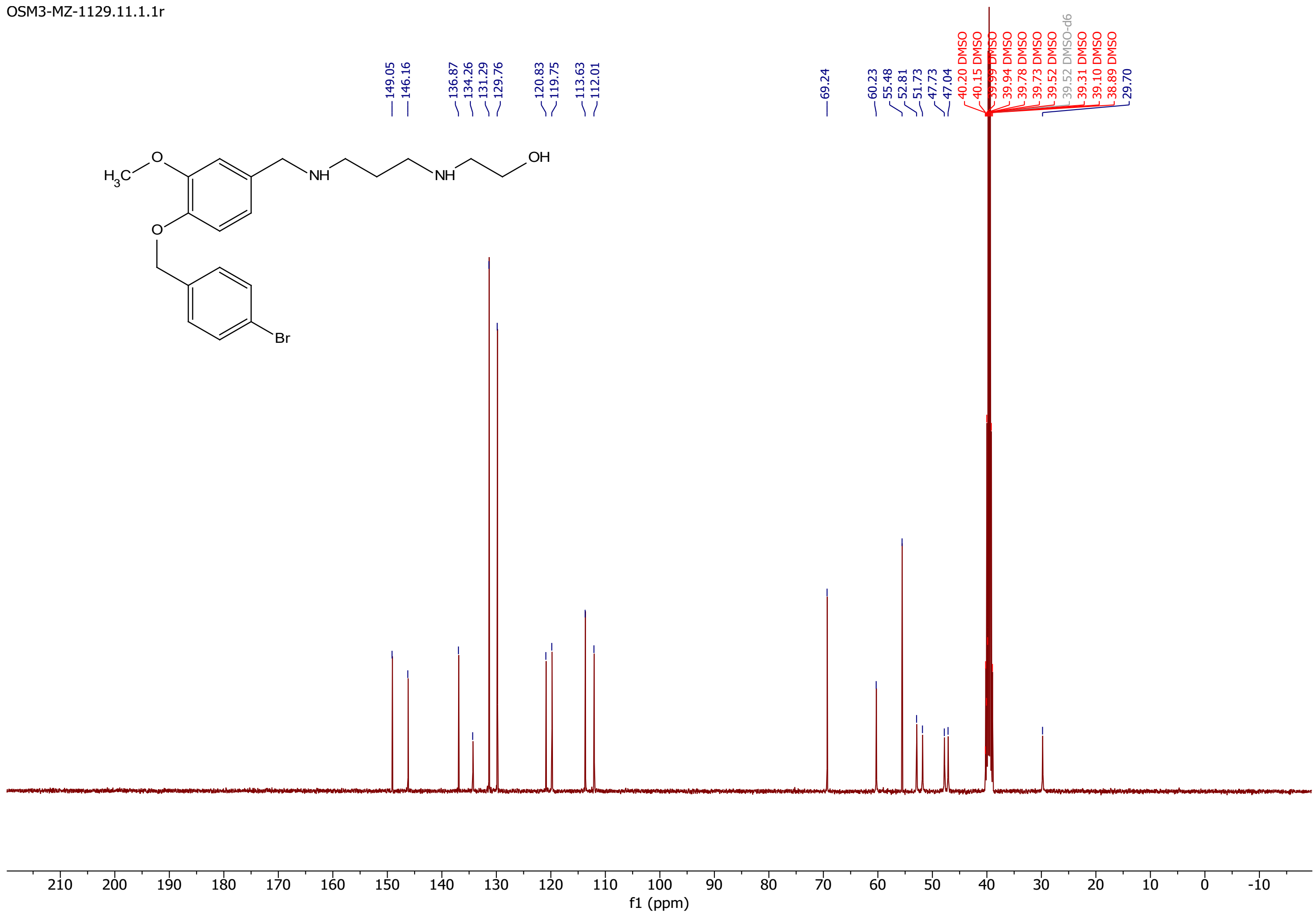
OSM3-MZ-1129.11.1.1r
149.05
146.16
136.87
134.26
131.29
129.76
120.83
119.75
113.63
112.01
69.24
60.23
55.48
52.81
51.73
47.73
47.04
40.20 DMSO
40.15 DMSO
39.99 DMSO
39.94 DMSO
39.78 DMSO
39.73 DMSO
39.52 DMSO
39.52 DMSO-d6
39.31 DMSO
39.10 DMSO
38.89 DMSO
29.70
H3C
O
O
NH
NH
OH
Br
210 200 190 180 170 160 150 140 130 120 110 100 90 80 70 60 50 40 30 20 10 0 -10
f1 (ppm)

*2-((3-((4-((3-Bromobenzyl)oxy)-3-methoxybenzyl)amino)propyl)amino)ethan-1-ol (**2g**).*

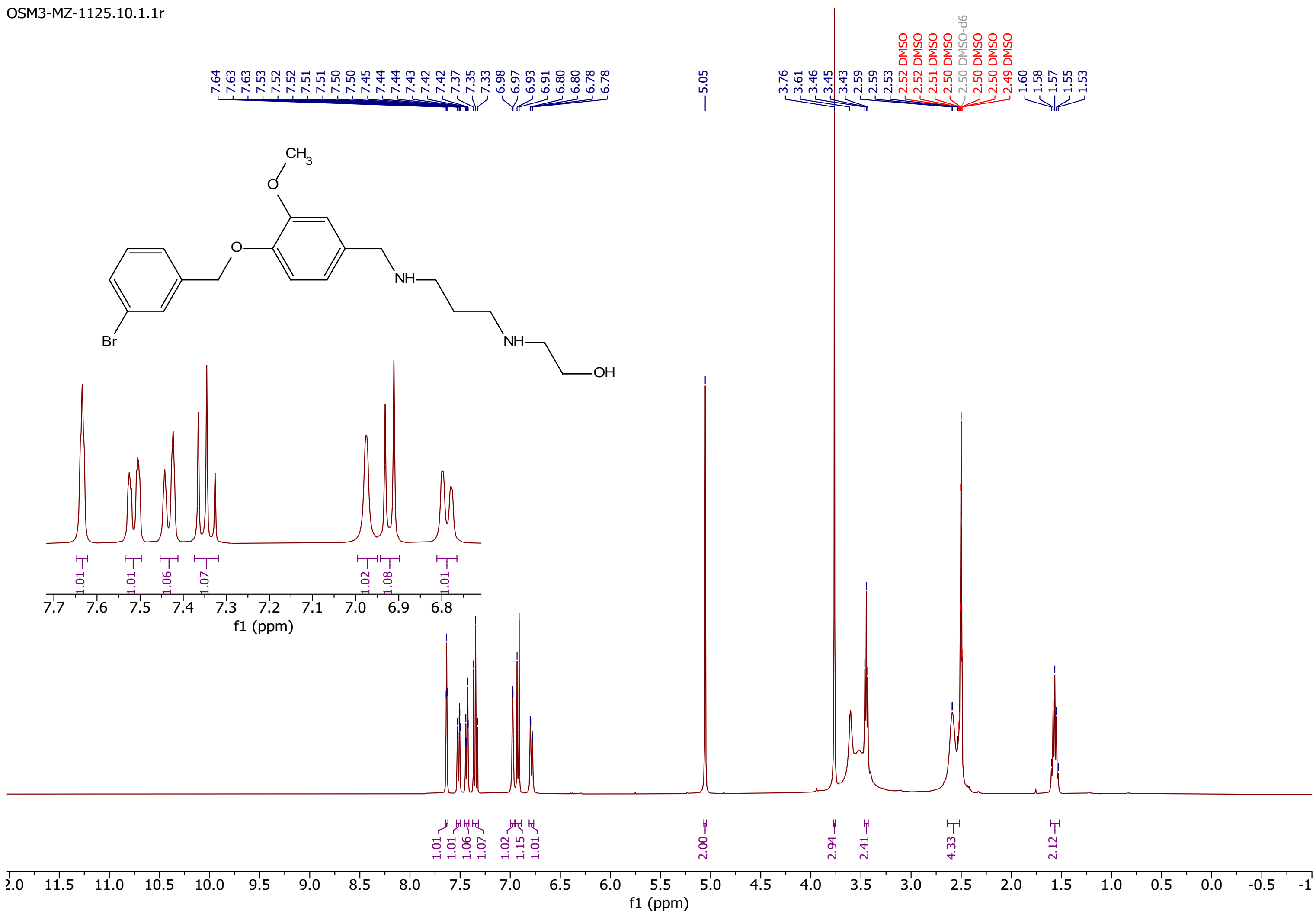

OSM3-MZ-1125.11.1.1r

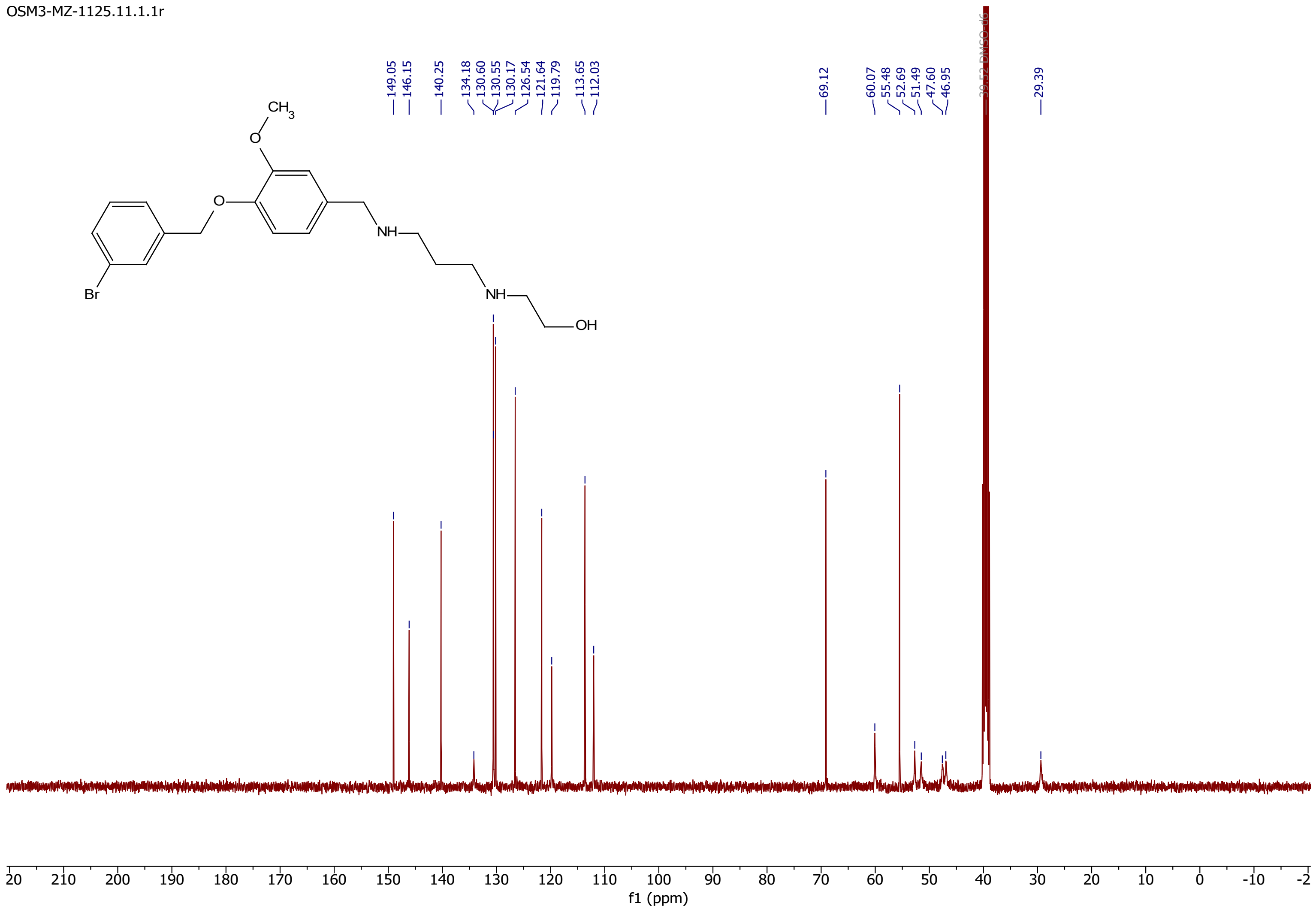

2-((3-((4-((4-Iodobenzyl)oxy)-3-methoxybenzyl)amino)propyl)amino)ethan-1-ol (***2h***).

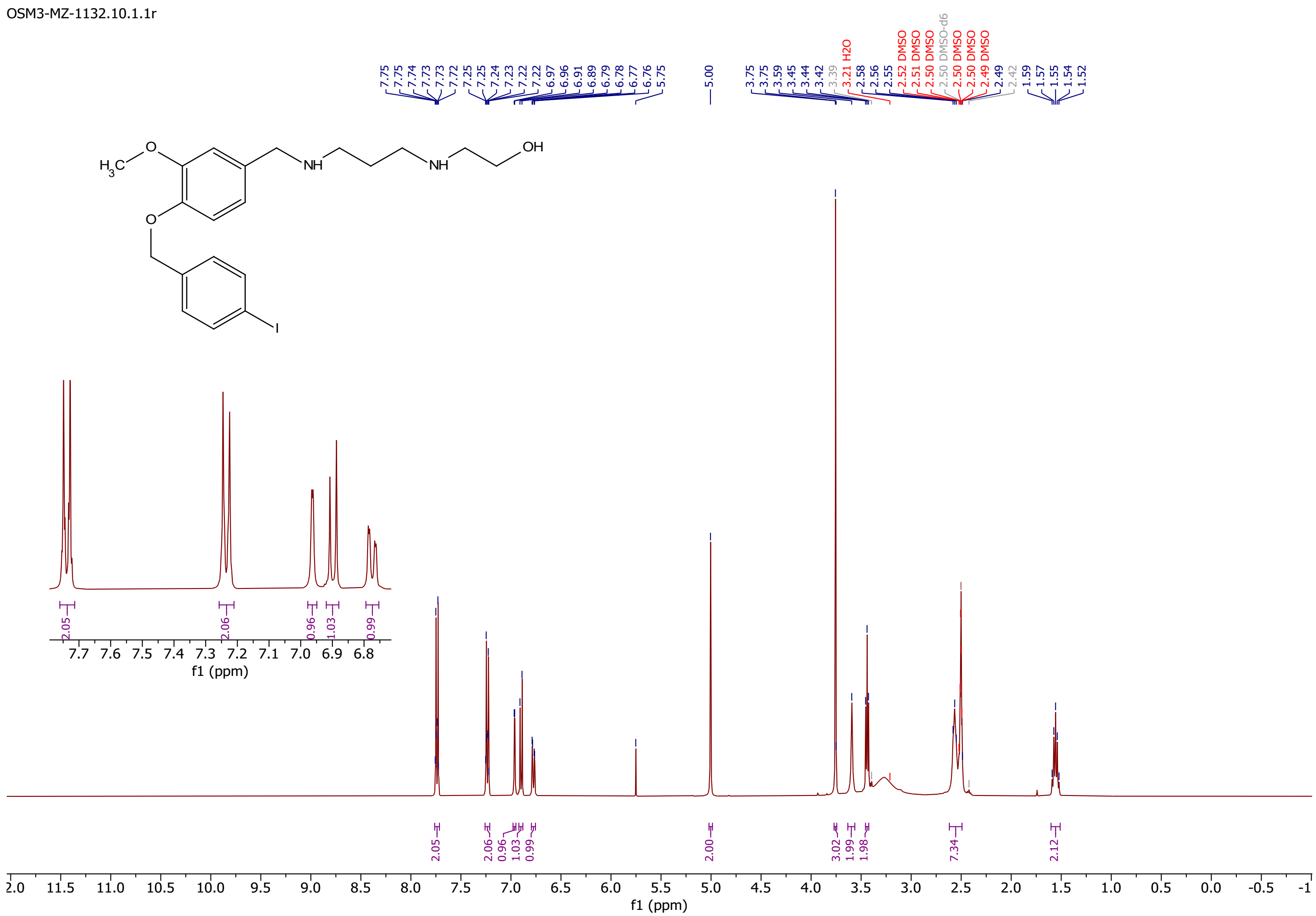

OSM3-MZ-1132.11.1.1r

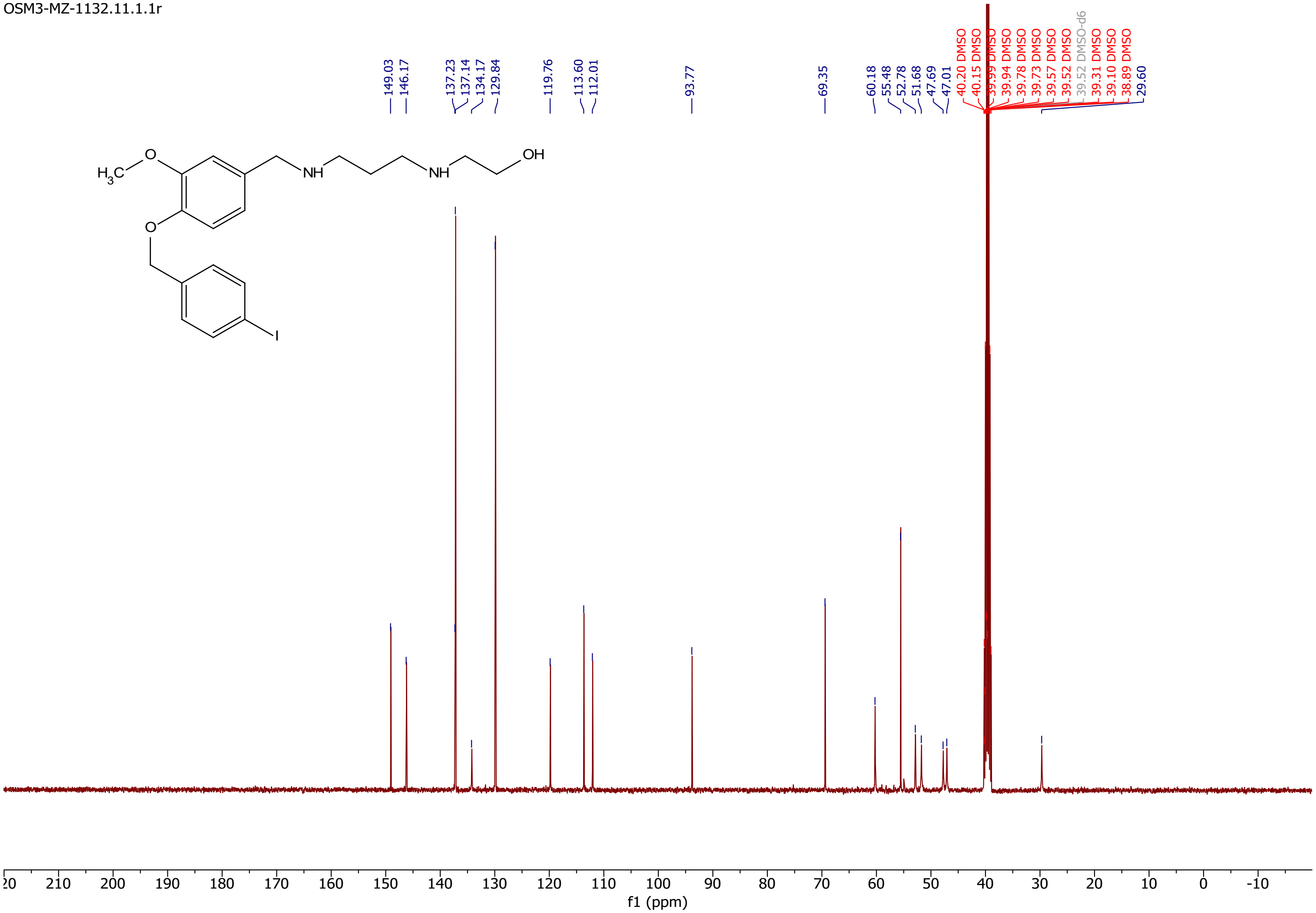

3-((4-(((3-((2-Hydroxyethyl)amino)propyl)amino)methyl)-2-methoxyphenoxy)- benzonitrile (***2i***).

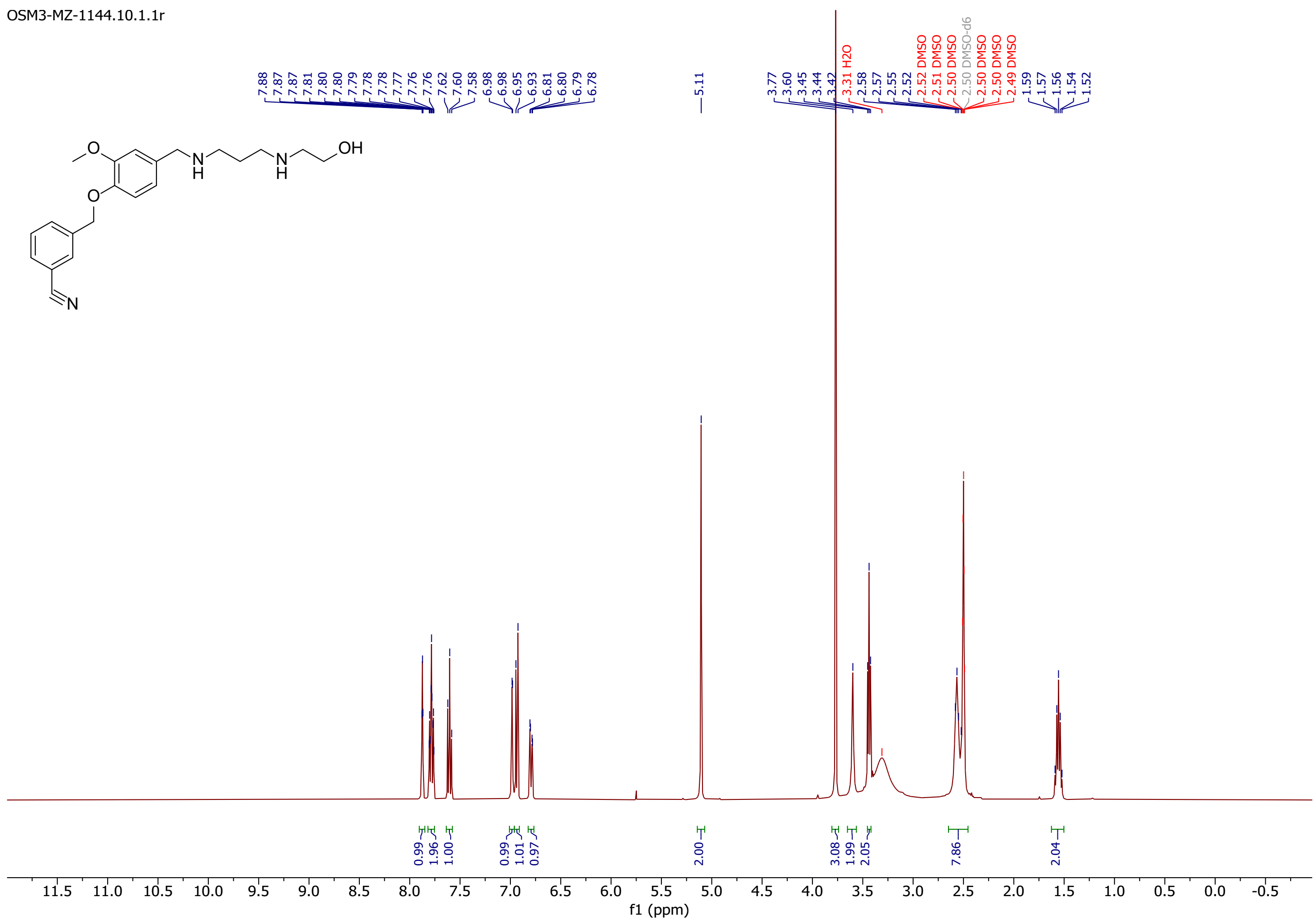

OSM3-MZ-1144.11.1.1r

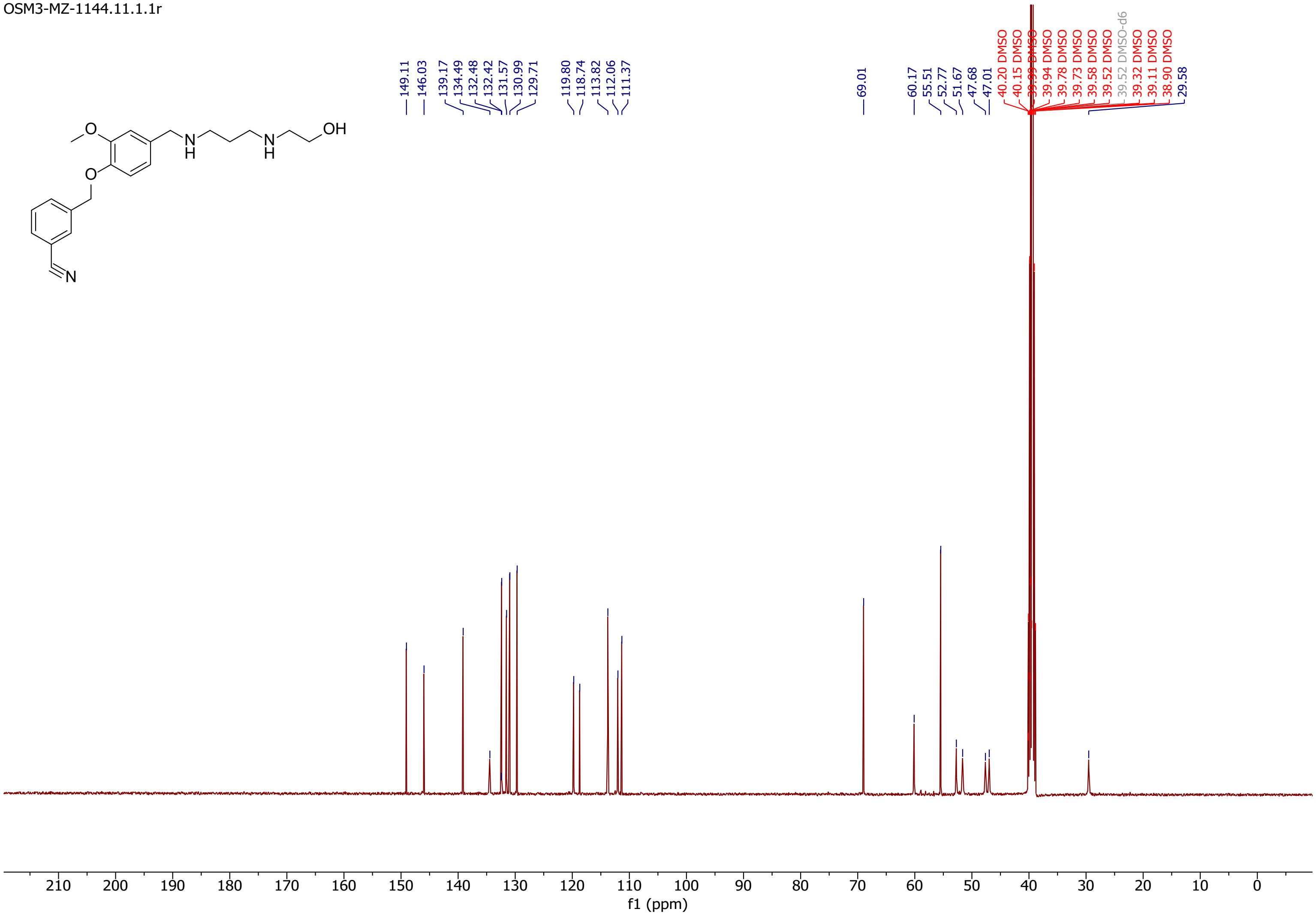

*2-((3-((3-Methoxy-4-((3-methoxybenzyl)oxy)benzyl)amino)propyl)amino)ethan-1-ol (**2j**).*

OSM3-MZ-1137.10.1.1r

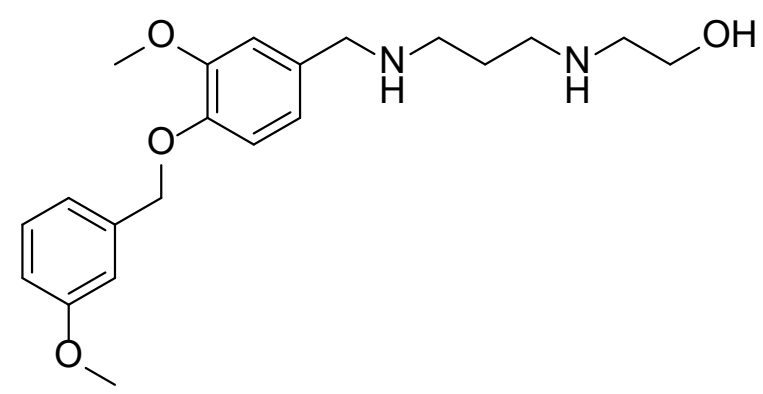

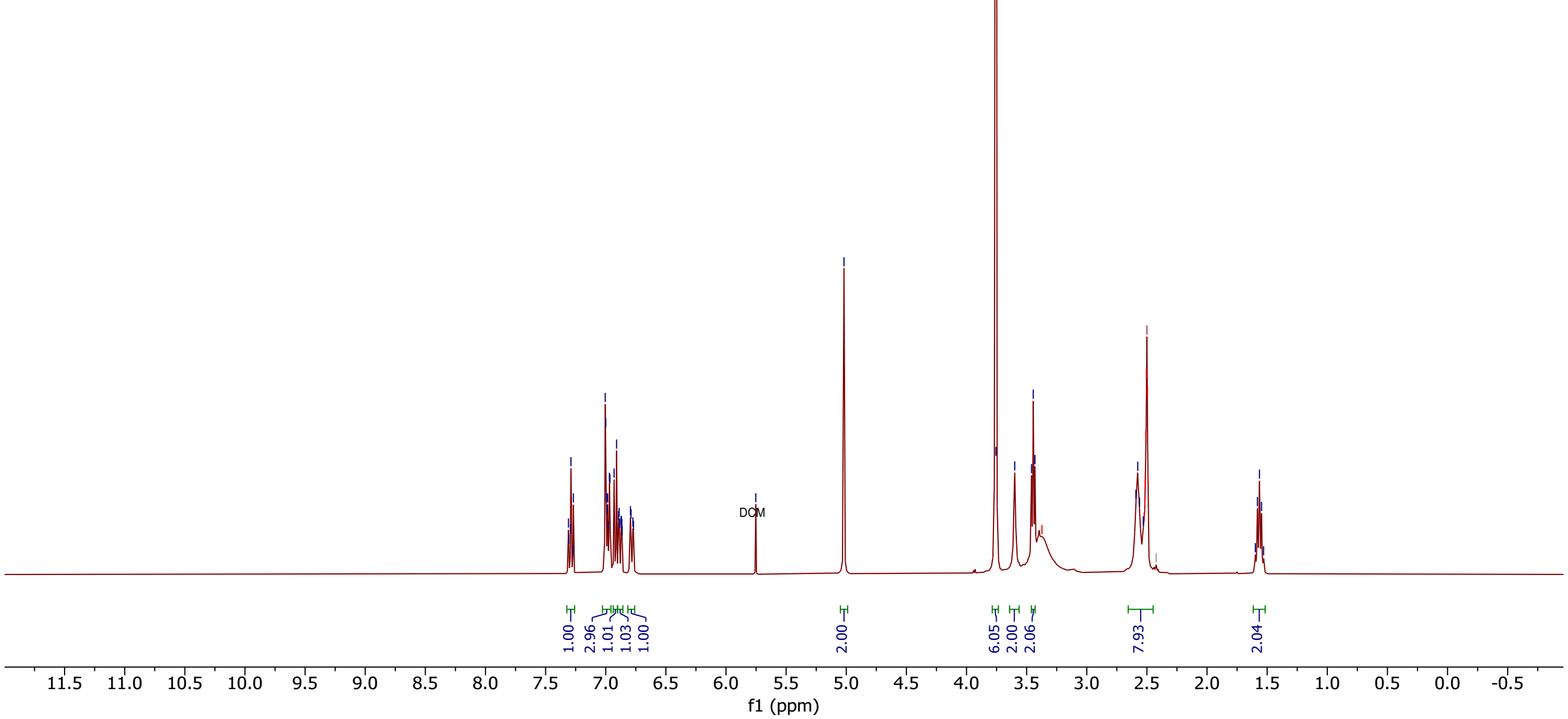

OSM3-MZ-1137.11.1.1r

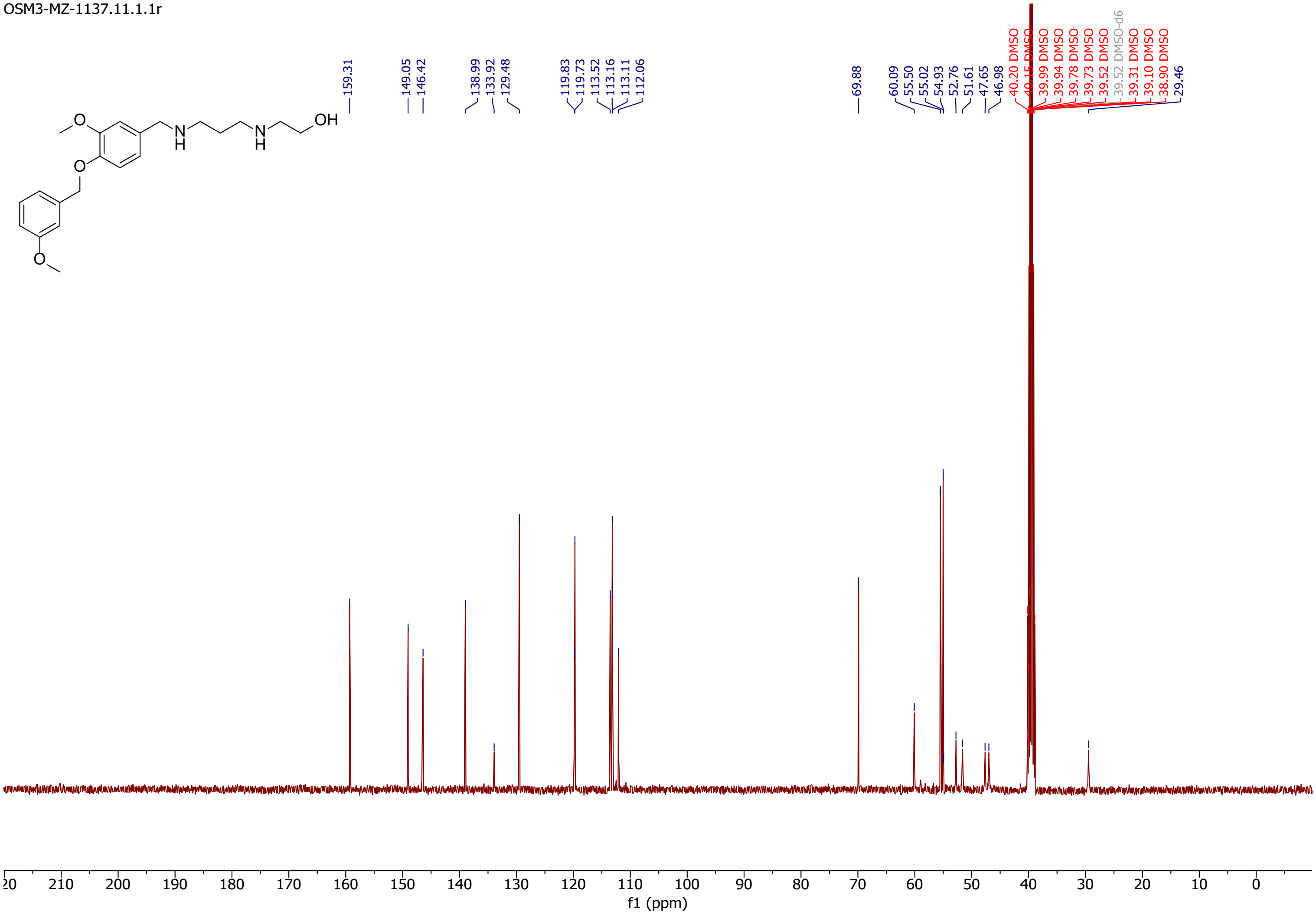

2-((3-((3-Methoxy-4-((3-methybenzyl)oxy)benzyl)amino)propyl)amino)ethan-1-ol (***2k***).

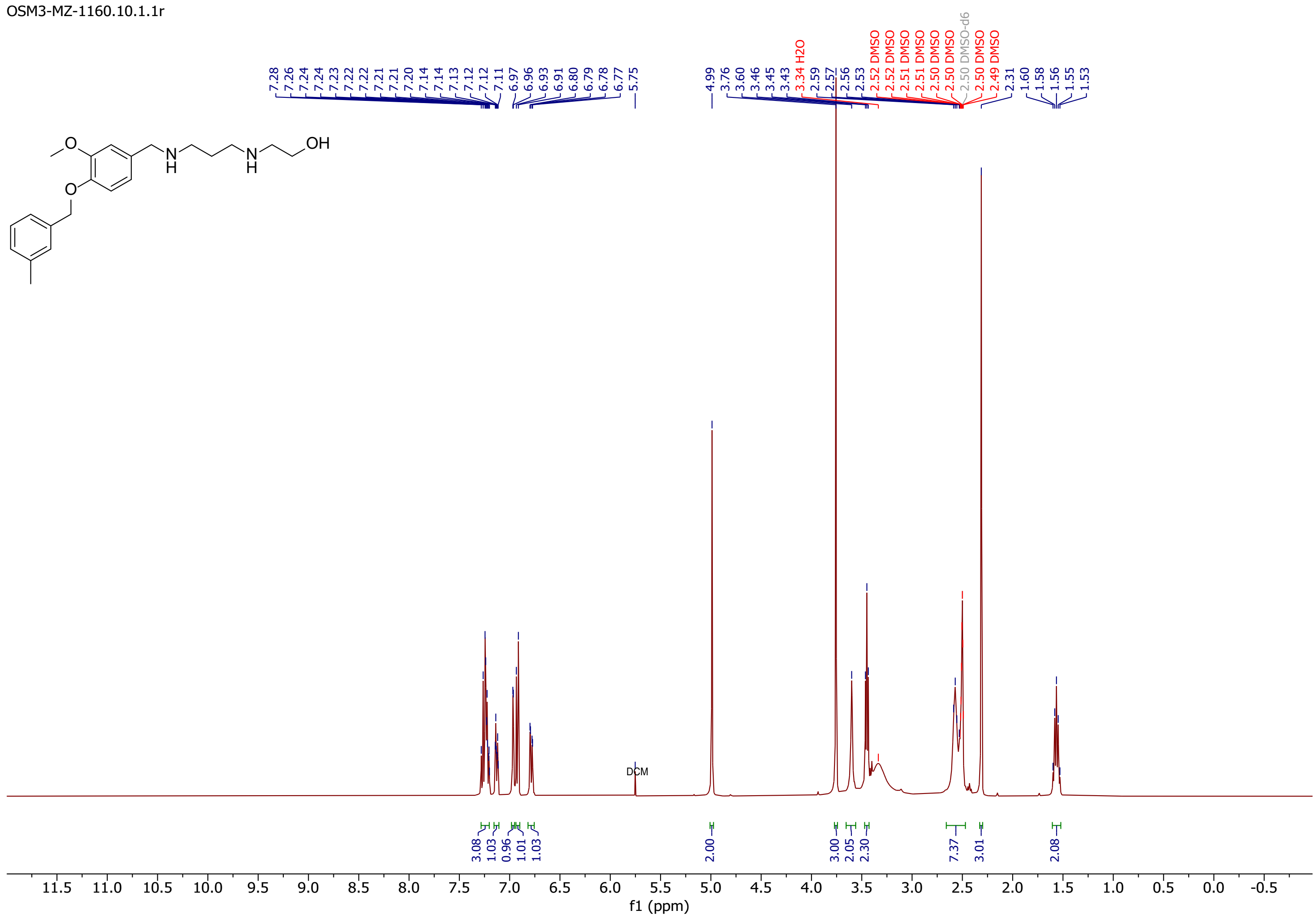

OSM3-MZ-1160.11.1.1r

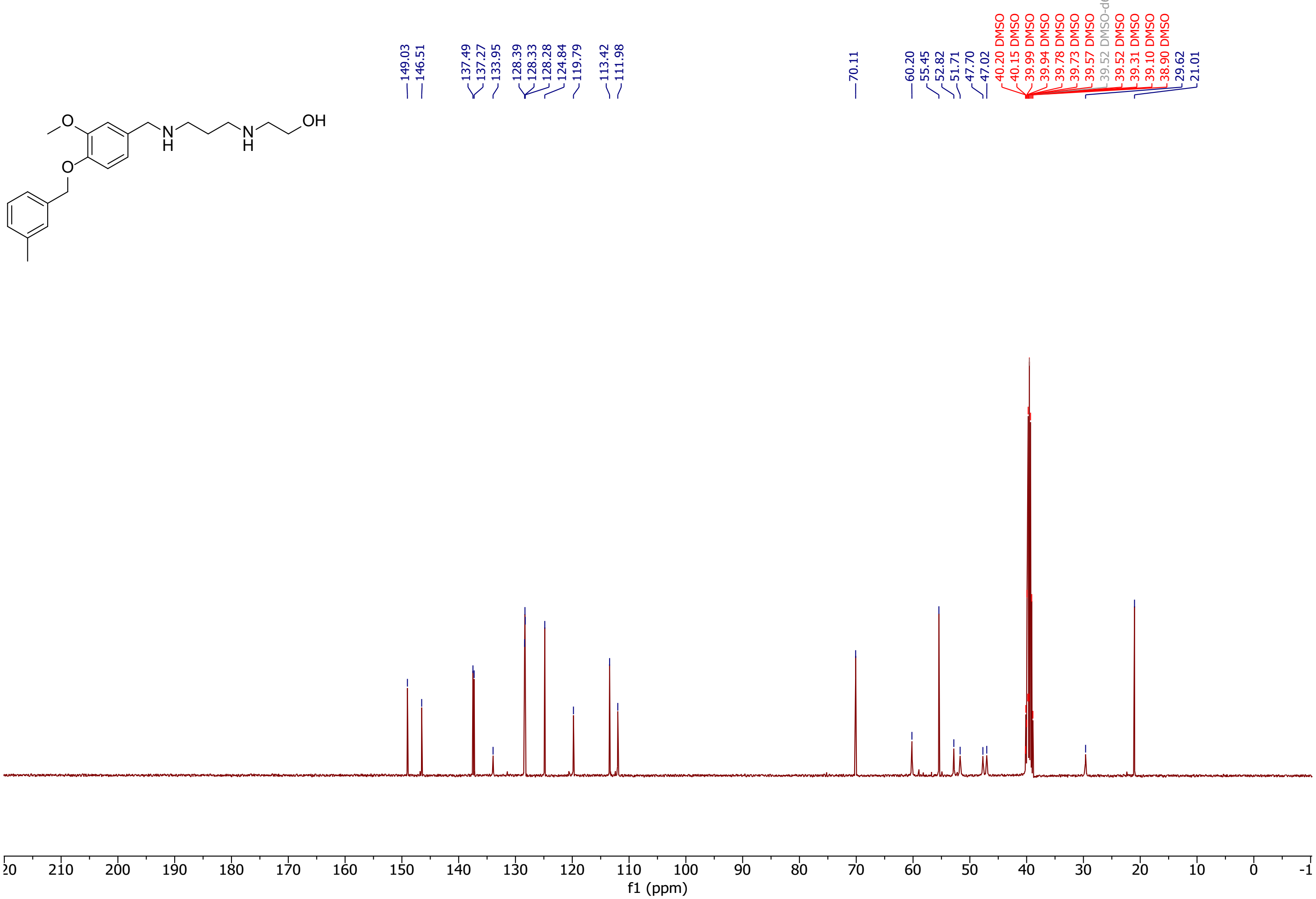

2-((3-((3-Methoxy-4-((3-trifluoromethylbenzyl)oxy)benzyl)amino)propyl)amino)- ethan-1-ol (***2l***).

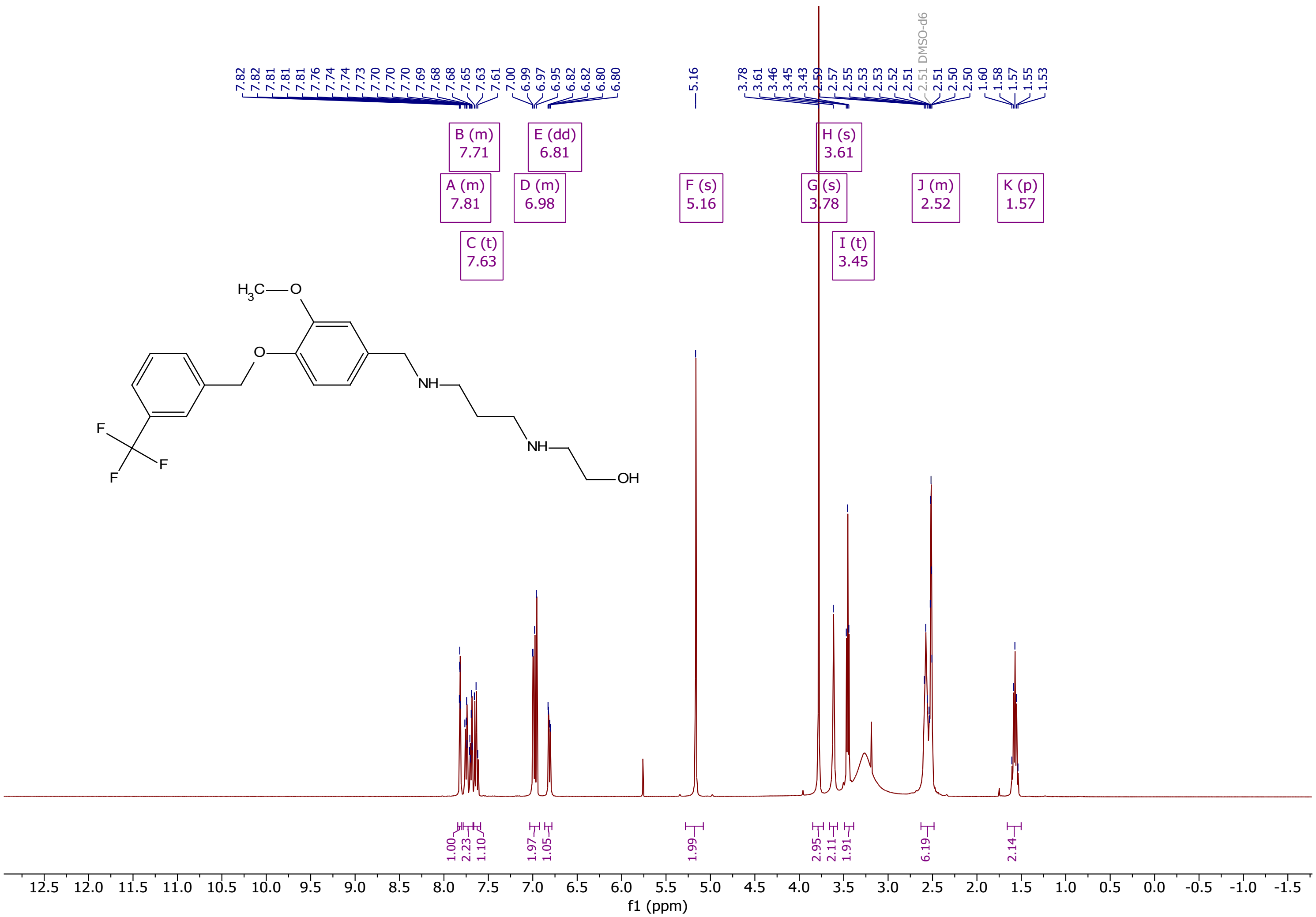

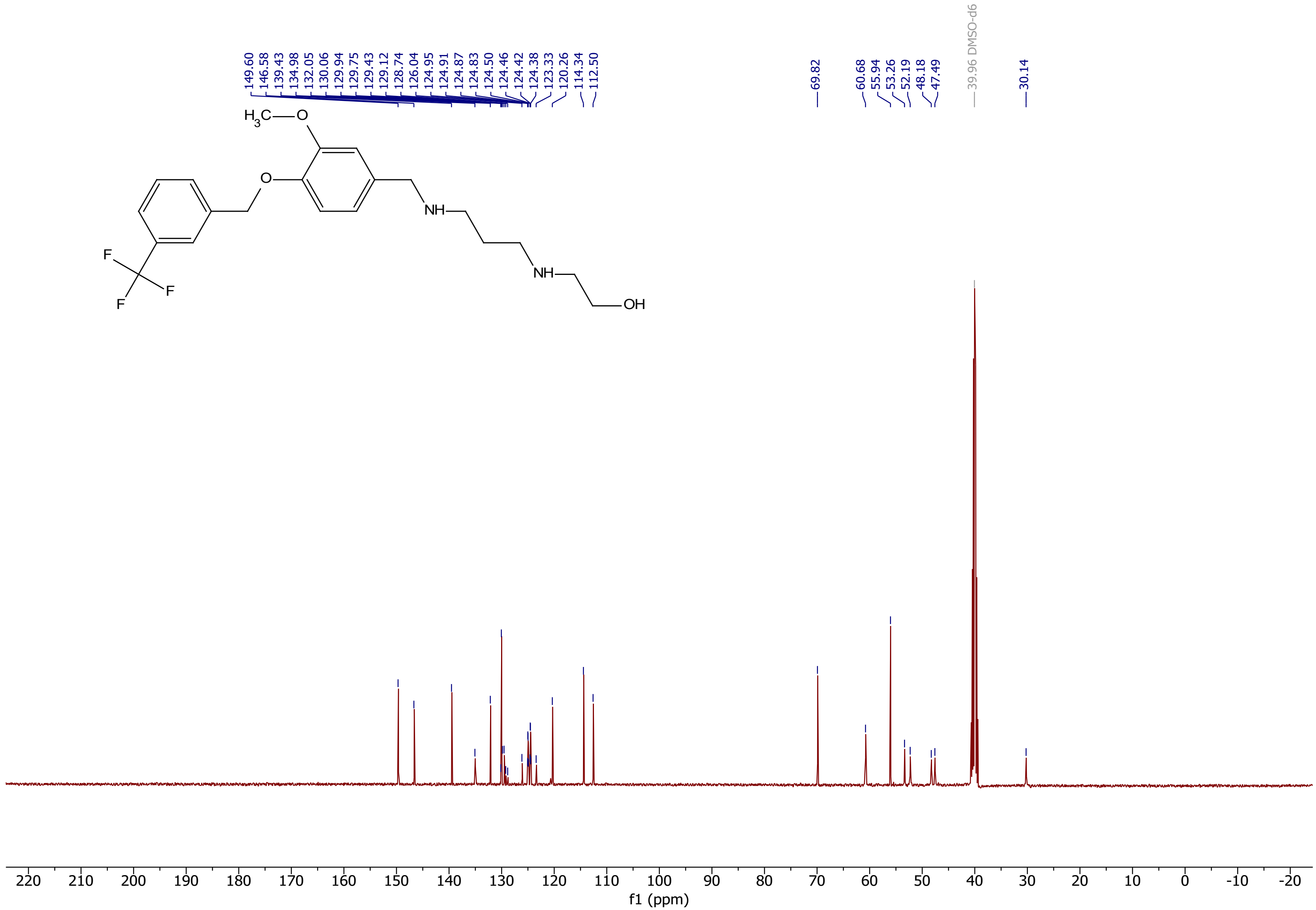
149.60
146.58
139.43
134.98
132.05
130.06
129.94
129.75
129.43
129.12
128.74
126.04
124.95
124.91
124.87
124.83
124.50
124.46
124.42
124.38
123.33
120.26
114.34
112.50
69.82
60.68
55.94
53.26
52.19
48.18
47.49
39.96 DMSO-d6
30.14
H3C
O
O
NH
NH
OH
F
F
F
220 210 200 190 180 170 160 150 140 130 120 110 100 90 80 70 60 50 40 30 20 10 0 -10 -20
f1 (ppm)

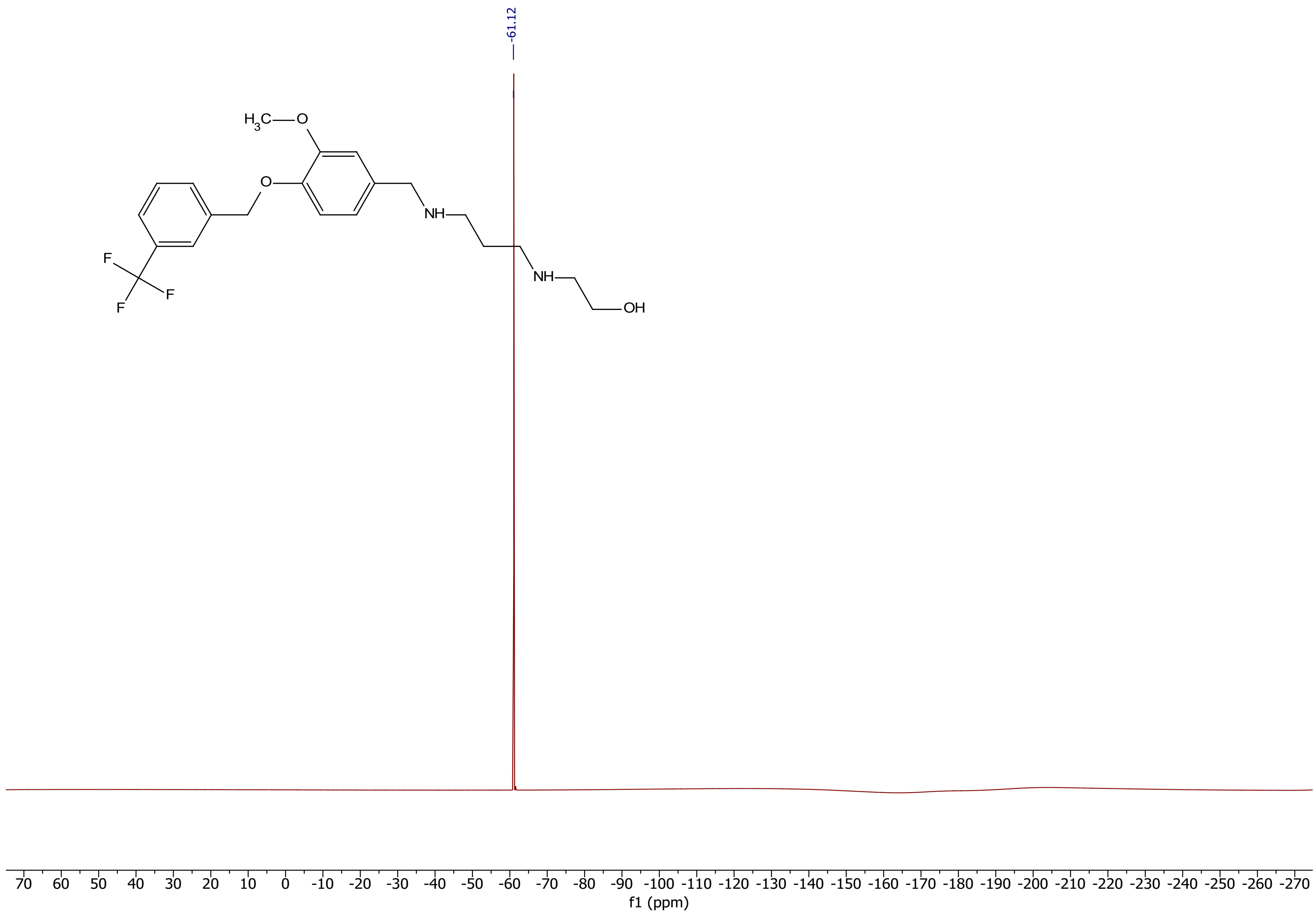
-61.12
H3C
O
O
NH
NH
OH
F
F
F
70 60 50 40 30 20 10 0 -10 -20 -30 -40 -50 -60 -70 -80 -90 -100 -110 -120 -130 -140 -150 -160 -170 -180 -190 -200 -210 -220 -230 -240 -250 -260 -270
f1 (ppm)

*2-((3-((4-((4-(tert-Butyl)benzyl)oxy)-3-methoxybenzyl)amino)propyl)amino)eth- an-1-ol (**2n**).*

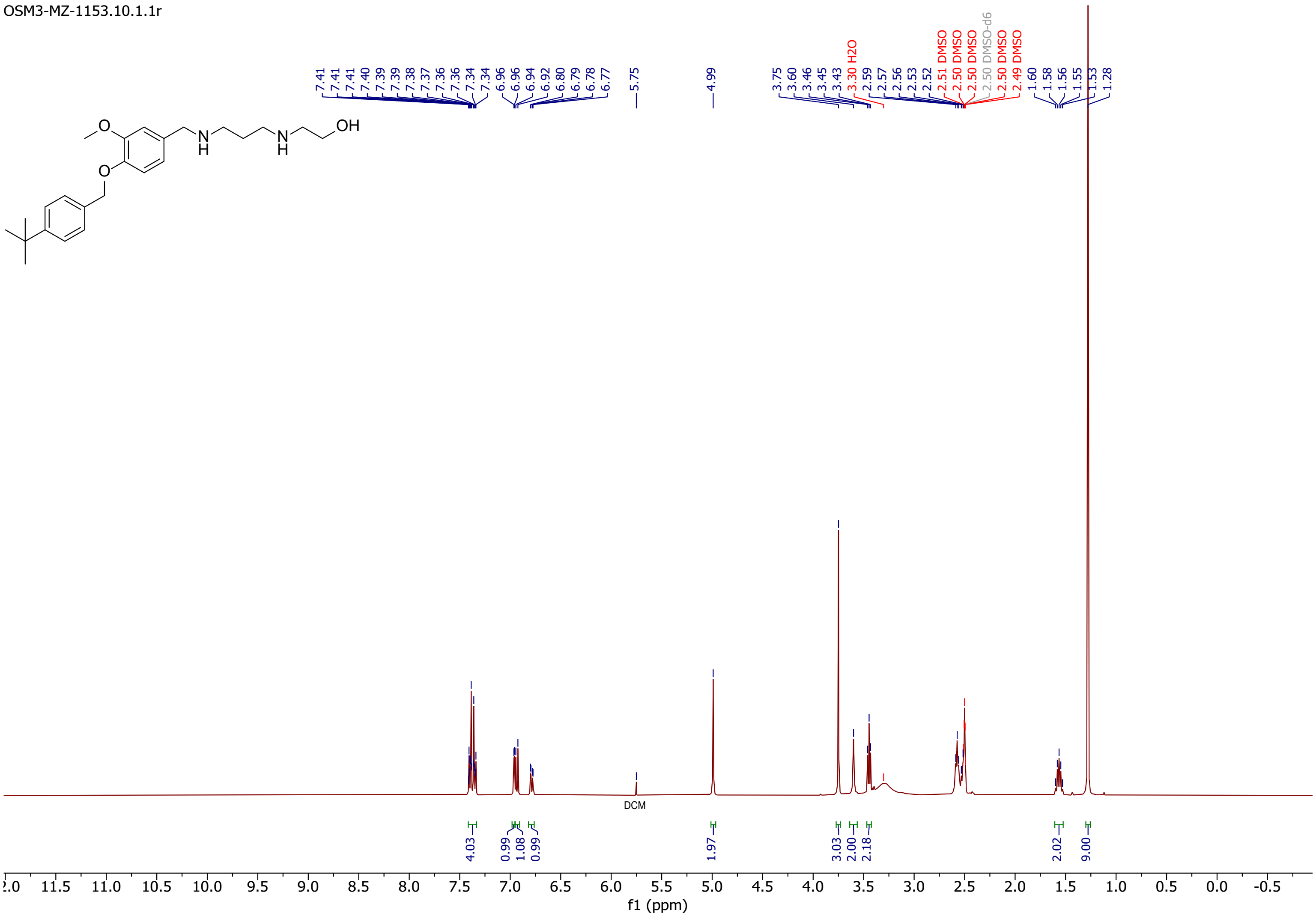

OSM3-MZ-1153.11.1.1r

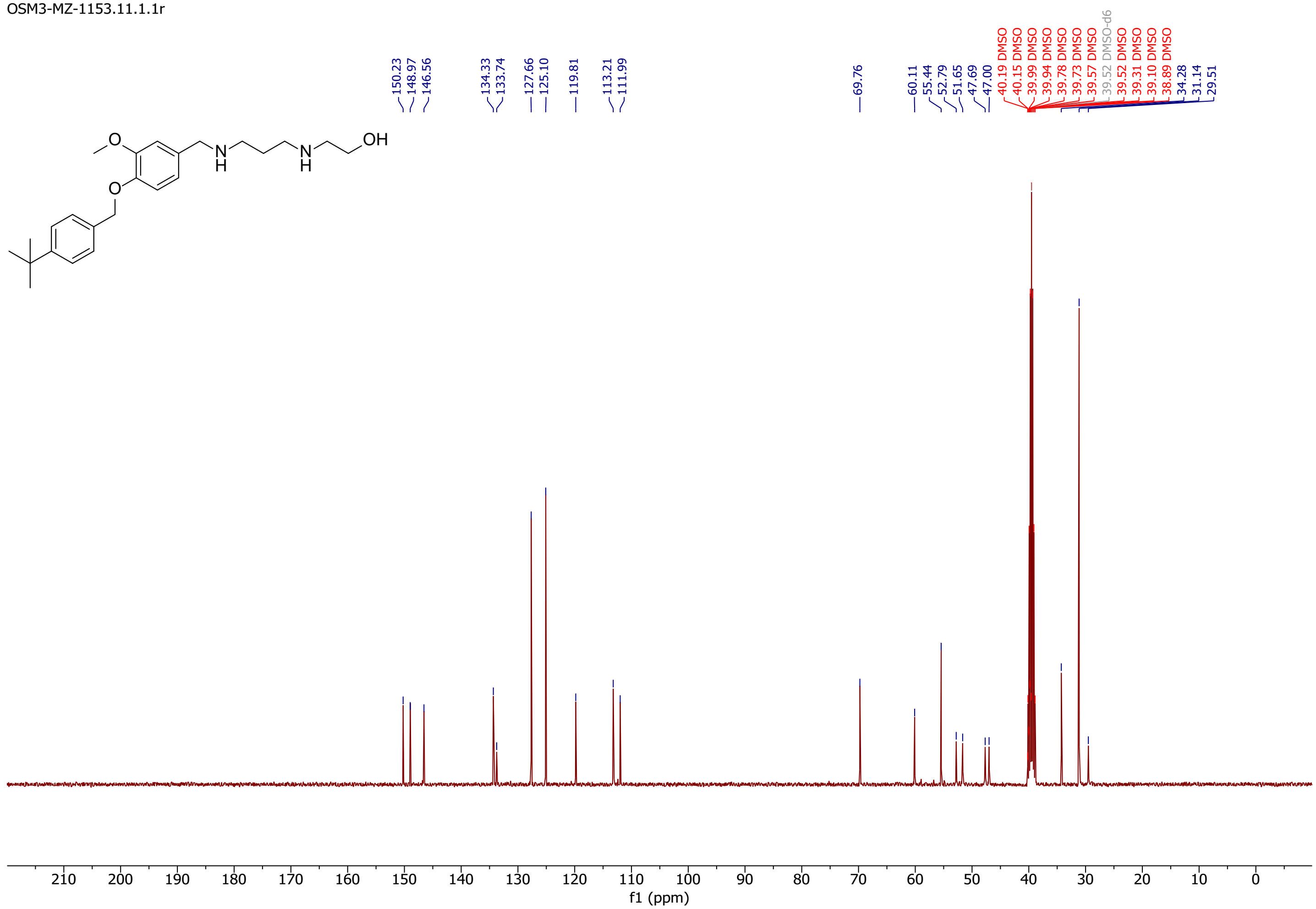

*2-((3-((4-([1,1'-Biphenyl]-4-ylmethoxy)-3-methoxybenzyl)amino)propyl)amino)ethan-1-ol (**2o**).*

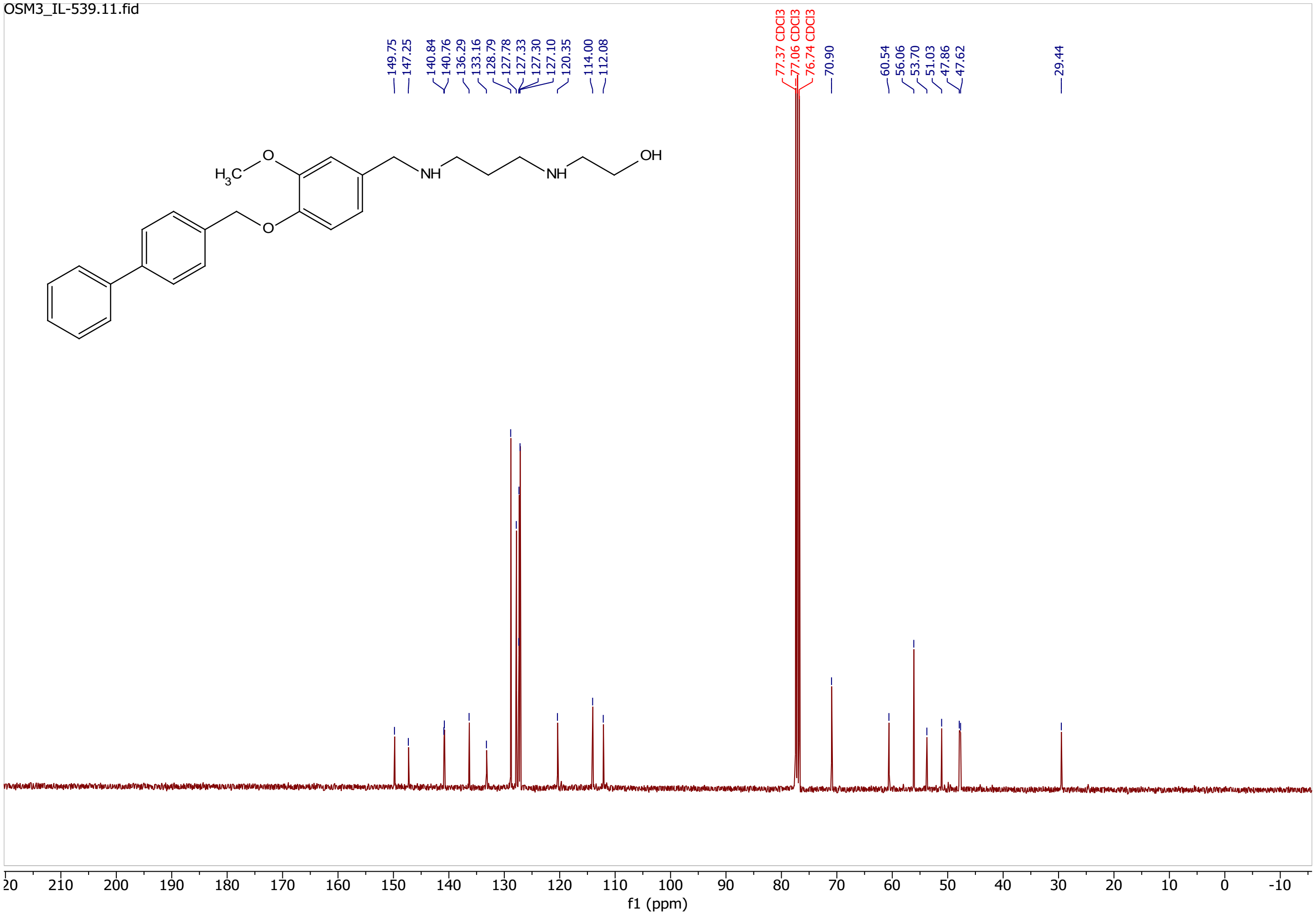

2-((3-((4-((2,6-Dichlorobenzyl)oxy)-3-methoxybenzyl)amino)propyl)amino)ethan-1-ol (***2p***).

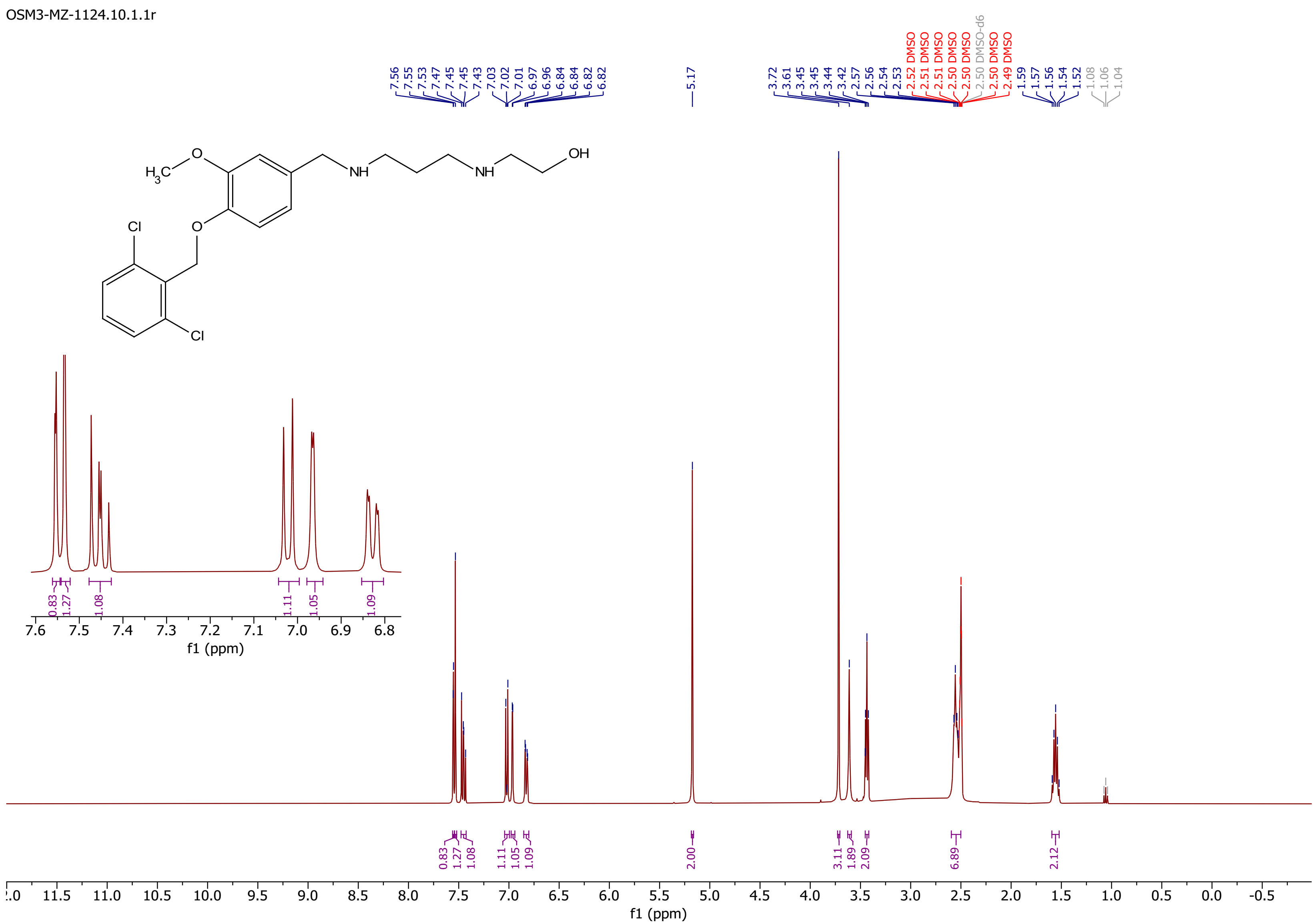

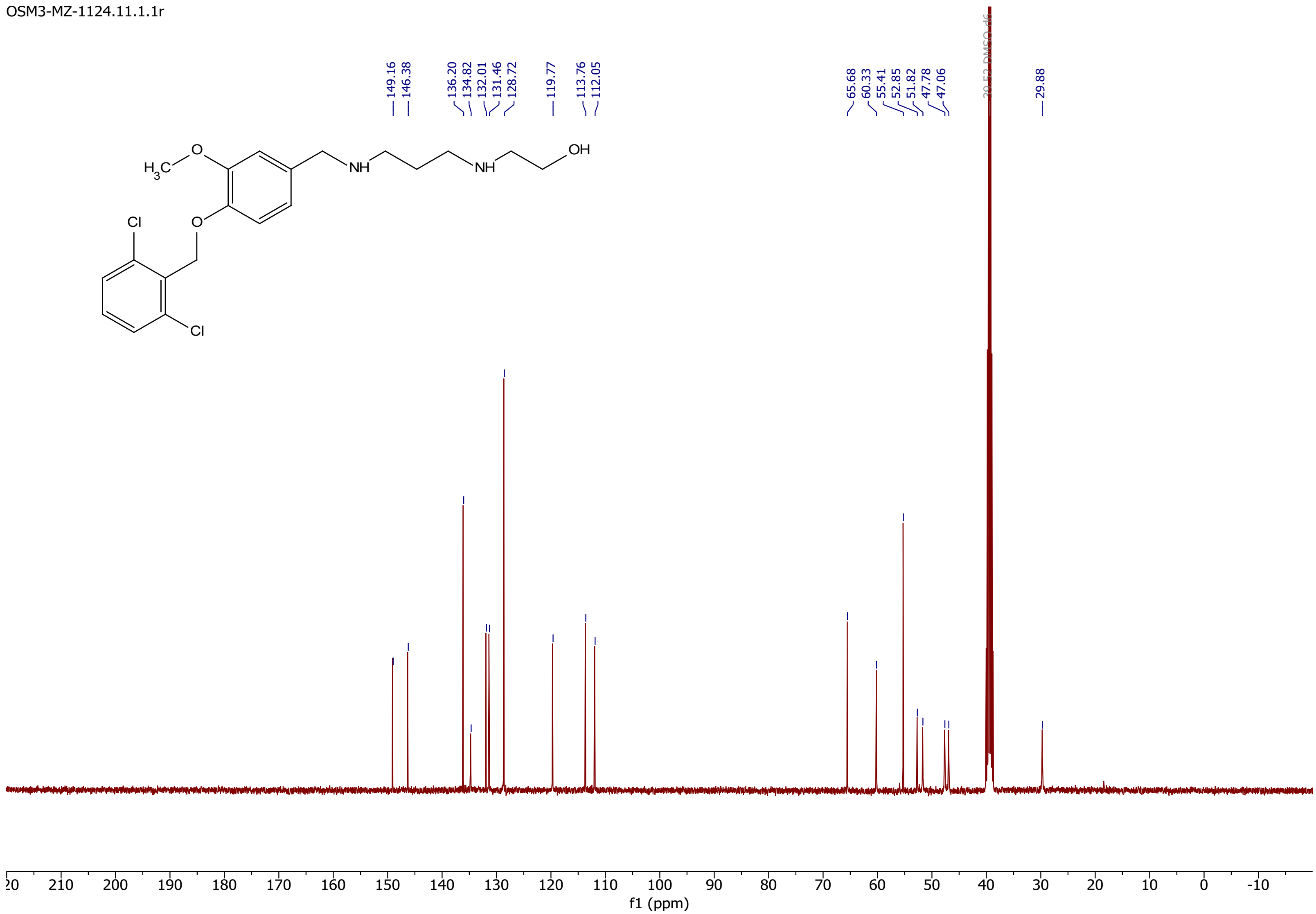
OSM3-MZ-1124.11.1.1r
149.16
146.38
136.20
134.82
132.01
131.46
128.72
119.77
113.76
112.05
65.68
60.33
55.41
52.85
51.82
47.78
47.06
29.88
H3C
O
NH
NH
OH
Cl
O
Cl
20 210 200 190 180 170 160 150 140 130 120 110 100 90 80 70 60 50 40 30 20 10 0 -10
f1 (ppm)

2-((3-((4-((3,4-Dichlorobenzyl)oxy)-3-methoxybenzyl)amino)propyl)amino)ethan-1-ol (***2q***)**.**

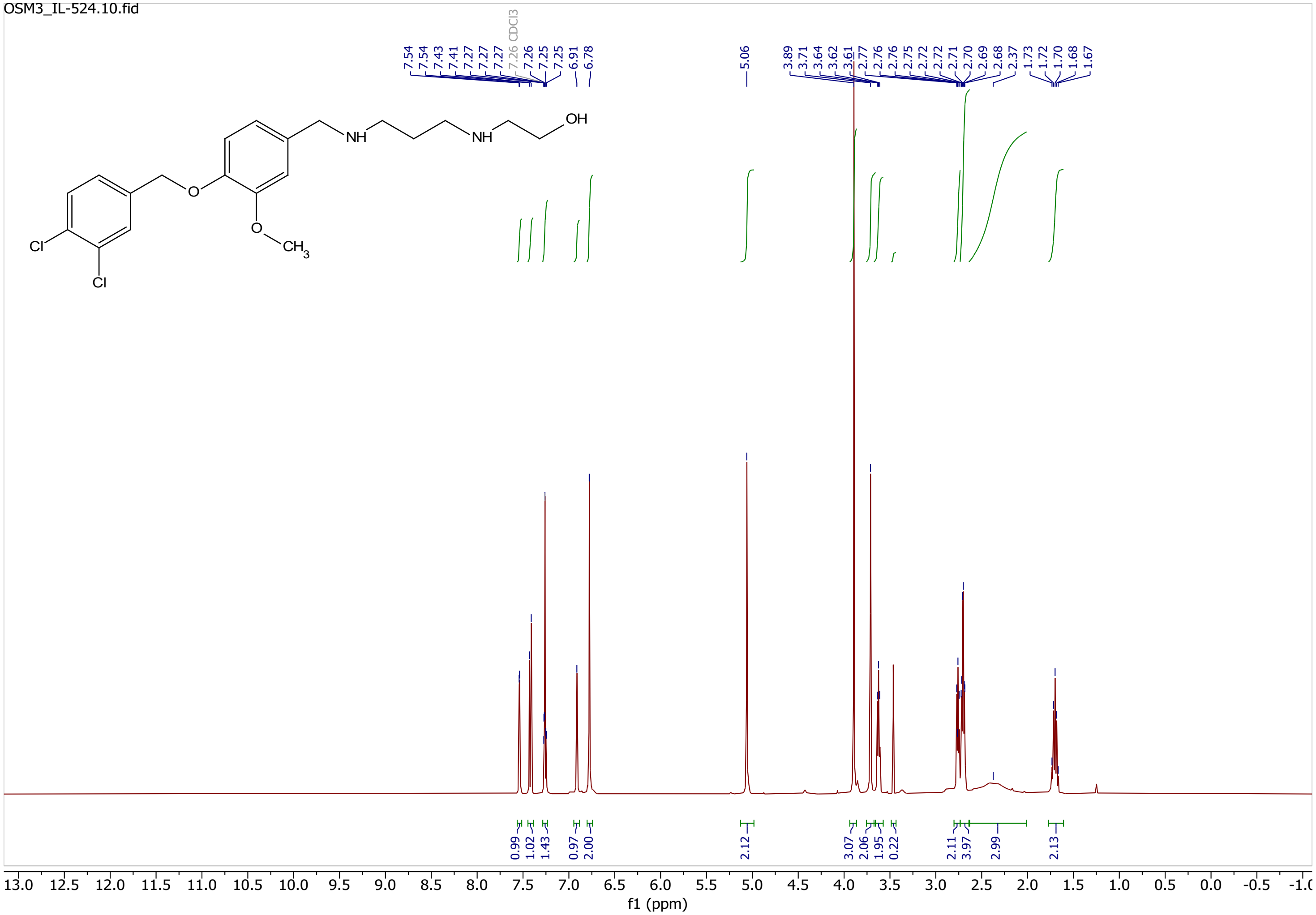

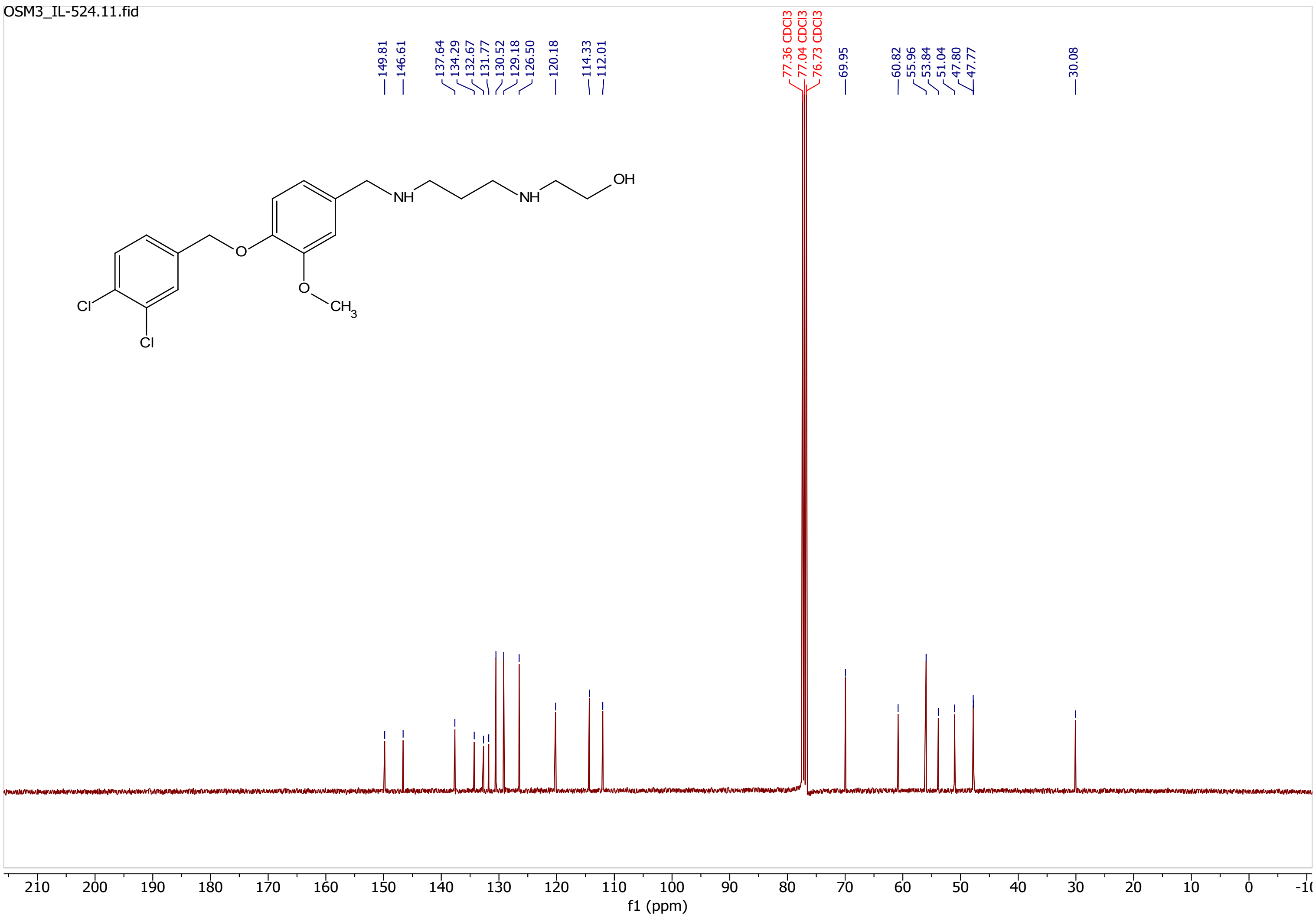
OSM3_IL-524.11.fid
149.81
146.61
137.64
134.29
132.67
131.77
130.52
129.18
126.50
120.18
114.33
112.01
77.36 CDCl3
77.04 CDCl3
76.73 CDCl3
69.95
60.82
55.96
53.84
51.04
47.80
47.77
30.08
f1 (ppm)

2-((3-((4-((4-Chloro-3-(trifluoromethyl)benzyl)oxy)-3-methoxybenzyl)amino)propyl)amino)ethan-1-ol (***2r***).

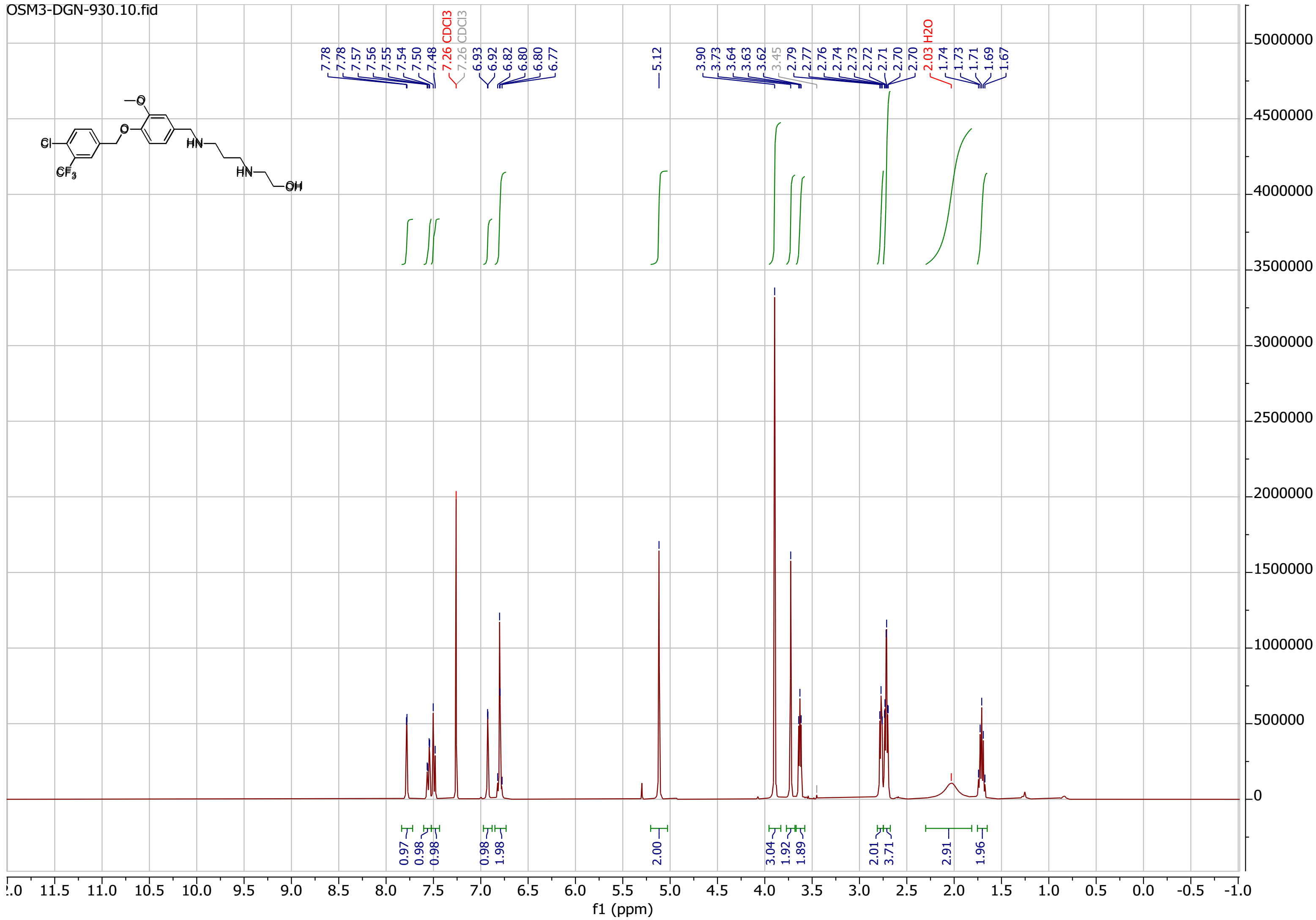

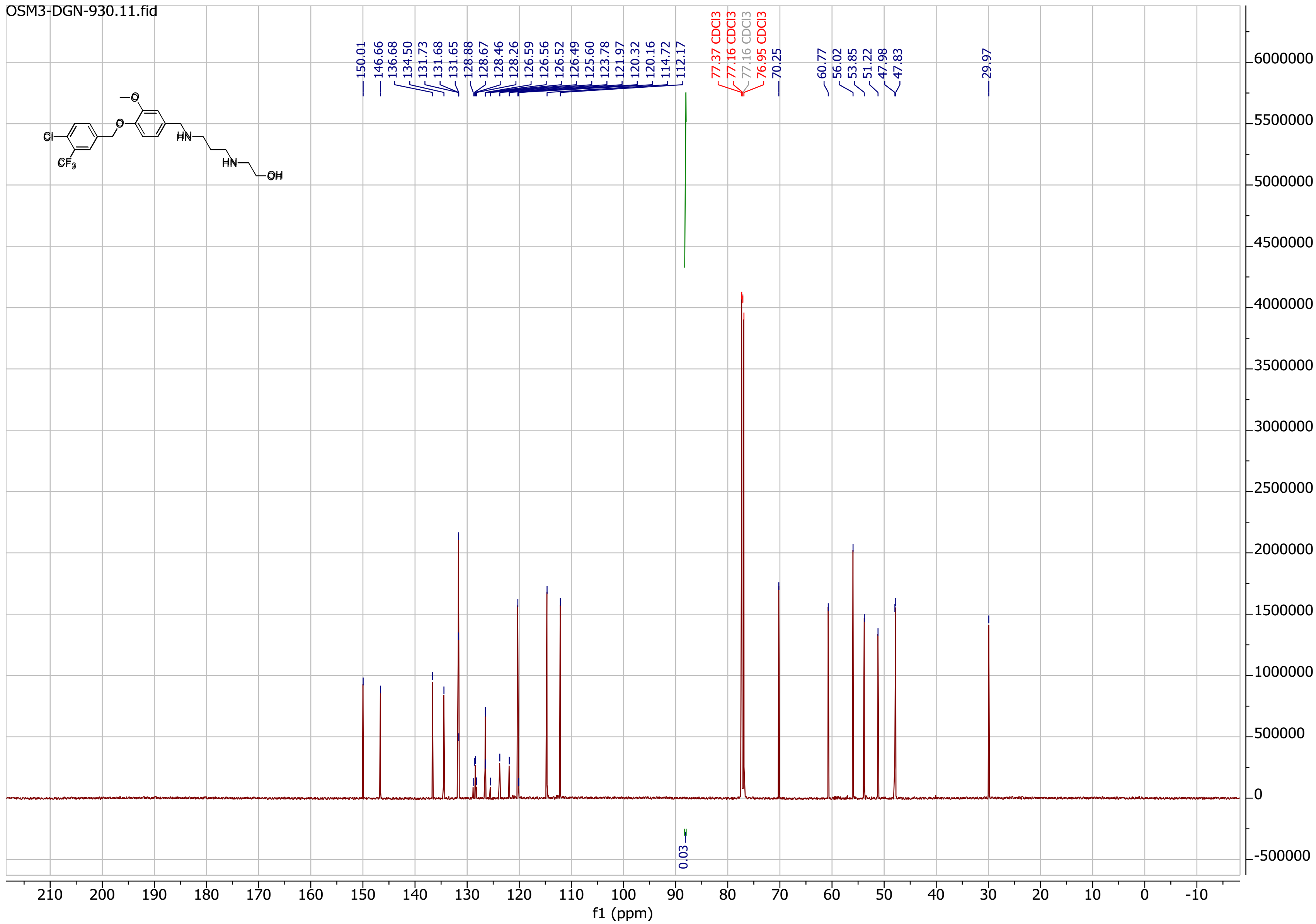
OSM3-DGN-930.11.fid
150.01
146.66
136.68
134.50
131.73
131.68
131.65
128.88
128.67
128.46
128.26
126.59
126.56
126.52
126.49
125.60
123.78
121.97
120.32
120.16
114.72
112.17
77.37 CDCl3
77.16 CDCl3
77.16 CDCl3
76.95 CDCl3
70.25
60.77
56.02
53.85
51.22
47.98
47.83
29.97
0.03
f1 (ppm)
Cl
CF3
O
HN
HN
OH

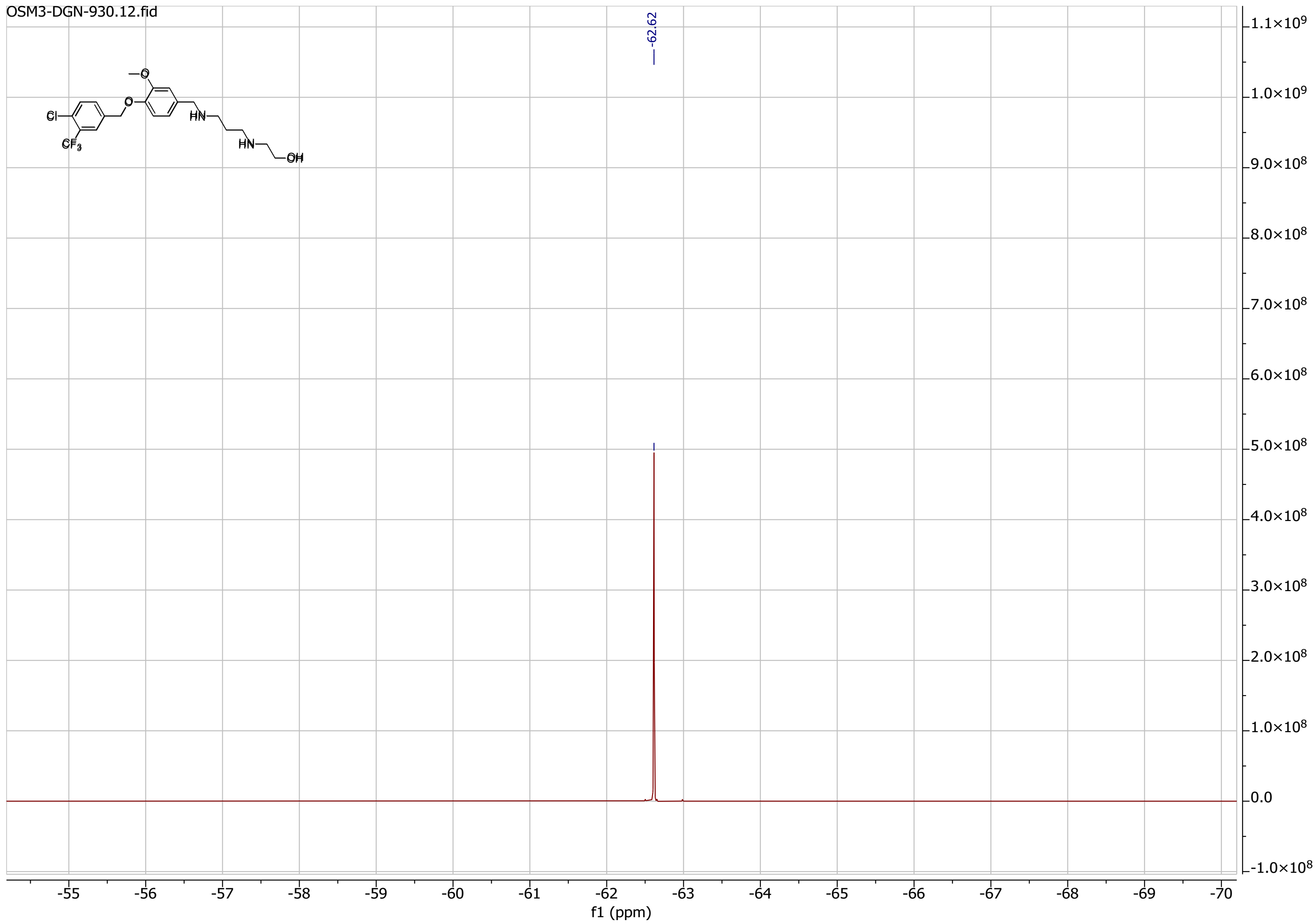
OSM3-DGN-930.12.fid
-62.62
Cl
CF3
O
O
HN
HN
OH
1.1×10^9
1.0×10^9
9.0×10^8
8.0×10^8
7.0×10^8
6.0×10^8
5.0×10^8
4.0×10^8
3.0×10^8
2.0×10^8
1.0×10^8
0.0
-1.0×10^8
-55
-56
-57
-58
-59
-60
-61
-62
-63
-64
-65
-66
-67
-68
-69
-70
f1 (ppm)

2-((3-((4-((2,3-Dichlorobenzyl)oxy)-3-methoxybenzyl)amino)propyl)amino)ethan-1-ol (***2s***).

OSM3-MZ-1139.11.1.1r

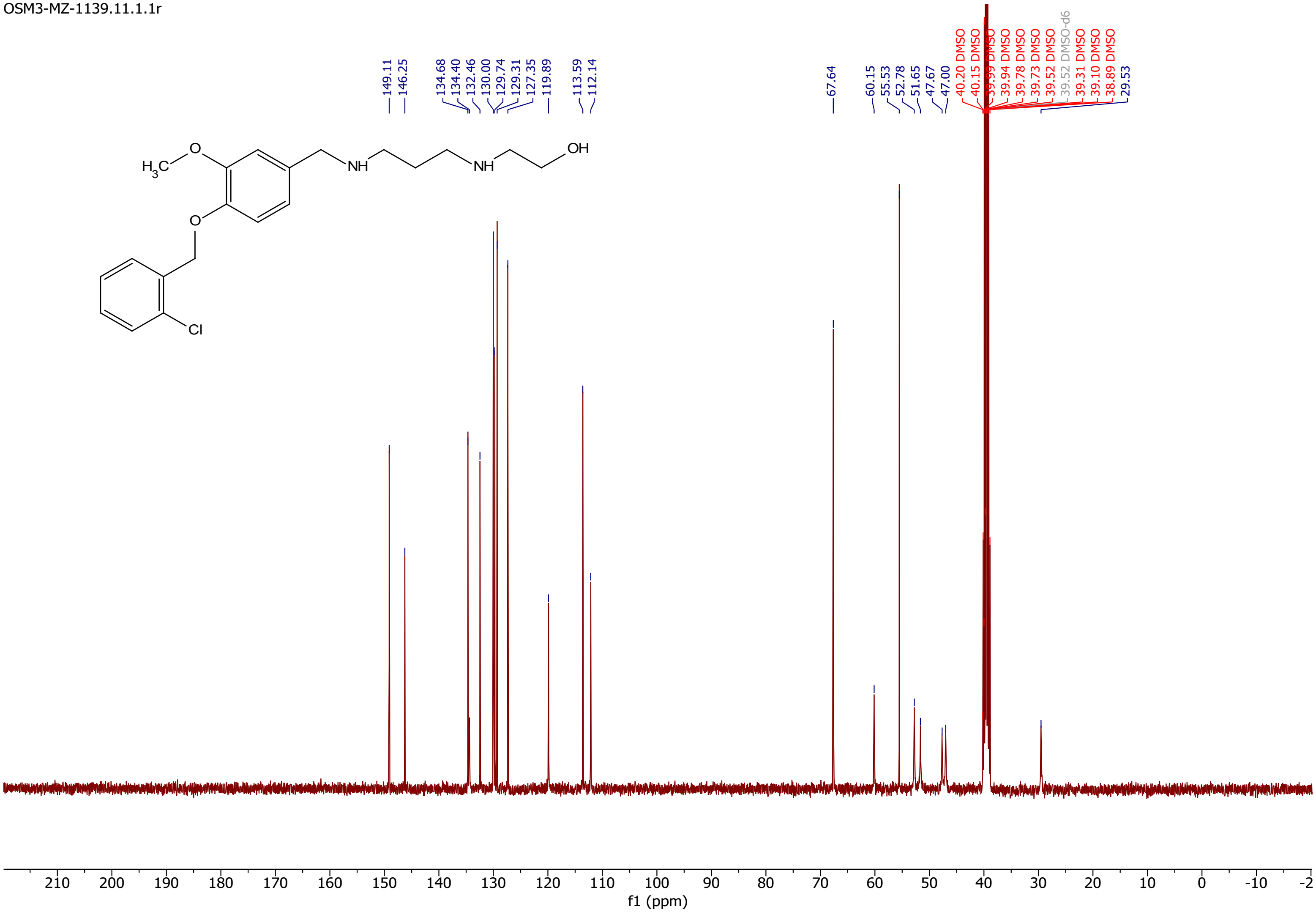

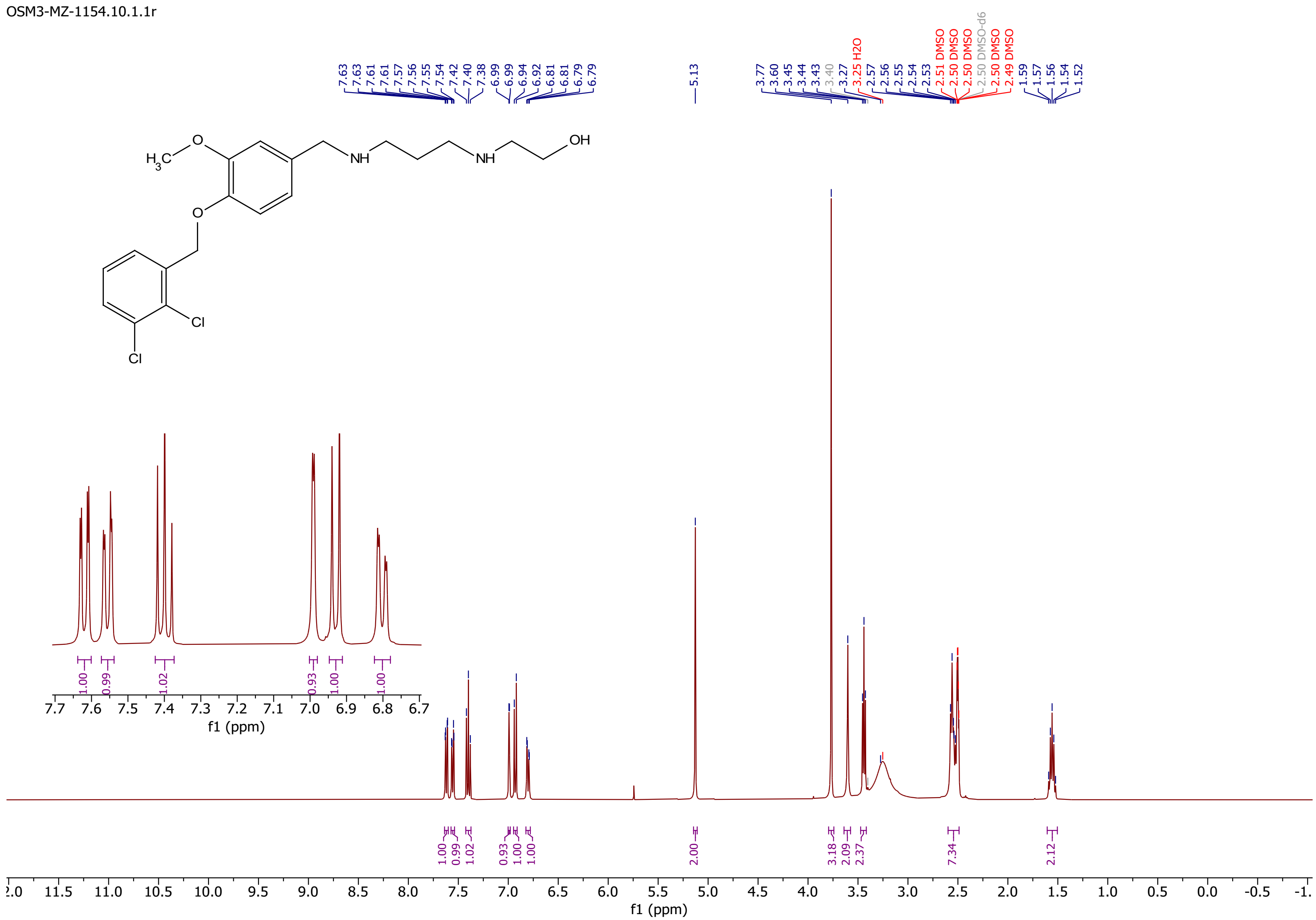
OSM3-MZ-1154.10.1.1r
7.63
7.63
7.61
7.61
7.57
7.56
7.55
7.54
7.42
7.40
7.38
6.99
6.99
6.94
6.92
6.81
6.79
6.79
5.13
3.77
3.60
3.45
3.44
3.43
3.40
3.27
3.25 H2O
2.57
2.56
2.55
2.54
2.53
2.51 DMSO
2.50 DMSO
2.50 DMSO
2.50 DMSO-d6
2.50 DMSO
2.49 DMSO
1.59
1.57
1.56
1.54
1.52
H3C
O
O
NH
NH
OH
Cl
Cl
1.00
0.99
1.02
0.93
1.00
1.00
7.7
7.6
7.5
7.4
7.3
7.2
7.1
7.0
6.9
6.8
6.7
f1 (ppm)
1.00
0.99
1.02
0.93
1.00
1.00
2.00
3.18
2.09
2.37
7.34
2.12
12.0
11.5
11.0
10.5
10.0
9.5
9.0
8.5
8.0
7.5
7.0
6.5
6.0
5.5
5.0
4.5
4.0
3.5
3.0
2.5
2.0
1.5
1.0
0.5
0.0
-0.5
-1.
f1 (ppm)

2-((3-((4-((2,4-Dichlorobenzyl)oxy)-3-methoxybenzyl)amino)propyl)amino)ethan-1-ol (***2t***).

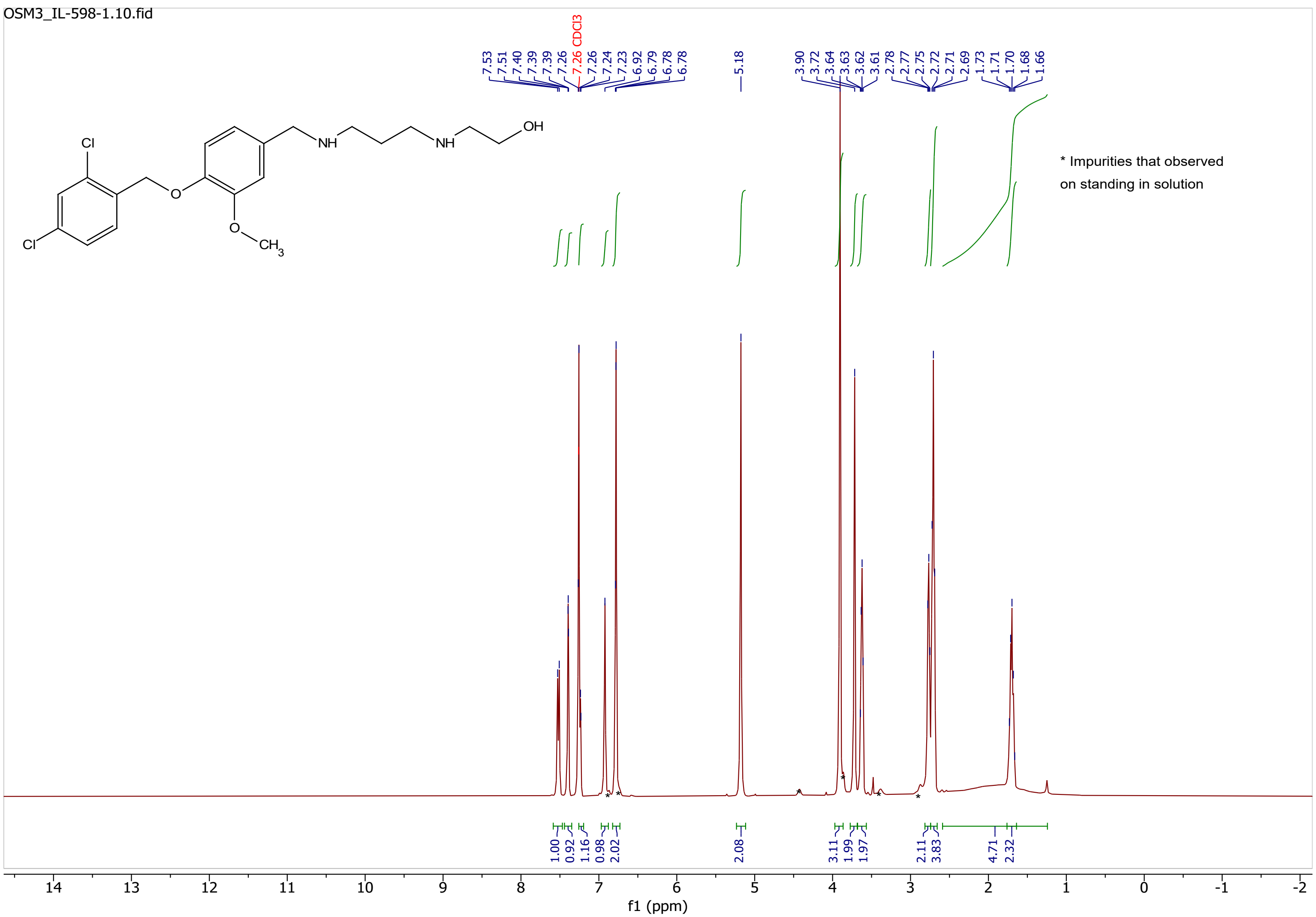

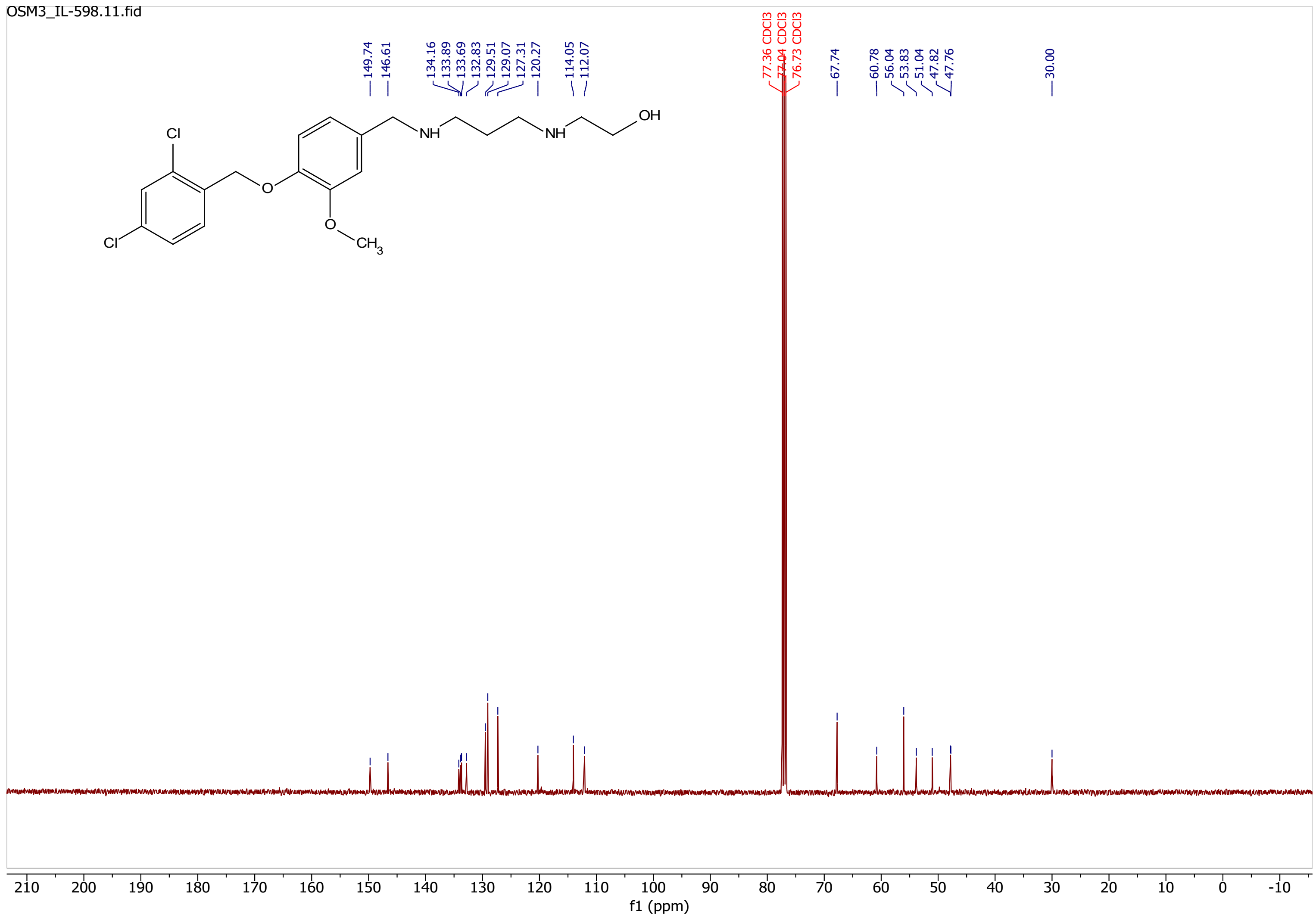
OSM3_IL-598.11.fid
149.74
146.61
134.16
133.89
133.69
132.83
129.51
129.07
127.31
120.27
114.05
112.07
77.36 CDCl3
77.04 CDCl3
76.73 CDCl3
67.74
60.78
56.04
53.83
51.04
47.82
47.76
30.00
Cl
Cl
O
O
CH3
NH
NH
OH
210
200
190
180
170
160
150
140
130
120
110
100
90
80
70
60
50
40
30
20
10
0
-10
f1 (ppm)

2-((3-((4-((3,5-Dichlorobenzyl)oxy)-3-methoxybenzyl)amino)propyl)amino)- ethan-1-ol (***2u***).

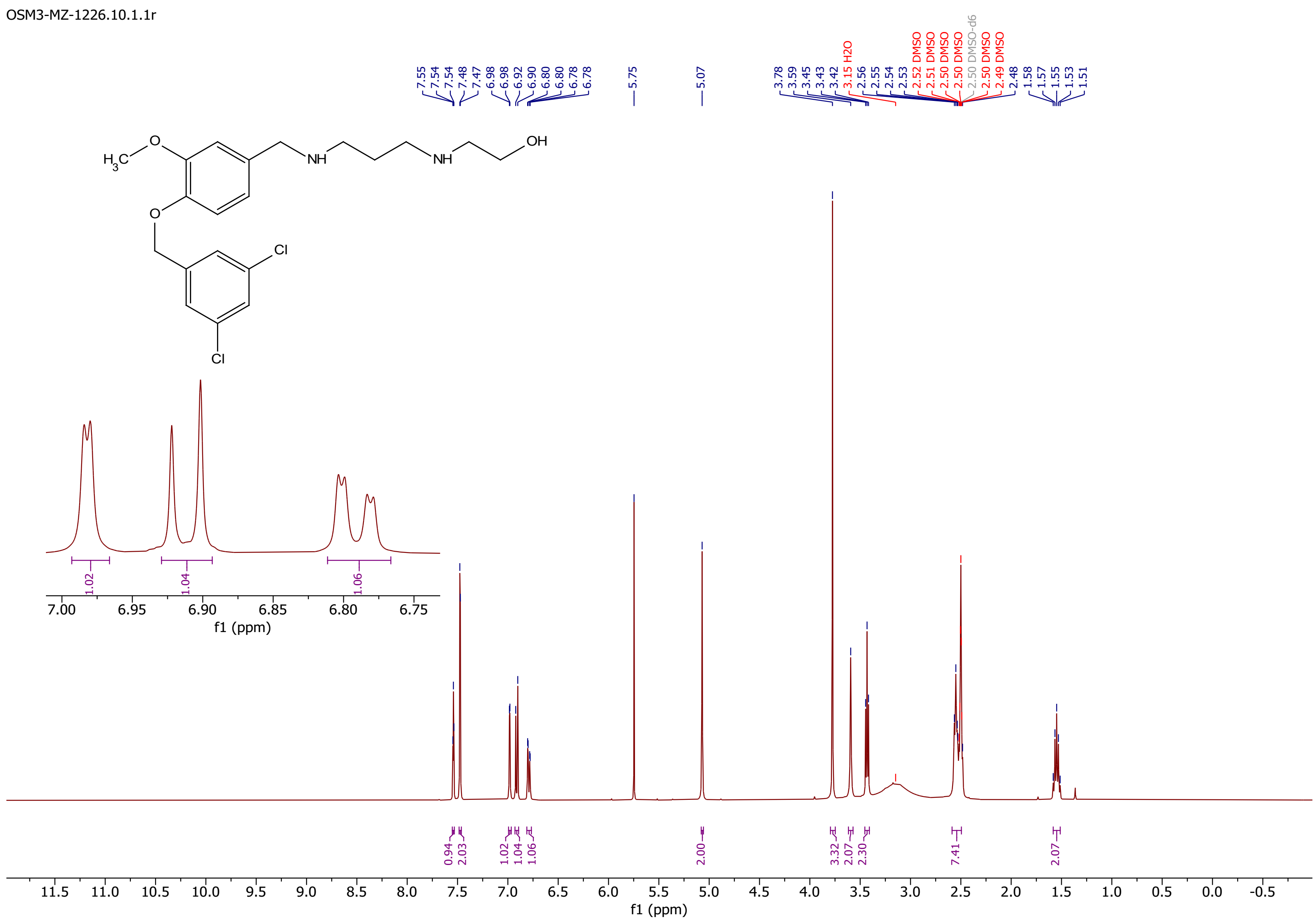

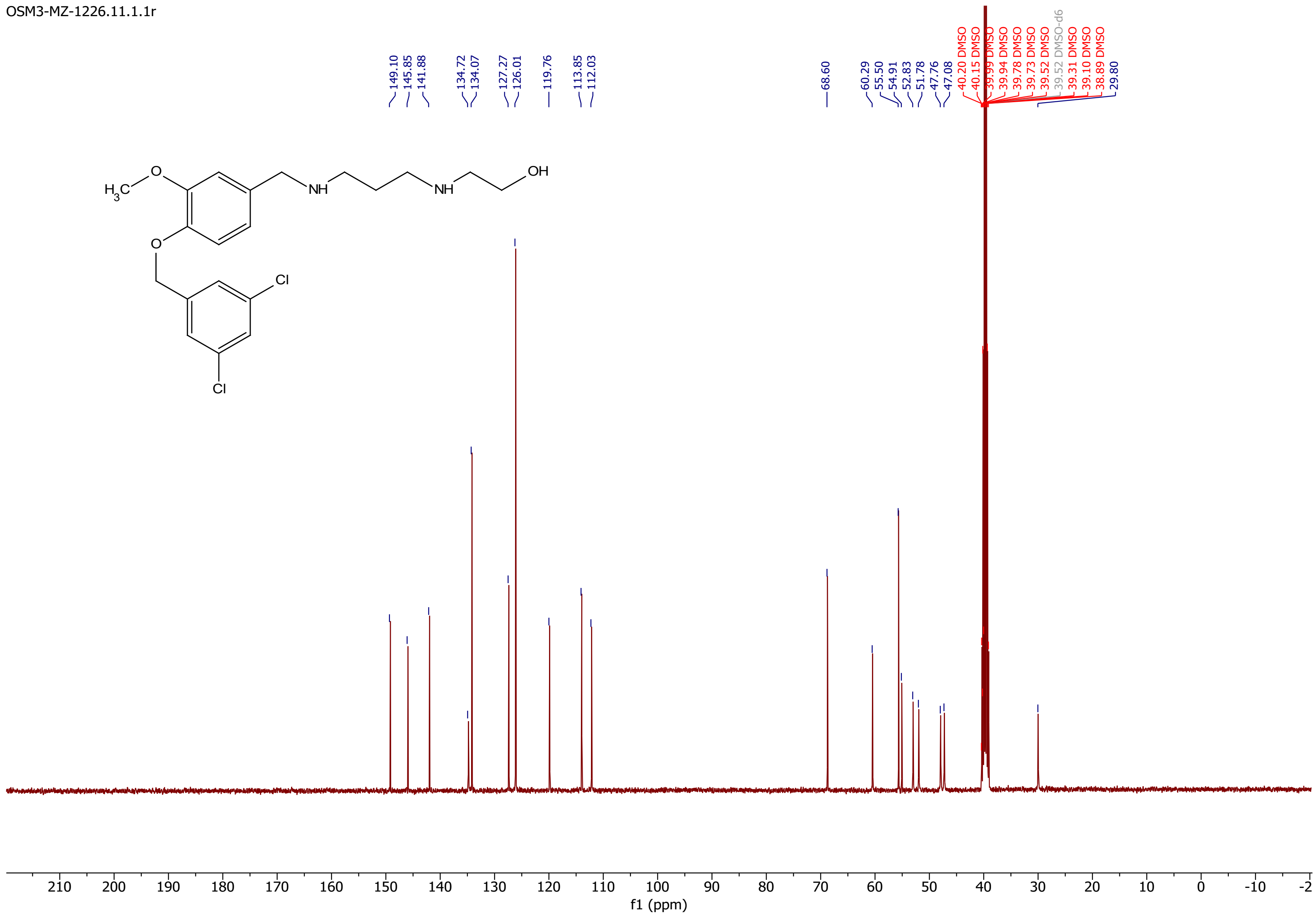
OSM3-MZ-1226.11.1.1r
149.10
145.85
141.88
134.72
134.07
127.27
126.01
119.76
113.85
112.03
68.60
60.29
55.50
54.91
52.83
51.78
47.76
47.08
40.20 DMSO
40.15 DMSO
39.99 DMSO
39.94 DMSO
39.78 DMSO
39.73 DMSO
39.52 DMSO
39.52 DMSO-d6
39.31 DMSO
39.10 DMSO
38.89 DMSO
29.80
H3C
O
NH
NH
OH
Cl
Cl
f1 (ppm)

2-((3-((4-((3,5-Trifluoromethylbenzyl)oxy)-3-methoxybenzyl)amino)propyl)- amino)ethan-1-ol (***2v***).

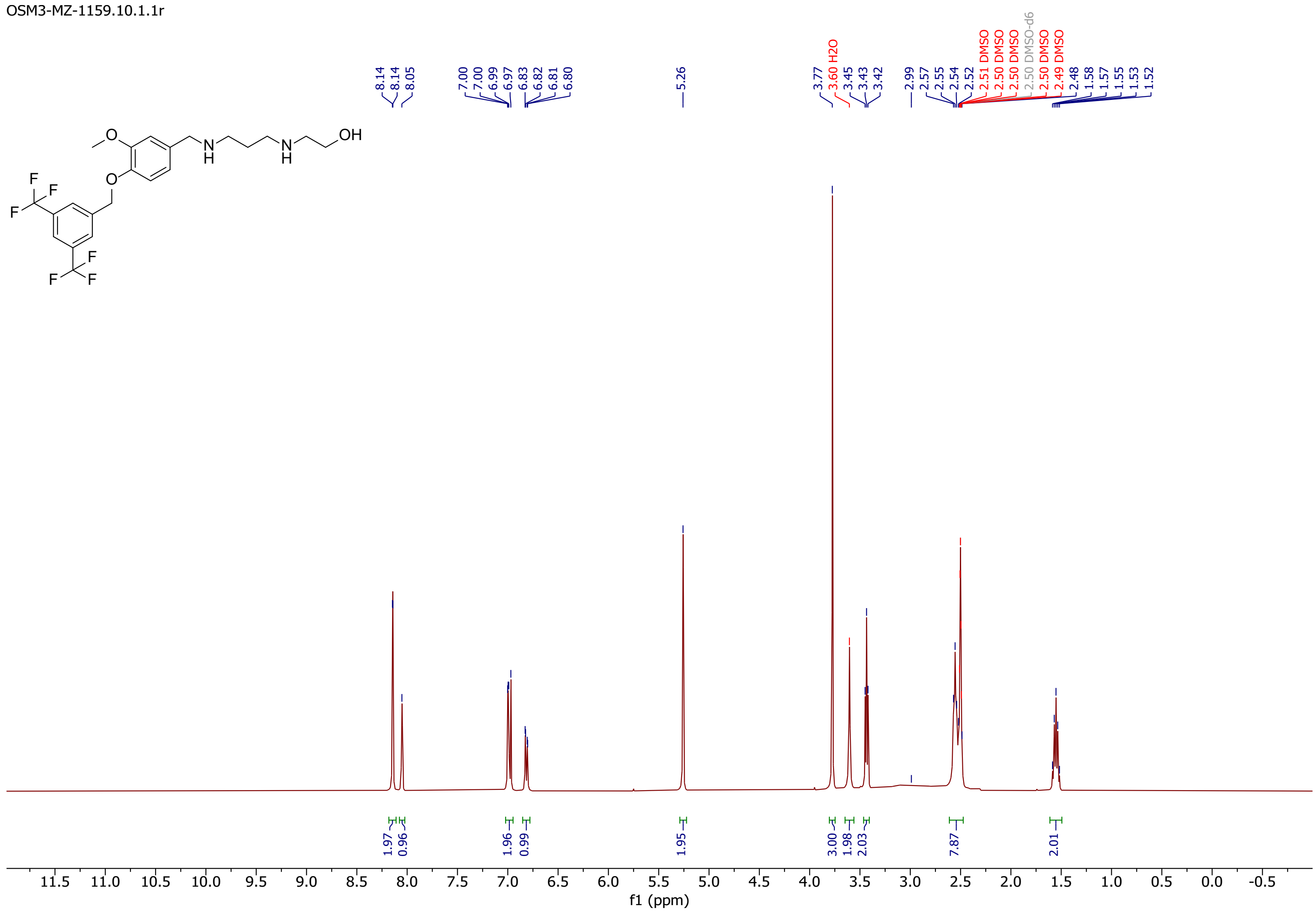

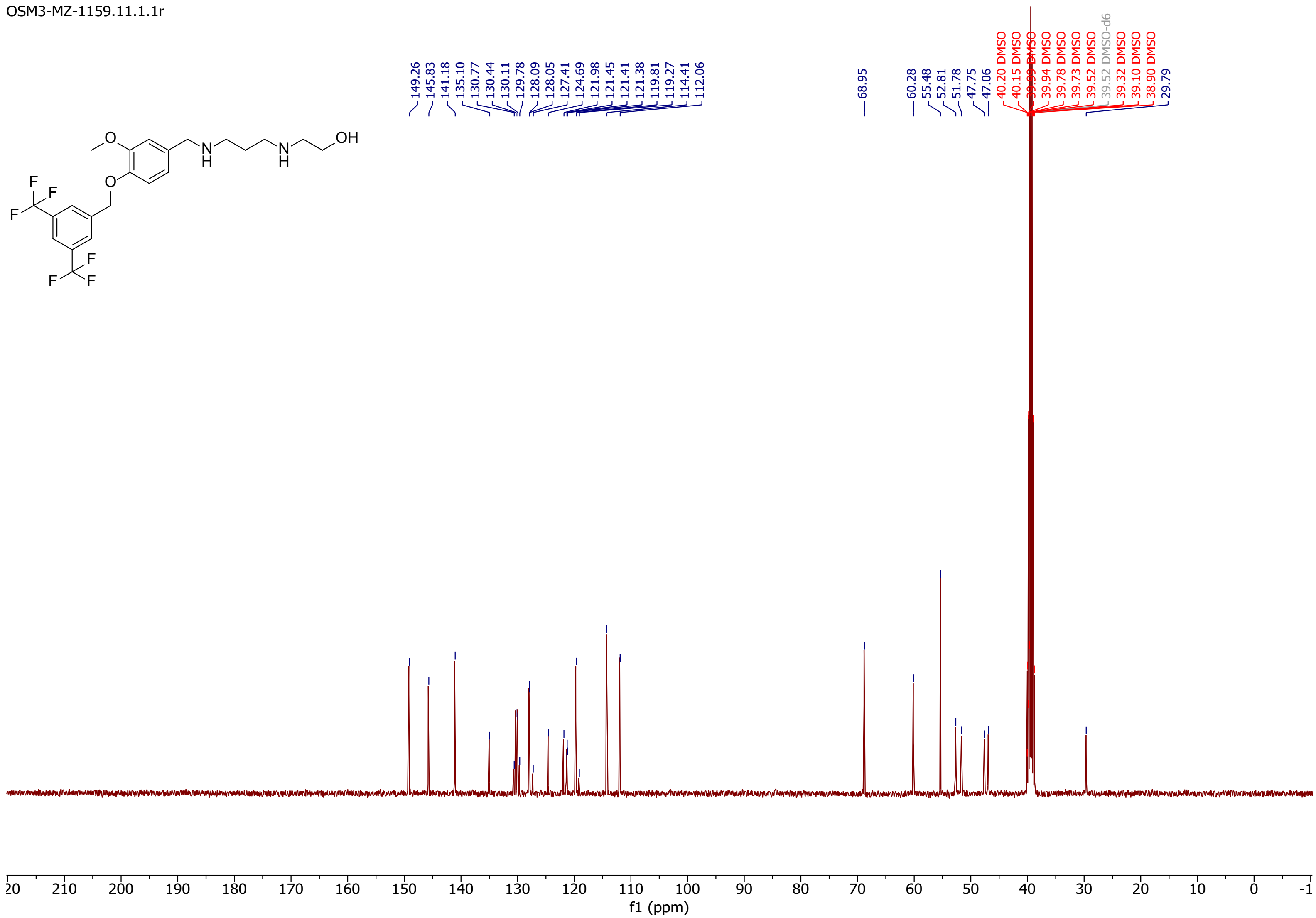
OSM3-MZ-1159.11.1.1r
149.26
145.83
141.18
135.10
130.77
130.44
130.11
129.78
128.09
128.05
127.41
124.69
121.98
121.45
121.41
121.38
119.81
119.27
114.41
112.06
68.95
60.28
55.48
52.81
51.78
47.75
47.06
40.20 DMSO
40.15 DMSO
39.94 DMSO
39.78 DMSO
39.73 DMSO
39.52 DMSO-d6
39.32 DMSO
39.10 DMSO
38.90 DMSO
29.79
f1 (ppm)

OSM3-MZ-1159.12.1.1r

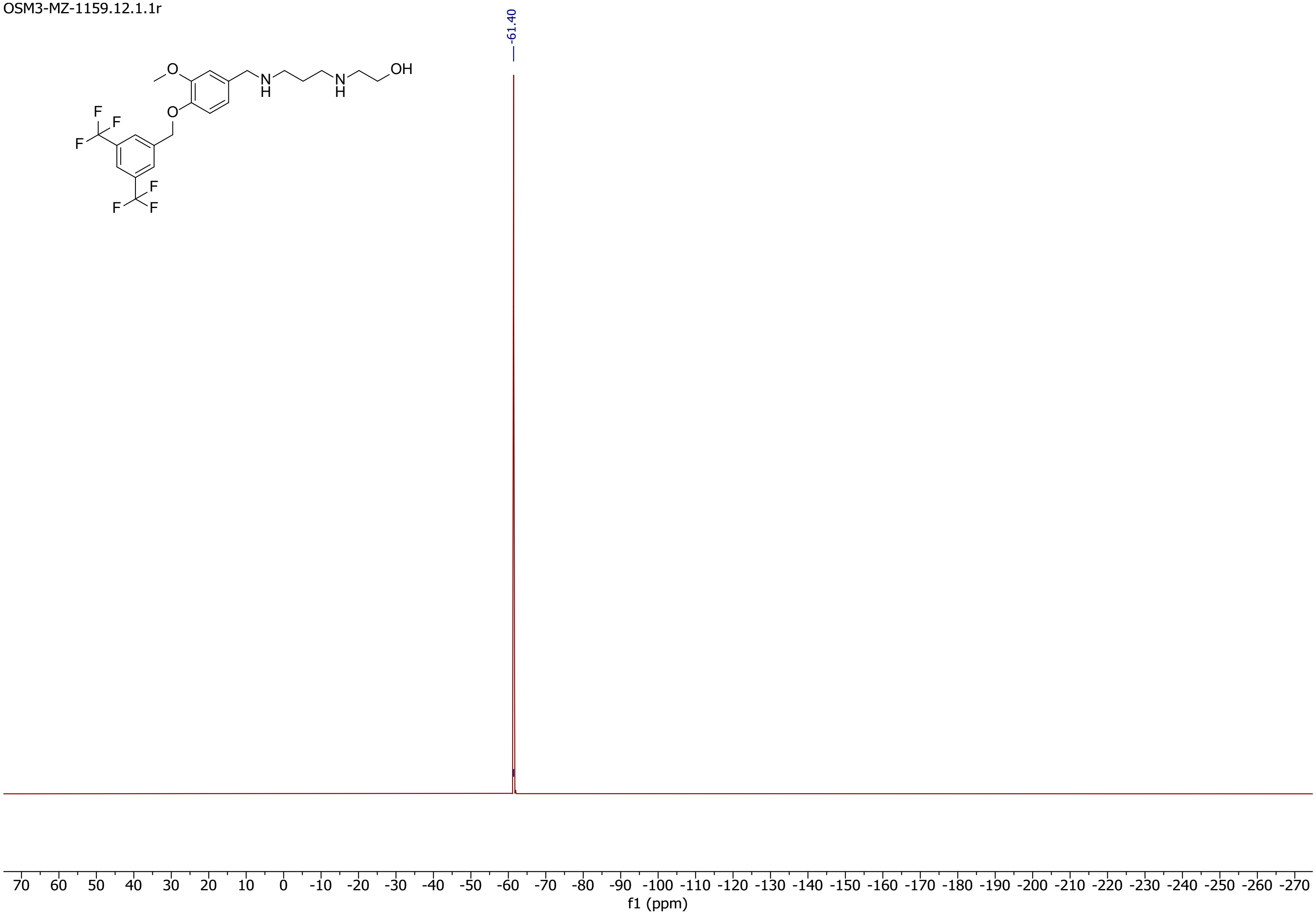

2-((3-((4-((5-chlorothiophen-2-yl)methoxy)-3-methoxybenzyl)amino)propyl)amino)ethan-1-ol (***2w***).

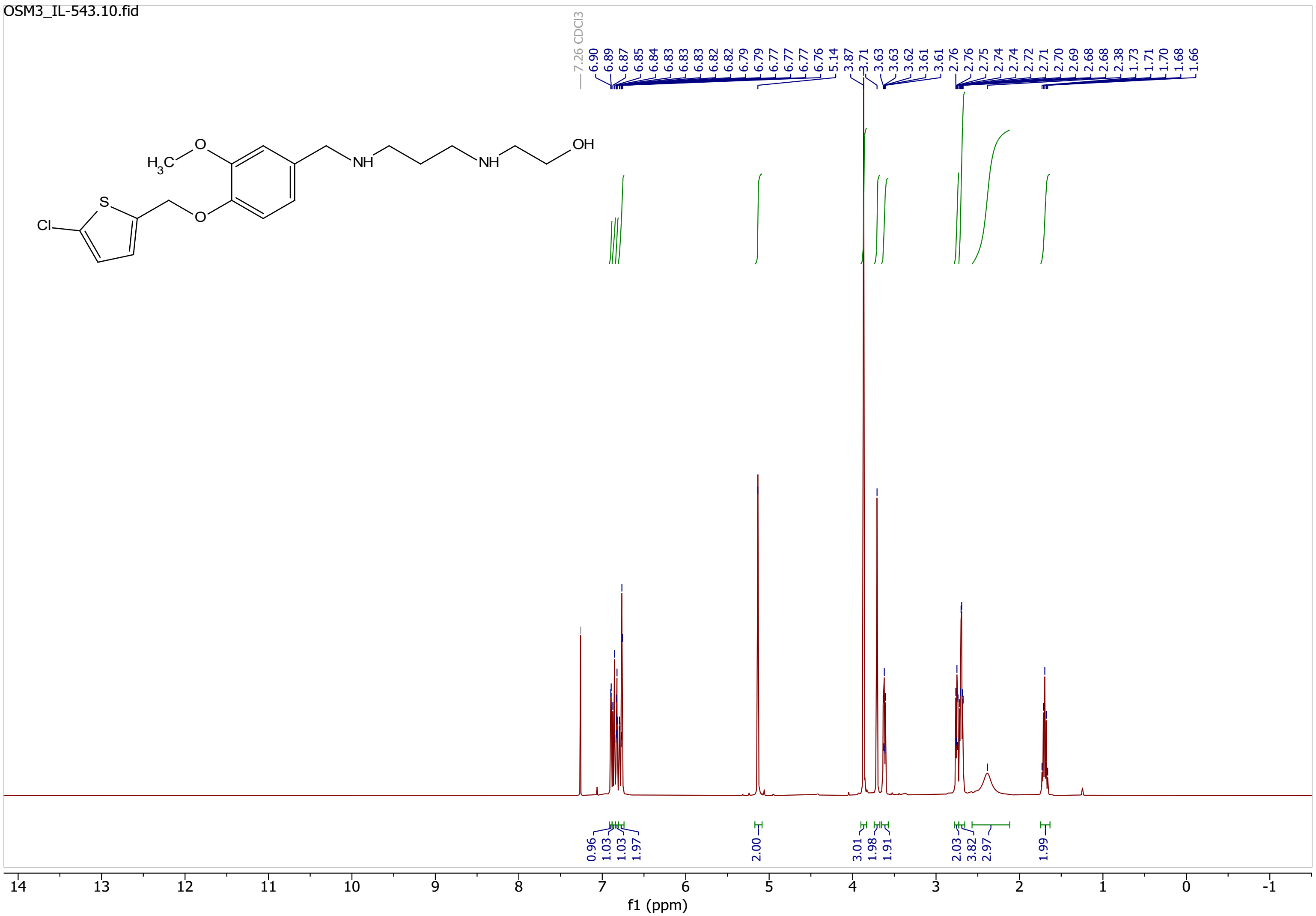

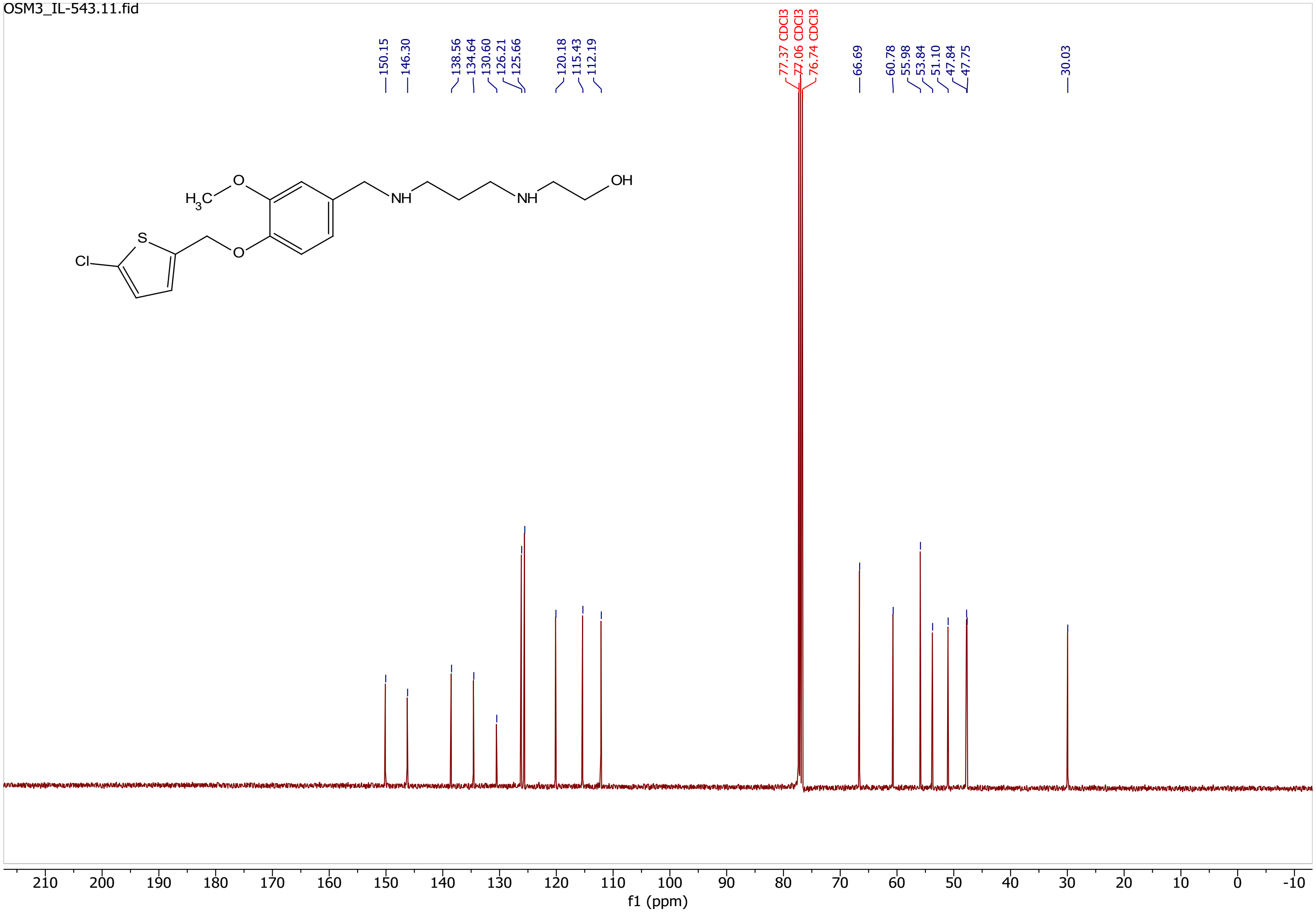
OSM3_IL-543.11.fid
150.15
146.30
138.56
134.64
130.60
126.21
125.66
120.18
115.43
112.19
77.37 CDCl3
77.06 CDCl3
76.74 CDCl3
66.69
60.78
55.98
53.84
51.10
47.84
47.75
30.03
H3C
O
NH
NH
OH
S
Cl
O
210 200 190 180 170 160 150 140 130 120 110 100 90 80 70 60 50 40 30 20 10 0 -10
f1 (ppm)

2-((3-((4-(Benzo[b]thiophen-2-ylmethoxy)-3-methoxybenzyl)amino)propyl)amino)ethan-1-ol (***2x***).

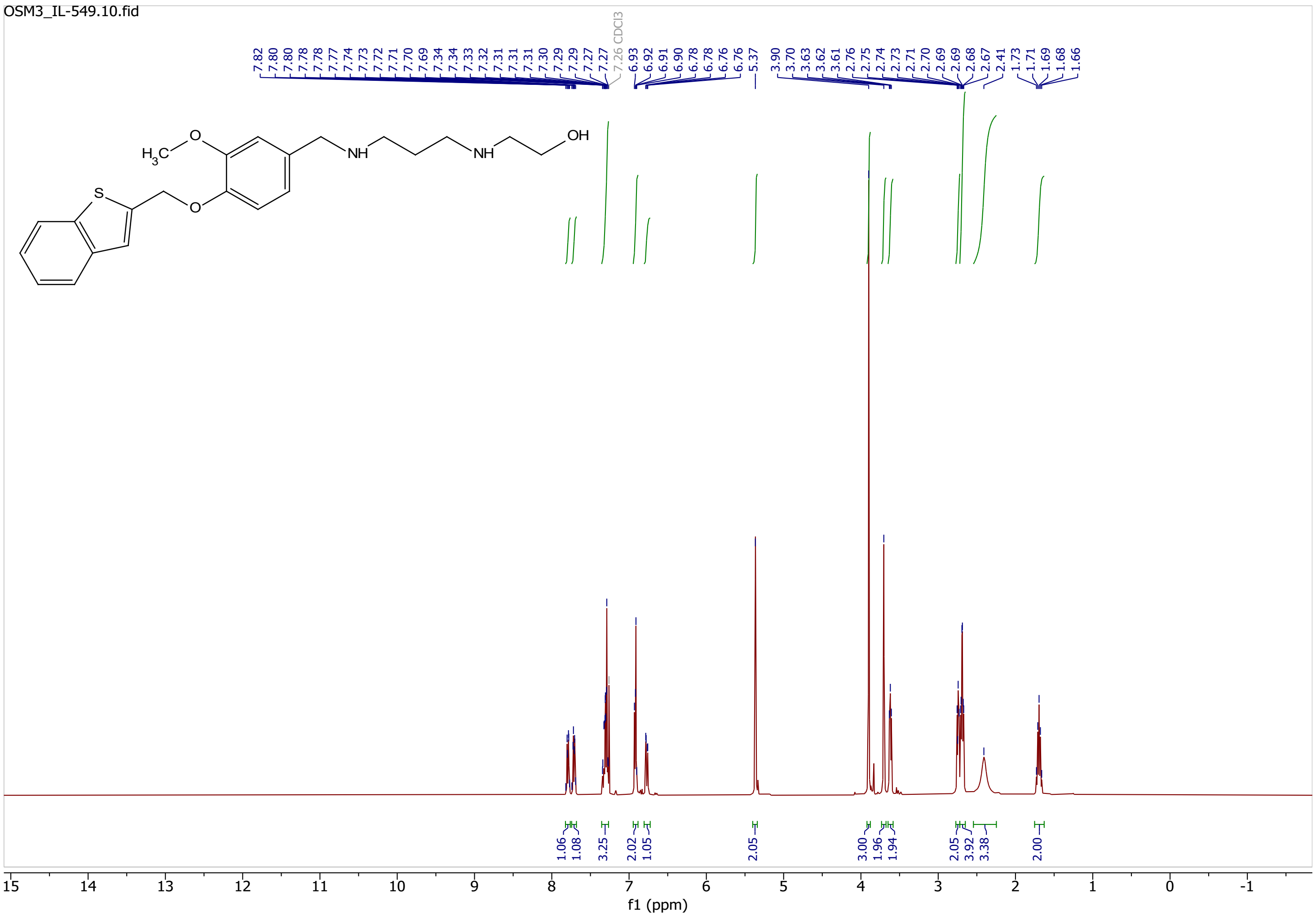

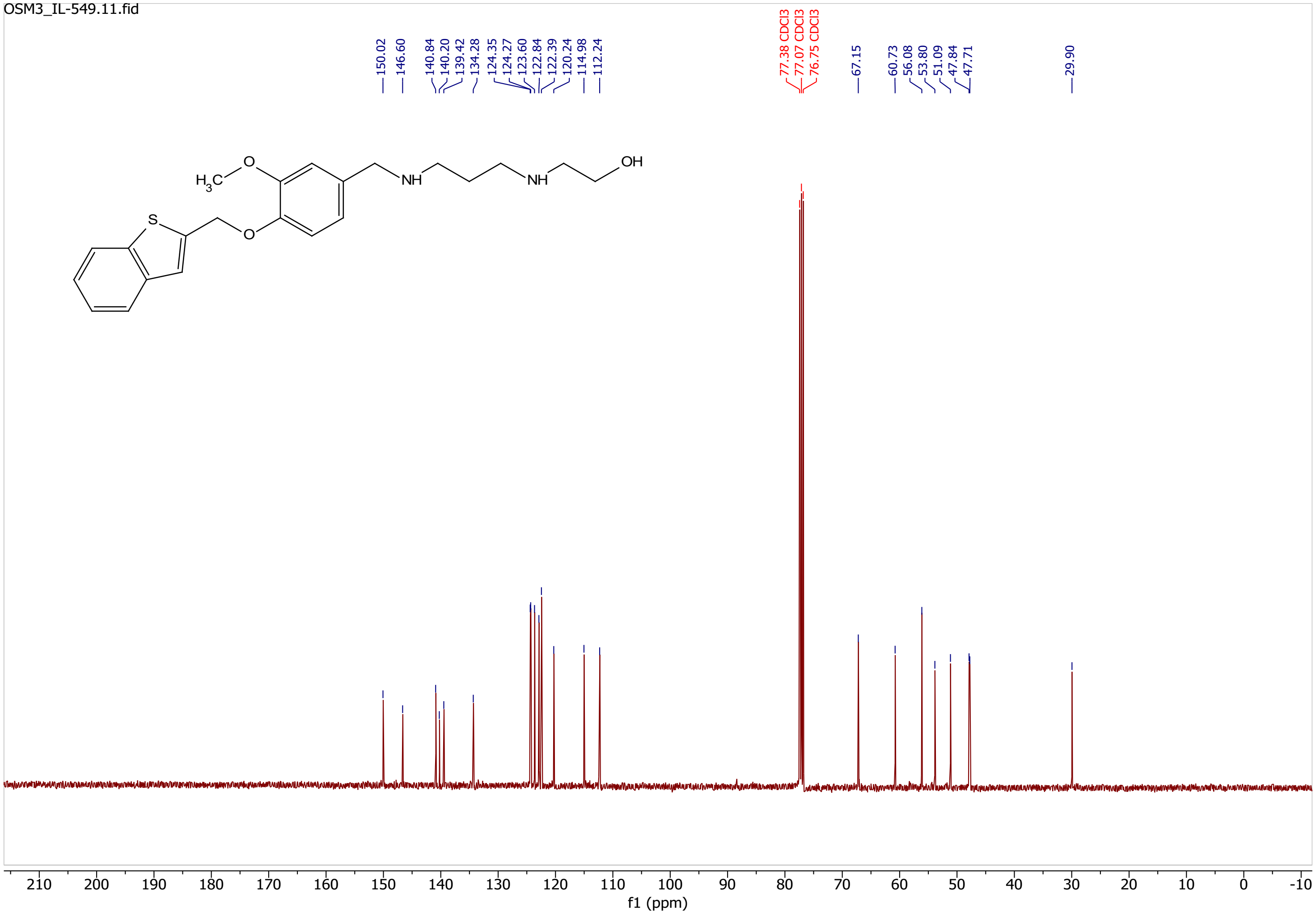
OSM3_IL-549.11.fid
150.02
146.60
140.84
140.20
139.42
134.28
124.35
124.27
123.60
122.84
122.39
120.24
114.98
112.24
77.38 CDCl3
77.07 CDCl3
76.75 CDCl3
67.15
60.73
56.08
53.80
51.09
47.84
47.71
29.90
H3C
O
S
O
NH
NH
OH
210
200
190
180
170
160
150
140
130
120
110
100
90
80
70
60
50
40
30
20
10
0
-10
f1 (ppm)

*2-((3-((4-(Isoquinolin-3-ylmethoxy)-3-methoxybenzyl)amino)propyl)amino)- ethan-1-ol (**2y**).*

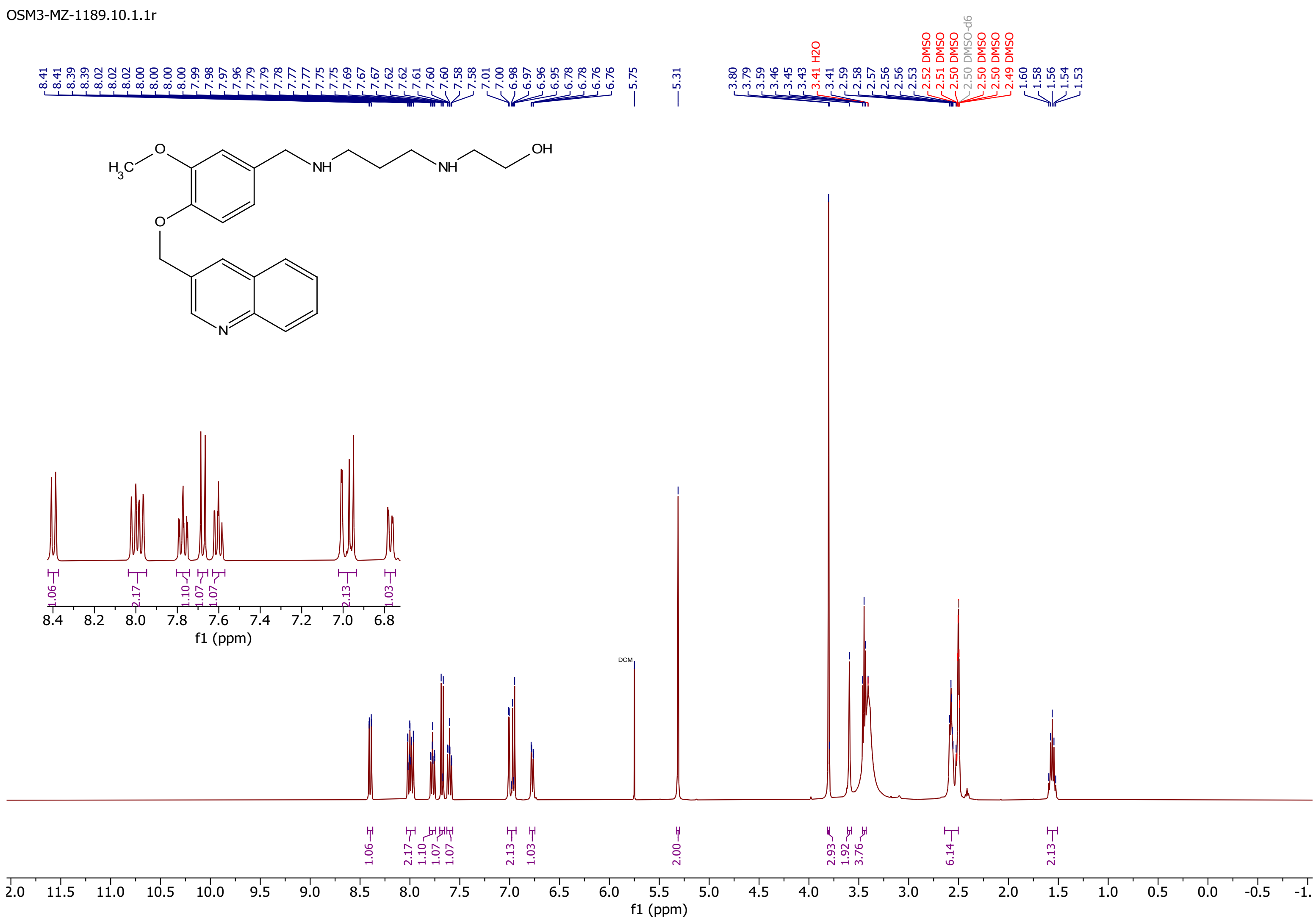

OSM3-MZ-1189.11.1.1r

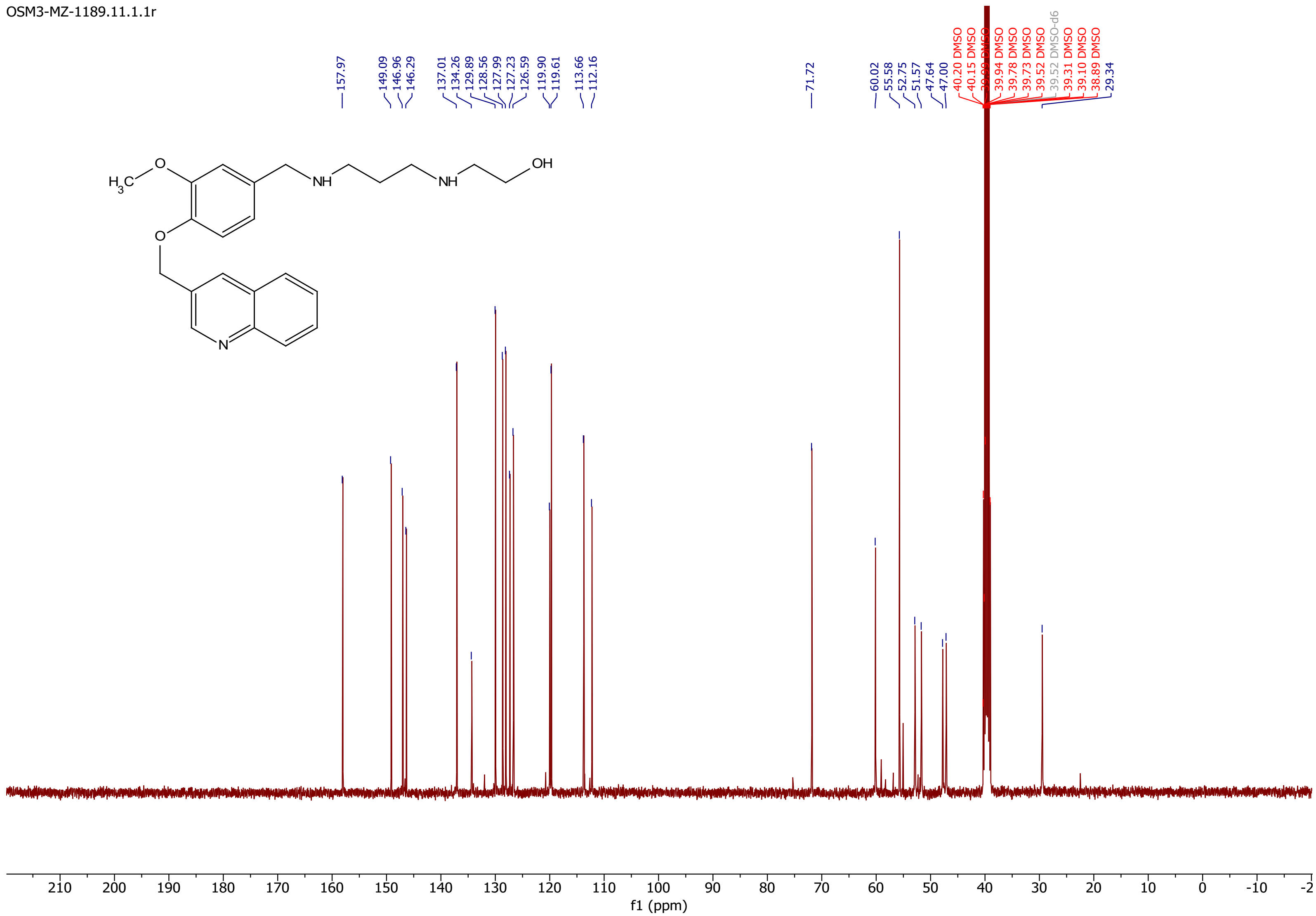

2-((3-((3-Methoxy-4-((6-methylpyridin-2-yl)methoxy)benzyl)amino)propyl)- amino)ethan-1-ol (***2z***).

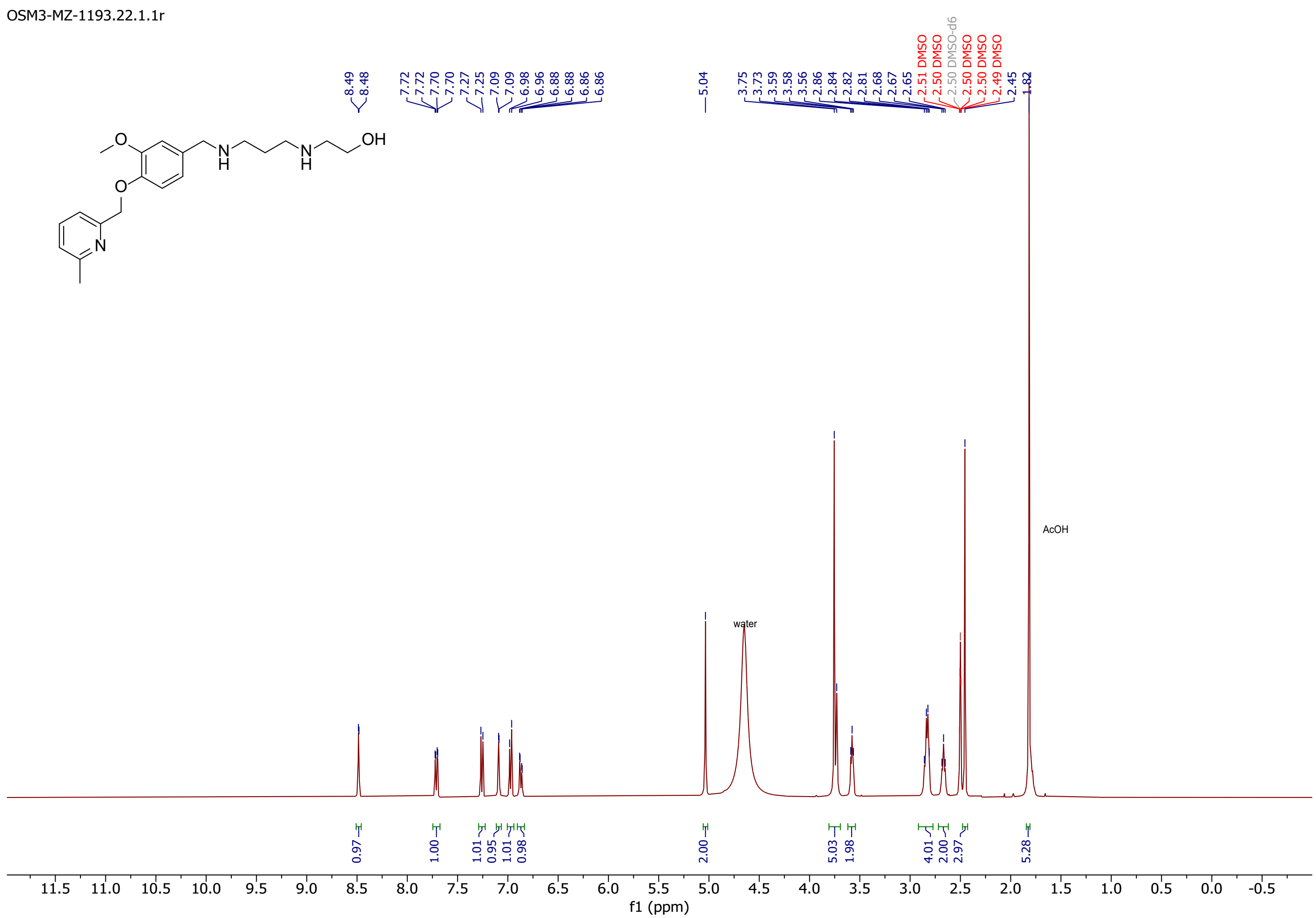

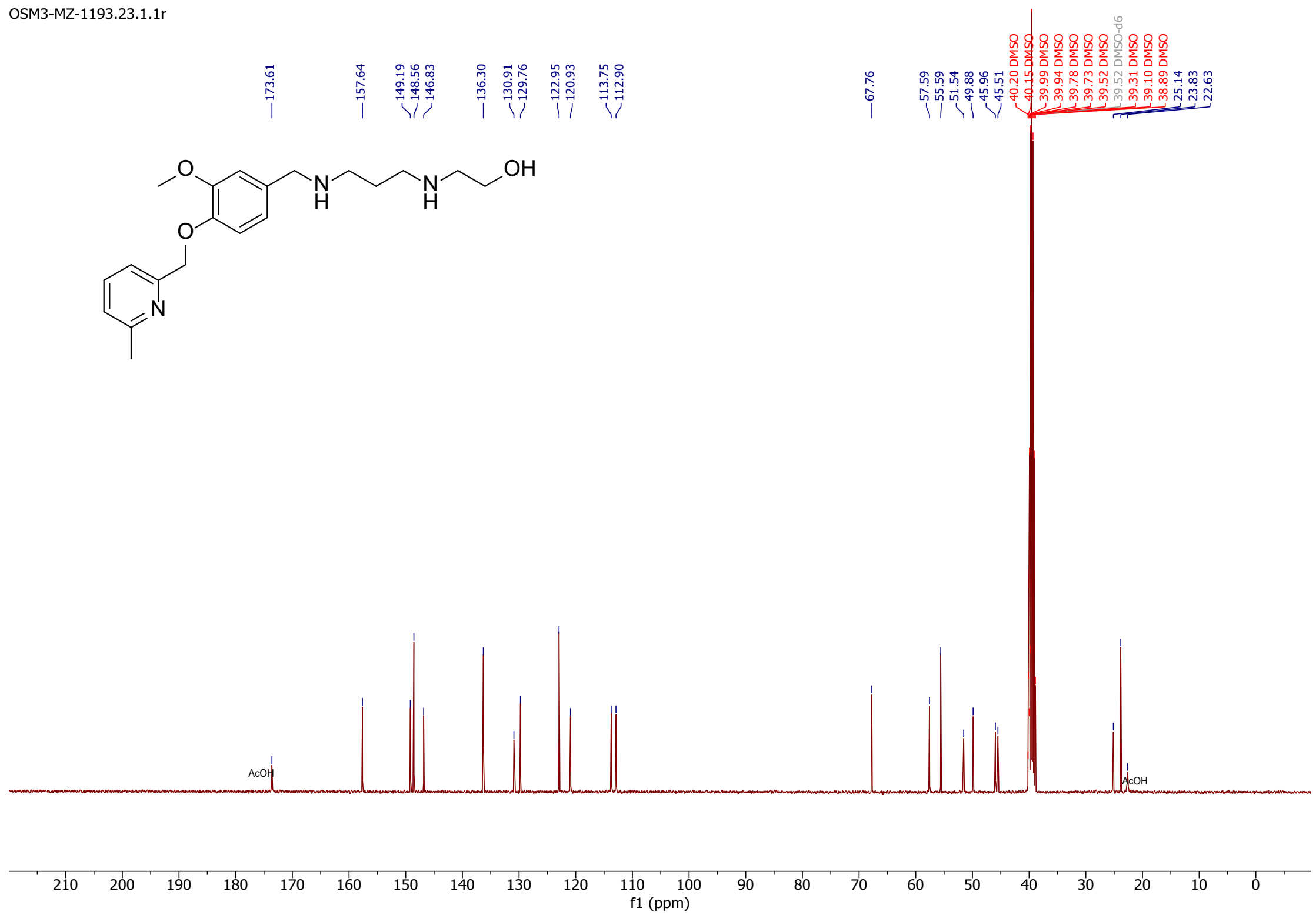
OSM3-MZ-1193.23.1.1r
173.61
157.64
149.19
148.56
146.83
136.30
130.91
129.76
122.95
120.93
113.75
112.90
67.76
57.59
55.59
51.54
49.88
45.96
45.51
40.20 DMSO
40.15 DMSO
39.99 DMSO
39.94 DMSO
39.78 DMSO
39.73 DMSO
39.52 DMSO
39.52 DMSO-d6
39.31 DMSO
39.10 DMSO
38.89 DMSO
25.14
23.83
22.63
AcOH
AcOH
210 200 190 180 170 160 150 140 130 120 110 100 90 80 70 60 50 40 30 20 10 0
f1 (ppm)

2-((3-((4-((3-Chlorobenzyl)oxy)-3-ethoxybenzyl)amino)propyl)amino)ethan-1-ol (***3a***).

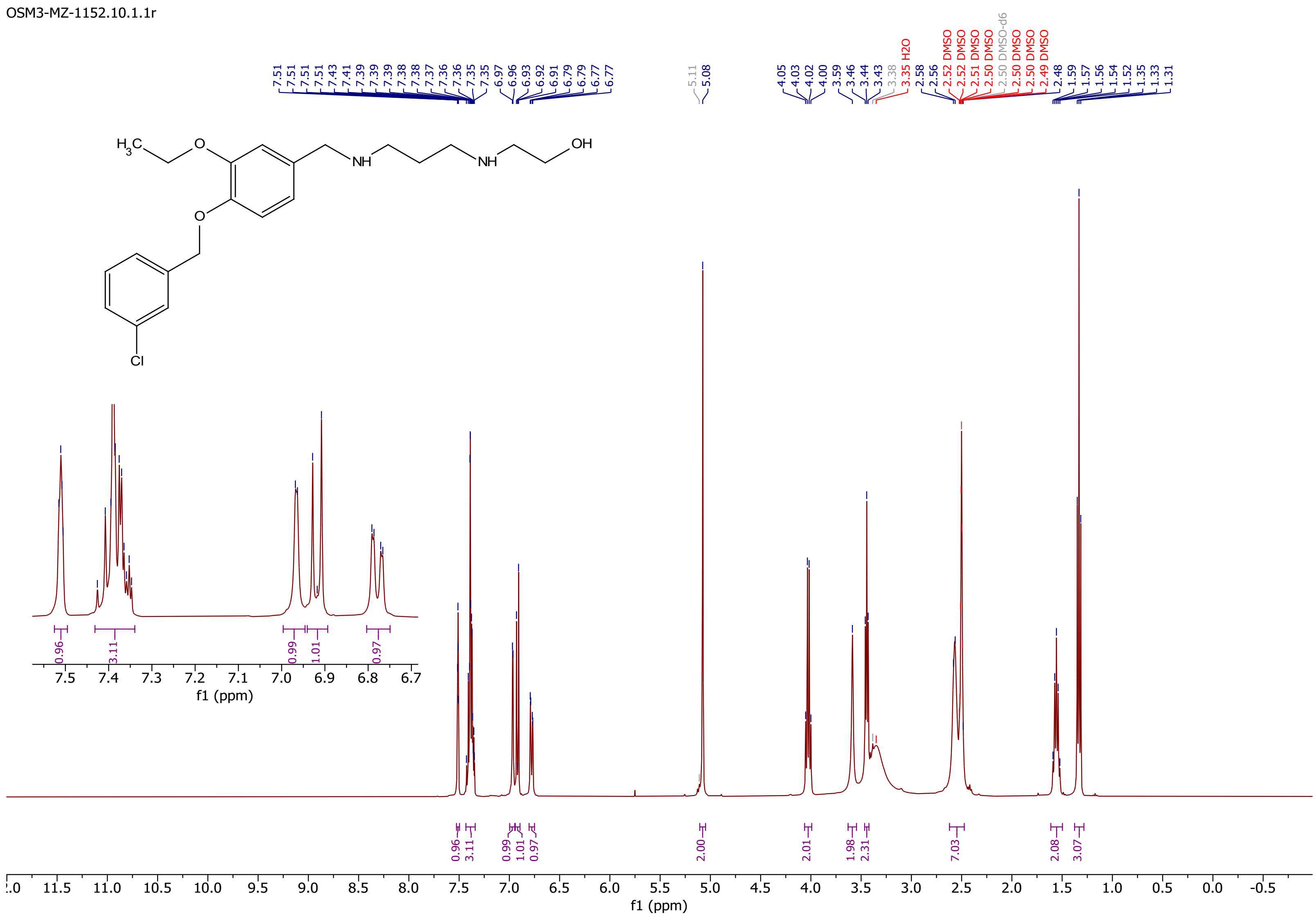

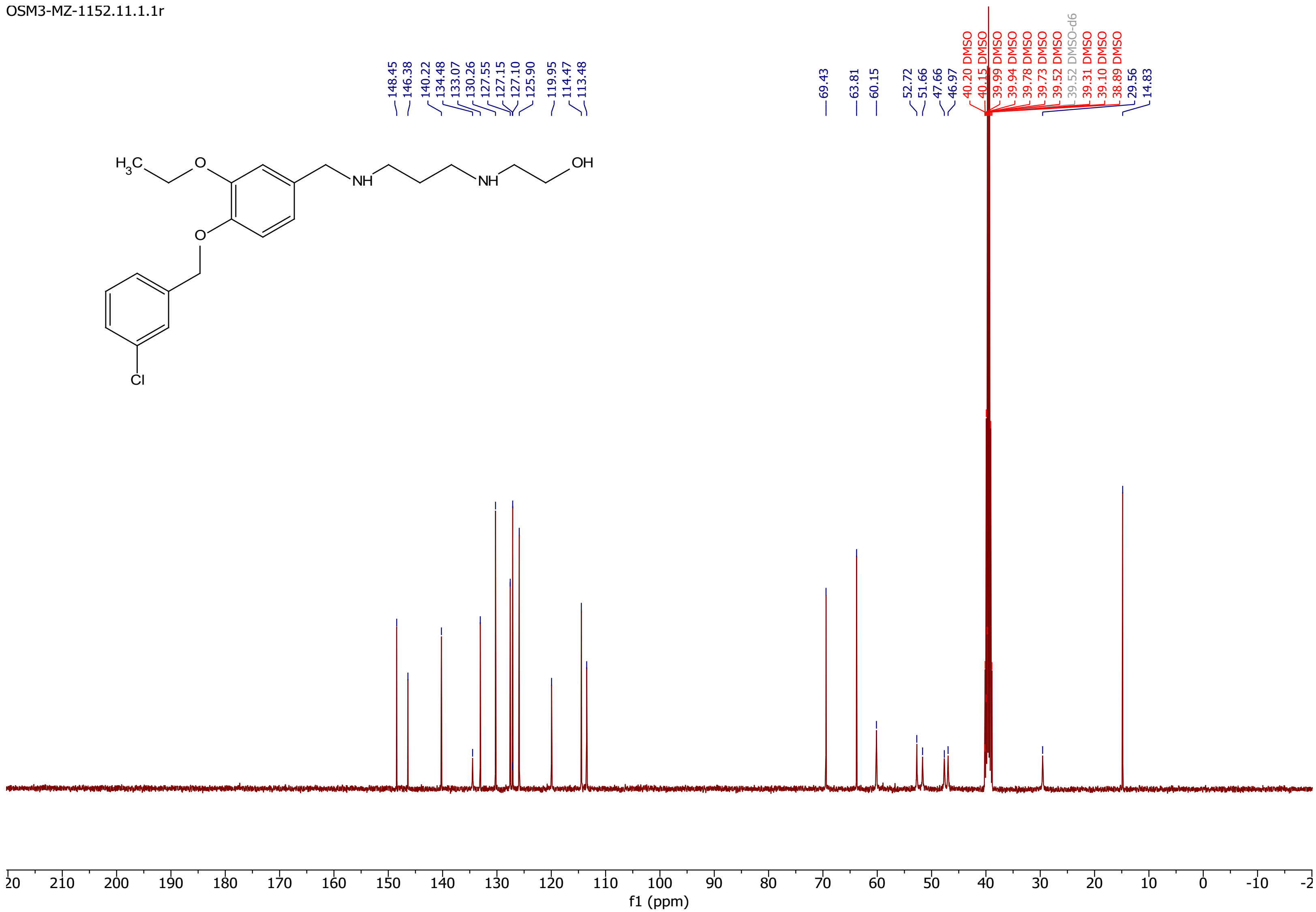
OSM3-MZ-1152.11.1.1r
148.45
146.38
140.22
134.48
133.07
130.26
127.55
127.15
127.10
125.90
119.95
114.47
113.48
69.43
63.81
60.15
52.72
51.66
47.66
46.97
40.20 DMSO
40.15 DMSO
39.99 DMSO
39.94 DMSO
39.78 DMSO
39.73 DMSO
39.52 DMSO
39.52 DMSO-d6
39.31 DMSO
39.10 DMSO
38.89 DMSO
29.56
14.83
H3C
O
O
NH
NH
OH
Cl
f1 (ppm)

2-((3-((4-((3-Chlorobenzyl)oxy)-3-(isopentyloxy)benzyl)amino)propyl)amino)ethan-1-ol (***3b***).

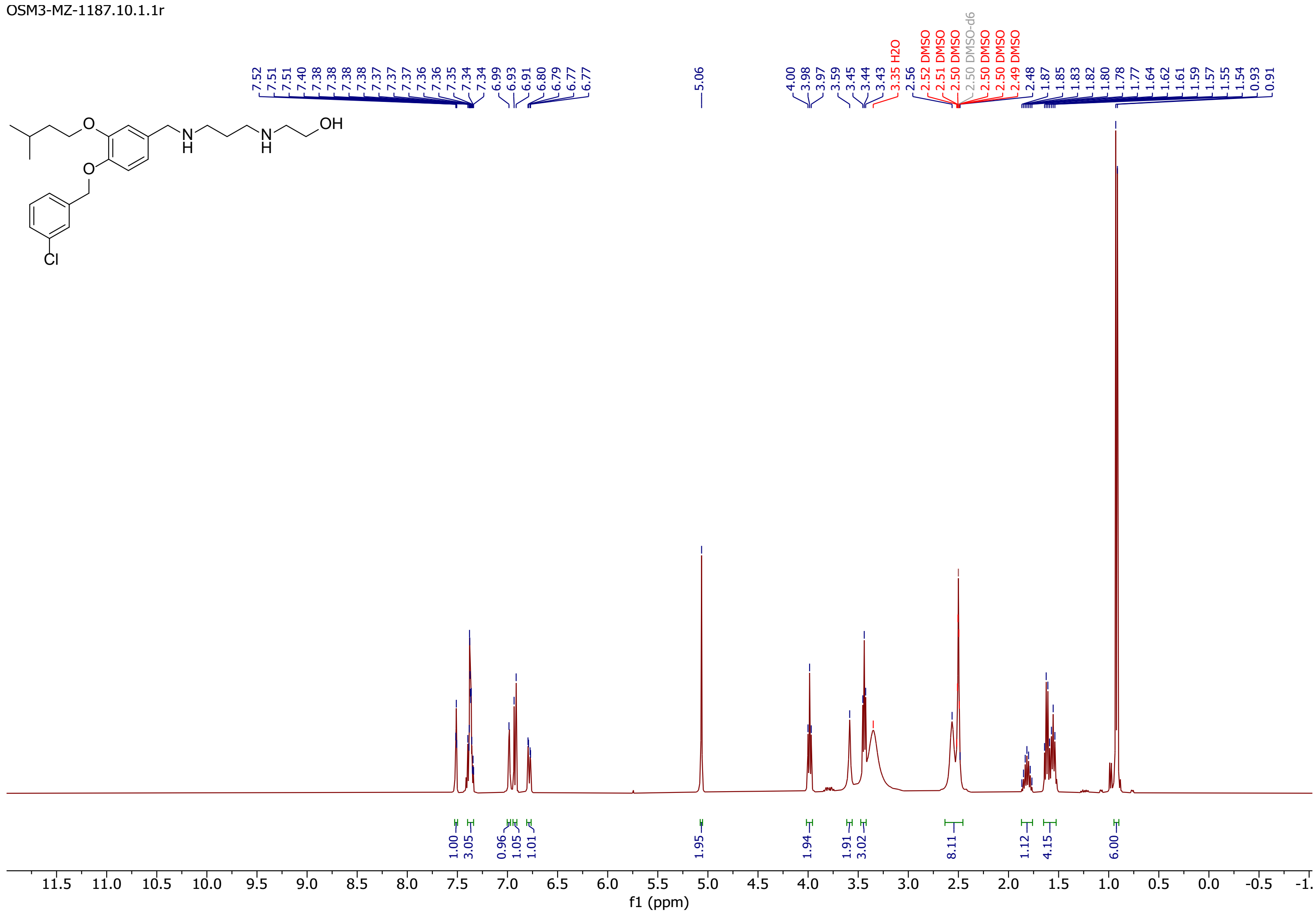

OSM3-MZ-1187.11.1.1r

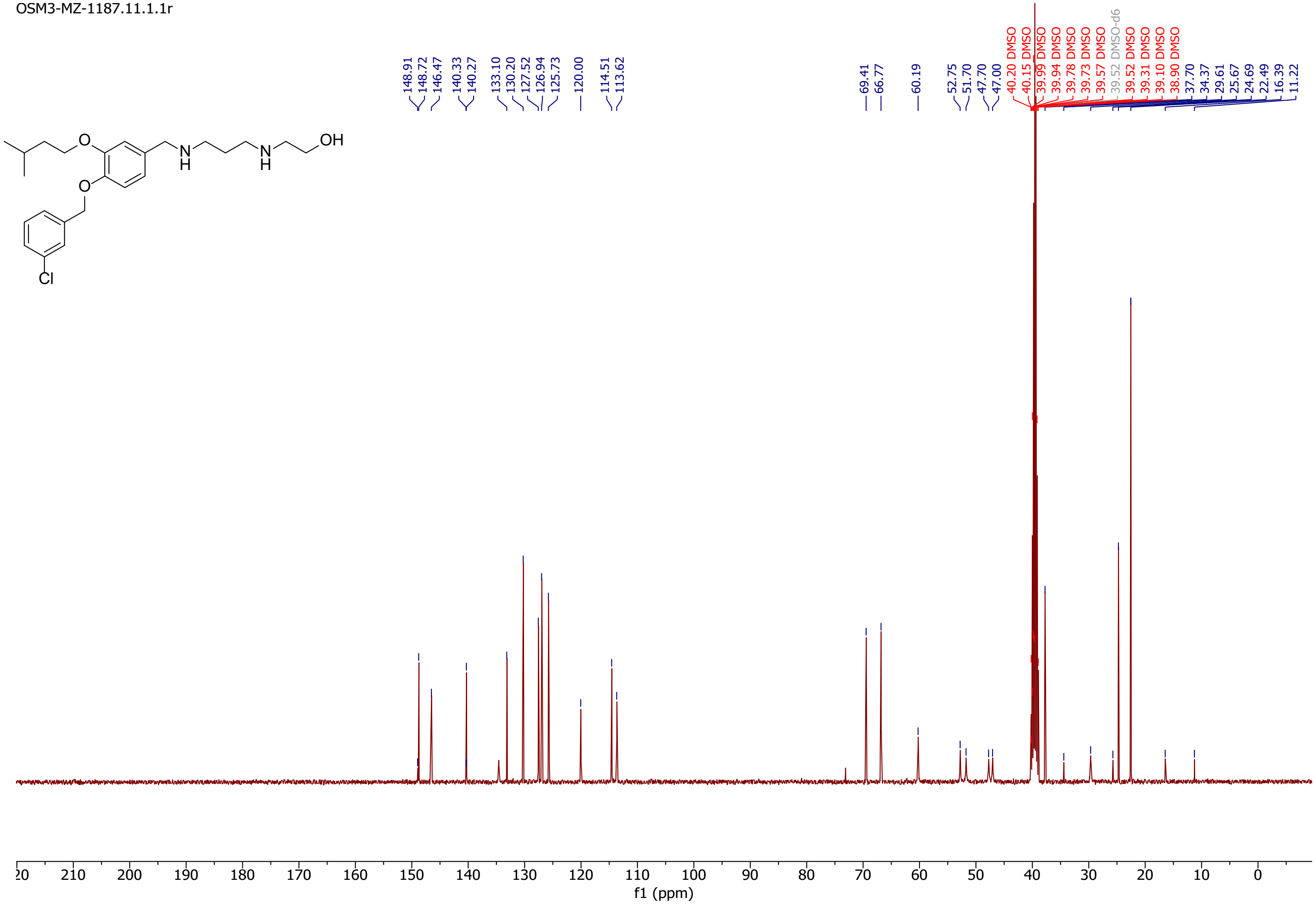

2-((3-((4-((3-Chlorobenzyl)oxy)-3-(cyclopentylmethoxy)benzyl)amino)propyl)amino)ethan-1-ol (***3c***).

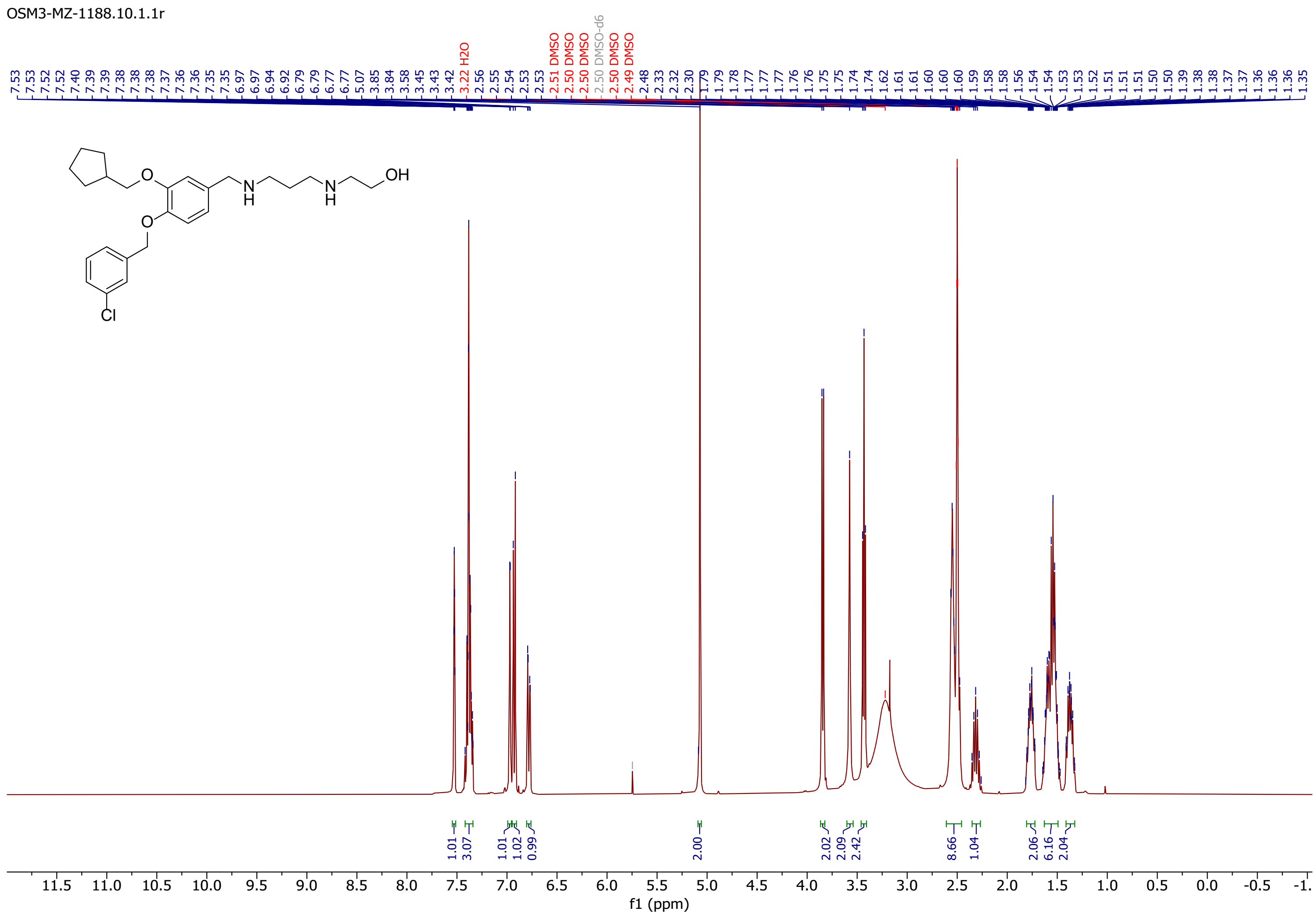

OSM3-MZ-1188.11.1.1r

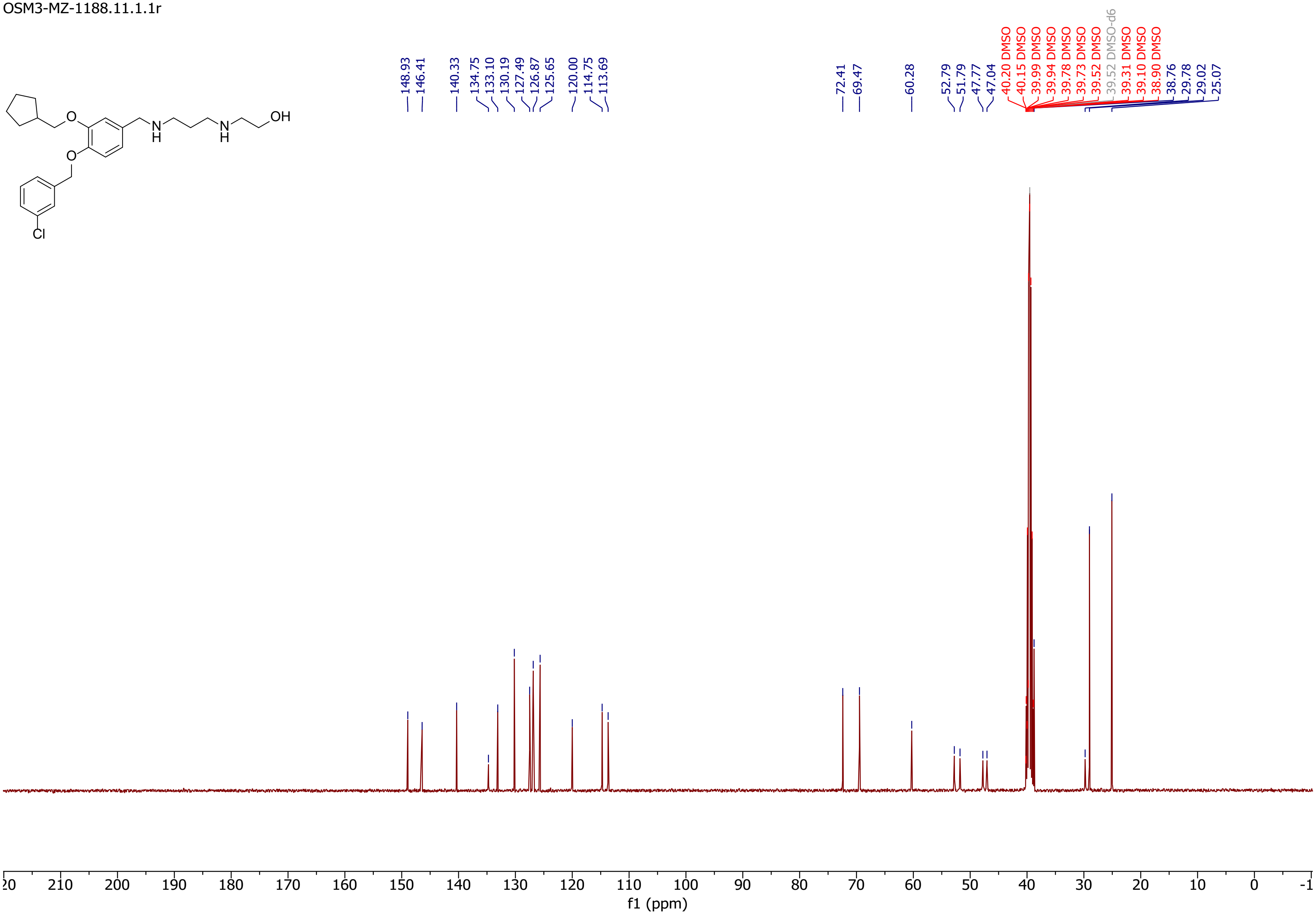

2-((3-((4-((3-Chlorobenzyl)oxy)-3-(isopentyloxy)benzyl)amino)propyl)amino)ethan-1-ol (***3d***).

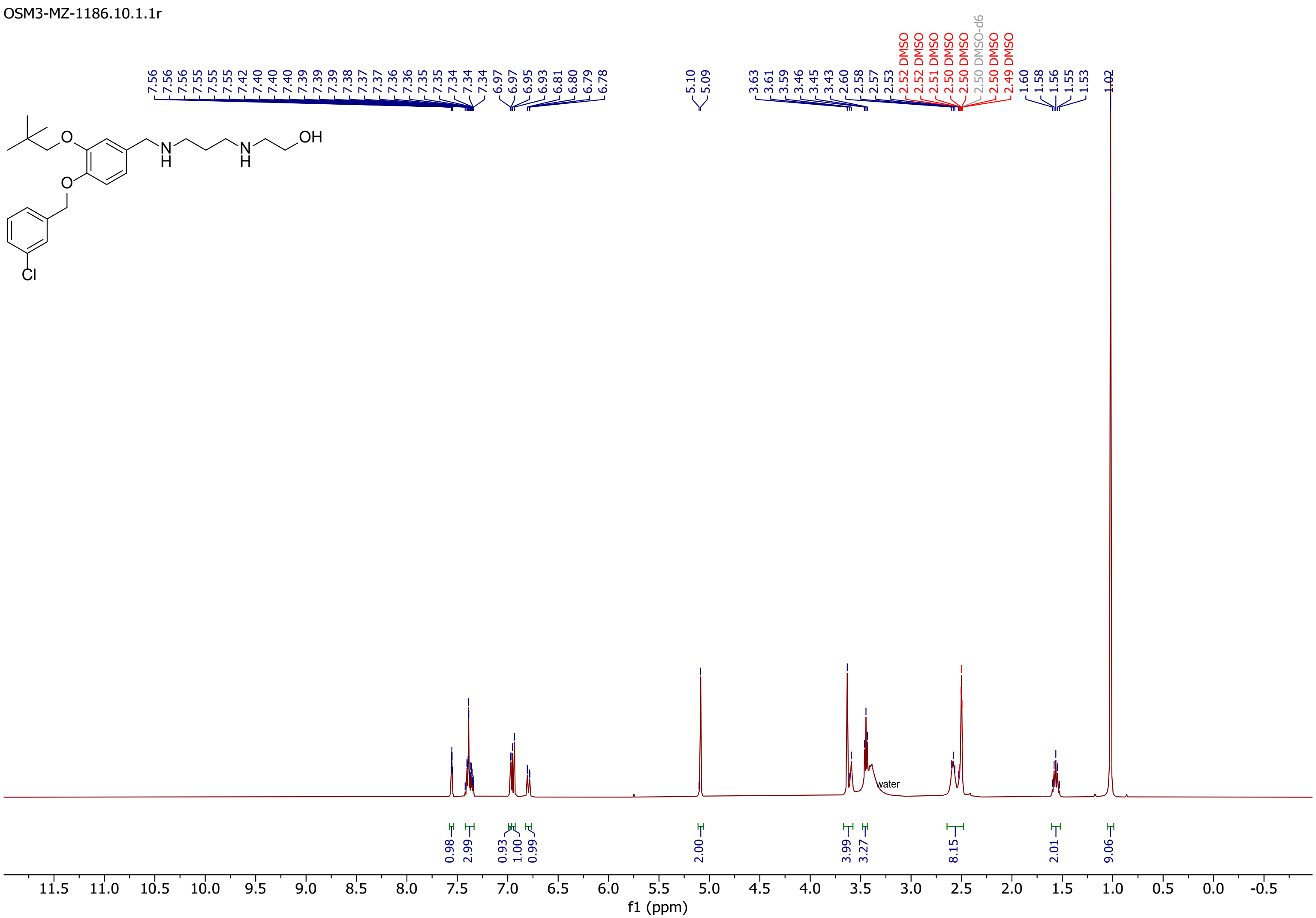

OSM3-MZ-1186.11.1.1r

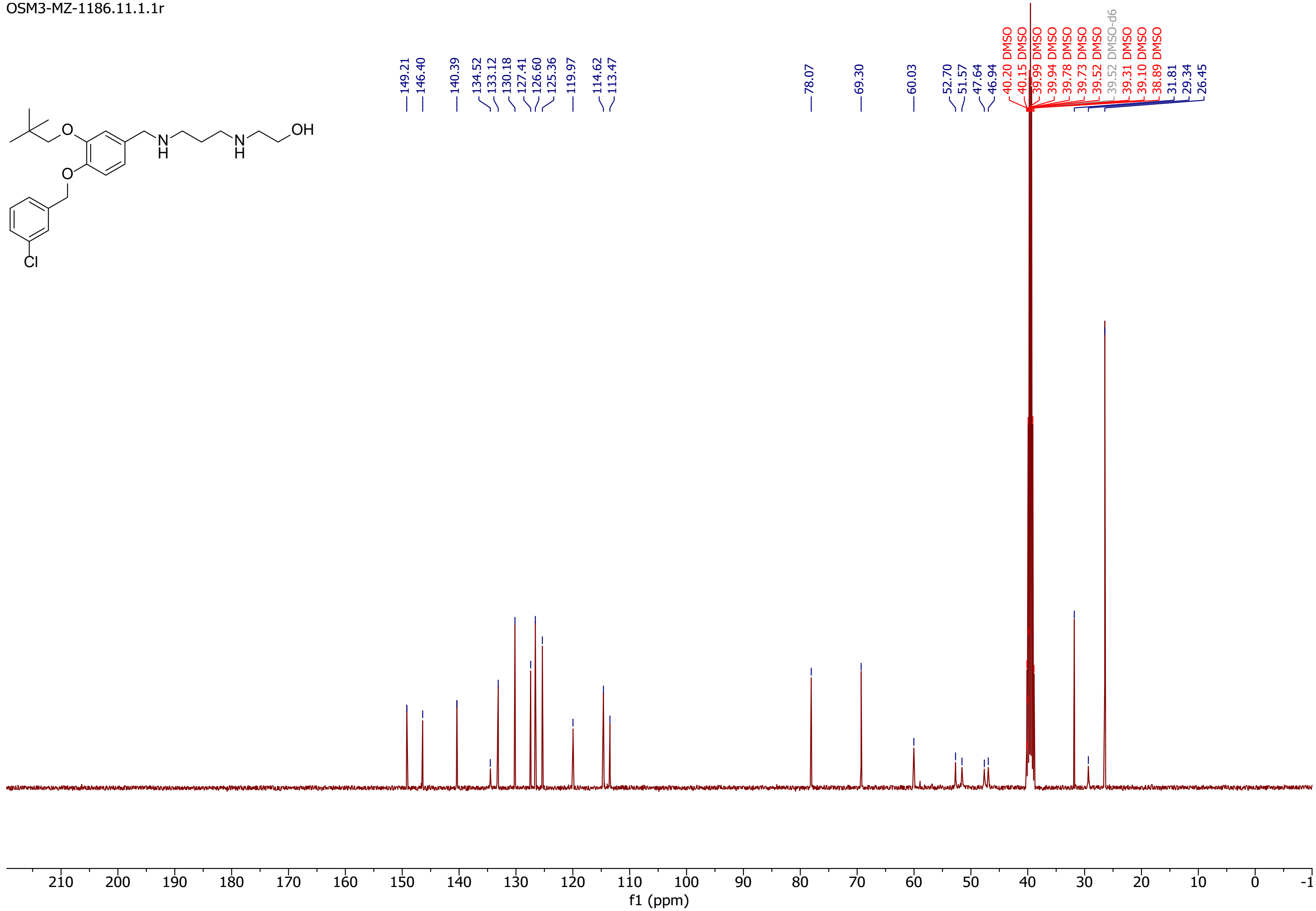

2-((3-((4-((3-Chlorobenzyl)oxy)-3-(cyclohexylmethoxy)benzyl)amino)propyl)amino)ethan-1-ol (***3e***).

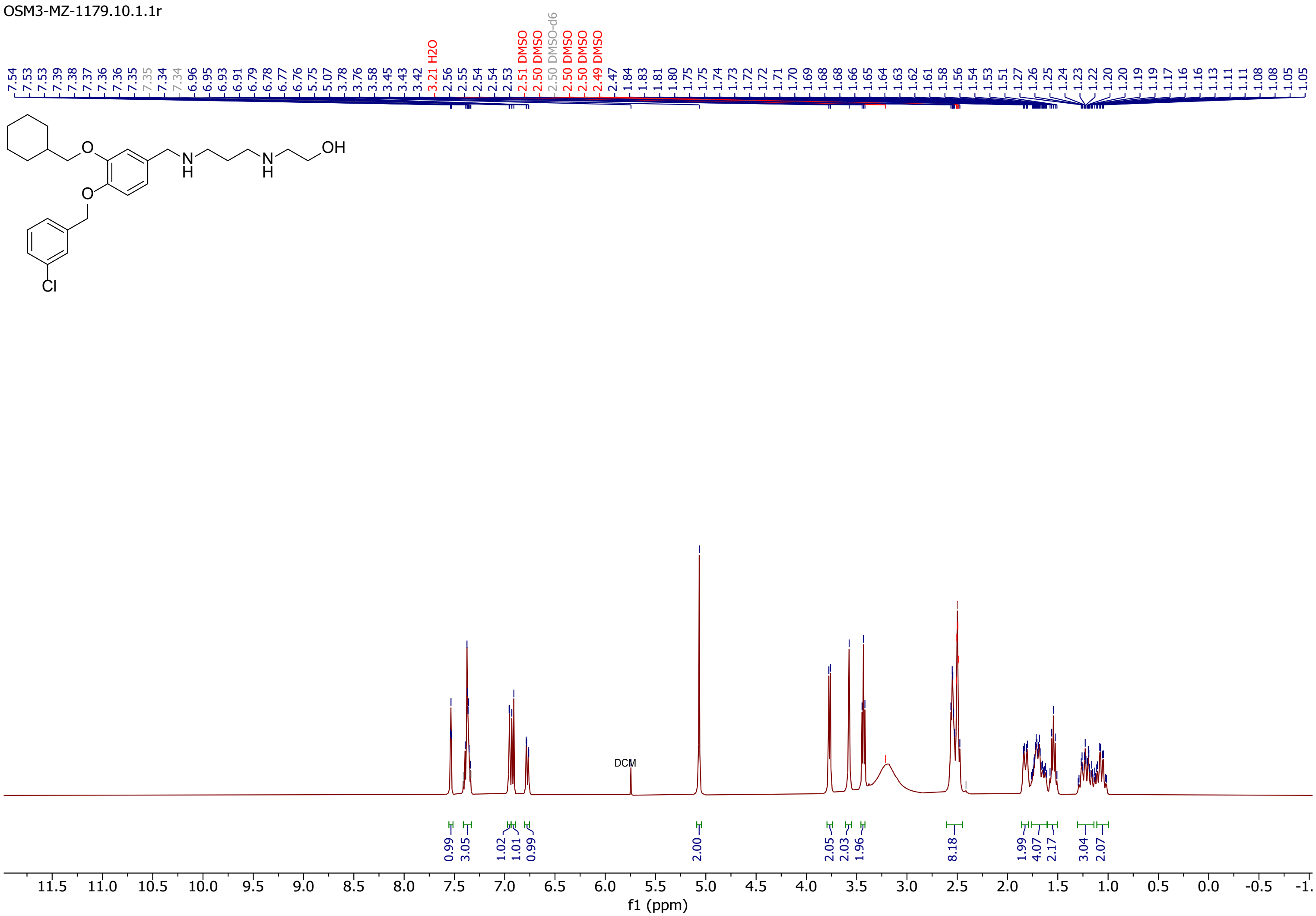

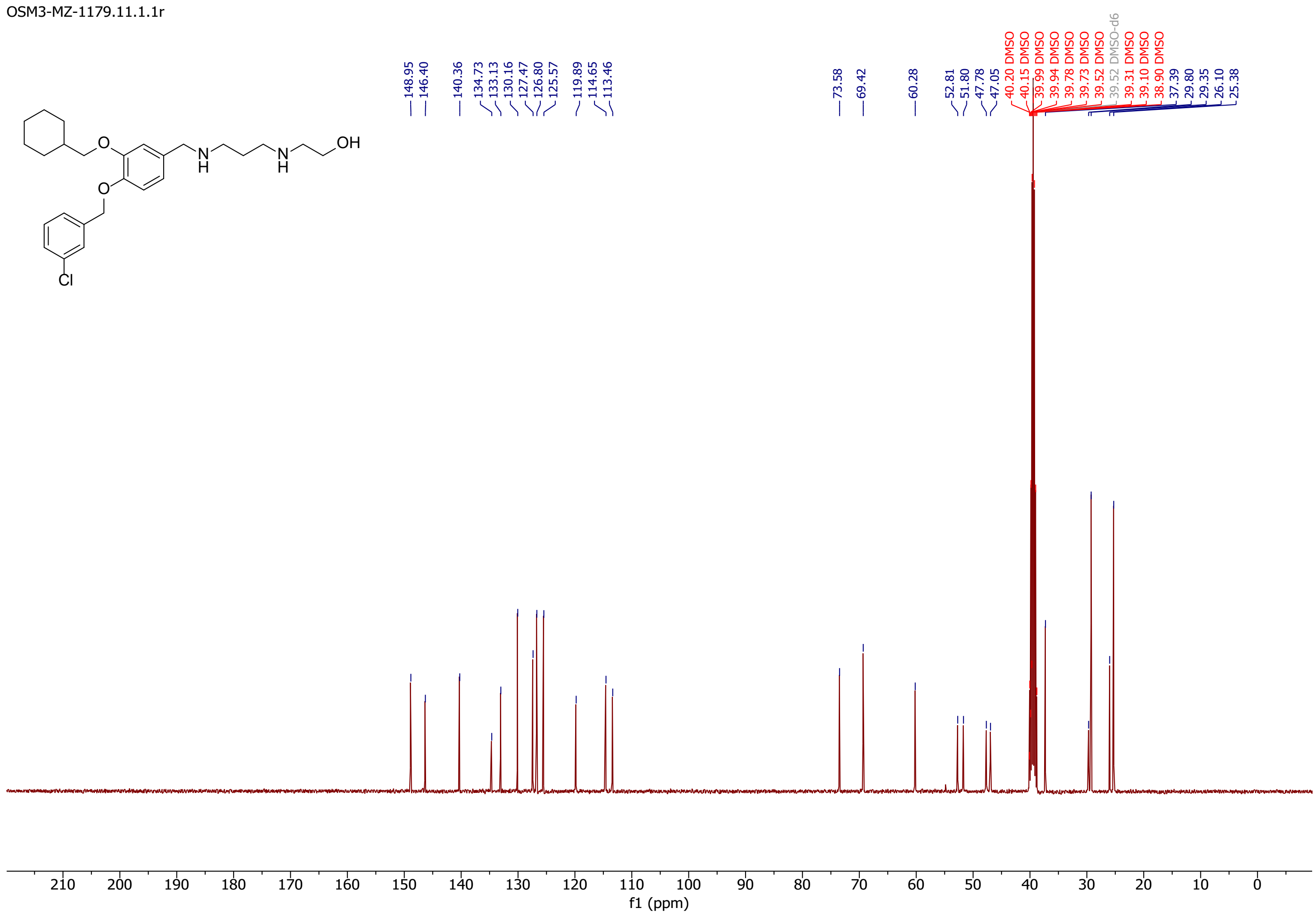
OSM3-MZ-1179.11.1.1r
148.95
146.40
140.36
134.73
133.13
130.16
127.47
126.80
125.57
119.89
114.65
113.46
73.58
69.42
60.28
52.81
51.80
47.78
47.05
40.20 DMSO
40.15 DMSO
39.99 DMSO
39.94 DMSO
39.78 DMSO
39.73 DMSO
39.52 DMSO
39.52 DMSO-d6
39.31 DMSO
39.10 DMSO
38.90 DMSO
37.39
29.80
29.35
26.10
25.38
O
O
N
H
N
H
OH
Cl
210 200 190 180 170 160 150 140 130 120 110 100 90 80 70 60 50 40 30 20 10 0
f1 (ppm)

*2-((3-((3-Benzyloxy-4-((3-chlorobenzyl)oxy)benzyl)amino)propyl)amino)ethan-1-ol (**3f**).*

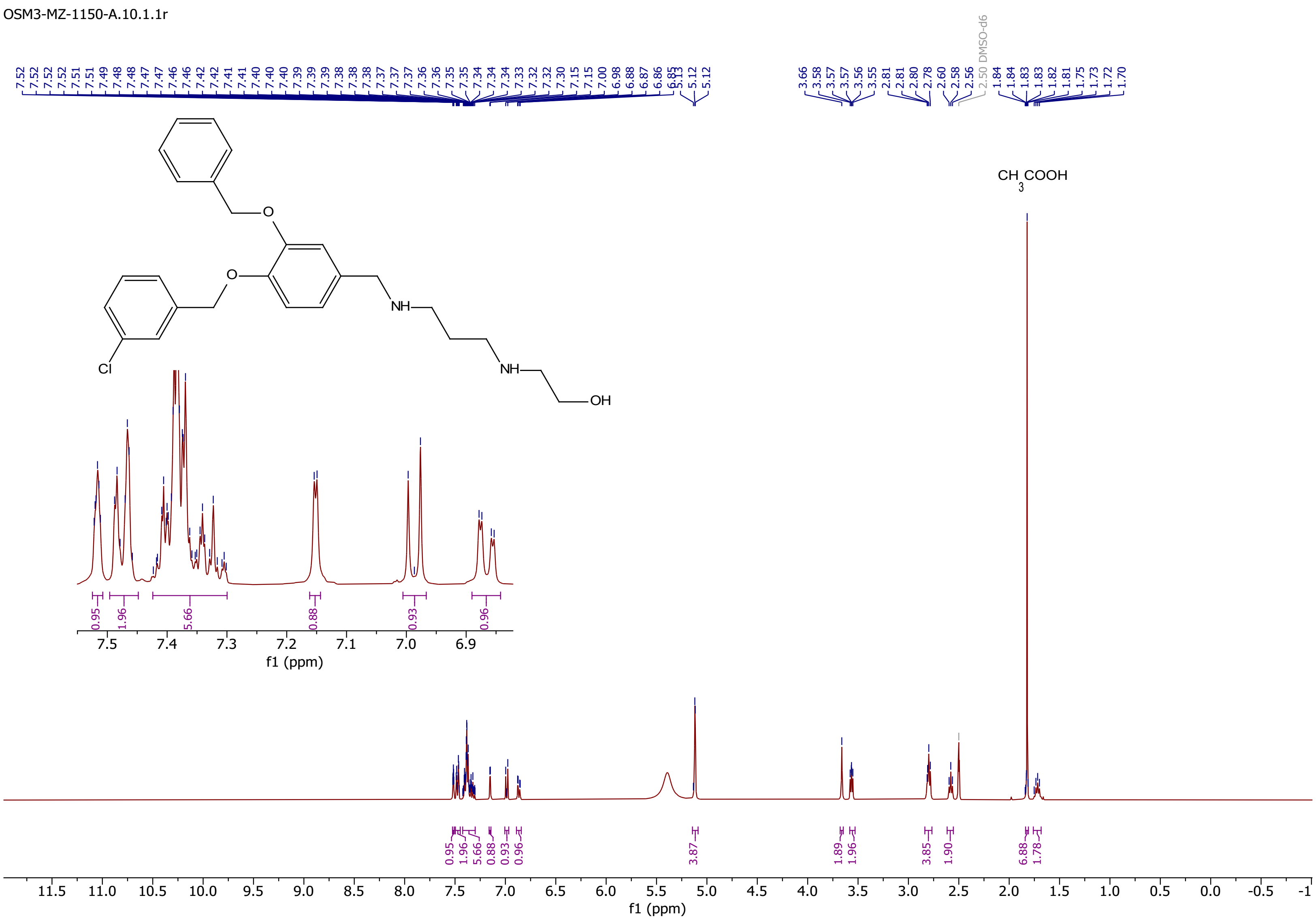

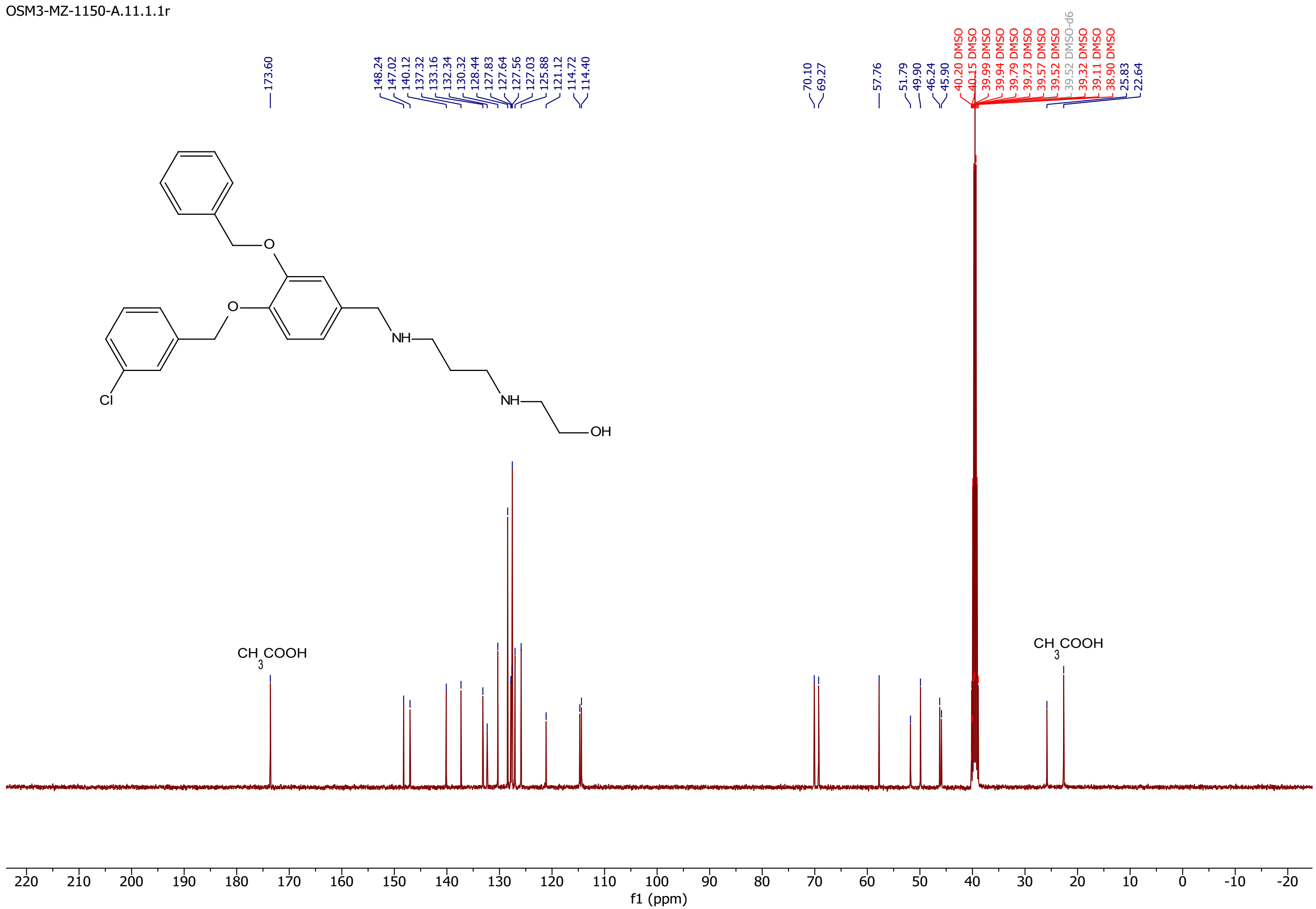
OSM3-MZ-1150-A.11.1.1r
173.60
148.24
147.02
140.12
137.32
133.16
132.34
130.32
128.44
127.83
127.64
127.56
127.03
125.88
121.12
114.72
114.40
70.10
69.27
57.76
51.79
49.90
46.24
45.90
40.20 DMSO
40.15 DMSO
39.99 DMSO
39.94 DMSO
39.79 DMSO
39.73 DMSO
39.57 DMSO
39.52 DMSO-d6
39.32 DMSO
39.11 DMSO
38.90 DMSO
25.83
22.64
O
O
NH
NH
OH
Cl
CH3COOH
CH3COOH
220 210 200 190 180 170 160 150 140 130 120 110 100 90 80 70 60 50 40 30 20 10 0 -10 -20
f1 (ppm)

2-((3-((4-((3,5-Dichlorobenzyl)oxy)benzyl)amino)propyl)amino)ethan-1-ol (***4a***).

OSM3-MZ-1237.10.1.1r

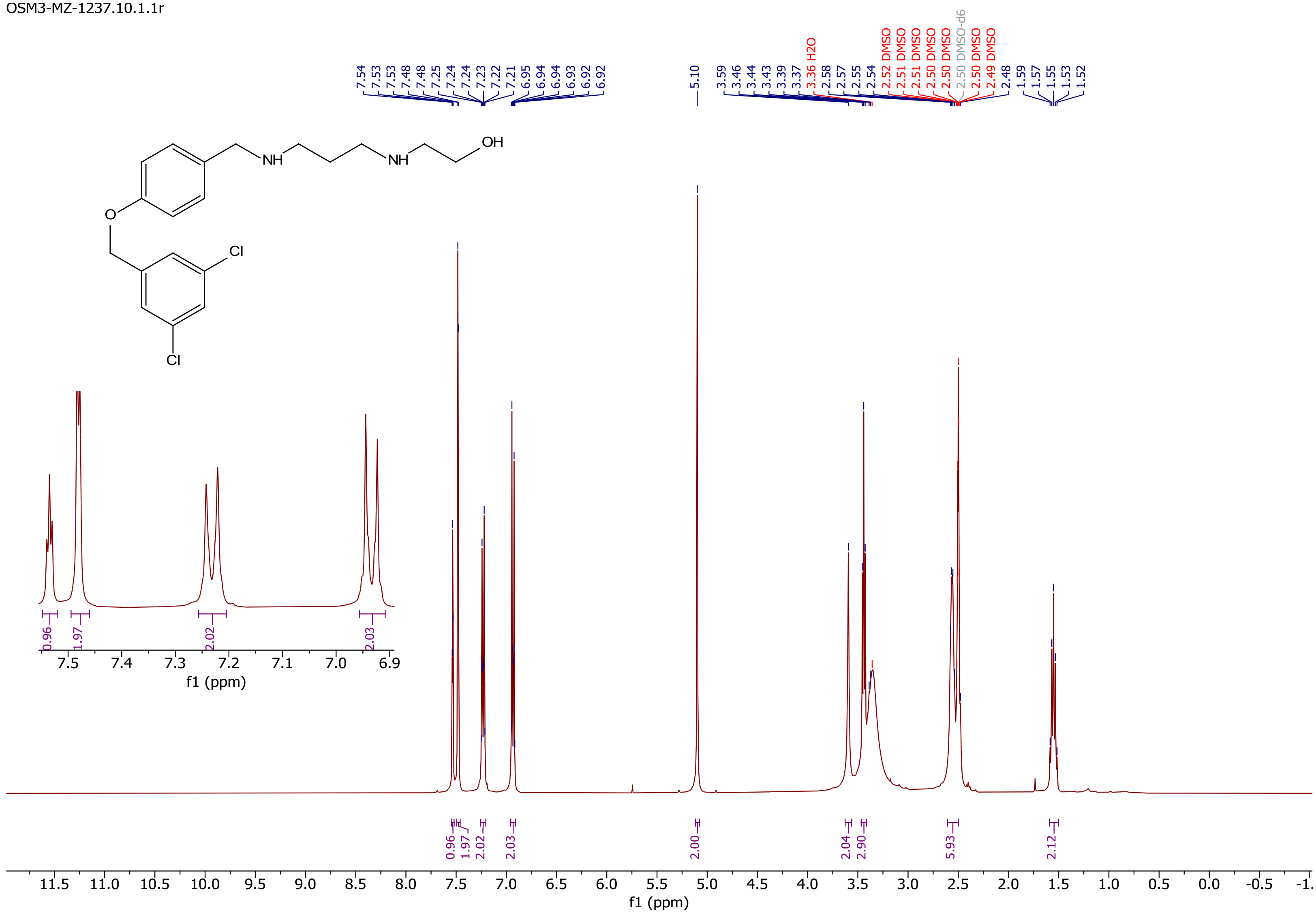

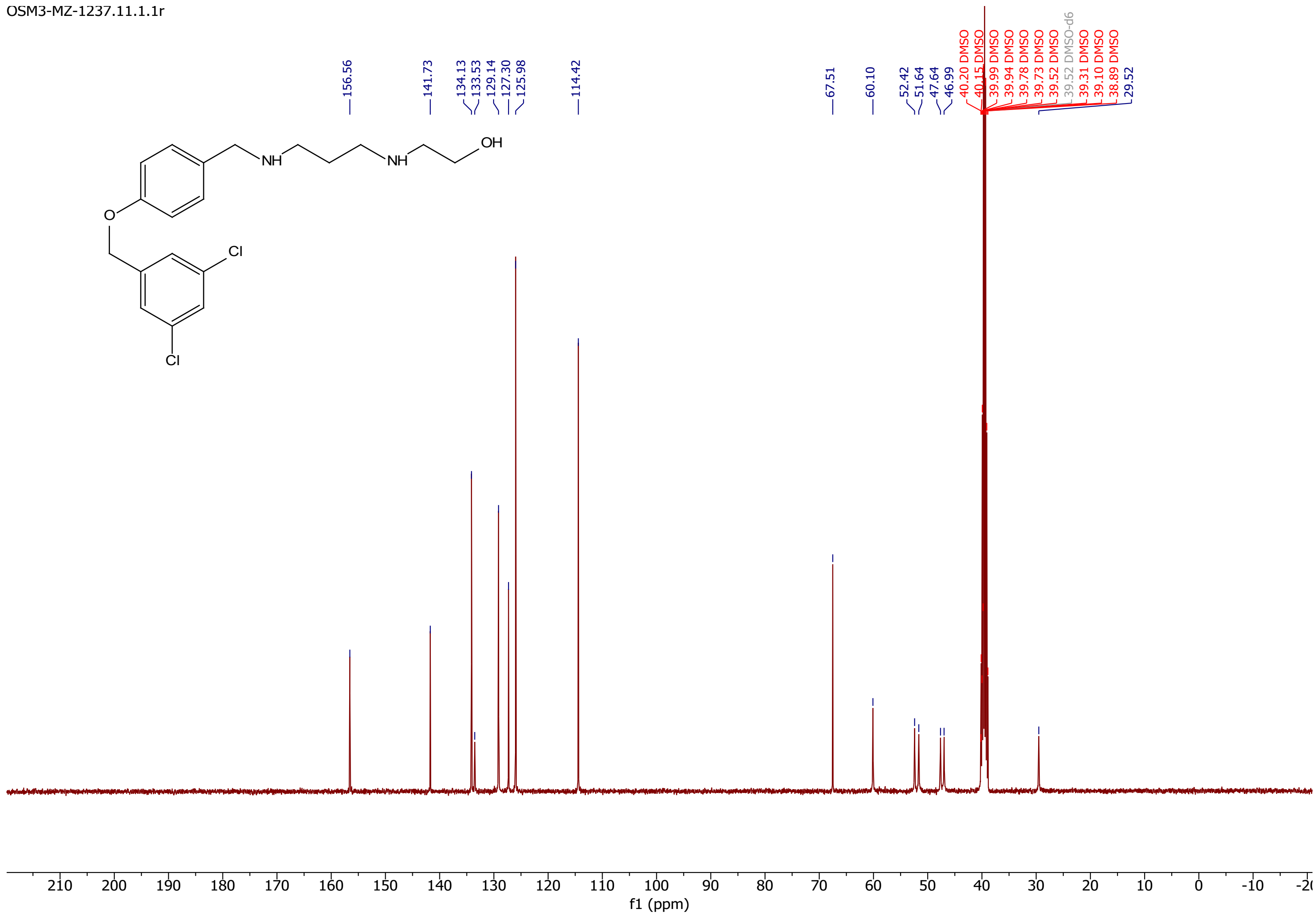
OSM3-MZ-1237.11.1.1r
156.56
141.73
134.13
133.53
129.14
127.30
125.98
114.42
67.51
60.10
52.42
51.64
47.64
46.99
40.20 DMSO
40.15 DMSO
39.99 DMSO
39.94 DMSO
39.78 DMSO
39.73 DMSO
39.52 DMSO
39.52 DMSO-d6
39.31 DMSO
39.10 DMSO
38.89 DMSO
29.52
NH
NH
OH
O
Cl
Cl
210 200 190 180 170 160 150 140 130 120 110 100 90 80 70 60 50 40 30 20 10 0 -10 -20
f1 (ppm)

2-((3-((4-((3,5-Bis(trifluoro)benzyl)oxy)benzyl)amino)propyl)amino)ethan-1-ol (***4b***).

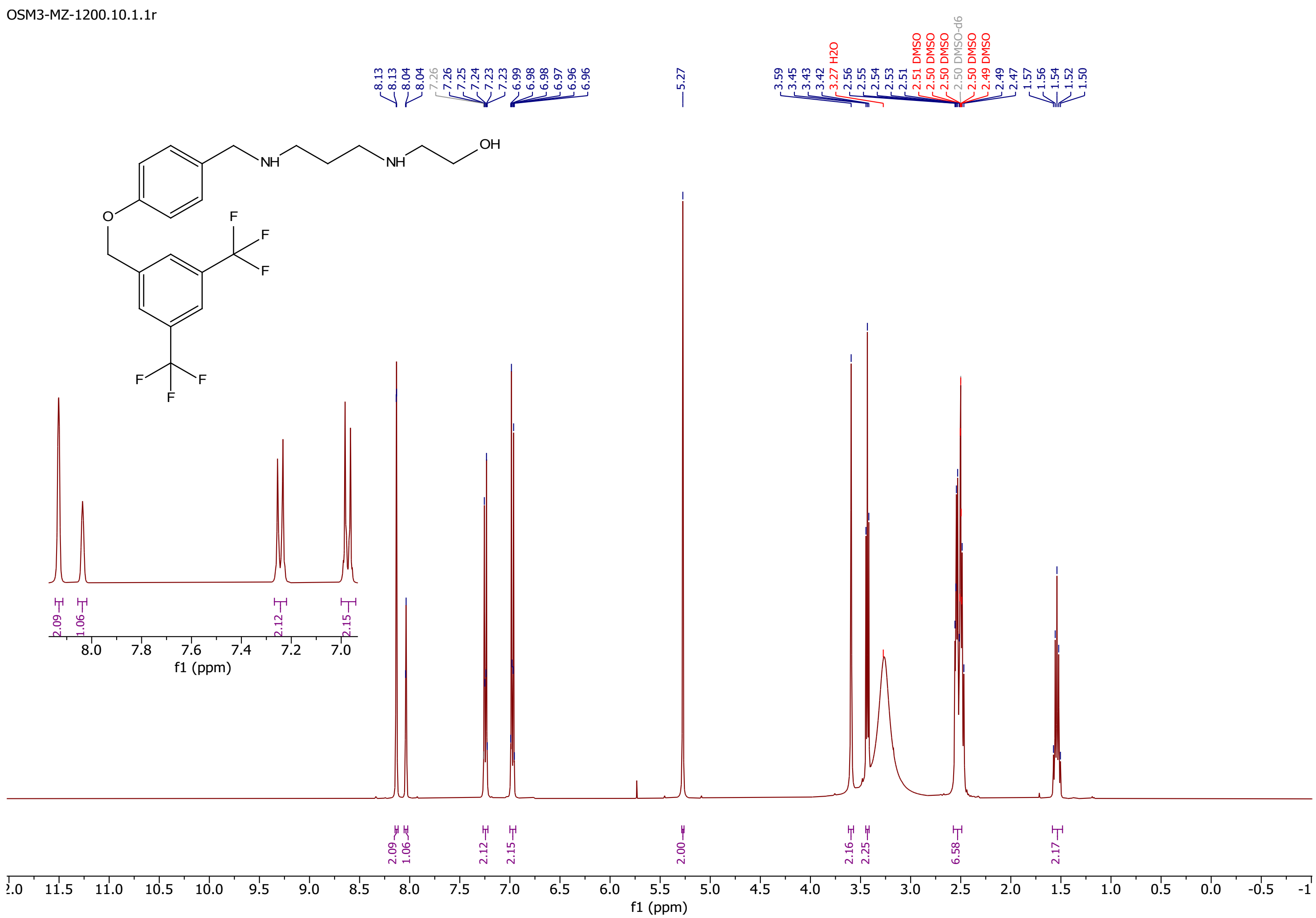

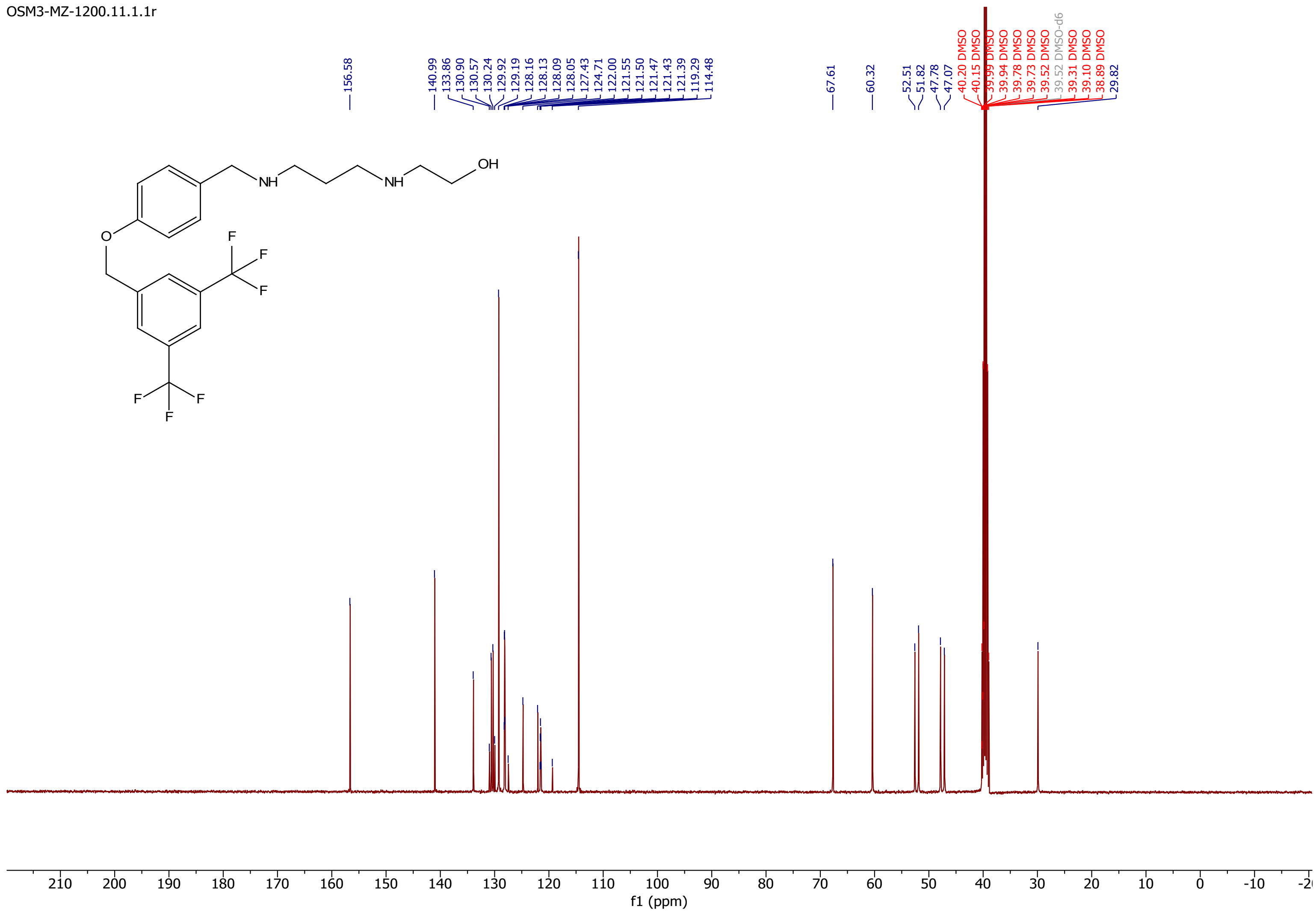
OSM3-MZ-1200.11.1.1r
156.58
140.99
133.86
130.90
130.57
130.24
129.92
129.19
128.16
128.13
128.09
128.05
127.43
124.71
122.00
121.55
121.50
121.47
121.43
121.39
119.29
114.48
67.61
60.32
52.51
51.82
47.78
47.07
40.20 DMSO
40.15 DMSO
39.99 DMSO
39.94 DMSO
39.78 DMSO
39.73 DMSO
39.52 DMSO
39.52 DMSO-d6
39.31 DMSO
39.10 DMSO
38.89 DMSO
29.82
NH
NH
OH
O
F
F
F
F
F
F
f1 (ppm)

OSM3-MZ-1200.12.1.1r

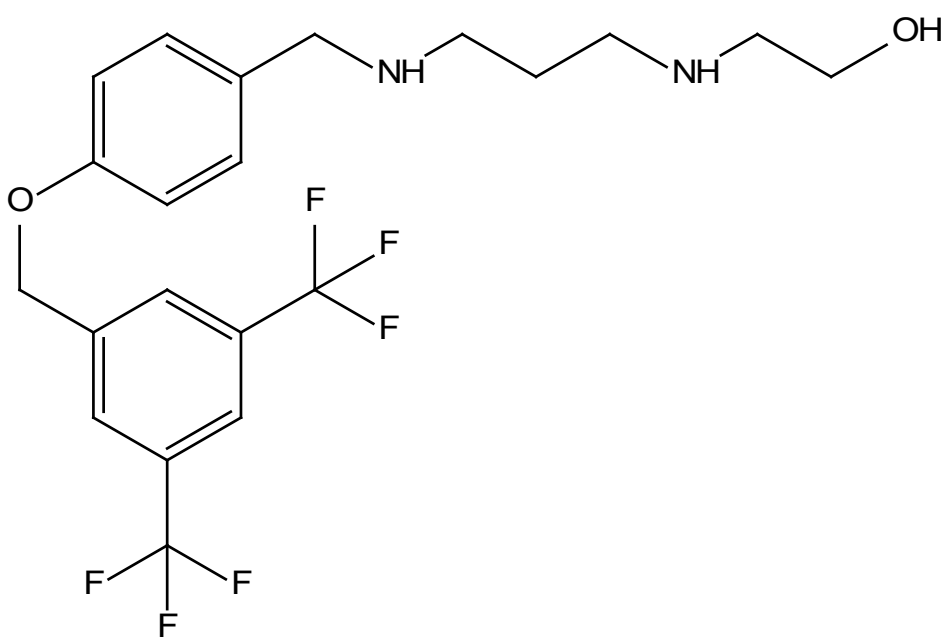

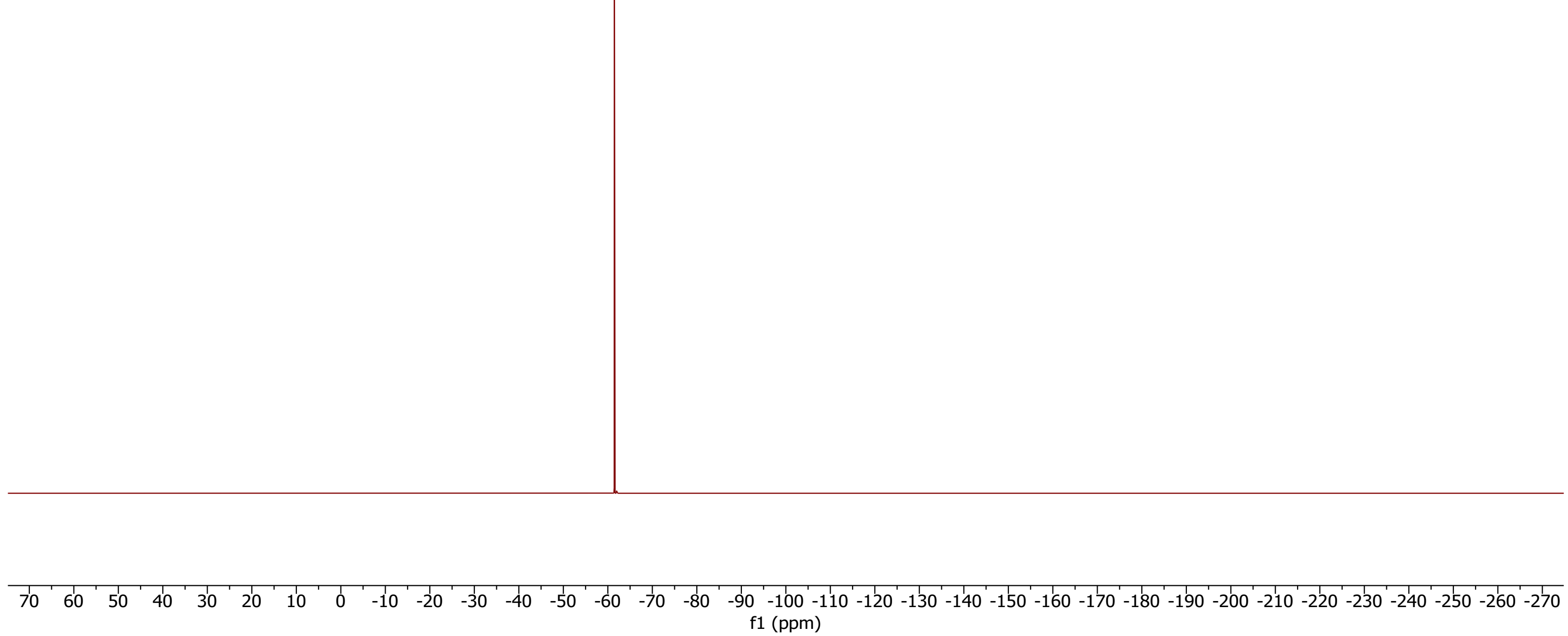

2-((3-((4-((3,5-Bis(trifluoromethyl)benzyl)oxy)-3-((4-isopropylbenzyl)oxy)benzyl)amino)propyl)amino)ethan-1-ol (***5a***).

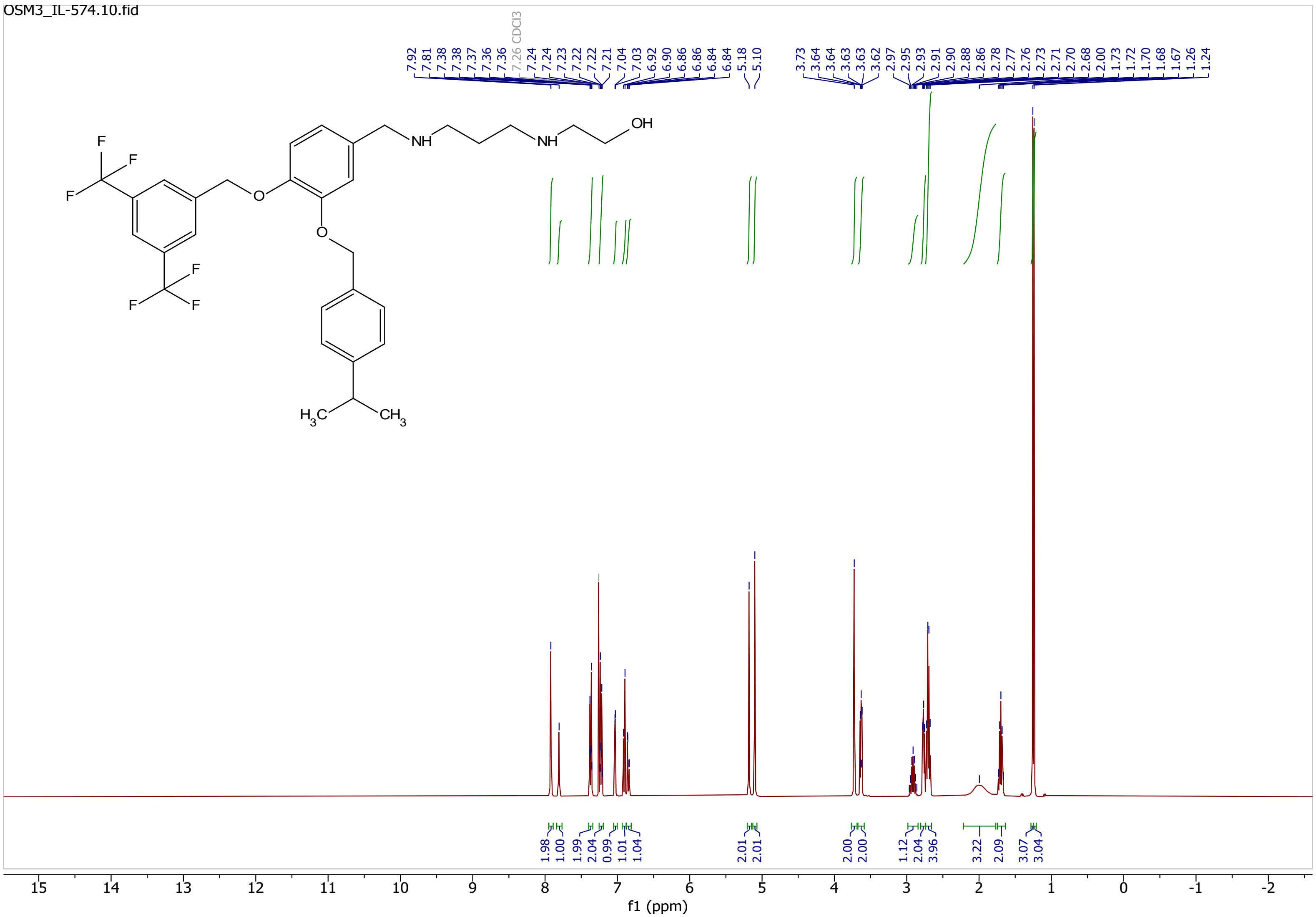

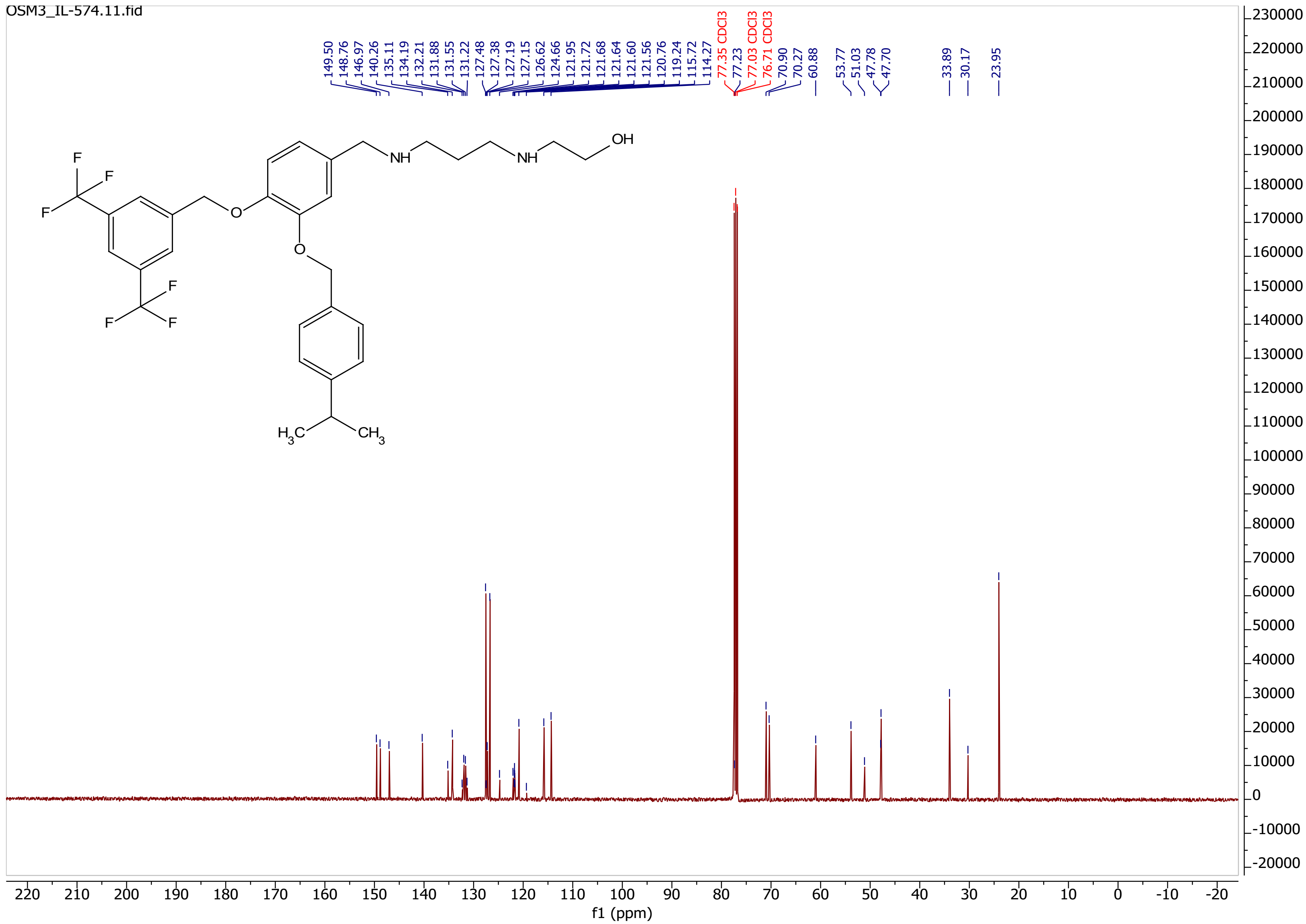
OSM3_IL-574.11.fid
149.50
148.76
146.97
140.26
135.11
134.19
132.21
131.88
131.55
131.22
127.48
127.38
127.19
127.15
126.62
124.66
121.95
121.72
121.68
121.64
121.60
121.56
120.76
119.24
115.72
114.27
77.35 CDCl3
77.23
77.03 CDCl3
76.71 CDCl3
70.90
70.27
60.88
53.77
51.03
47.78
47.70
33.89
30.17
23.95
F
F
F
F
F
F
O
O
NH
NH
OH
H3C
CH3
f1 (ppm)

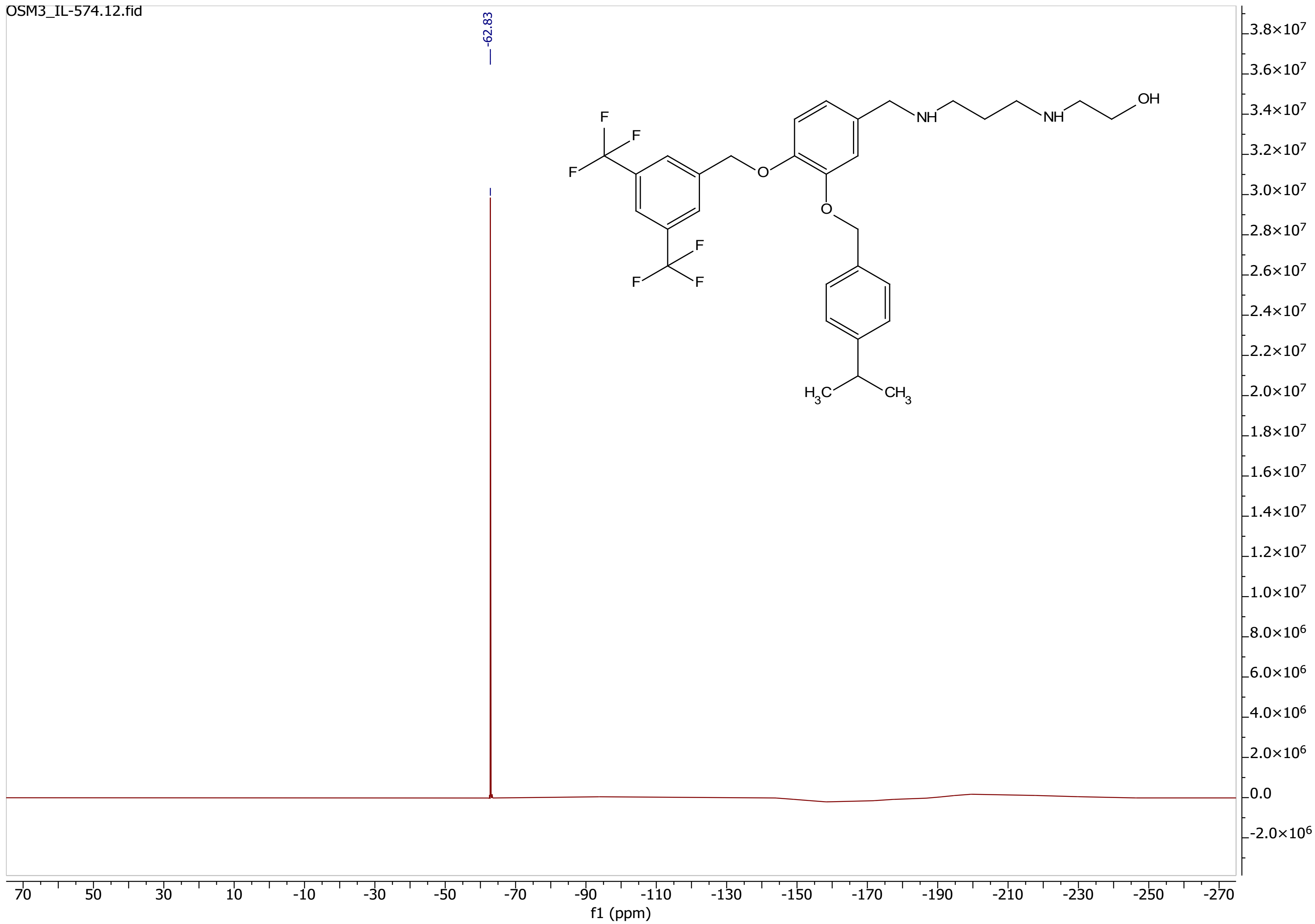
OSM3_IL-574.12.fid
-62.83
f1 (ppm)

2-((3-((3,4-bis((3,5-bis(trifluoromethyl)benzyl)oxy)benzyl)amino)propyl)amino)ethan-1-ol (***5b***)**.**

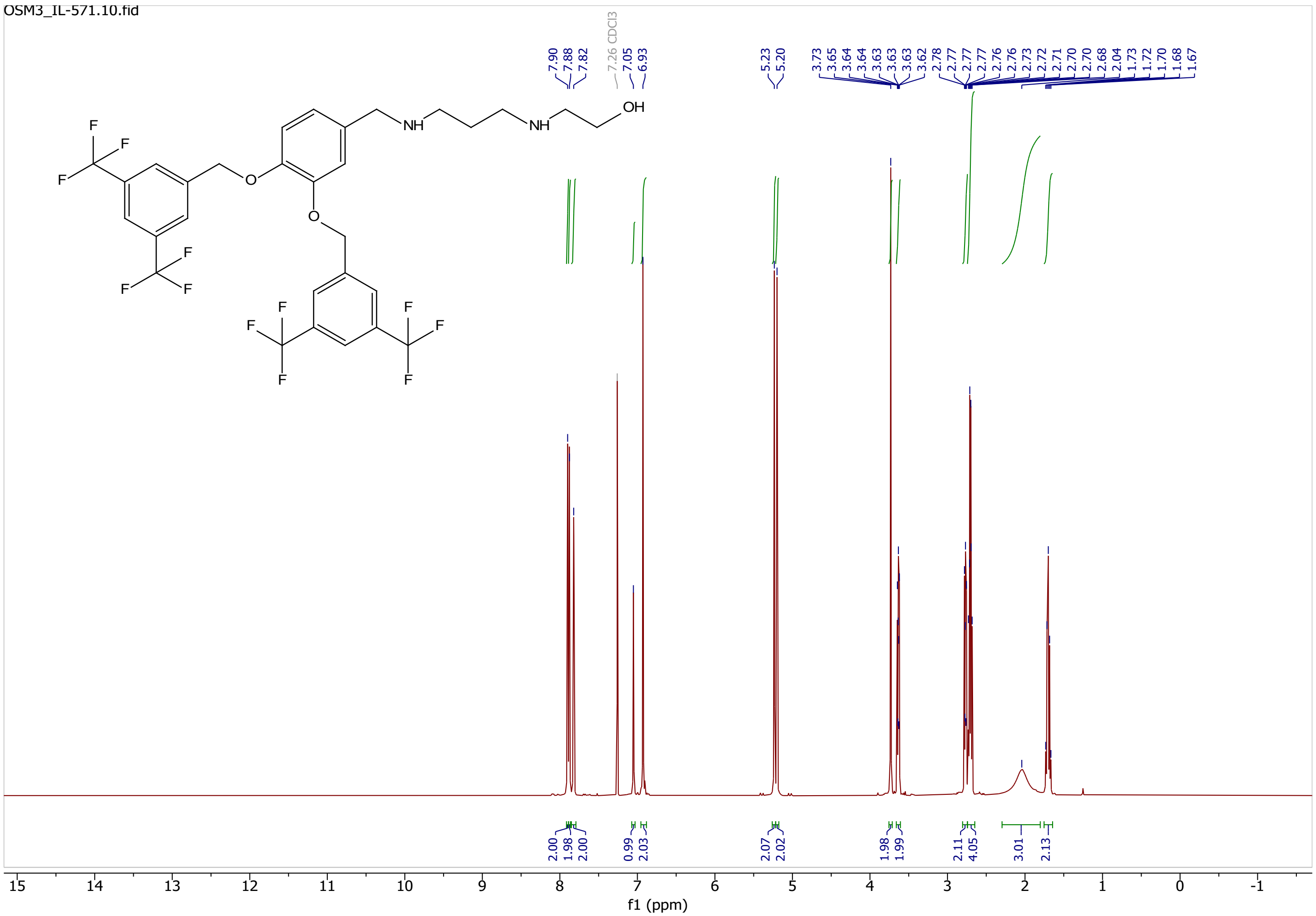

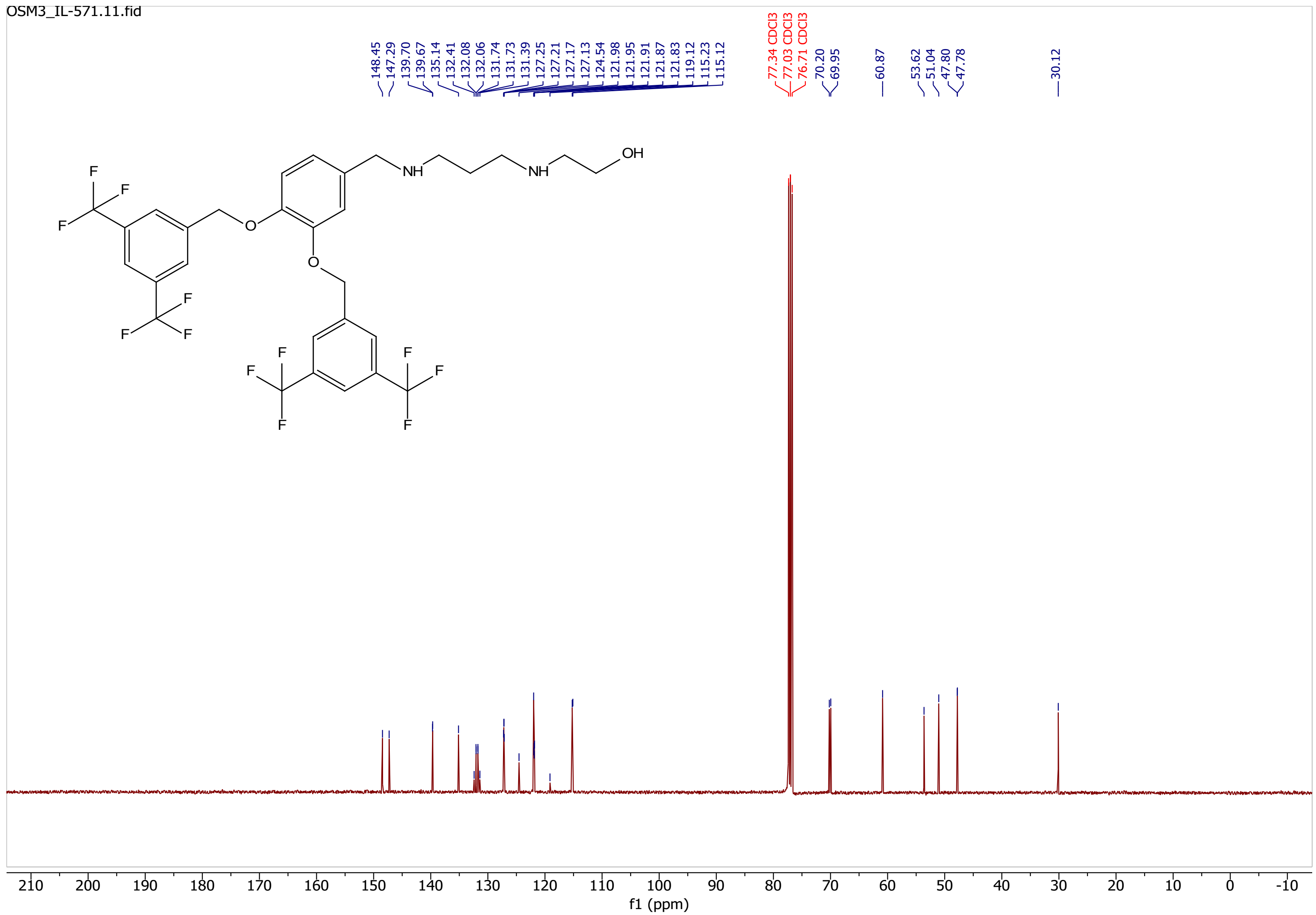
OSM3_IL-571.11.fid
148.45
147.29
139.70
139.67
135.14
132.41
132.08
132.06
131.74
131.73
131.39
127.25
127.21
127.17
127.13
124.54
121.98
121.95
121.91
121.87
121.83
119.12
115.23
115.12
77.34 CDCl3
77.03 CDCl3
76.71 CDCl3
70.20
69.95
60.87
53.62
51.04
47.80
47.78
30.12
F
F
F
F
F
F
F
F
F
F
F
F
O
O
NH
NH
OH
210
200
190
180
170
160
150
140
130
120
110
100
90
80
70
60
50
40
30
20
10
0
-10
f1 (ppm)

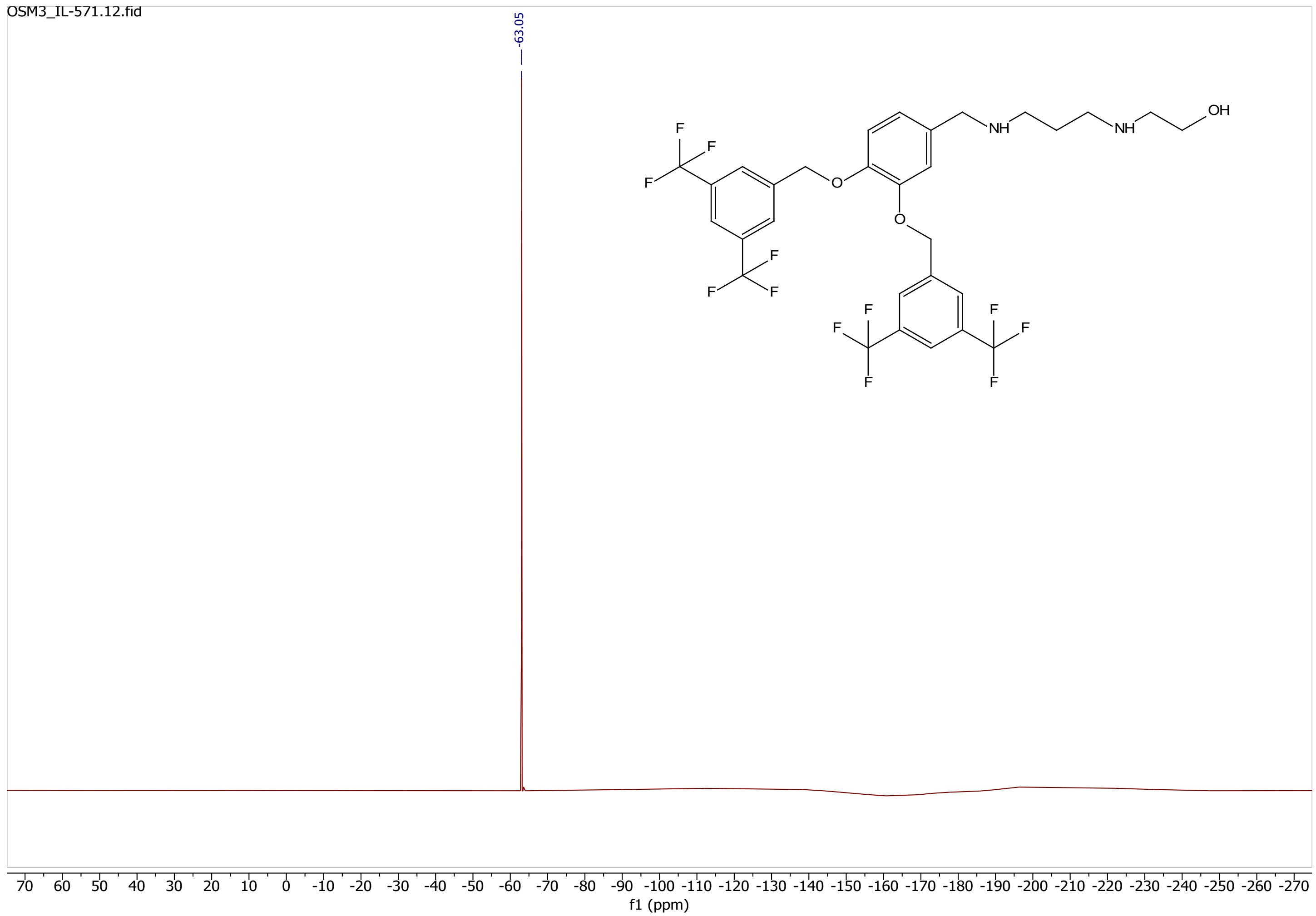
OSM3_IL-571.12.fid
-63.05
F
F
F
F
F
F
O
O
NH
NH
OH
F
F
F
F
F
F
70 60 50 40 30 20 10 0 -10 -20 -30 -40 -50 -60 -70 -80 -90 -100 -110 -120 -130 -140 -150 -160 -170 -180 -190 -200 -210 -220 -230 -240 -250 -260 -270
f1 (ppm)

2-((3-((4-((3,5-Bis(trifluoromethyl)benzyl)oxy)-3-((2,3-dichlorobenzyl)oxy)benzyl)amino)propyl)amino)ethan-1-ol (***5c***)**.**

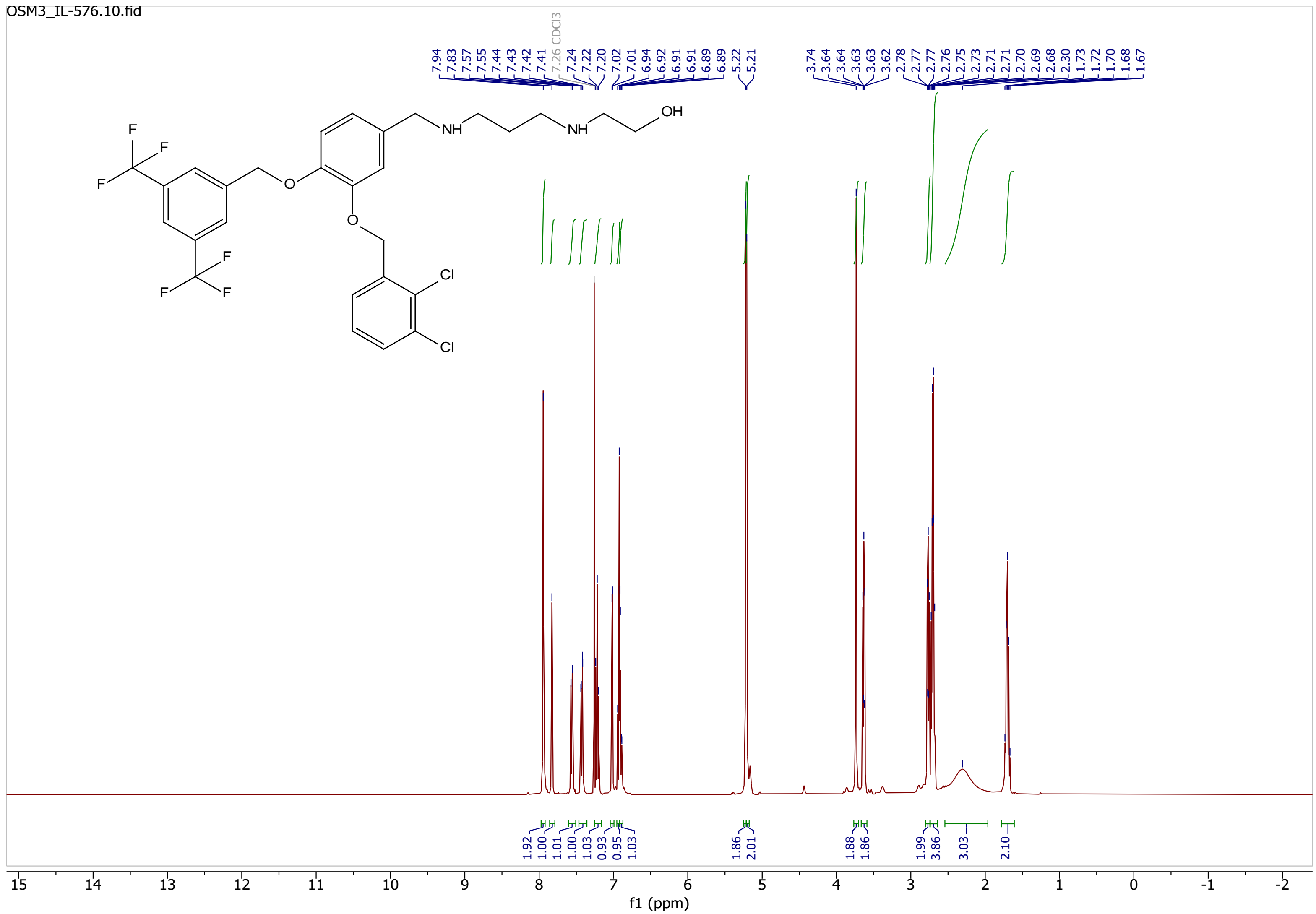

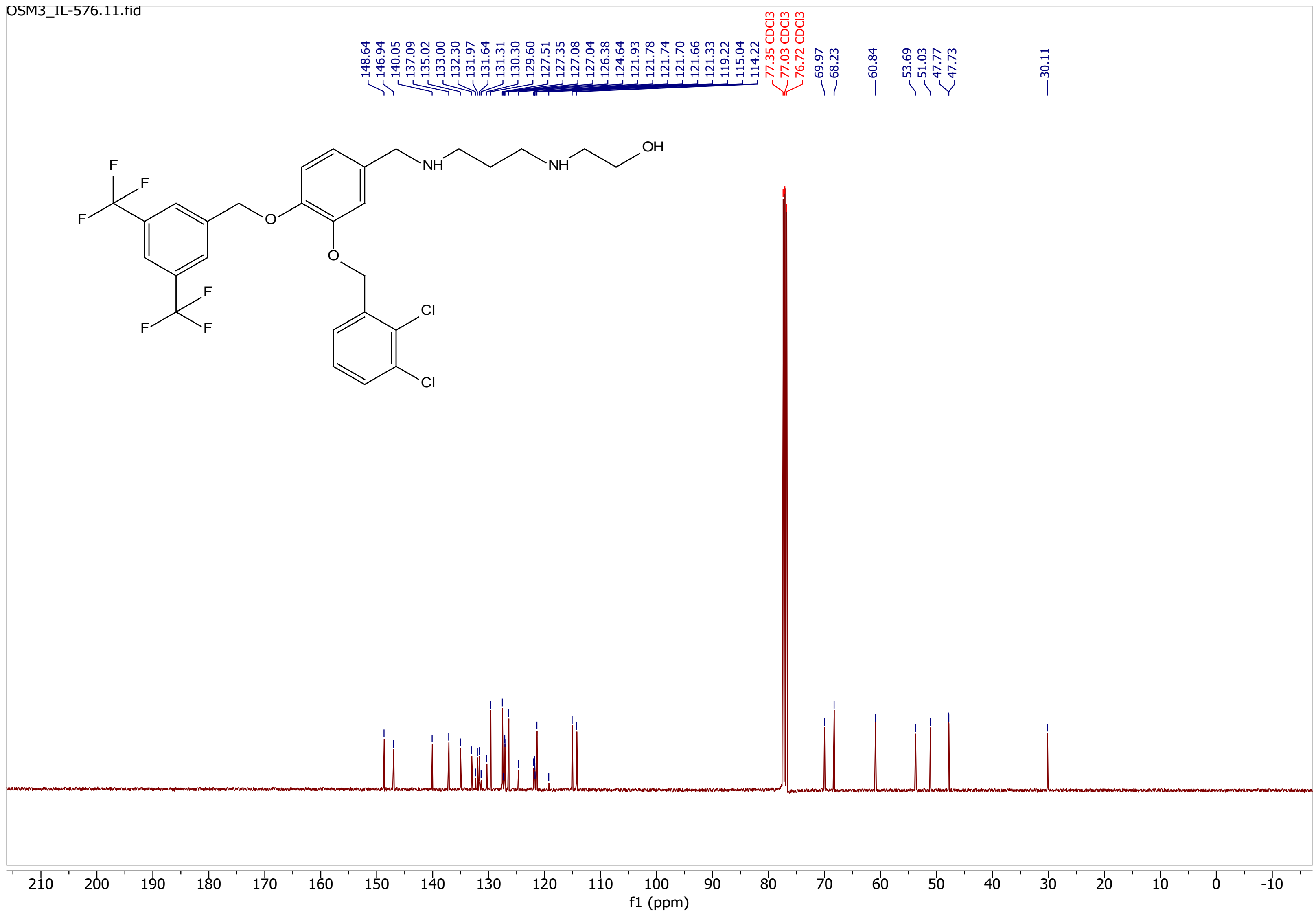

OSM3_IL-576.11.fid
148.64
146.94
140.05
137.09
135.02
133.00
132.30
131.97
131.64
131.31
130.30
129.60
127.51
127.35
127.08
127.04
126.38
124.64
121.93
121.78
121.74
121.70
121.66
121.33
119.22
115.04
114.22
77.35 CDCl3
77.03 CDCl3
76.72 CDCl3
69.97
68.23
60.84
53.69
51.03
47.77
47.73
30.11
F
F
F
O
NH
NH
OH
O
F
F
F
Cl
Cl
210
200
190
180
170
160
150
140
130
120
110
100
90
80
70
60
50
40
30
20
10
0
-10
f1 (ppm)

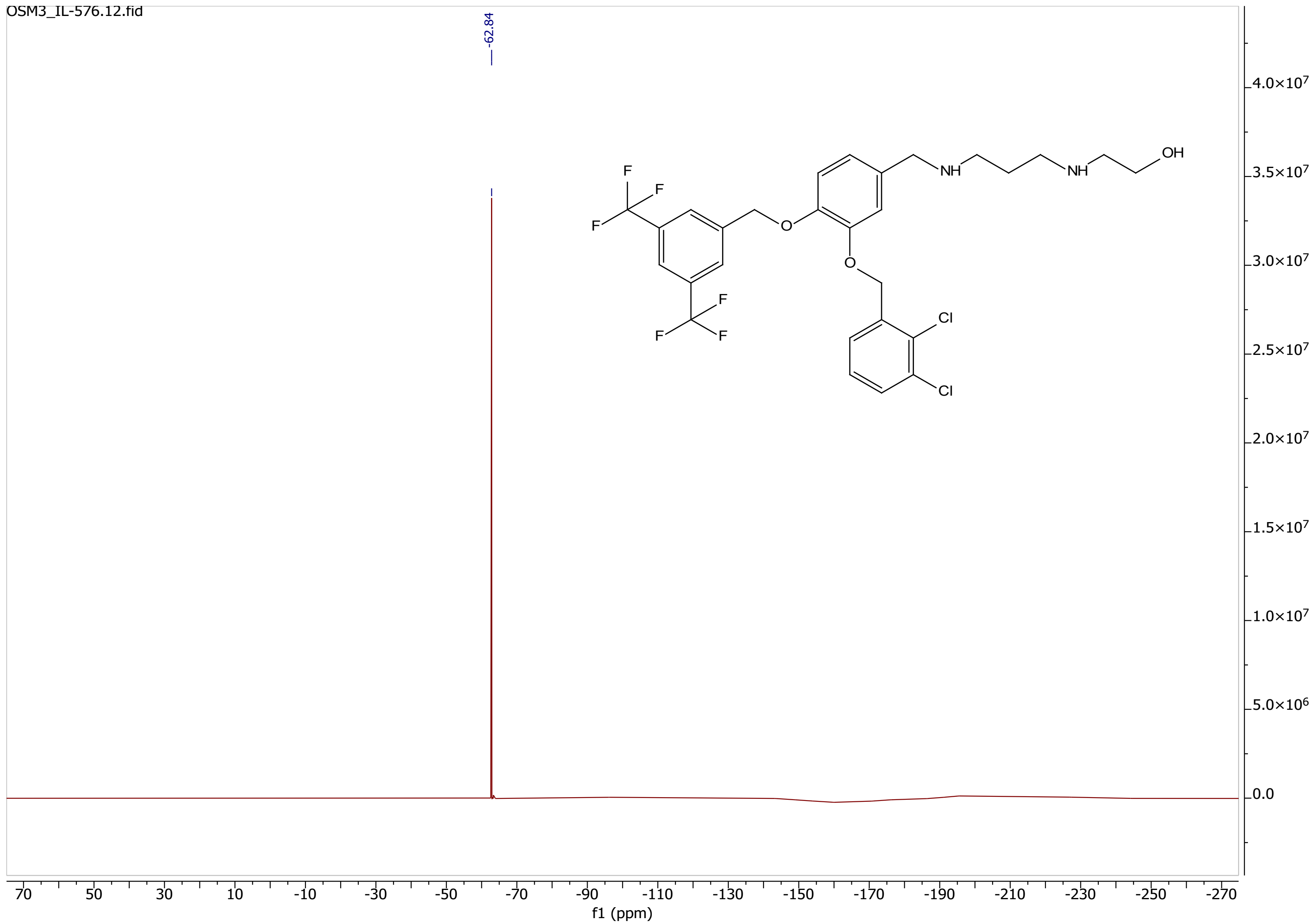
OSM3_IL-576.12.fid
-62.84
F
F
F
F
F
F
O
O
NH
NH
OH
Cl
Cl
4.0×10^7
3.5×10^7
3.0×10^7
2.5×10^7
2.0×10^7
1.5×10^7
1.0×10^7
5.0×10^6
0.0
70 50 30 10 -10 -30 -50 -70 -90 -110 -130 -150 -170 -190 -210 -230 -250 -270
f1 (ppm)

2-((3-((4-((3,5-Bis(trifluoromethyl)benzyl)oxy)-3-((3,4-dichlorobenzyl)oxy)benzyl)amino)propyl)amino)ethan-1-ol (***5d***).

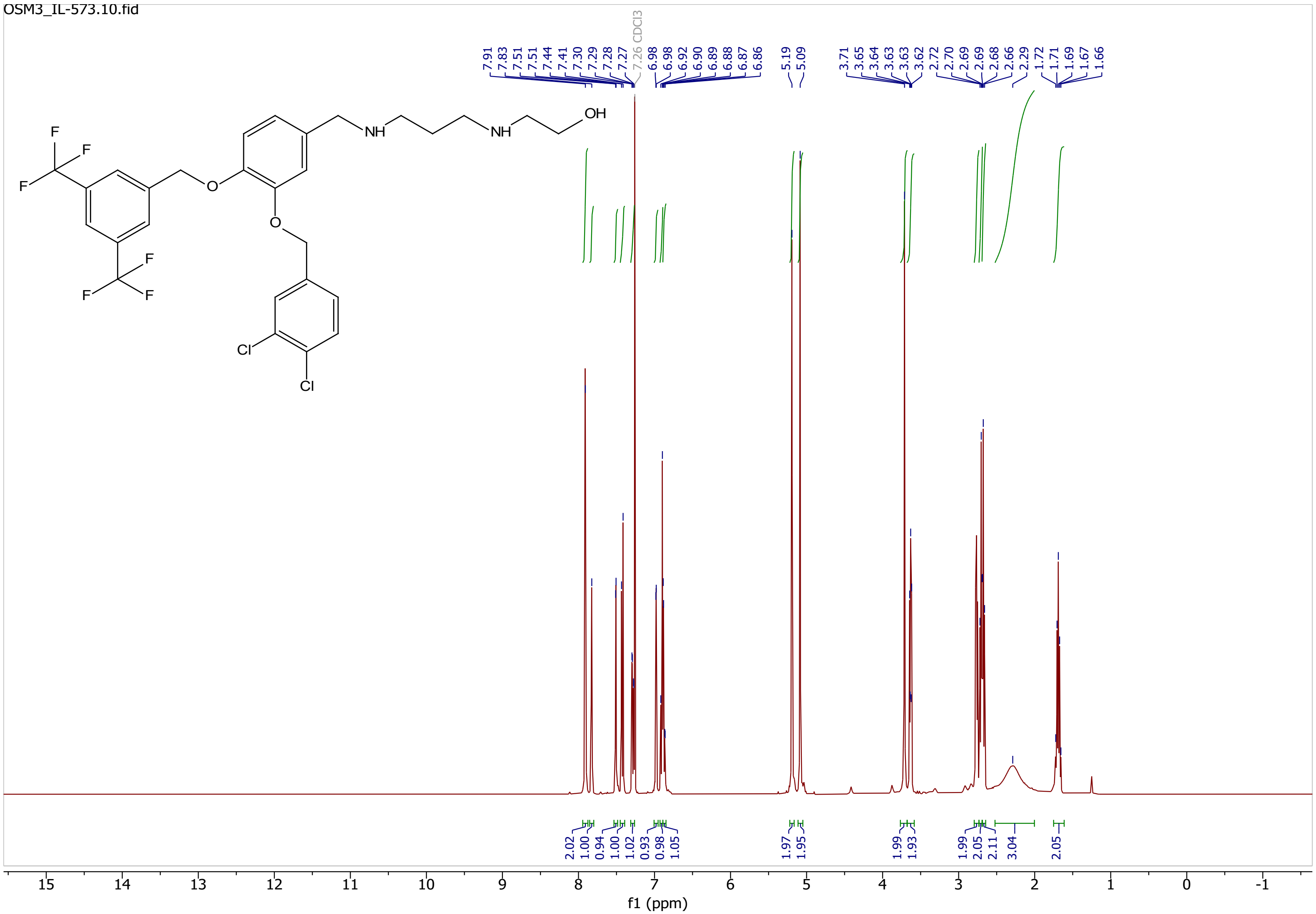

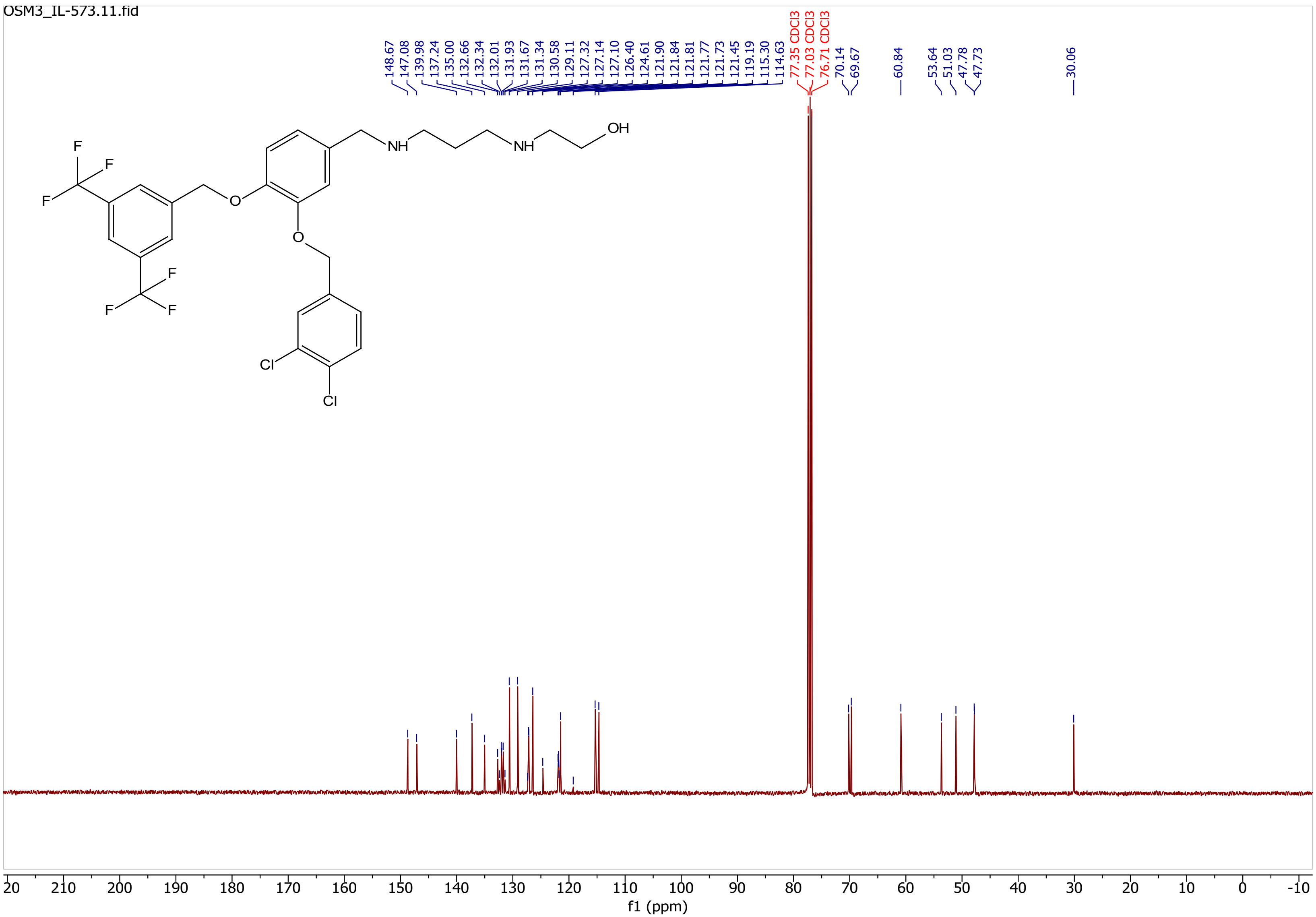
OSM3_IL-573.11.fid
148.67
147.08
139.98
137.24
135.00
132.66
132.34
132.01
131.93
131.67
131.34
130.58
129.11
127.32
127.14
127.10
126.40
124.61
121.90
121.84
121.81
121.77
121.73
121.45
119.19
115.30
114.63
77.35 CDCl3
77.03 CDCl3
76.71 CDCl3
70.14
69.67
60.84
53.64
51.03
47.78
47.73
30.06
F
F
F
F
F
F
O
O
NH
NH
OH
Cl
Cl
220 210 200 190 180 170 160 150 140 130 120 110 100 90 80 70 60 50 40 30 20 10 0 -10
f1 (ppm)

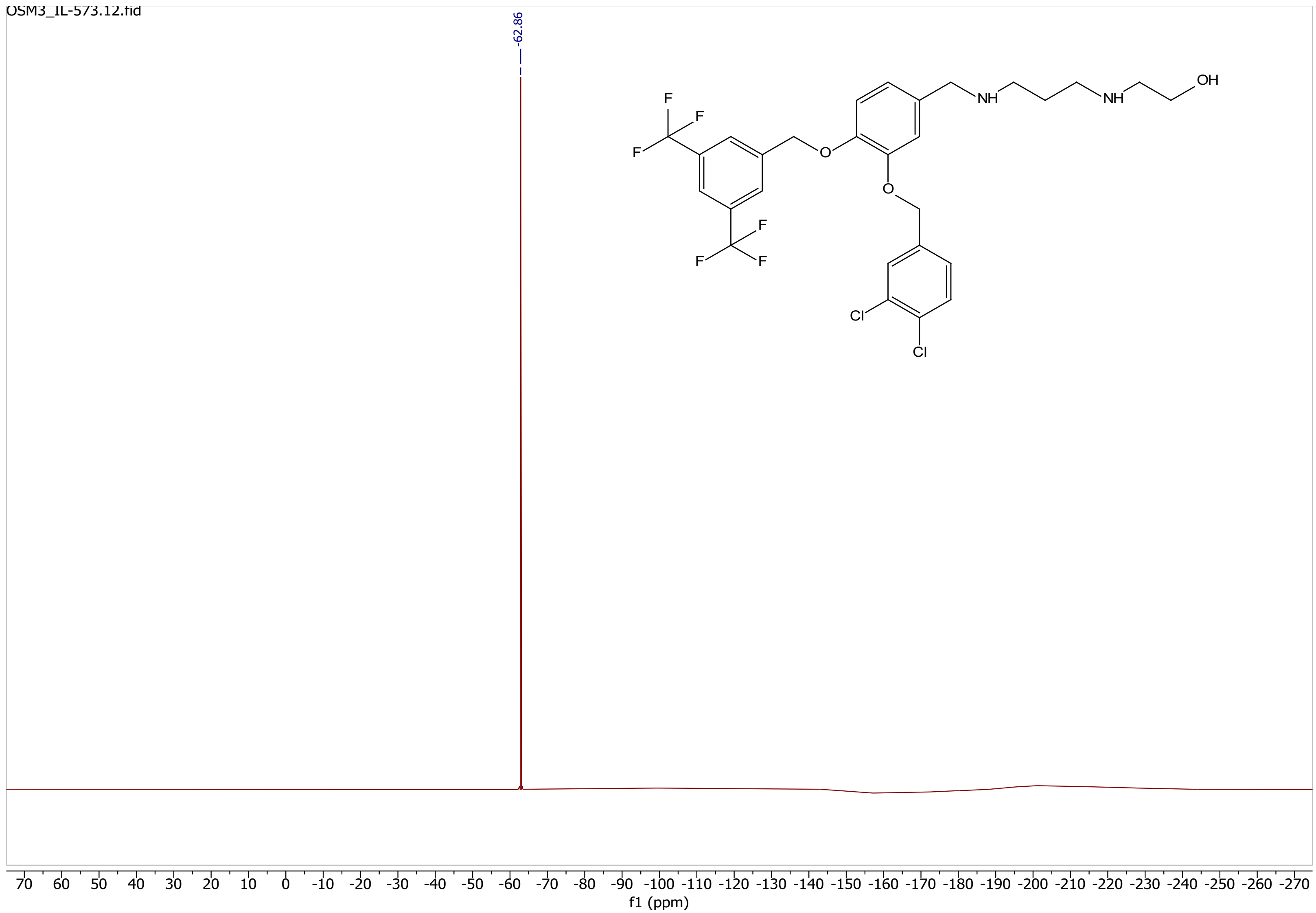
OSM3_IL-573.12.fid
-62.86
F
F
F
F
F
F
O
O
NH
NH
OH
Cl
Cl
70 60 50 40 30 20 10 0 -10 -20 -30 -40 -50 -60 -70 -80 -90 -100 -110 -120 -130 -140 -150 -160 -170 -180 -190 -200 -210 -220 -230 -240 -250 -260 -270
f1 (ppm)

2 Supplementary Figures and Tables

2.1 Supplementary Tables

Table S1. Selected Compounds for Experimental Testing

Compound ID	MolPort ID	Vendor	Catalogue #	H33342 accumulation (% average ± SD)[a]
**S1**	n.a.	n.a.	n.a.	73.6 ± 1.4
**S2**	MolPort-002-132-505	ChemDiv	1829-0095	44.57 ± 1.4
**S3**	MolPort-044-335-591	ChemDiv	N121-0620	68.1 ± 0.4
**S4**	MolPort-028-836-970	Enamine	Z900472518	67.1 ±0.8
**S5**	MolPort-035-945-594	Otava	7128132	78.3 ± 2.9
**S6**	MolPort-002-539-399	Vitas-M Laboratory	STL322022	42.3 ± 0.4
**S7**	MolPort-000-841-426	Vitas-M Laboratory	STK613362	14.6 ± 0.4
**S8**	MolPort-009-452-984	Enamine	Z232690672	61.7 ± 0.1
**S9**	MolPort-046-418-742	BIONET/Key Organics	AS-66956	48.7 ± 0.9
**S10**	MolPort-002-966-614	Vitas-M Laboratory	STK182840	77.8 ± 6.5
**S11 (1a)**	n.a.	n.a.	n.a.	142.1 ± 1.4

n.a.: not available (see synthesis protocols); the purity of compounds S2-S11 is >90 % according to information from vendor.

[a] Compounds were tested at 100 µM. The accumulation of H33342 is expressed as a percentage and standard deviation in relation to tolc-deficient strain *E. coli* JW5503. Values obtained in relative fluorescence units were converted into percentage and values for *E. coli* JW5503 were attributed as 100%. Experiments were performed once in duplicate.

Table S2. Modification of substructure A.

Compound	Structure, R	MPC4[a] PIP (µM)	MIC[b] (µM)	MIC / MPC4	H33342 accumulation (% average ± SD)
**1a**	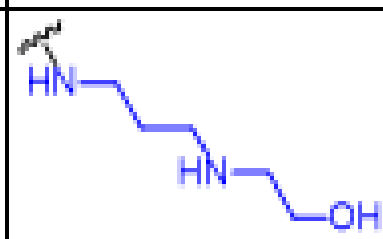	75	>125	>1.67	142.1 ± 1.4
**1b**	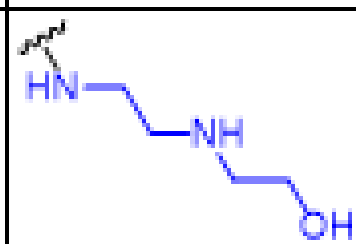	>125	n.d.	n.d.	92.6 ± 1.5
**1c**	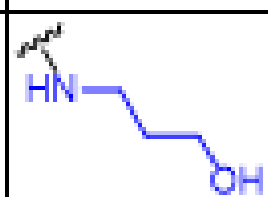	>125	n.d.	n.d.	82.8 ± 0.5

[a] MPC4 PIP: Minimum Potentiating Concentration was defined as the concentration of compound that decreased MIC of piperacillin by 4-fold (i.e., from 4 µg/mL to 1µg/mL) in *E. coli* BW25113. If MPC4 was not achieved, the value is represented by the highest concentration of compound tested.

[b] MIC: Minimum Inhibitory Concentration was defined as the concentration of compound that inhibited *E. coli* BW25113 growth by ≥90%. If MIC was not achieved, the value is represented by the highest concentration of compound tested.

Table S3. Modification of substructure B at the middle benzene ring.

Compound	Structure, R	MPC4[a] PIP (µM)	MIC[b] (µM)	MIC/MPC4	H33342 accumulation (% average ± SD)
**1a**	Me-	75	>125	>1.67	142.1 ± 1.4
**2d**	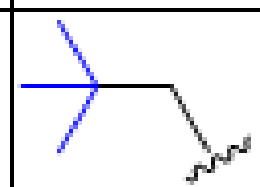	12.5	12.5	1	148.7 ± 1.8
**2e**	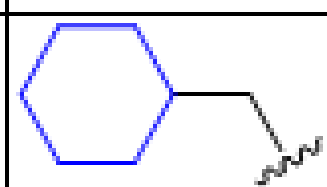	6.25	6.25	1	135.1 ± 3.7

[a] MPC4 PIP: Minimum Potentiating Concentration was defined as the concentration of compound that decreased MIC of piperacillin by 4-fold (i.e., from 4 µg/mL to 1µg/mL) in *E. coli* BW25113. If MPC4 was not achieved, the value is represented by the highest concentration of compound tested.

[b] MIC: Minimum Inhibitory Concentration was defined as the concentration of compound that inhibited *E. coli* BW25113 growth by ≥90%. If MIC was not achieved, the value is represented by the highest concentration of compound tested.

Table S4. Substructure C modifications connected via an oxymethylene group to substructure B.

Compound	Structure, R	MPC_4^a PIP (µM)	MIC^b (µM)	MIC/MPC_4	H33342 accumulation (% average ± SD)
**1a**	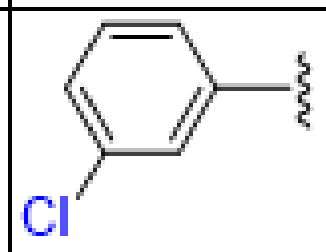	75	>125	>1.67	142.1 ± 1.4
**3a**	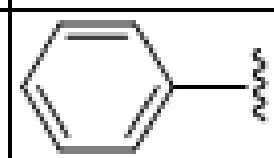	>125	n.d.	n.d.	82.6 ± 0.7
**3b**	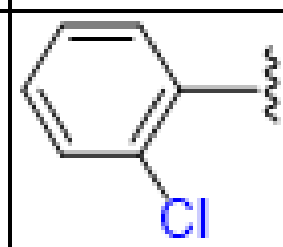	100	>125	>1.25	93.0 ± 0.4
**3c**	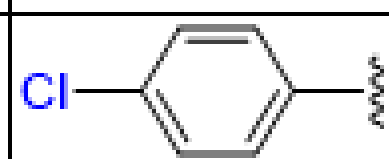	100	>125	>1.25	101.7 ± 0.7
**3d**	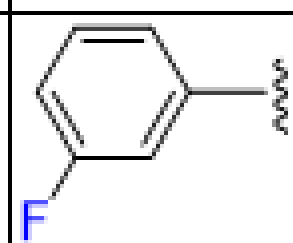	>125	n.d.	n.d.	98.9 ± 0.9
**3f**	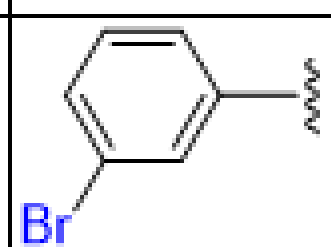	75	>125	>1.67	94.0 ± 0.7
**3h**	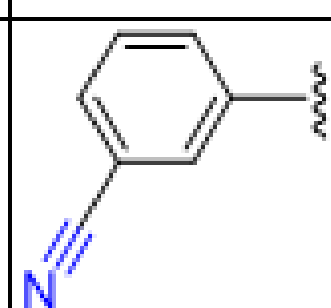	>125	n.d.	n.d.	88.1 ± 1.4
**3i**	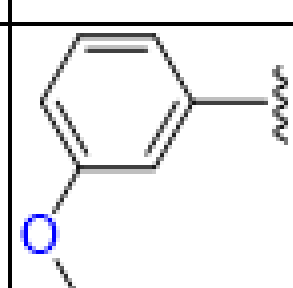	>125	n.d.	n.d.	87.1 ± 1.0
**3j**	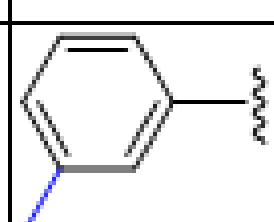	125	>125	>1	104.6 ± 1.6
**3n**	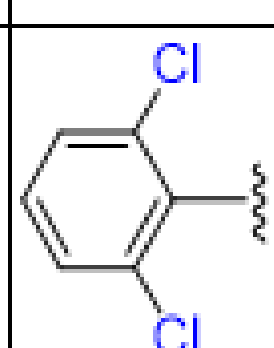	75	>125	>1.67	90.9 ± 5.4
**3t**	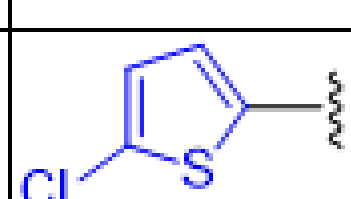	75	>125	>1.67	103.6 ± 0.7

**3v**	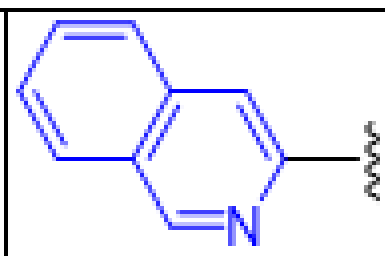	>125	n.d.	n.d.	91.0 ± 0.3
**3w**	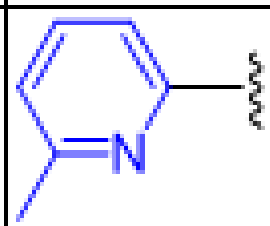	>125	n.d.	n.d.	122.4 ± 0.3

[a] MPC_4 PIP: Minimum Potentiating Concentration was defined as the concentration of compound that decreased MIC of piperacillin by 4-fold (i.e., from 4 µg/mL to 1µg/mL) in *E. coli* BW25113. If MPC_4 was not achieved, the value is represented by the highest concentration of compound tested.

[b] MIC: Minimum Inhibitory Concentration was defined as the concentration of compound that inhibited *E. coli* BW25113 growth by ≥90%. If MIC was not achieved, the value is represented by the highest concentration of compound tested.

Table S5. Inhibition of *E. coli* BW25113 growth

Compounds	Percentage of bacterial growth inhibition (±SD)[a]	
	Alone	In combination with piperacillin[b]
**1a**	25.91 (±0.11)	n.a.
**1b**	n.d.	41.55 (±38.20)
**1c**	n.d.	21.37 (±15.21)
**2a**	56.08 (±5.05)	n.a.
**3a**	n.d.	9.68 (±30.12)
**3b**	21.62 (±0.62)	n.a.
**3c**	33.58 (±1.45)	n.a.
**3d**	n.d.	23.36 (±46.26)
**3e**	41.12 (±0.56)	n.a.
**3f**	35.84 (±3.37)	n.a.
**3g**	56.44 (±3.02)	n.a.
**3h**	n.d.	29.19 (±24.20)
**3i**	n.d.	50.14 (±7.07)
**3j**	16.78 (±3.06)	n.a.
**3k**	28.86 (±5.74)	n.a.
**3n**	4.17 (±7.28)	n.a.
**3o**	78.90 (±5.13)	n.a.
**3q**	52.19 (±14.58)	n.a.
**3r**	84.80 (±2.24)	n.a.
**3t**	21.70 (±7.48)	n.a.
**3u**	35.79 (±3.75)	n.a.
**3v**	n.d.	n.a.
**3w**	n.d.	n.a.
**PAβN**[c]	32.12 (±14.18)	n.a.

[a]Compounds at 125 μM, unless otherwise specified.

[b]Piperacillin at 1 μg/mL

[c]PAβN at 50 μg/mL

n.a.: MPC was achieved at ≤125 μM. Results are presented in the main manuscript.

n.d.: Potentiation was not achieved; thus, MIC of compound alone was not determined

2.2 Supplementary Figures

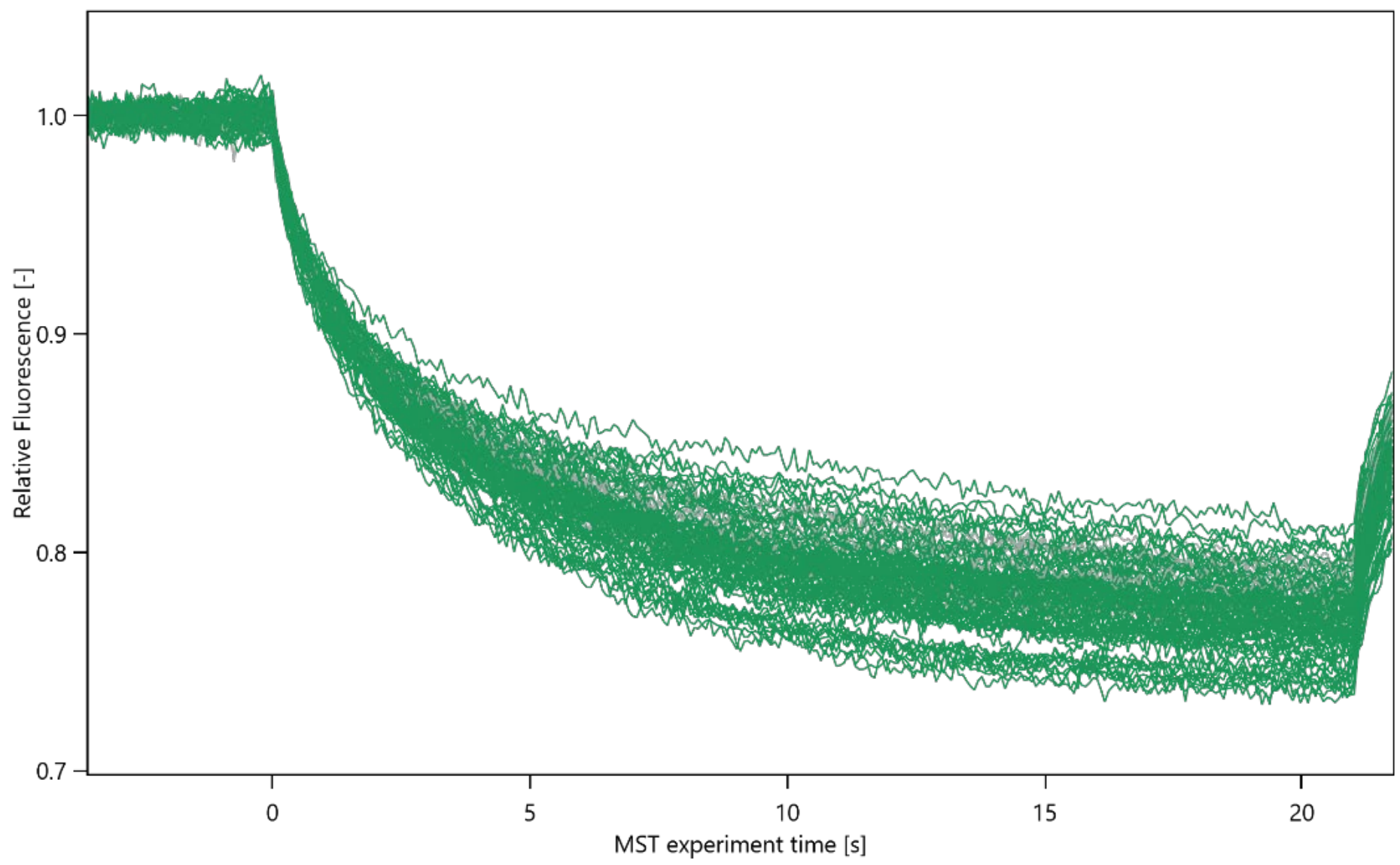

Figure S1. MST Traces obtained by microscale thermophoresis using His-tagged TolC in presence of compound **1a** normalized on the initial fluorescence signal. Traces from six experiments are included in the diagram, excluded traces are colored grey.

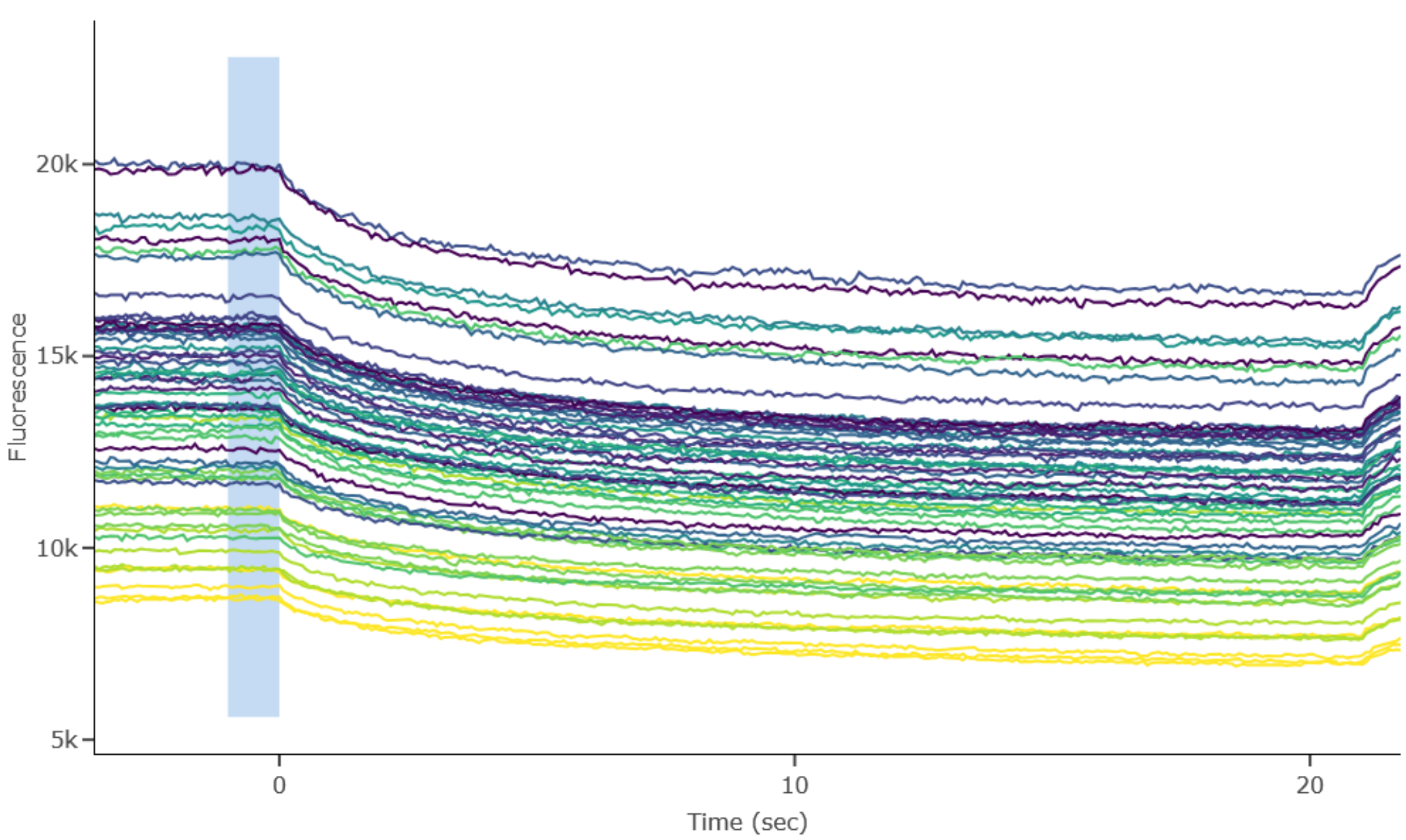

Figure S2. MST Traces obtained by microscale thermophoresis using His-tagged AcrB in presence of compound **1a**. Traces from five experiments are included in the diagram and colored according to the compound concentration (yellow (1000 µM) to violet (0.5 µM). The blue range marks the signals used for analysis.

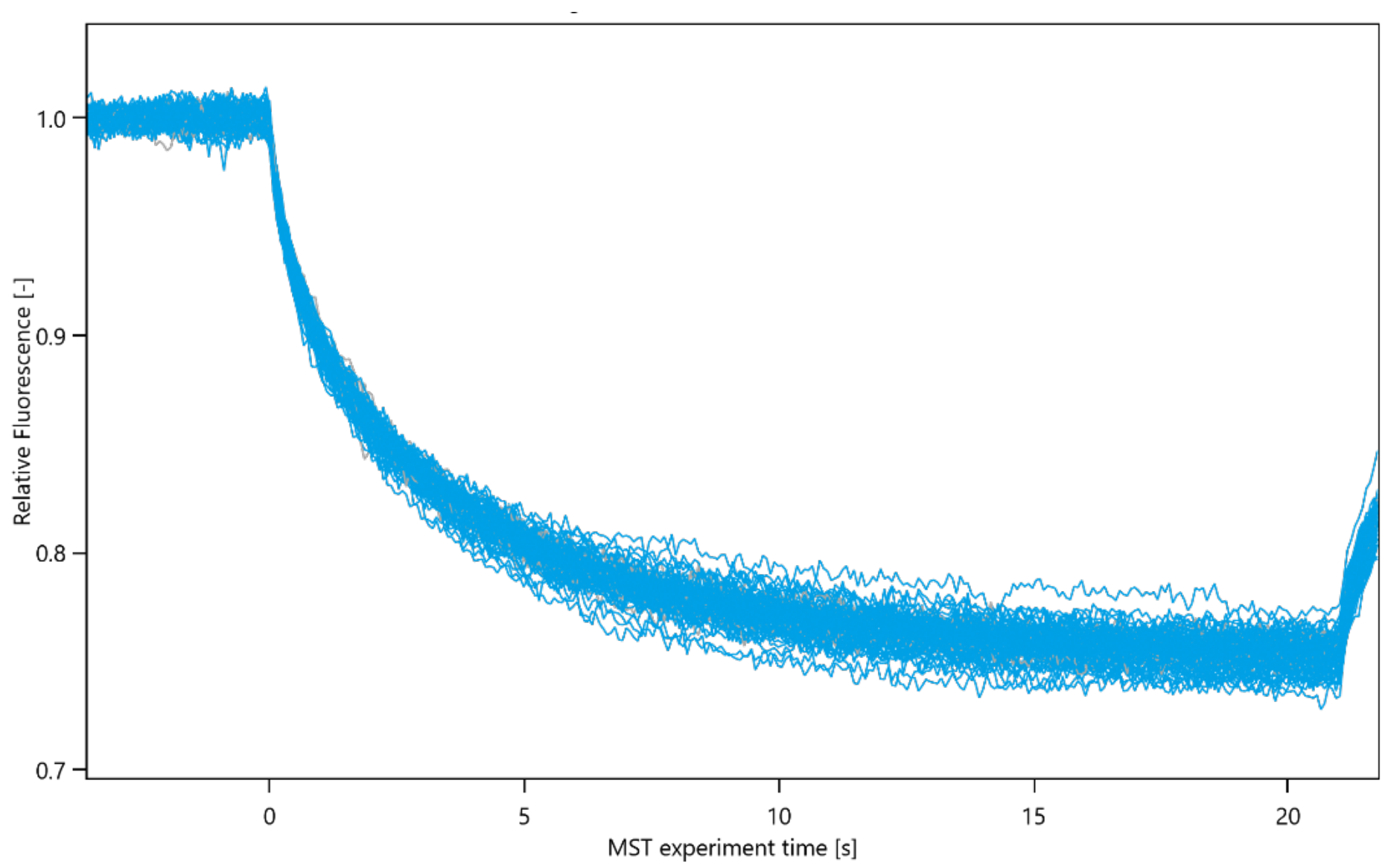

Figure S3. MST Traces obtained by microscale thermophoresis using His-tagged AcrA in presence of compound **1a** normalized on the initial fluorescence signal. Traces from five experiments are included in the diagram, excluded traces are colored grey.

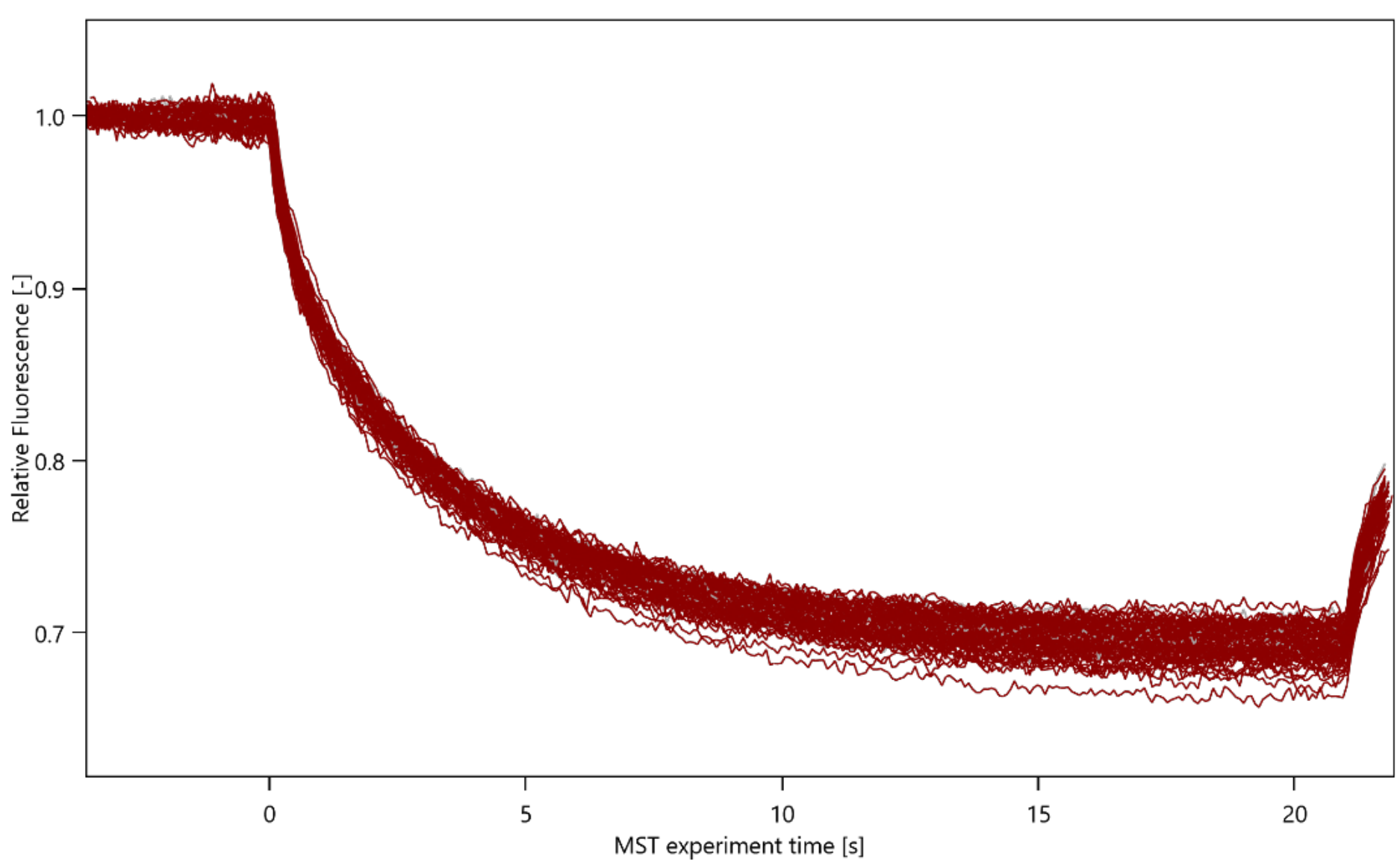

Figure S4. MST Traces obtained by microscale thermophoresis using His-tagged TEVp in presence of compound **1a** normalized on the initial fluorescence signal. Traces from five experiments are included in the diagram, excluded traces are colored grey.

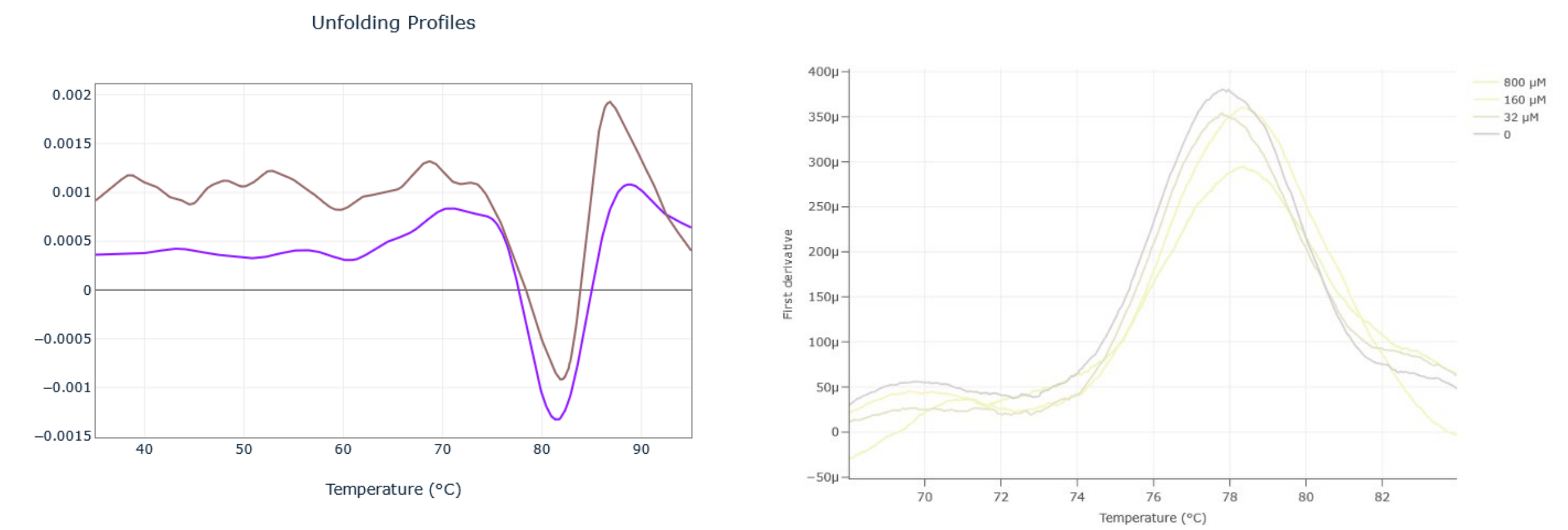

Figure S5. Left: First derivative of the melting curve of 5 µM TolC with 1 mM concentration of compound **1a** (violet) and in absence of any compound (brown), measured using a Tycho (NanoTemper Technologies), detecting the ratio between the protein fluorescence at 330 and 350 nm. Right: First derivative of the melting curves of 5 µM TolC with different concentrations of **1a** and in absence of any compound (grey), measured using a Prometheus (NanoTemper Technologies), heating 1°C per minute and detecting the ratio between the protein fluorescence at 330 and 350 nm. The plot was generated using the MoltenProt tool at the eSPC platform.

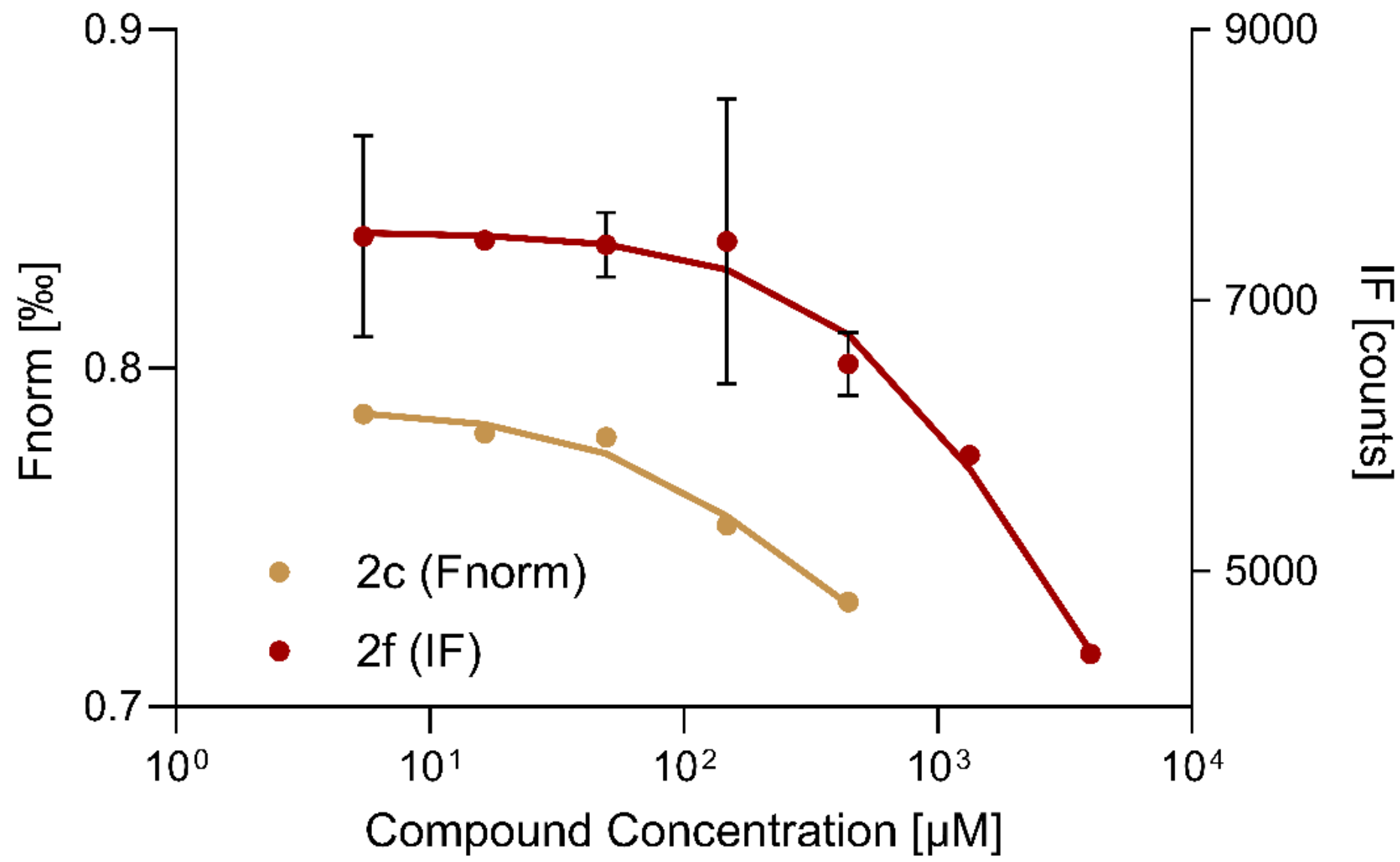

Figure S6. Binding curves obtained by microscale thermophoresis using His-tagged TEVp in presence of compounds **2c** and **2f**. For the binding curve of **2c** MST signals at 19 to 20 sec (Fnorm) were analyzed, for **2f** the initial fluorescence (IF) was analyzed. Curves were fitted using the ThermoAffinity tool at the eSPC platform.

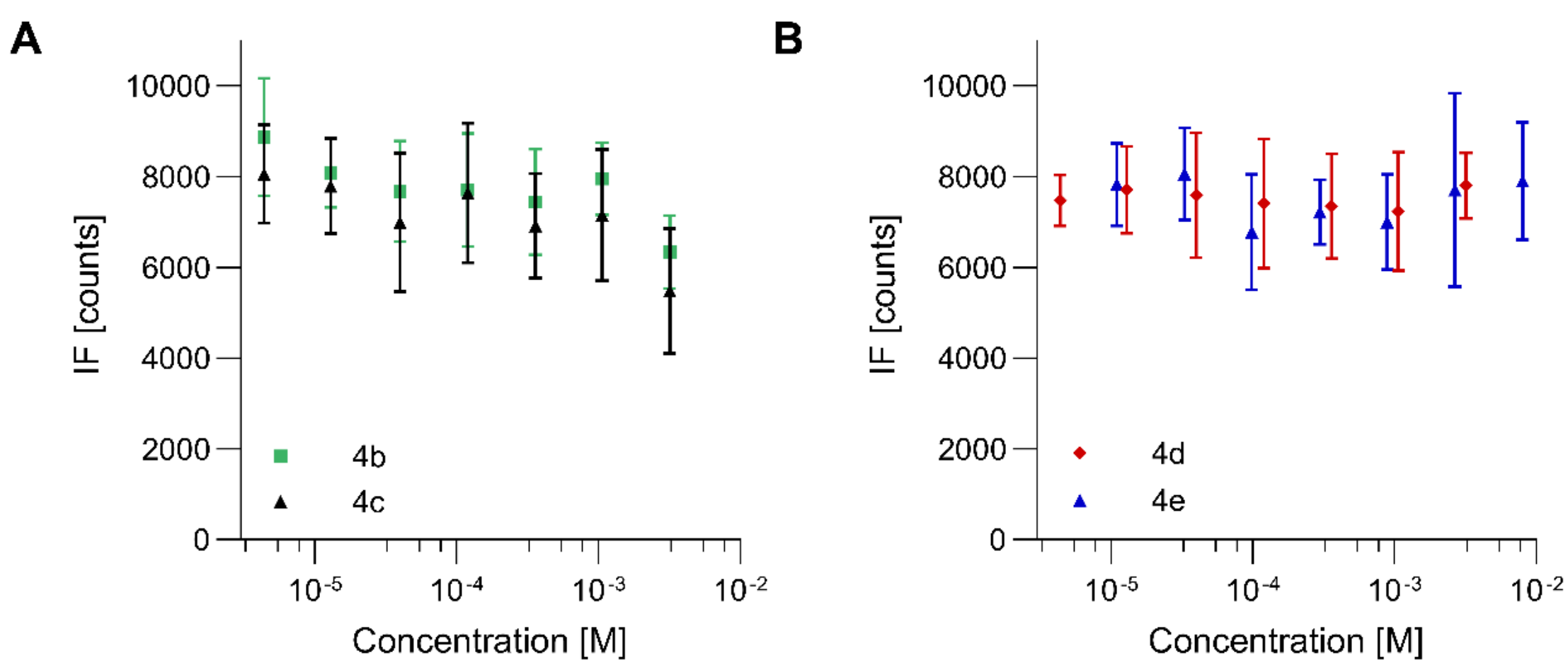

Figure S7. Binding curves obtained by microscale thermophoresis using His-tagged TEVp in presence of compounds (A) **4b** and **4c**, (B) **4d** and **4e**. For the binding curves initial fluorescence (IF) signals were analyzed without normalization. Each data point is presented as mean ±SD (n=5). Data points were derived from Figures S8-S11.

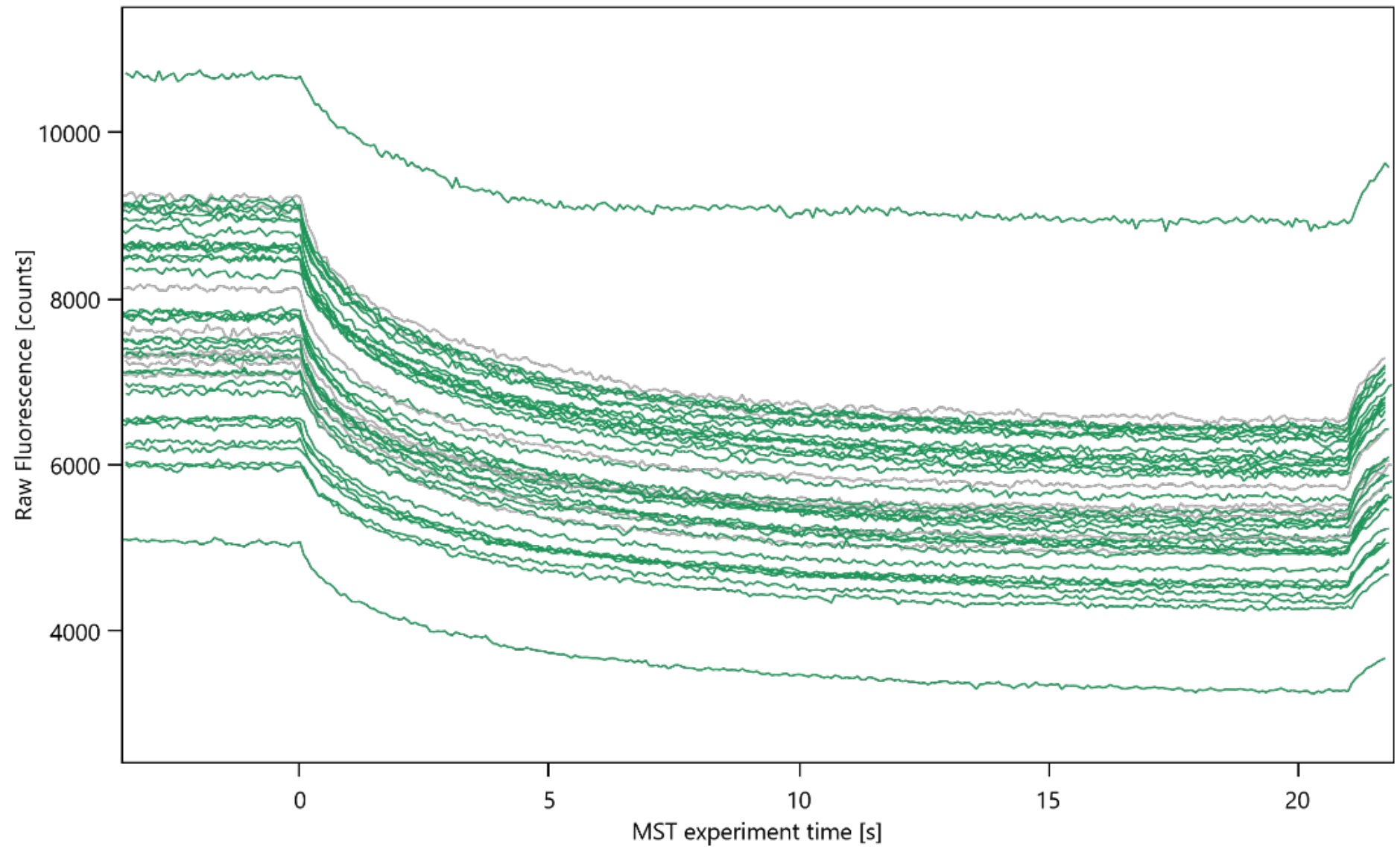

Figure S8. MST Traces obtained by microscale thermophoresis using His-tagged TEVp in presence of compound **4b**. Traces from five experiments are included in the diagram, excluded traces are colored grey.

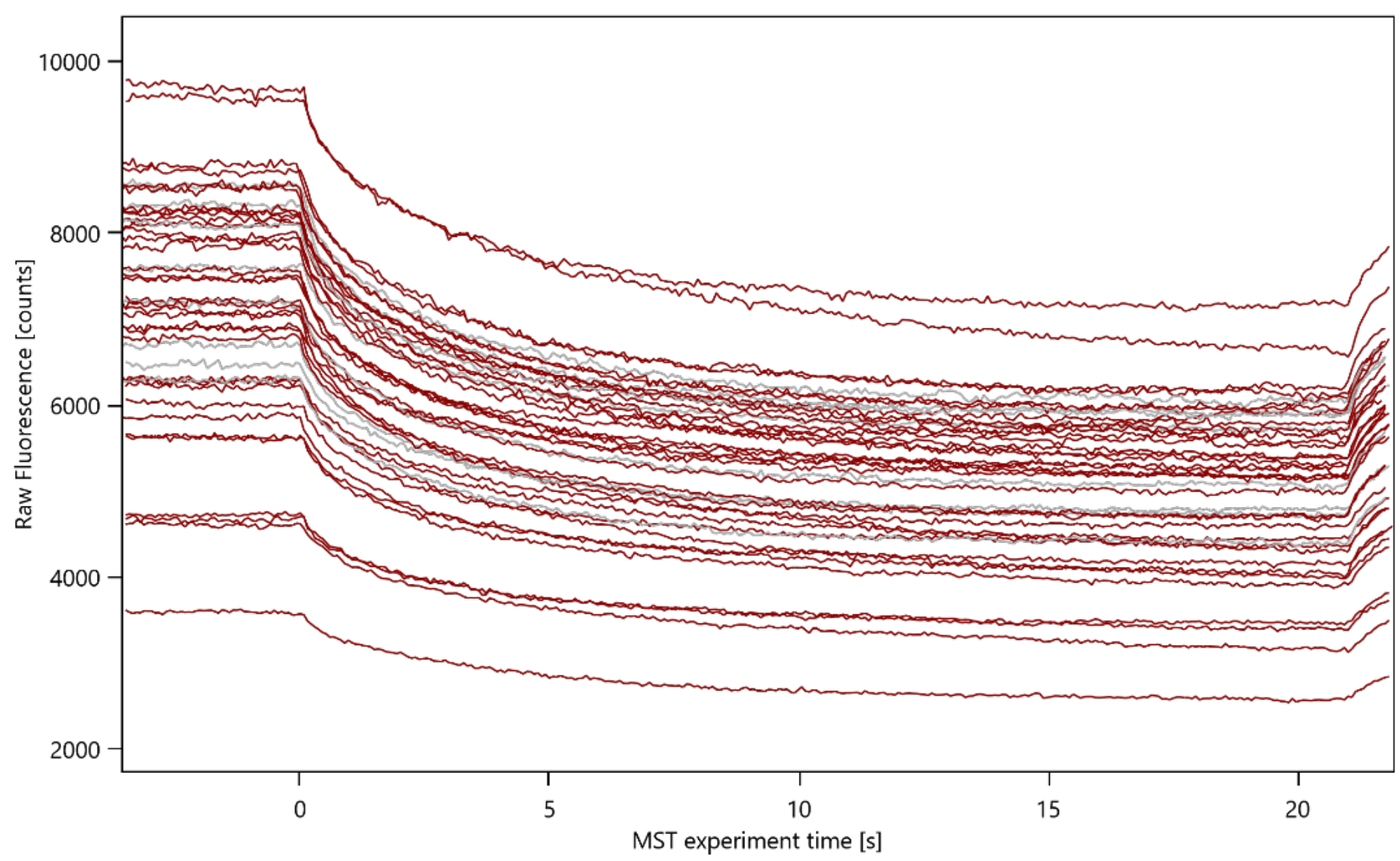

Figure S9. MST Traces obtained by microscale thermophoresis using His-tagged TEVp in presence of compound **4c**. Traces from five experiments are included in the diagram, excluded traces are colored grey.

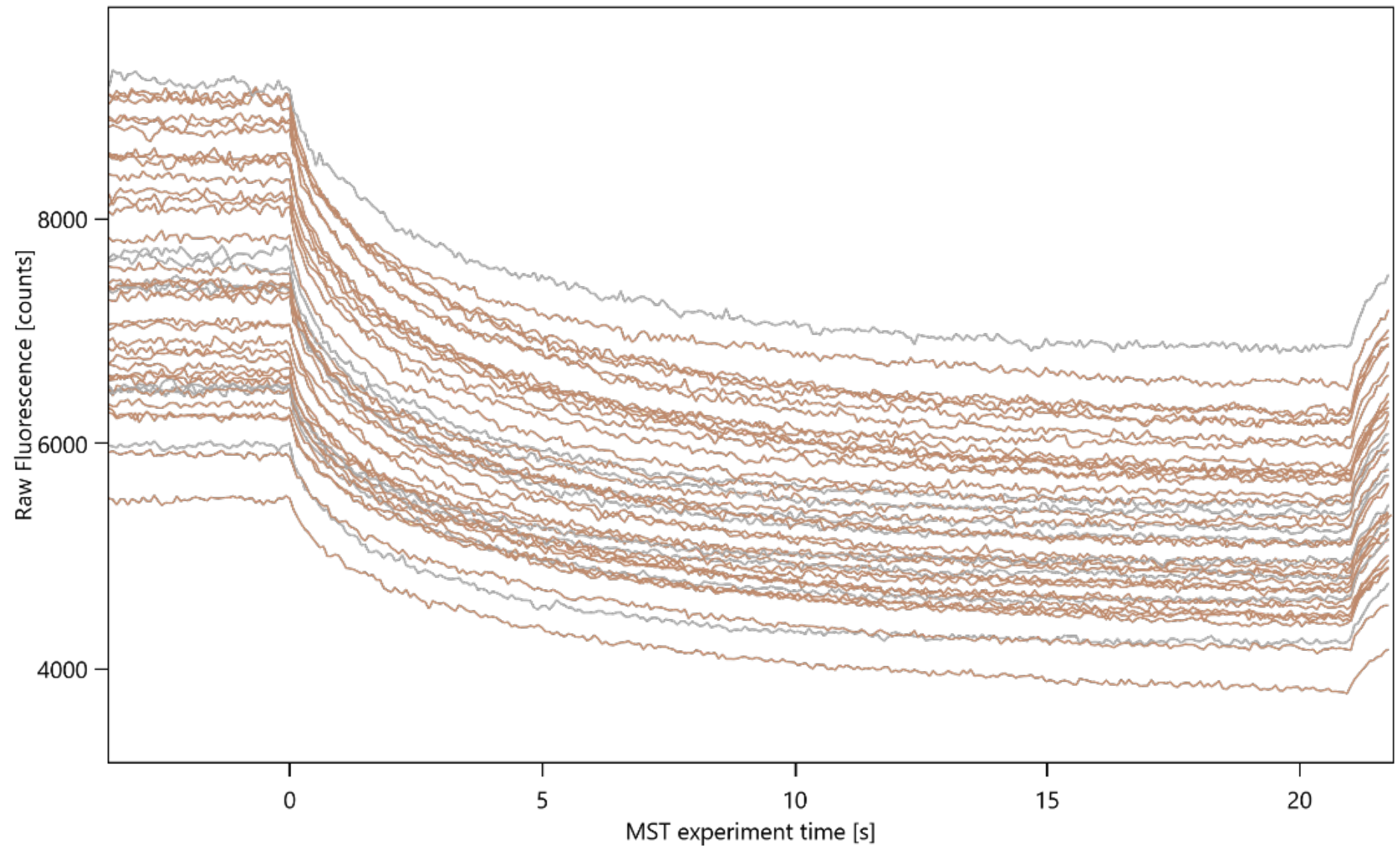

Figure S10. MST Traces obtained by microscale thermophoresis using His-tagged TEVp in presence of compound **4d**. Traces from five experiments are included in the diagram, excluded traces are colored grey.

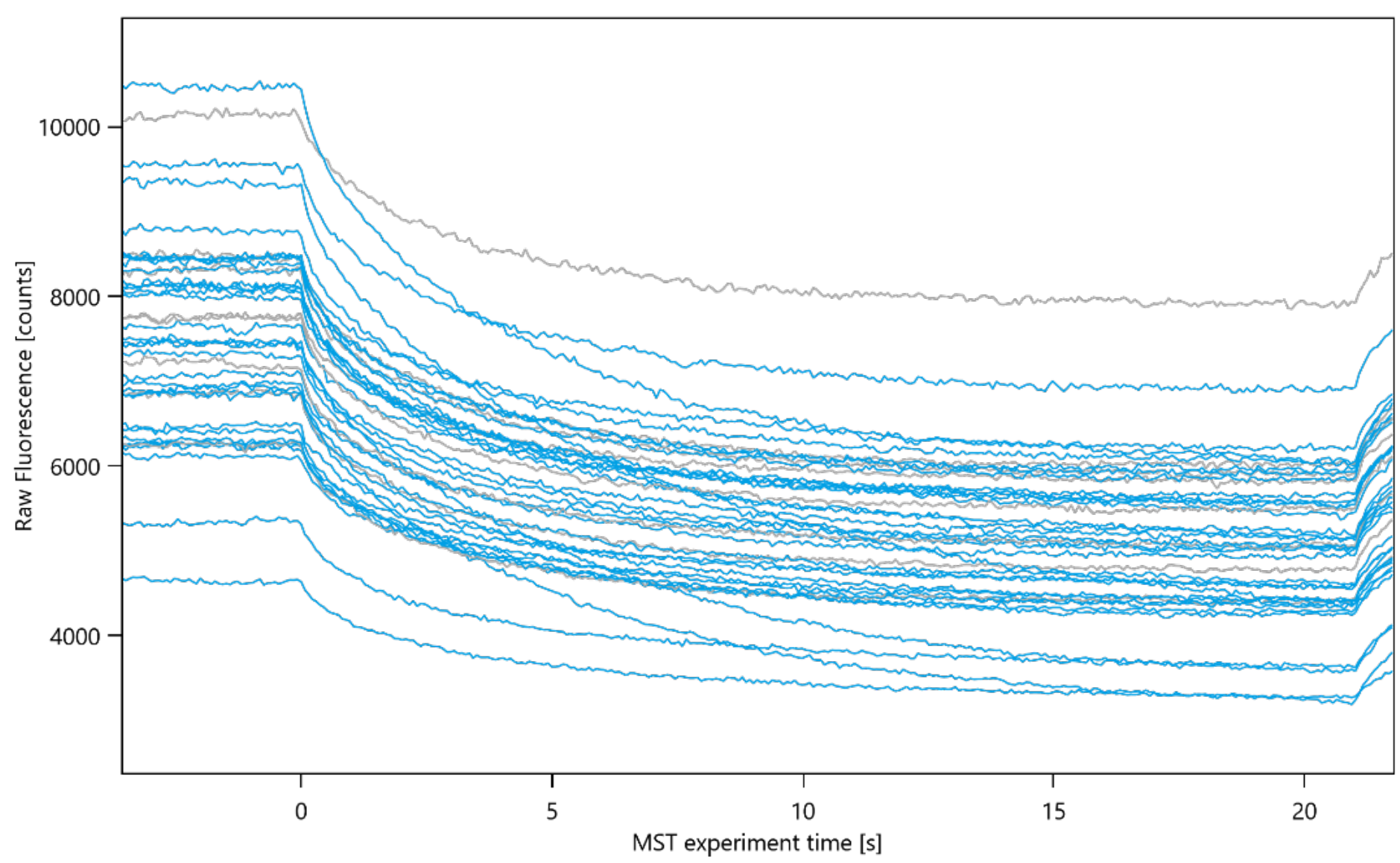

Figure S11. MST Traces obtained by microscale thermophoresis using His-tagged TEVp in presence of compound **4e**. Traces from five experiments are included in the diagram, excluded traces are colored grey.

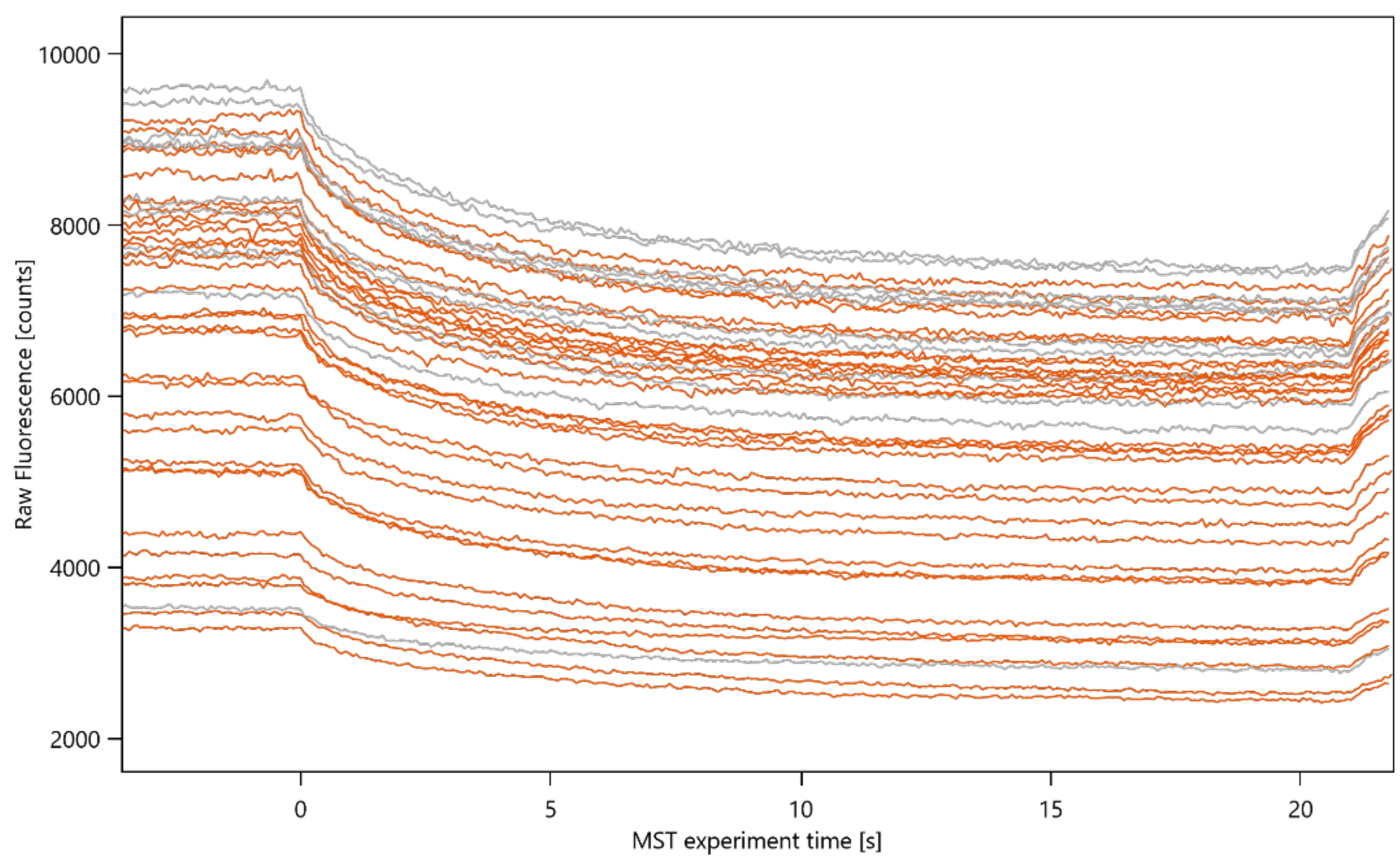

Figure S12. MST Traces obtained by microscale thermophoresis using His-tagged TolC in presence of compound **4c**. Traces from five experiments are included in the diagram, excluded traces are colored grey.

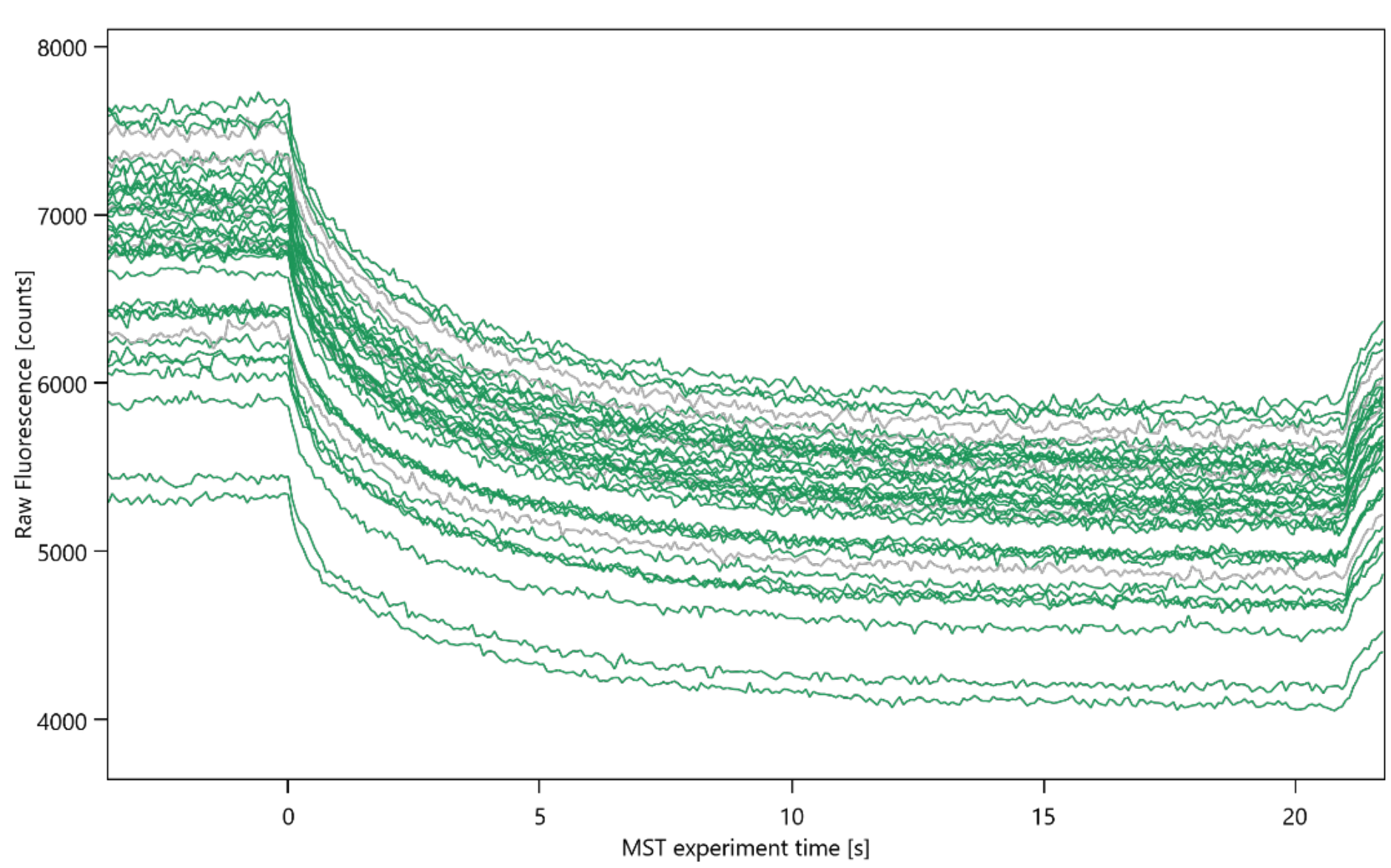

Figure S13. MST Traces obtained by microscale thermophoresis using His-tagged AcrA in presence of compound **4c**. Traces from five experiments are included in the diagram, excluded traces are colored grey.

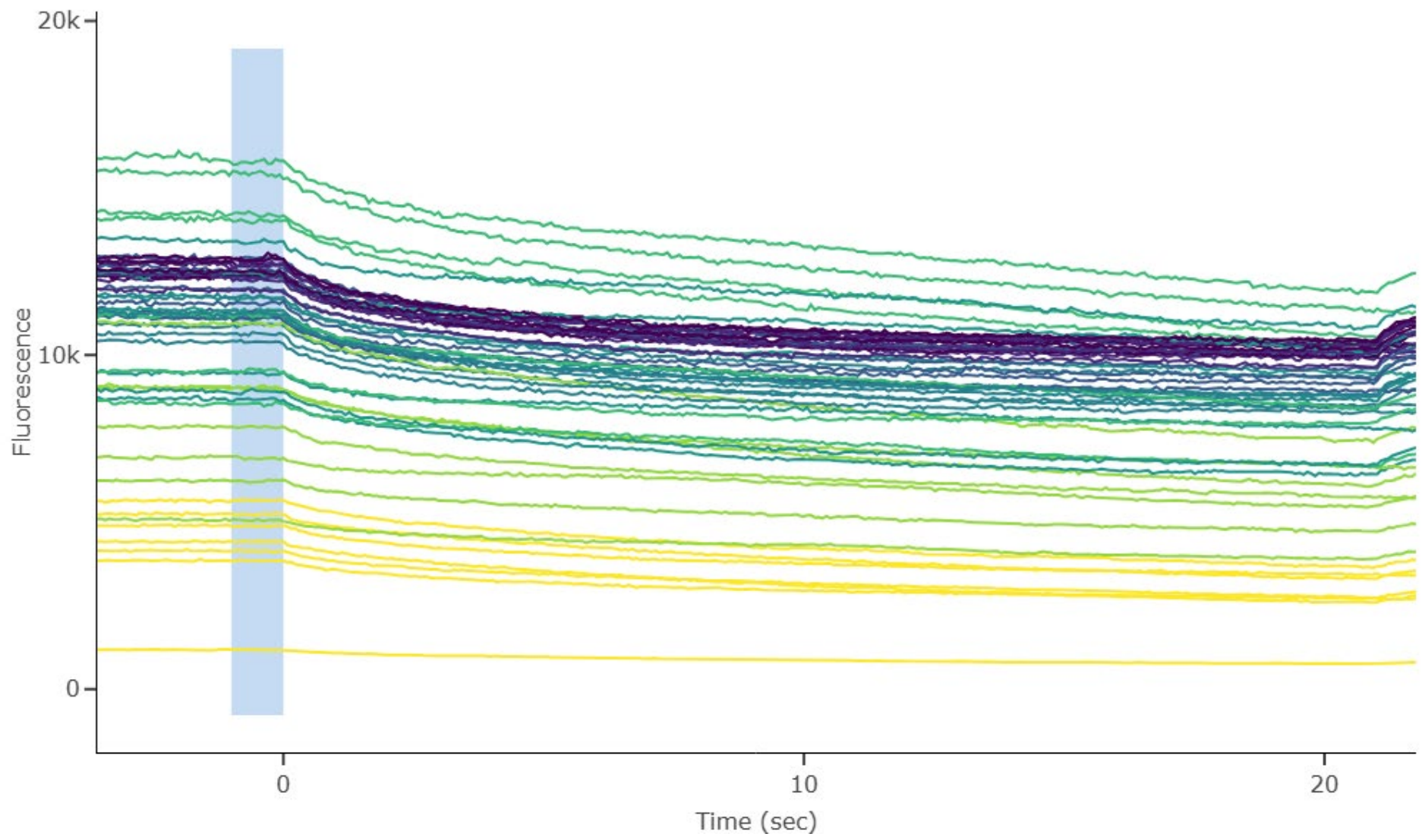

Figure S14. MST Traces obtained by microscale thermophoresis using His-tagged AcrB in presence of compound **4c**. Traces from five experiments are included in the diagram and colored according to the compound concentration (yellow (3200 µM) to violet (1.5 µM). The blue range marks the signals used for analysis.

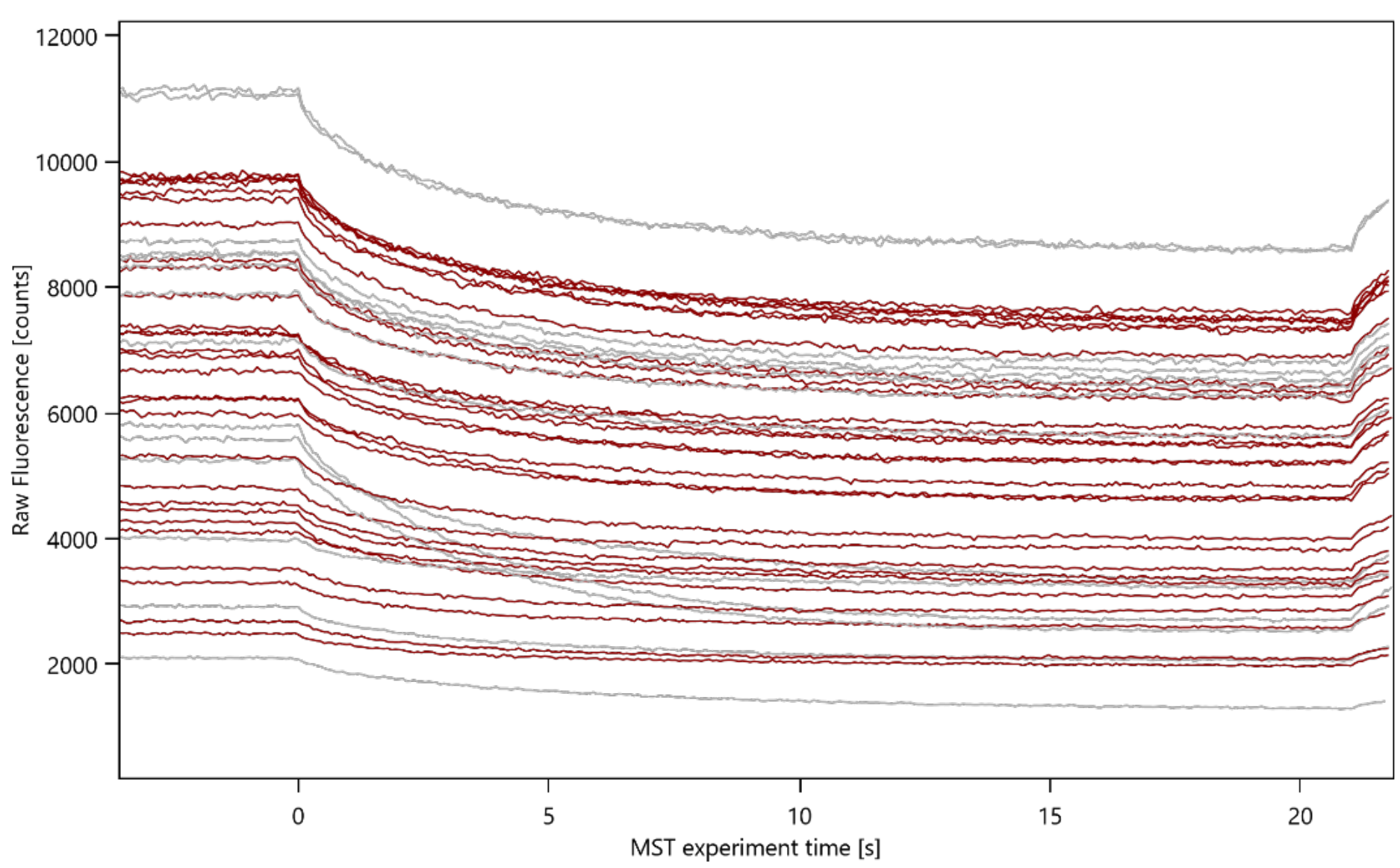

Figure S15. MST Traces obtained by microscale thermophoresis using His-tagged TolC in presence of compound **4e**. Traces from five experiments are included in the diagram, excluded traces are colored grey.

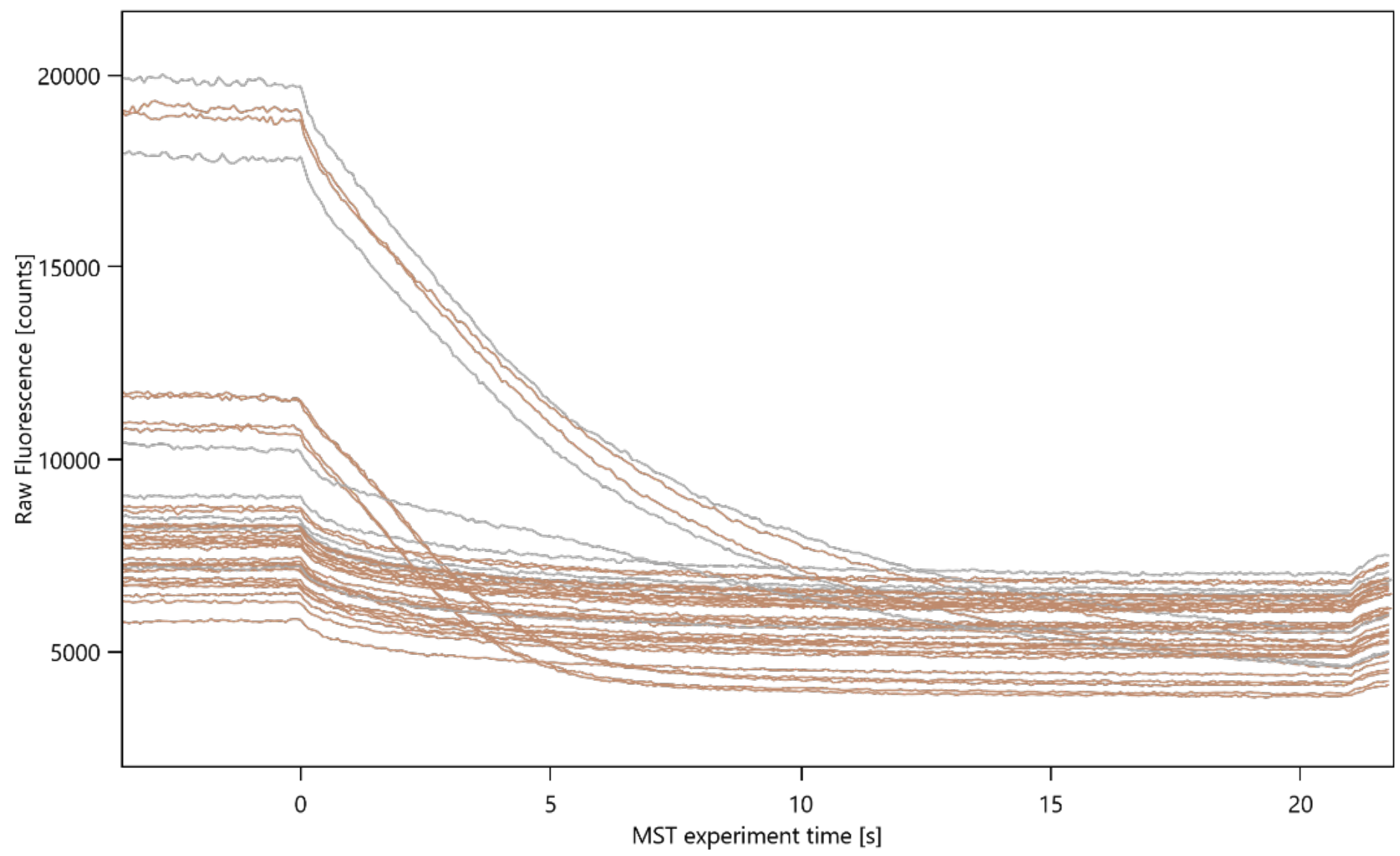

Figure S16. MST Traces obtained by microscale thermophoresis using His-tagged AcrA in presence of compound **4e**. Traces from five experiments are included in the diagram, excluded traces are colored grey.

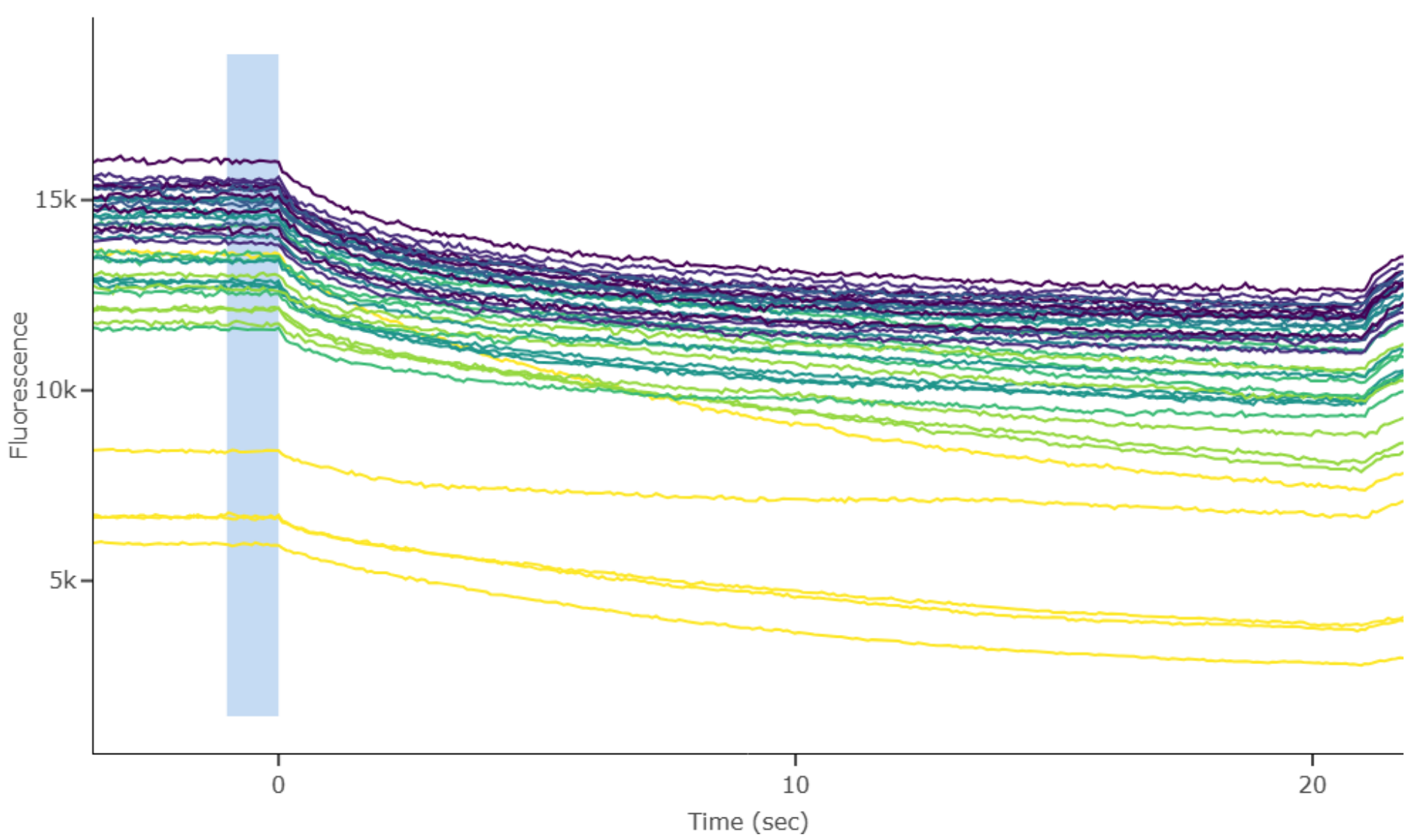

Figure S17. MST Traces obtained by microscale thermophoresis using His-tagged AcrB in presence of compound **4e**. Traces from five experiments are included in the diagram and colored according to the compound concentration (yellow (2667 µM) to violet (1.2 µM). The blue range marks the signals used for analysis.

A **B**

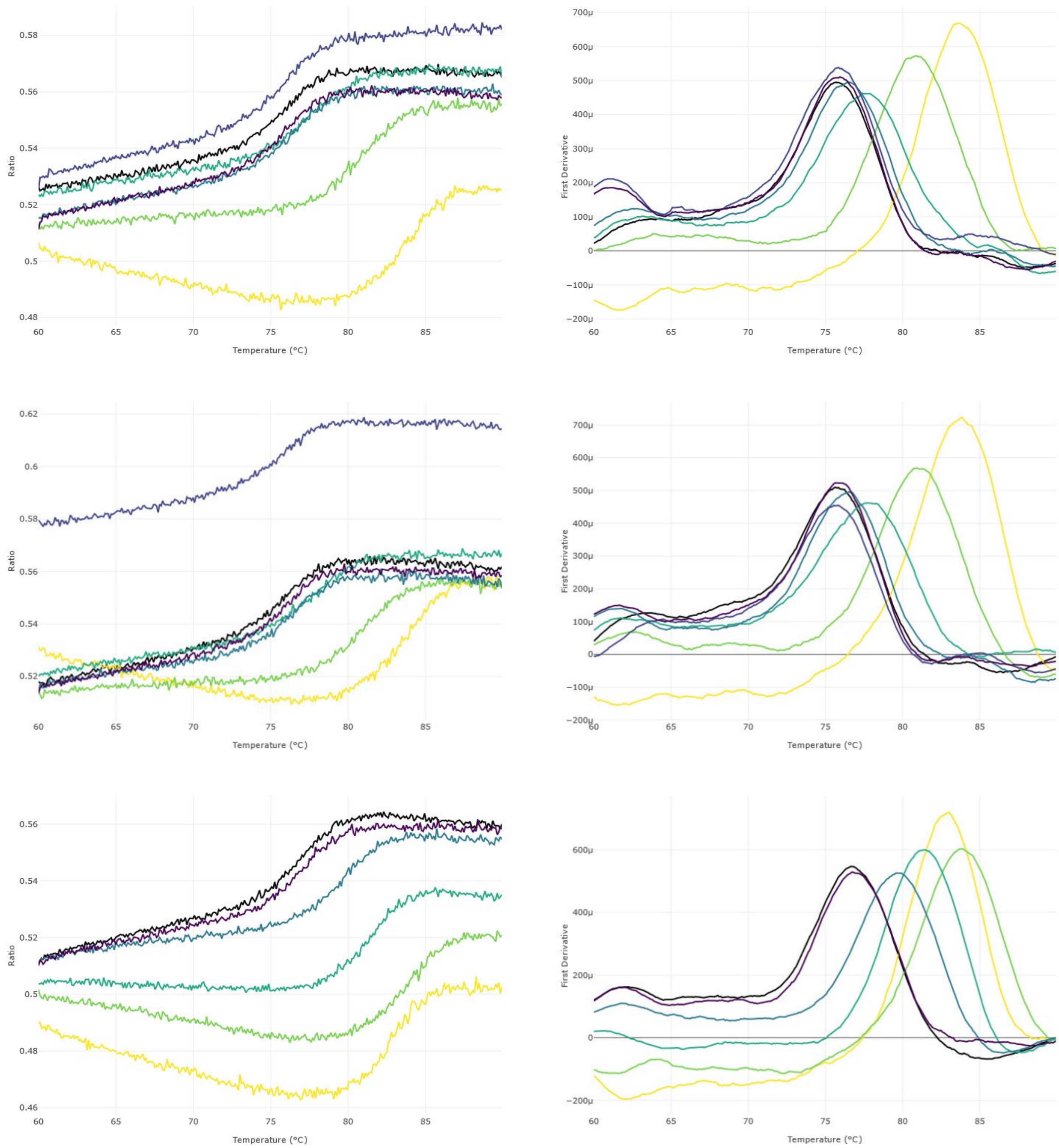

Figure S18. Melting Curves (**A**) and the respective first derivative (**B**) obtained from three repetitions of nDSF measurements using His-tagged TolC in presence of compound **4c** at different concentration (violet, 3.3 µM; dark blue, 9.9 µM; blue, 30 µM; dark green, 89 µM, light green, 267 µM; yellow, 800 µM). Curves of TolC without compound are colored black. Curves were analyzed with FoldAffinity at eSPC.

A **B**

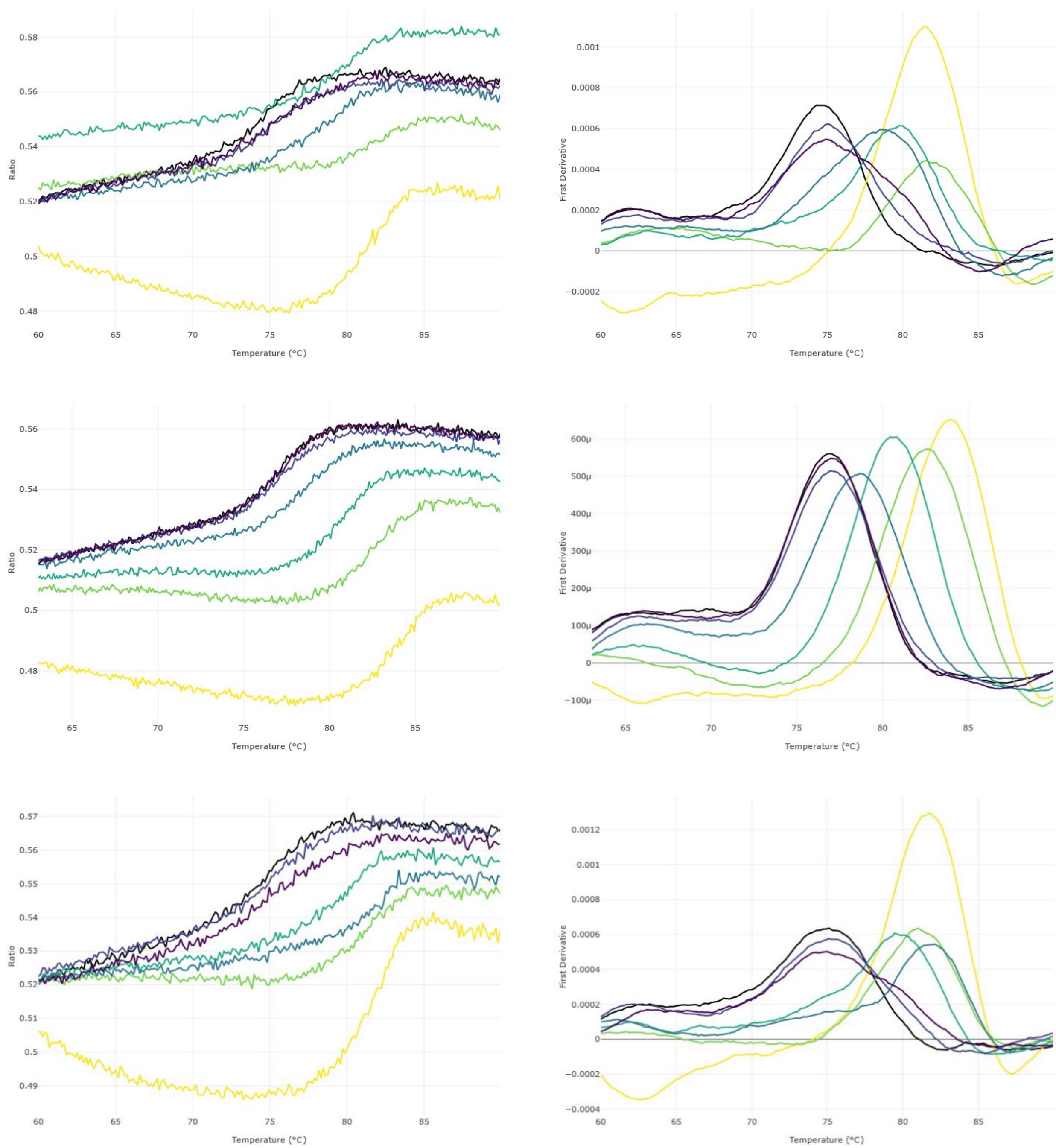

Figure S19. Melting Curves (**A**) and the respective first derivative (**B**) obtained from three repetitions of nDSF measurements using His-tagged TolC in presence of compound **4e** at different concentration (violet, 8.2 µM; dark blue, 24.7 µM; blue, 74 µM; dark green, 222 µM, light green, 667 µM; yellow, 2000 µM). Curves of TolC without compound are colored black. Curves were analyzed with FoldAffinity at eSPC.
